# AMPK-OXSR1 signaling coordinates proximal tubular sodium transport and is modulated by canagliflozin

**DOI:** 10.3389/fendo.2026.1828652

**Published:** 2026-07-31

**Authors:** Motonobu Nakamura, George Seki, Nobuhiko Satoh, Mayuko Takagi, Tomohito Mizuno, Hiroyuki Tsukada, Shoko Horita, Yusuke Sato, Haruki Kume, Masaomi Nangaku

**Affiliations:** 1 Division of Nephrology and Endocrinology, The University of Tokyo, Tokyo, Japan; 2 Department of Nephrology, Yaizu City Hospital, Shizuoka, Japan; 3 Division of Nephrology, Japan Community Healthcare Organization, Tokyo Yamate Medical Center, Tokyo, Japan; 4 Division of Nephrology, Mitsui Memorial Hospital, Tokyo, Japan; 5 Teikyo University School of Medicine, Tokyo, Japan; 6 Department of Urology, The University of Tokyo, Tokyo, Japan

**Keywords:** AMPKα2, oxidative stress-responsive kinase 1, proximal tubules, SGLT2, sodium reabsorption

## Abstract

**Introduction:**

The with-no-lysine kinase (WNK)/oxidative stress-responsive kinase 1 (OXSR1) pathway is vital for renal sodium transport in the distal nephrons as it regulates chloride cotransporters. However, its role in proximal tubule (PT) sodium transport remains unclear. We aimed to investigate whether this pathway acts as signaling convergence point involved in coordinating transepithelial sodium reabsorption using isolated human and rat PTs.

**Methods:**

Freshly isolated human and rat PTs were treated with WNK or OXSR1 inhibitors to evaluate their effects on sodium reabsorption stimulated by insulin, a peroxisome proliferator-activated receptor gamma agonist, and angiotensin II (AngII).

**Results:**

Inhibition of WNK or OXSR1 suppressed the stimulatory effects of insulin and AngII on sodium reabsorption in human and rat PTs. The sodium–glucose cotransporter 2 (SGLT2) inhibitor canagliflozin inhibited OXSR1 phosphorylation via AMP-activated protein kinase (AMPK)α1/2, thereby attenuating insulin- and AngII-induced PT Na^+^ transport, blood pressure elevation, and renal injury. Moreover, canagliflozin administration improved kidney function and increased AMPKα1/2 phosphorylation, suggesting that SGLT2-regulated OXSR1 is integral to Na^+^ transport stimulation. Incubation of PTs with different glucose concentrations and gene silencing of SGLT2 revealed that canagliflozin may activate AMPK by suppressing the transcellular glucose flux but not by reducing intracellular glucose in PTs.

**Discussion:**

These findings identify OXSR1 as a crucial signaling convergence point involved in coordinating transepithelial sodium reabsorption and demonstrate that SGLT2 regulates OXSR1 activity via AMPKα1/2.

## 1 Introduction

Recent clinical trials have demonstrated that sodium (Na)-glucose cotransporter 2 (SGLT2) inhibitors (SGLT2is) exert protective effects by slowing the progression of chronic kidney disease (CKD) and diabetic kidney disease (DKD) (1–4). Na^+^ transport in the proximal tubule (PT) is primarily regulated by the coordinated action of the Na^+^/H^+^ exchange transporter 3 (NHE3) and Na^+^/HCO_3_^-^ cotransporter (NBCe1) expressed on the luminal and basement membrane sides, respectively (5). We previously reported that insulin stimulates NBCe1 activity in the PT via the insulin receptor substrate (IRS)2/AKT2 (6), peroxisome proliferator-activated receptor gamma (PPARγ) agonists stimulate NHE3 and NBCe1 via extracellular signal-regulated kinases (ERKs) (7, 8). Moreover, angiotensin II (AngII) stimulates NHE3 and NBCe1 via ERKs (9). These results suggest the existence of master signaling convergence point governing Na^+^ transport in the PT, although the precise mechanisms remain unclear.

The with-no-lysine kinase (WNK)/STE20/SPS1-related proline/alanine-rich kinase (SPAK) or its homolog oxidative stress-responsive kinase 1 (OXSR1) pathway has recently been implicated as a master regulator of chloride (Cl) transport, which plays a crucial role in Na^+^ handling in the distal nephron. Although the expression of SPAK in the PTs is low, OXSR1 mRNA has been detected in all segments (10), and OXSR1 in the PT is closely associated with the regulation of Na-dependent phosphate transporters (11). In addition, an association with SGLT1 and NBCe1 has been reported in non-renal epithelial cells, suggesting a relationship between Na^+^ transporters expressed in the PT (10).

The WNK1/OXSR1 pathway is regulated by osmotic stress and AMP-activated protein kinase (AMPK); however, the underlying mechanisms remain unclear (12, 13). In this study, we aimed to determine whether this pathway acts as the primary signaling convergence point governing PT sodium transport using isolated human and rat PTs.

## 2 Materials and methods

### 2.1 Animal samples

All animal experiments were conducted following local institutional guidelines (authorization number: P17–070 and H-18-260).

### 2.2 Human samples

Intact human renal cortical tissue was obtained from surgical specimens based on a study protocol approved by the Research Ethics Committee of the Faculty of Medicine of the University of Tokyo (approval number: 2520–[13]). Written informed consent was obtained from all participants. The study was conducted in accordance with the principles of the Declaration of Helsinki.

Detailed materials and methods are provided in **Supplementary Methods**.

## 3 Results

### 3.1 WNK/OXSR1 localization in the nephron segment

To validate the purity of the isolated nephron segments, the mRNA expression levels of segment-specific markers were evaluated via quantitative polymerase chain reaction (**Supplementary Figures 1B–E**). *Nphs2*, a glomerulus-specific marker, was exclusively detected in the glomeruli fraction and was below the limit of detection (Not Detected; N.D.) in both the PT and the distal tubule + collecting duct (DT+CD) fractions (**Supplementary Figure 1B**). Similarly, *Slc5a2*, a PT marker, was detected solely in the PT fraction and was N.D. in the other fractions (**Supplementary Figure 1C**). Conversely, the expression of *Slc12a3*, a DT marker, and *Aqp2*, a CCD marker, was successfully detected only in the DT+CD fraction, whereas it was N.D. in the glomeruli and PT fractions (**Supplementary Figures 1D, E**). These results confirm that each nephron segment was successfully isolated with high purity and without significant cross-contamination. Following this validation, the localization of *WNK1/4*, *STK39*, and *OXSR1* was further assessed by evaluating their mRNA expression in microdissected nephron segments, including glomeruli, PTs, DTs, and CCDs from rat kidneys. *Wnk1* and *Wnk4* were most abundantly expressed in compartments containing DTs, whereas *Stk39* mRNA (encoding SPAK) was minimally expressed in the PTs. *Oxsr1* expression was comparable across all compartments (**Supplementary Figures 1F–I**). Notably, *Wnk1* and *Oxsr1* mRNA levels were upregulated in the PTs following insulin, pioglitazone (PGZ), and AngII stimulation (**Supplementary Figures 1J, K**).

### 3.2 Insulin enhances proximal tubular Na^+^ transport via the WNK1/OXSR1 axis

**Figure 1:**
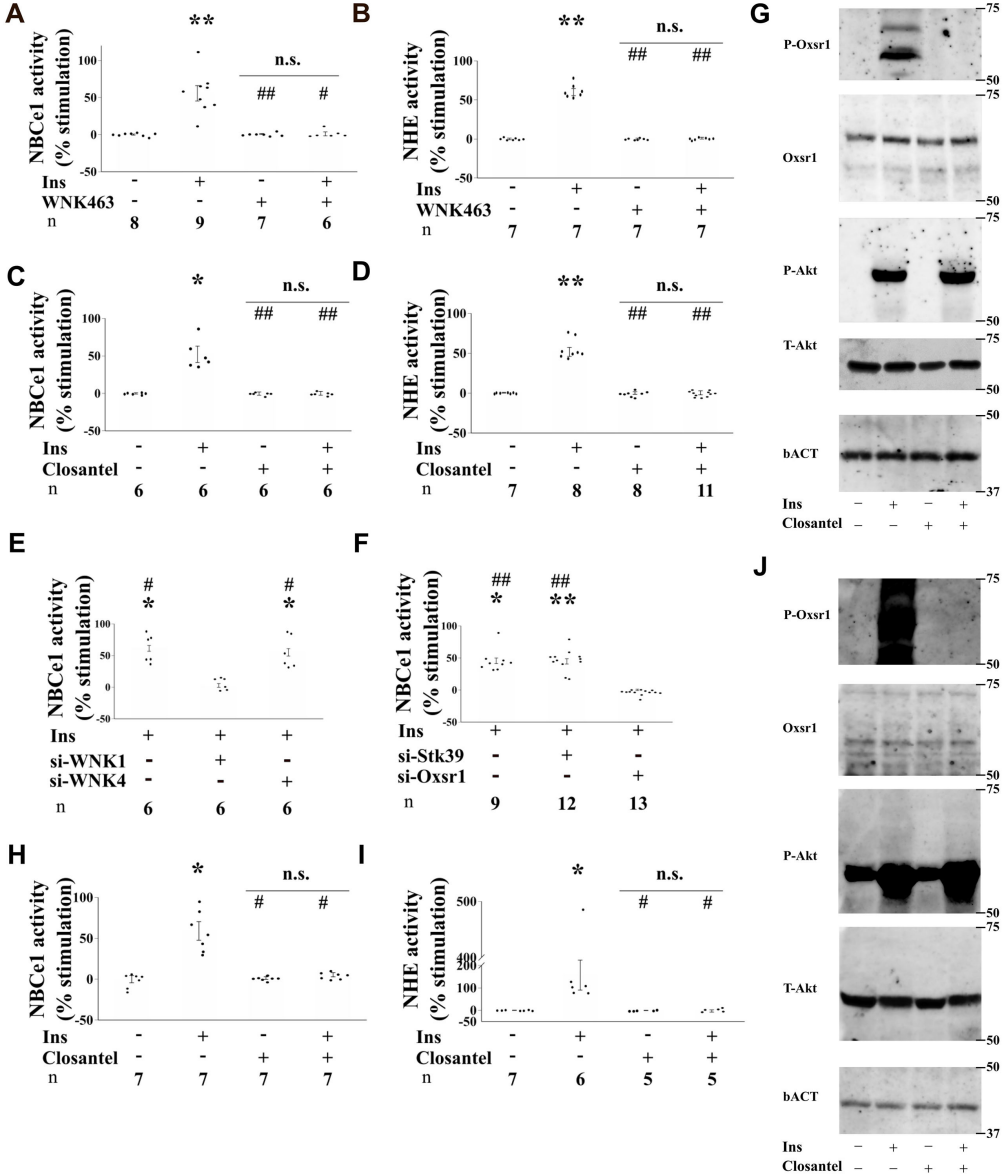
Effect of with-no-lysine kinase (WNK)1/oxidative stress–responsive kinase 1 (OXSR1) pathway inhibition on insulin-stimulated sodium transport. **(A)** Effect of 1 µM WNK463 on 1 nM insulin (Ins)–stimulated Na^+^–HCO_3_⁻ cotransporter (NBCe1) activity in isolated rat proximal tubules (PTs) (n = 6–9). **(B)** Effect of 1 µM WNK463 on 1 nM Ins-stimulated Na^+^/H^+^ exchanger (NHE) activity in isolated rat PTs (n = 7). **(C)** Effect of 10 μM closantel on 1 nM Ins-stimulated NBCe1 activity in isolated rat PTs (n = 6). **(D)** Effect of 10 μM closantel on 1 nM Ins-stimulated NHE activity in isolated rat PTs (n = 7–11). **(E)** Effect of small interfering RNA (siRNA) targeting WNK1 or WNK4 on 1 nM Ins-stimulated NBCe1 activity in isolated rat PTs (n = 6); #p<0.05 versus Ins-treated PTs with si-WNK1. **(F)** Effect of siRNA targeting *Stk39* or *Oxsr1* on 1 nM Ins-stimulated NBCe1 activity in isolated rat PTs (*n* = 9–13); ##p<0.01 versus Ins-treated PTs with si-OXSR1. **(G)** Insulin-induced AKT phosphorylation and the effect of closantel on AKT and OXSR1 phosphorylation in rat renal cortex. **(H)** Effect of 10 μM closantel on 1 nM Ins-stimulated NBCe1 activity in isolated human PTs (*n* = 7). **(I)** Effect of 10 μM closantel on 1 nM Ins-stimulated NHE activity in isolated human PTs (*n* = 5–7). **(J)** Insulin-induced AKT phosphorylation and the effect of closantel on AKT and OXSR1 phosphorylation in human renal cortex. **(A–D, H–I)** *p<0.05 versus untreated PTs; **p<0.01 versus untreated PTs; #p<0.05 versus Ins-treated PTs; ##p<0.01 versus Ins-treated PTs. **(E, F)** *p<0.05 versus control PTs treated with scrambled siRNA. “n.s.” indicates no significant difference.

We investigated the impact of WNK1 and OXSR1 on insulin-mediated activation of Na^+^ transporters in the rat PT. Insulin administration increased NBCe1 and NHE activity within the PT; this effect was completely inhibited by WNK463, a pan-WNK inhibitor (**Figures 1A, B**).

Subsequently, we analyzed the association between insulin-induced Na^+^ transporter activation and WNK1, along with its downstream effectors, OXSR1 and SPAK. Treatment with closantel, an OXSR1/SPAK inhibitor, abrogated the stimulatory effect of insulin onNBCe1 and NHE activities (**Figures 1C, D**).

We further investigated the involvement of WNK and OXSR1 using small interfering (si)RNA-mediated specific gene silencing in isolated PTs (**Supplementary Figures 2A–C**). Silencing *Wnk4* in the PT preserved the stimulatory effect of insulin, whereas silencing *Wnk1* inhibited this effect on NBCe1 activity (**Figure 1E**). Similarly, silencing *Stk39* did not alter the stimulatory effect of insulin on NBCe1 activity in PTs. In contrast, specific silencing of *Oxsr1* completely abolished insulin-induced activation of NBCe1 (**Figure 1F**).We analyzed the expression of proteins associated with insulin/AKT signaling pathways. Insulin remarkably phosphorylated AKT (Ser473) and OXSR1 (Ser325) in PT bundles collected from rat kidney cortices. Treatment with closantel suppressed OXSR1 phosphorylation without affecting insulin-induced AKT phosphorylation (**Figure 1G**; **Supplementary Figures 3A, B**). To determine whether AKT or OXSR1 acts upstream in the insulin-signaling pathway, we performed protein phosphorylation experiments using AKT inhibitor VIII, an AKT-specific inhibitor, in PT bundles isolated from rat renal cortices. Treatment with 1 μM AKT inhibitor VIII prevented insulin-induced phosphorylation of OXSR1 (**Supplementary Figures 3C–E**), indicating that AKT functions upstream of OXSR1 in the PT insulin-signaling pathway.

Moreover, we analyzed the association between insulin-induced Na^+^ transporter activation and the WNK1/OXSR1 pathway in human samples from individuals without metabolic abnormalities (**Supplementary Table 1**). Consistent with the observations in rat PTs, the stimulatory effect of insulin on Na^+^ transport activity was entirely inhibited by closantel in human samples (**Figures 1H, I**). At the protein level, closantel suppressed OXSR1 phosphorylation without affecting insulin-induced AKT phosphorylation in PT bundles from human kidney cortices (**Figure 1J**; **Supplementary Figures 3F, G**).

### 3.3 PPARγ agonists enhance PT Na^+^ transport via the WNK1/OXSR1 pathway

**Figure 2:**
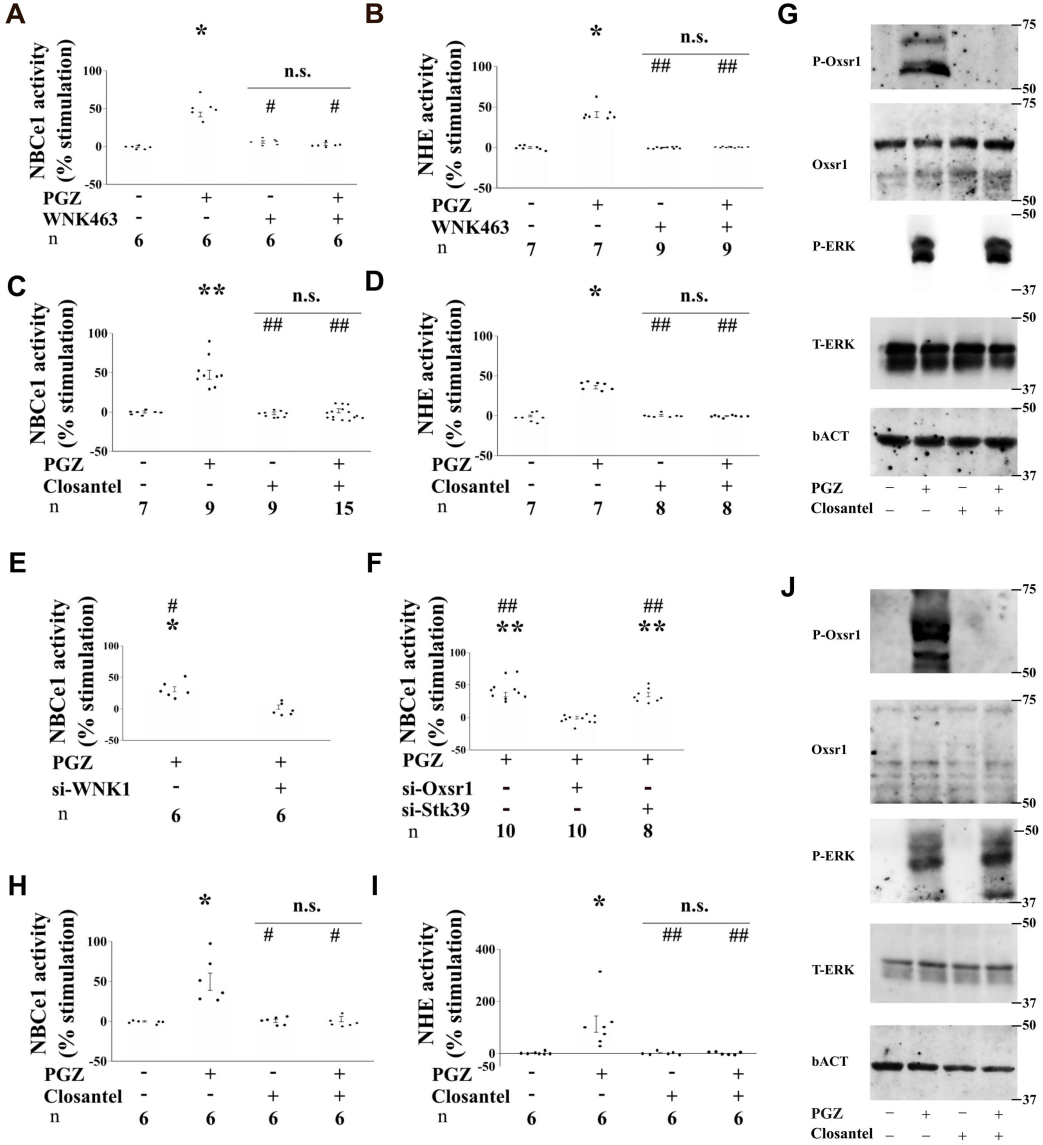
Effect of with-no-lysine kinase (WNK)1/oxidative stress–responsive kinase 1 (OXSR1) pathway inhibition on pioglitazone-stimulated sodium transport. **(A)** Effect of 1 µM WNK463 on 0.3 μM pioglitazone (PGZ)–stimulated NBCe1 activity in isolated rat proximal tubules (PTs) (n = 6). **(B)** Effect of 1 µM WNK463 on 0.3 μM PGZ-stimulated NHE activity in isolated rat PTs (n = 7–9). **(C)** Effect of 10 μM closantel on 0.3 μM PGZ-stimulated NBCe1 activity in isolated rat PTs (n = 7–15). **(D)** Effect of 10 μM closantel on 0.3 μM PGZ-stimulated NHE activity in isolated rat PTs (n = 7–8). **(E)** Effect of siRNA treatment against WNK1 on 0.3 μM PGZ-stimulated NBCe1 activity in isolated rat PTs (n = 6); *p<0.05 versus control PTs treated with scrambled siRNA; #p<0.05 versus PGZ-treated PTs with si-WNK1. **(F)** Effect of siRNA treatment against *Stk39* or *Oxsr1* on 0.3 μM PGZ-stimulated NBCe1 activity in isolated rat PTs (n = 8–10); **p<0.01 versus control PTs treated with scrambled siRNA; ##p<0.01 versus PGZ-treated PTs with si-OXSR1. **(G)** PGZ-induced ERK phosphorylation and the effect of closantel on ERK and OXSR1 phosphorylation in rat renal cortex. **(H)** Effect of 10 μM closantel on 0.3 μM PGZ-stimulated NBCe1 activity in isolated human PTs (n = 6). **(I)** Effect of 10 μM closantel on 0.3 μM PGZ-stimulated NHE activity in isolated human PTs (n = 6–7). **(J)** PGZ-induced ERK phosphorylation and the effect of closantel on ERK and OXSR1 phosphorylation in human renal cortex. **(A–D, H–I)** *p<0.05 versus untreated PTs; **p<0.01 versus untreated PTs; #p<0.05 versus PGZ-treated PTs; ##p<0.01 versus PGZ-treated PTs. “n.s.” indicates no significant difference.

PGZ stimulated NHE and NBCe1 activity (**Figures 2A, B**) and increased *Wnk1* and *Oxsr1* mRNA in rat PTs (**Supplementary Figures 1J, K**). These effects were inhibited by WNK463 and closantel (**Figures 2A–D**). In the PTs, siRNA-mediated silencing of *Wnk1* inhibited the stimulatory effect of PGZ on NBCe1 activity (**Figure 2E**).

To further define the roles of SPAK and OXSR1 in PGZ-induced Na^+^ reabsorption, we performed silencing experiments. Silencing *Stk39* did not alter the stimulatory effect of PGZ on NBCe1 activity, whereas specific silencing of *Oxsr1* abolished this effect (**Figure 2F**).

Subsequently, protein expression patterns related to these signaling pathways were examined in PT bundles isolated from rat kidney cortices. PGZ induced a marked increase in ERK and OXSR1 phosphorylation, whereas closantel suppressed OXSR1 phosphorylation without impacting PGZ-induced ERK phosphorylation (**Figure 2G**; **Supplementary Figures 4A, B**). To determine whether ERK or OXSR1 acts upstream of the PPARγ signaling pathway, we performed protein phosphorylation experiments in PT bundles isolated from rat renal cortices using PD98059, an ERK-specific inhibitor. Treatment with PD98059 (10 μM) inhibited the PGZ-induced phosphorylation of OXSR1, indicating that ERK functions upstream of OXSR1 in the PT PPARγ signaling pathway (**Supplementary Figures 4C–E**).

Similar experiments were performed using human samples. Closantel nearly completely suppressed the stimulatory effects of PGZ on NBCe1 and NHE activity (**Figures 2H, I**). At the protein level, as observed in rat samples, closantel suppressed OXSR1 phosphorylation without affecting PGZ-induced ERK phosphorylation (**Figure 2J**; **Supplementary Figures 4F, G**).

### 3.4 AngII enhances PT Na^+^ transport via the WNK1/OXSR1 axis

**Figure 3:**
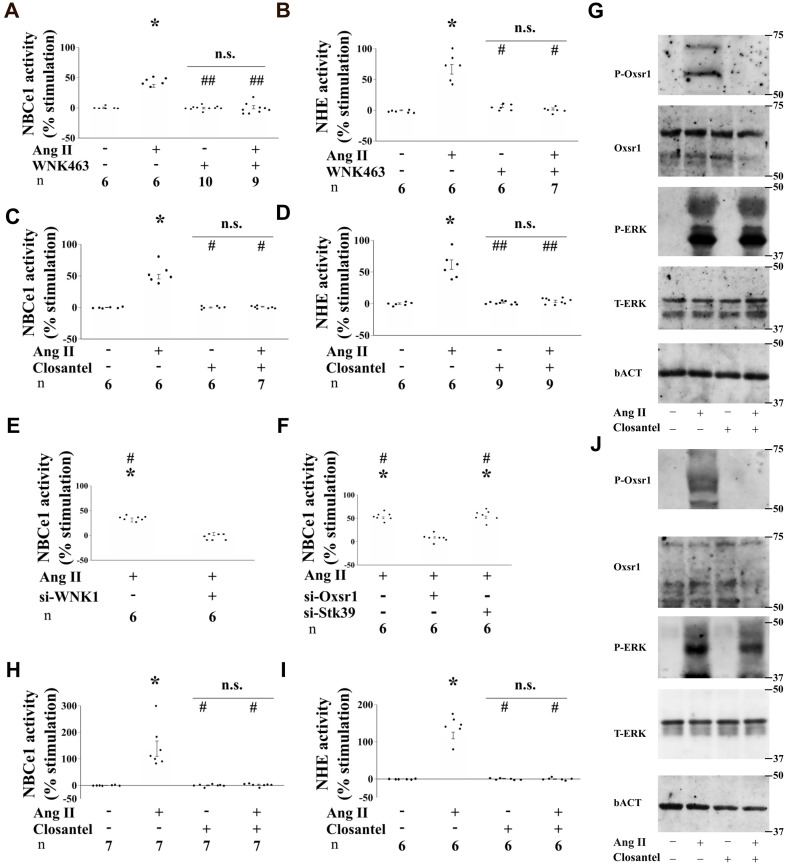
Effect of with-no-lysine kinase (WNK)1/oxidative stress–responsive kinase 1 (OXSR1) pathway inhibition on angiotensin II–stimulated sodium transport. **(A)** Effect of 1 nM WNK463 on 0.1 nM angiotensin II (AngII)–stimulated NBCe1 activity in isolated rat proximal tubules (PTs) (n = 6–10). **(B)** Effect of 1 nM WNK463 on 0.1 nM AngII-stimulated NHE activity in isolated rat PTs (n = 6–7). **(C)** Effect of 10 μM closantel on 0.1 nM AngII-stimulated NBCe1 activity in isolated rat PTs (n = 6–7). **(D)** Effect of 10 μM closantel on 0.1 nM AngII-stimulated NHE activity in isolated rat PTs (n = 6–9). **(E)** Effect of siRNA treatment against WNK1on 0.1 nM AngII-stimulated NBCe1 activity in isolated rat PTs (n = 6); #p<0.05 versus AngII-treated PTs with si-WNK1. **(F)** Effect of siRNA treatment against *Stk39* or *Oxsr1* on 0.1 nM AngII-stimulated NBCe1 activity in isolated rat PTs (n = 6); #p<0.05 versus AngII-treated PTs with si-OXSR1. **(G)** AngII-induced ERK phosphorylation and the effect of closantel on ERK and OXSR1 phosphorylation in rat renal cortex. **(H)** Effect of 10 μM closantel on 0.1 nM AngII-stimulated NBCe1 activity in isolated human PTs (n = 7). **(I)** Effect of 10 μM closantel on 0.1 nM AngII-stimulated NHE activity in isolated human PTs (n = 6). **(J)** AngII-induced ERK phosphorylation and the effect of closantel on ERK and OXSR1 phosphorylation in human renal cortex. **(A–D, H–I)** *p<0.05 versus untreated PTs; **p<0.01 versus untreated PTs; #p<0.05 versus AngII-treated PTs; ##p<0.01 versus AngII-treated PTs. **(E, F)** *p<0.05 versus control PTs treated with scrambled siRNA. “n.s.” indicates no significant difference.

AngII stimulated NHE and NBCe1 activity and increased Wnk1 and Oxsr1 mRNA in rat PTs, whereas WNK463 inhibited the stimulatory effect of AngII on Na^+^ reabsorption (**Figures 3A, B**; **Supplementary Figures 1J, K**). Similarly, closantel blocked the stimulatory effect of AngII on PT Na^+^ reabsorption (**Figures 3C, D**).

Knockdown of *Wnk1* in PTs abrogated AngII-induced activation of NBCe1 (**Figure 3E**). Similarly, silencing *Stk39* did not alter the stimulatory effect of AngII on NBCe1 activity, whereas specific silencing of *Oxsr1* completely abolished this effect (**Figure 3F**). At the protein level, AngII induced ERK and OXSR1 phosphorylation in PT bundles from rat kidney cortices, whereas closantel inhibited AngII-induced OXSR1 phosphorylation without affecting ERK phosphorylation (**Figure 3G**; **Supplementary Figures 4H, I**).

To investigate whether ERK or OXSR1 acts upstream of the AngII signaling pathway, we performed protein phosphorylation experiments in PT bundles from rat renal cortices using PD98059. Treatment with PD98059 (10 μM) prevented AngII-induced OXSR1 phosphorylation (**Supplementary Figures 4J–L**).

Consistent with the results observed in rat samples, closantel inhibited the stimulatory effect of AngII on NBCe1 and NHE activity in human PTs (**Figures 3H, I**). Closantel suppressed OXSR1 phosphorylation in protein expression, without affecting AngII-induced ERK phosphorylation in human samples, as observed in rat samples (**Figure 3J**; **Supplementary Figures 4M, N**).

### 3.5 SGLT2i regulates Na^+^ reabsorption via the WNK1/OXSR1 pathway in PTs

**Figure 4:**
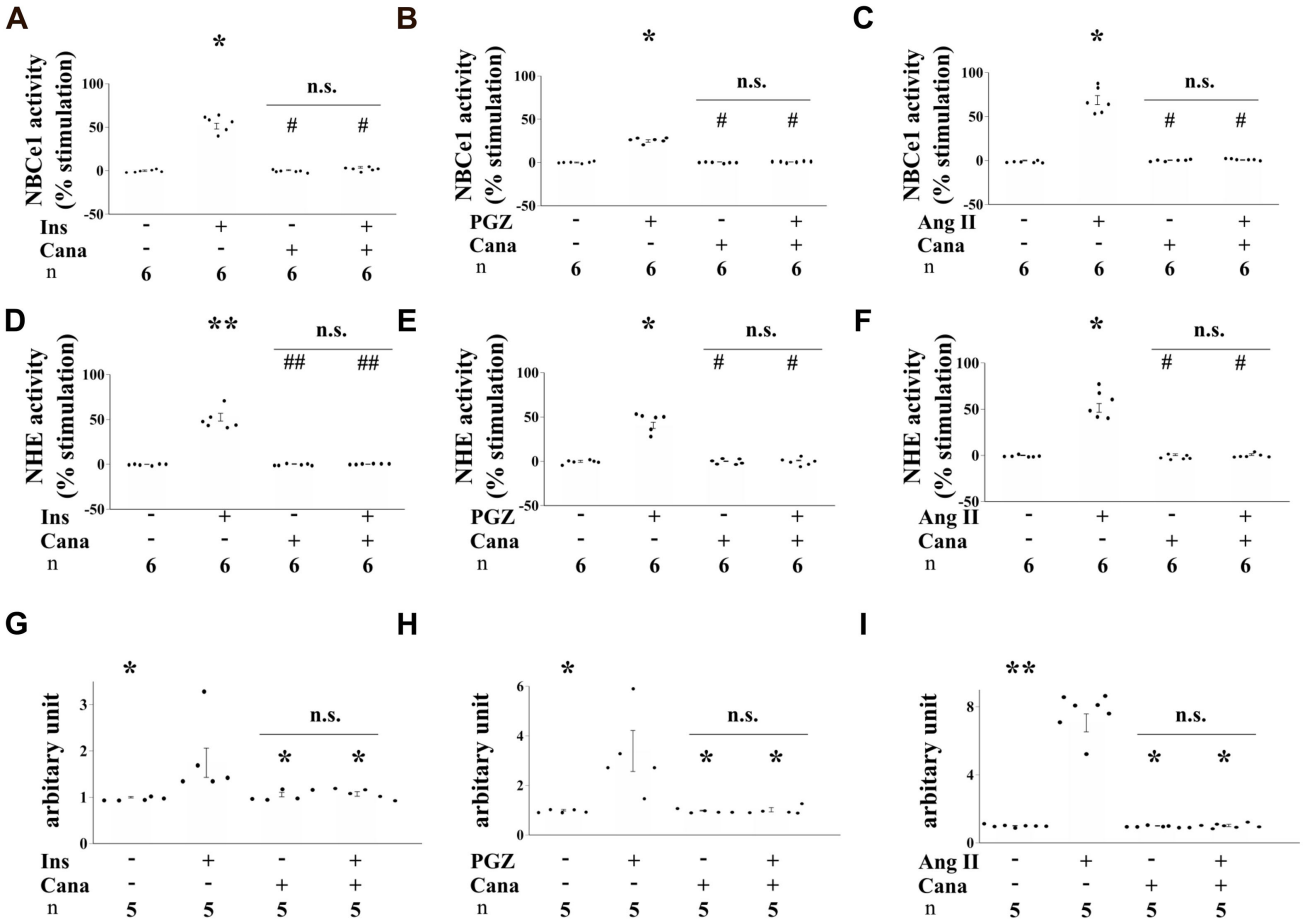
Effect of canagliflozin on sodium reabsorption enhanced by sodium transport stimulators via the WNK1/OXSR1 pathway in the proximal tubule. **(A–F)** Effects of 300 nM canagliflozin (Cana) on insulin-, pioglitazone-, or angiotensin II–stimulated NBCe1 and NHE activities in isolated rat PTs (n = 6 each). **(G)** Effect of 300 nM Cana on 1 nM Ins-stimulated OXSR1 mRNA expression in isolated rat PTs (n = 5); *p < 0.05 versus Ins-treated PTs. **(H)** Effect of 300 nM Cana on 0.3 µM PGZ-stimulated OXSR1 mRNA expression in isolated rat PTs (n = 5); *p < 0.05 versus PGZ-treated PTs. **(I)** Effect of 300 nM Cana on 0.1 nM AngII-stimulated OXSR1 mRNA expression in isolated rat PTs (n = 7); **p < 0.01 versus AngII-treated PTs. Statistical comparisons are indicated for each panel. **(A, D)**; *p < 0.05 versus untreated PTs, #p < 0.05 versus Ins-treated PTs. **(B, E)**; *p < 0.05 versus untreated PTs, #p < 0.05 versus PGZ-treated PTs. **(C, F)**; *p < 0.05 versus untreated PTs, ##p < 0.01 versus AngII-treated PTs. “n.s.” indicates no significant difference.

We further investigated the association between SGLT2 and the WNK1/OXSR1 pathway in rat PTs, as previous studies have suggested a connection between OXSR1 and SGLT or NHE (13, 14). Canagliflozin, an SGLT2i, suppressed the stimulatory effects of insulin, PGZ, and Ang II on NBCe1 activity in the PT (**Figures 4A–C**). Furthermore, it inhibited the stimulatory effects of insulin, PGZ, and AngII on NHE activity without affecting the basal activity (**Figures 4D–F**).

Then, we examined the canagliflozin-induced changes in mRNA expression in the PT. *Oxsr1* and *Wnk1* mRNA expression increased following the addition of insulin, PGZ, and AngII. Canagliflozin selectively suppressed the upregulation of *Oxsr1* mRNA, whereas no such effects were observed on *Wnk1* mRNA expression (**Figures 4G–I**; **Supplementary Figures 5A–C**).

### 3.6 WNK1/OXSR1 pathway regulators in PTs

**Figure 5:**
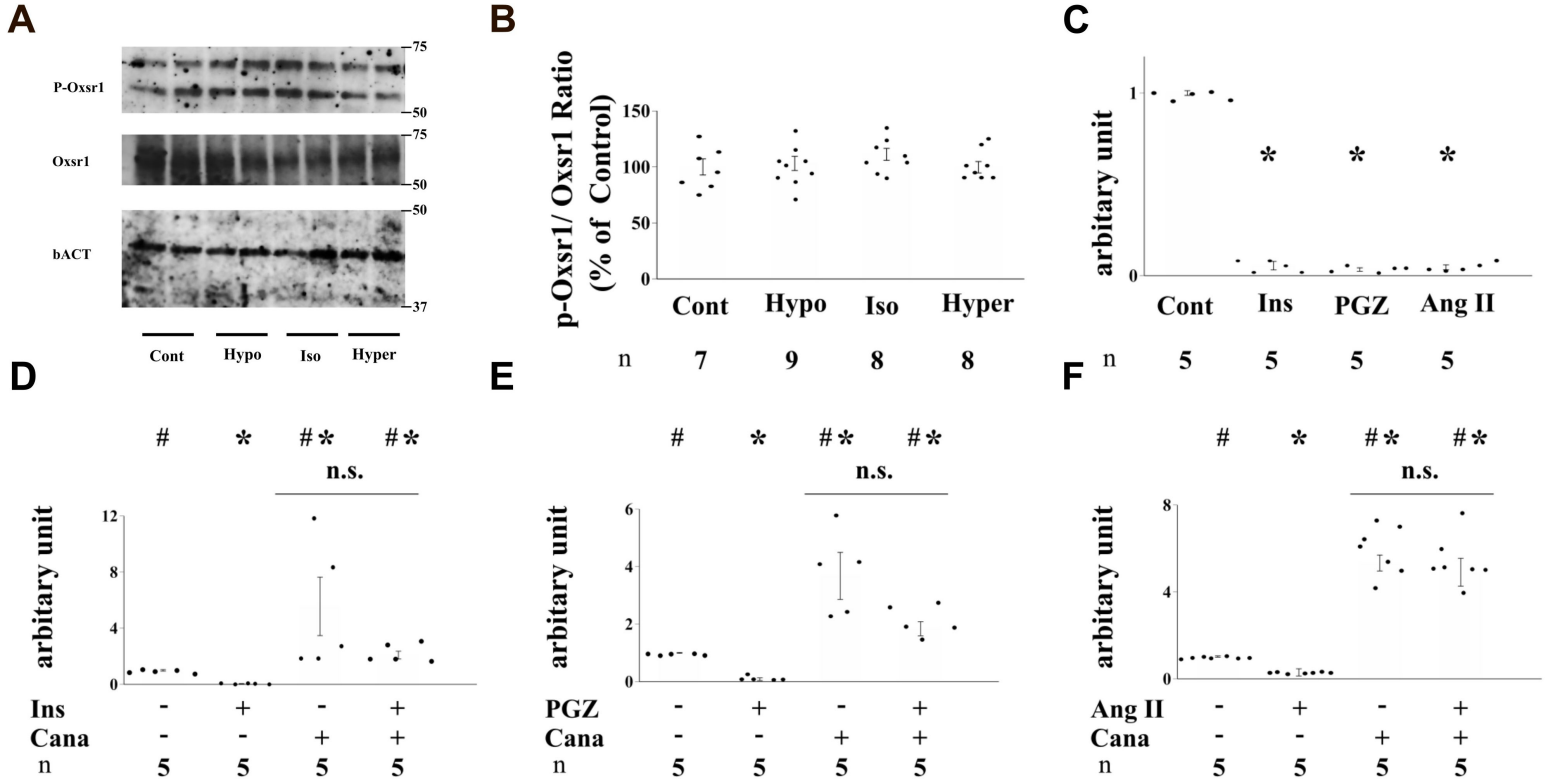
Effects of osmotic stress and metabolic factors on Prkaa2 expression in proximal tubules. **(A, B)** Effects of osmotic stress on OXSR1 phosphorylation in isolated PTs and rat renal cortex. **(C)** *Prkaa2* mRNA expression in isolated proximal tubules (PTs). *p<0.05 versus untreated PTs. (n = 5 each). **(D–F)** Regulation of *Prkaa2* mRNA expression in PTs by insulin (Ins), pioglitazone (PGZ), angiotensin II (AngII), and canagliflozin.

We examined osmotic-induced phosphorylation of OXSR1; osmotic shock did not cause OXSR1 phosphorylation in PTs (**Figures 5A, B**).

Moreover, we assessed the association between AMPK and the WNK1/OXSR1 pathway in PTs. When isolated PTs were independently cultured in media with insulin, AngII, or PGZ, no change was observed in *Prkaa1/Ampka1*, but *Prkaa2/Ampka2* mRNA expression was reduced (**Figure 5C**; **Supplementary Figure 5D**). Therefore, we focused on *Prkaa2/Ampka2* and evaluated its relationship with canagliflozin. We found that canagliflozin inhibited the insulin, AngII, and PGZ-induced suppression of *Prkaa2*/Ampka2 mRNA in PTs (**Figures 5D–F**).

**Figure 6:**
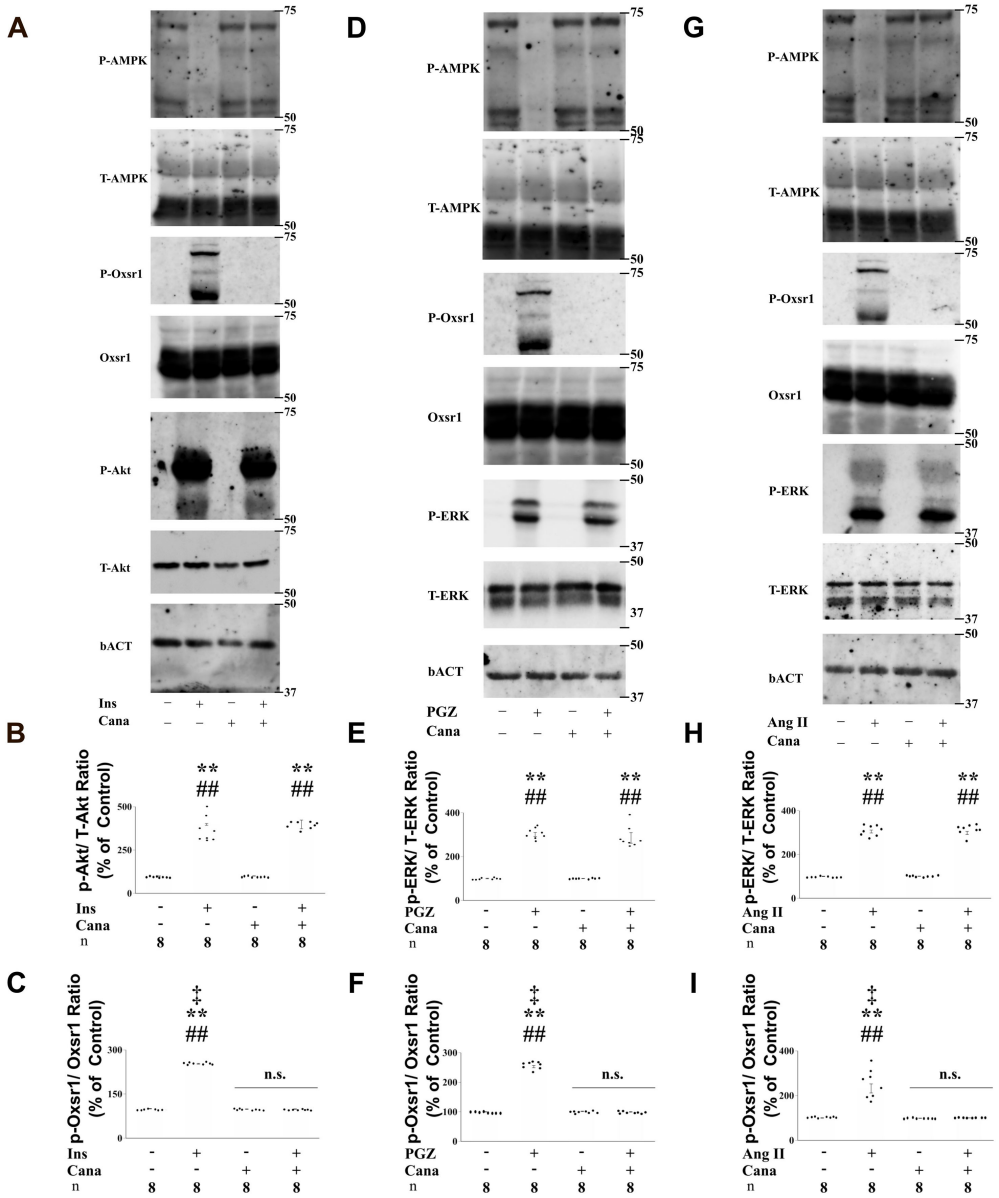
Regulation of OXSR1 and downstream kinase phosphorylation in proximal tubules. **(A)** Representative western blots of OXSR1, AMPKα1/2, and AKT phosphorylation in response to insulin. **(B)** Insulin-induced AKT phosphorylation and the effect of canagliflozin on AKT phosphorylation in rat renal cortex (*n* = 8). *p<0.05 versus insulin-untreated PTs, **p<0.01 versus untreated PTs, ^##^p<0.01 versus canagliflozin-treated PTs. **(C)** Insulin-induced oxidative stress-responsive kinase 1 (OXSR1) phosphorylation and the effect of canagliflozin on OXSR1 phosphorylation in rat renal cortex (*n* = 8). **p<0.01 versus untreated PTs, ^##^p<0.01 versus canagliflozin-treated PTs, ^‡^p<0.01 versus canagliflozin and insulin-treated PTs. **(D)** Representative western blots of OXSR1, AMPKα1/2, and ERK phosphorylation in response to PGZ. **(E)** PGZ-induced ERK phosphorylation and the effect of Cana on ERK phosphorylation in rat renal cortex (*n* = 8). *p<0.05 versus untreated PTs, **p<0.01 versus untreated PTs, ^##^p<0.01 versus Cana-treated PTs. **(F)** PGZ-induced OXSR1 phosphorylation and the effect of Cana on OXSR1 phosphorylation in rat renal cortex (*n* = 8). **p<0.01 versus untreated PTs, ^##^p<0.01 versus Cana-treated PTs, ^‡^p<0.01 versus Cana and PGZ-treated PTs. **(G)** Representative western blots of OXSR1, AMPKα1/2, and ERK phosphorylation in response to AngII. **(H)** AngII-induced ERK phosphorylation and effect of Cana on ERK phosphorylation in rat renal cortex (*n* = 8). *p<0.05 versus untreated PTs, **p<0.01 versus untreated PTs, ^##^p<0.01 versus Cana-treated PTs. **(I)** AngII-induced OXSR1 phosphorylation and the effect of Cana on OXSR1 phosphorylation in rat renal cortex (*n* = 8). *p<0.05 versus untreated PTs, **p<0.01 versus untreated PTs, ^##^p<0.01 versus Cana-treated PTs.

Building on these findings at the mRNA level, the research further investigated the effects of canagliflozin on protein phosphorylation. Similar to closantel, canagliflozin inhibited OXSR1 phosphorylation without affecting insulin-induced AKT phosphorylation in PT bundles from rat kidney cortices (**Figures 6A–C**). Additionally, it suppressed OXSR1 phosphorylation without impacting PGZ- or AngII-induced ERK phosphorylation (**Figures 6D–H**).

Furthermore, the study analyzed AMPKα1/2 phosphorylation in the same PT bundles. Insulin, PGZ, and AngII were observed to suppress AMPK phosphorylation, while canagliflozin was found to offset these inhibitory effects (**Figures 6A, D, G**; **Supplementary Figures 5E–G**). These results collectively demonstrate that canagliflozin not only prevents the suppression of *Prkaa2*/*Ampka2* at the genetic level, but also directly inhibits OXSR1 phosphorylation and restores suppressed AMPKα1/2 phosphorylation at the protein level.

### 3.7 AMPKα2 negatively regulates Na^+^ transport in PTs

**Figure 7:**
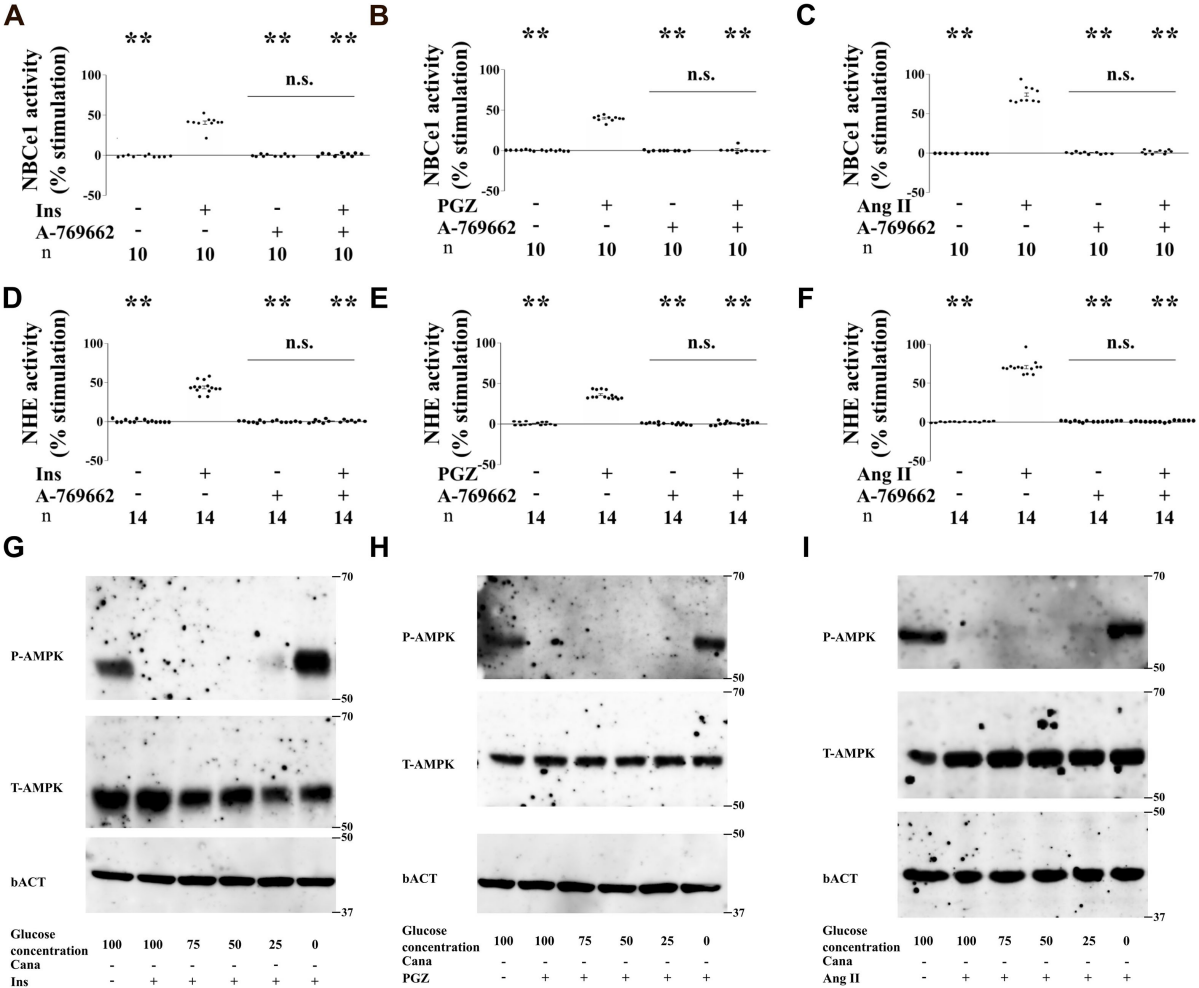
AMPK-mediated regulation of transporter activities and its modulation by glucose and canagliflozin. **(A)** Effects of the AMPK activator A-769662 on Ins-stimulated NBCe1 activity in isolated rat PTs. *p<0.05 versus Ins-treated PTs (n = 10 each). **(B)** Effects of the AMPK activator A-769662 on PGZ-stimulated NBCe1 activity in isolated rat PTs. *p<0.05 versus PGZ-treated PTs (n = 10 each). **(C)** Effects of the AMPK activator A-769662 on AngII-stimulated NBCe1 activity in isolated rat PTs. *p<0.05 versus AngII-treated PTs (n = 10 each). **(D)** Effects of the AMPK activator A-769662 on Ins-stimulated NHE activity in isolated rat PTs. *p<0.05 versus Ins-treated PTs (n = 14 each). **(E)** Effects of the AMPK activator A-769662 on PGZ-stimulated NHE activity in isolated rat PTs. *p<0.05 versus PGZ-treated PTs (n = 14 each). **(F)** Effects of the AMPK activator A-769662 on AngII-stimulated NHE activity in isolated rat PTs. *p<0.05 versus AngII-treated PTs (n = 14 each). (**G–I**) Effects of glucose concentration and canagliflozin on insulin-, PGZ-, and AngII-induced AMPK dephosphorylation in PTs. “n.s.” indicates no significant difference.

Isolated PTs cultured with the AMPK activator A-769662 inhibited the stimulatory effects of insulin, PGZ, or AngII on Na^+^ transport (**Figures 7A–F**). In contrast, the AMPK inhibitor dorsomorphin did not affect these stimulatory effects (**Supplementary Figures 5H–M**). These stimulatory effects also persisted in isolated PTs subjected to overnight incubation with siRNA targeting *Prkaa2/Ampka2*. However, the inhibitory effects of canagliflozin on the insulin-, PGZ-, and AngII-induced stimulation of Na^+^ transport were completely abolished by siRNA targeting *Prkaa2/Ampka2*, indicating that the inhibitory effect of canagliflozin was dependent on AMPK (**Supplementary Figures 5N–S**). Notably, dorsomorphin did not affect the mRNA expression of *Wnk1* and *Oxsr1*, and isolated PTs with siRNA-silenced *Prkaa2/Ampka2* expression showed similar results (**Supplementary Figures 5T–W**).

Subsequently, we examined whether the inhibitory effect of canagliflozin via AMPK originated from the decrease in intracellular glucose as previously suggested (15, 16). However, AMPK dephosphorylation by insulin, PGZ, and Ang II was preserved in the PTs incubated overnight with as low as 25 mg/dL glucose and was lost only when incubated without glucose (**Figures 7G–I**; **Supplementary Figures 5X–Z**). In addition, we observed no relationship between glucose concentration and NBCe1 or NHE activity in PTs (data not shown).

To examine whether the inhibitory effect of canagliflozin via AMPK rather originated from the suppression of transcellular glucose flux, we performed additional experiments using Cytochalasin B to selectively block basolateral GLUT transporters. This intervention strategically halts transepithelial glucose flux while maintaining apical entry via SGLT2, thereby preventing intracellular glucose depletion (17, 18). Crucially, western blot analysis revealed that Cytochalasin B abolished the insulin-induced dephosphorylation of AMPKα1/2, thereby maintaining elevated AMPK phosphorylation levels in a manner similar to canagliflozin treatment (**Supplementary Figures 6A, B**). These data suggest that restoration of AMPK activity is driven by the inhibition of transcellular glucoseflux rather than by the decrease in intracellular glucose.

### 3.8 SGLT2 is indispensable for the stimulatory effects of multiple factors on PT transport

**Figure 8:**
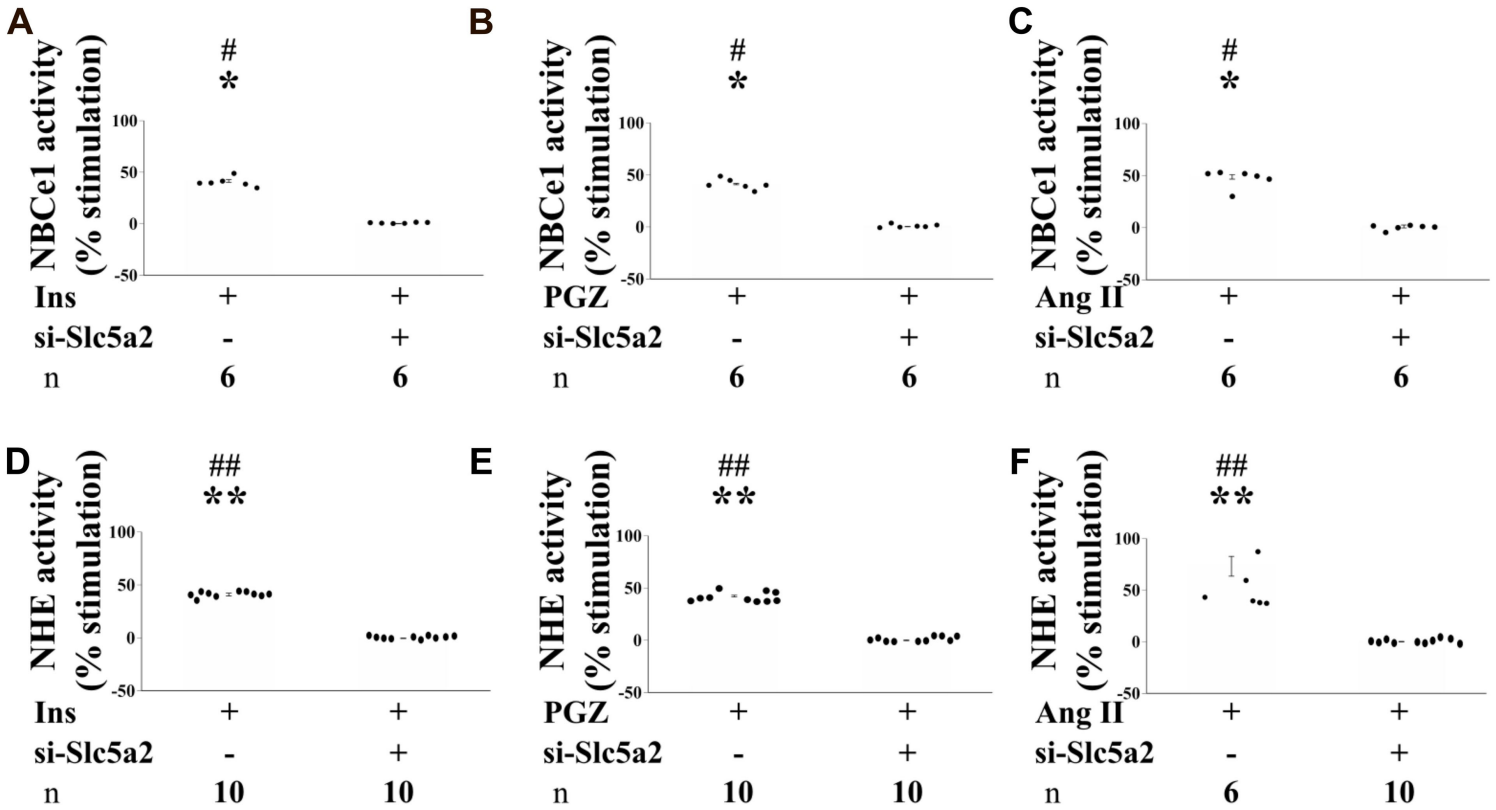
*Slc5a2* knockdown abolishes the stimulatory effects of Ins, PGZ, and Ang II on sodium transport in PTs. **(A)** Effect of siRNA treatment on 1 nM Ins-stimulated NBCe1 activity in isolated rat proximal tubules (PTs) (n = 6); *p<0.05 versus untreated PTs, #p<0.05 versus Ins-treated PTs with si-Slc5a2. **(B)** Effect of siRNA treatment on 0.3 μM PGZ-stimulated NBCe1 activity in isolated rat PTs (n = 6); *p<0.05 versus control PTs treated with scrambled siRNA; #p<0.05 versus PGZ-treated PTs with si-Slc5a2. **(C)** Effect of siRNA treatment on 0.1 nM AngII-stimulated NBCe1 activity in isolated rat PTs (n = 6); *p<0.05 versus untreated PTs, #p<0.05 versus AngII-treated PTs with si-Slc5a2. **(D)** Effect of siRNA targeting Slc5a2 on 1 nM Ins-stimulated NHE activity in isolated rat PTs (n = 10); **p<0.01 versus untreated PTs, ##p<0.05 versus Ins-treated PTs with si-Slc5a2. **(E)** Effect of siRNA treatment on 0.3 μM PGZ-stimulated NHE activity in isolated rat PTs (n = 10); **p<0.01 versus control PTs treated with scrambled siRNA; ##p<0.01 versus PGZ-treated PTs with si-Slc5a2. **(F)** Effect of siRNA treatment on 0.1 nM AngII-stimulated NHE activity in isolated rat PTs (n = 6–10); **p<0.01 versus untreated PTs, ##p<0.01 versus AngII-treated PTs with si-Slc5a2.

To further determine whether SGLT2 is required for the stimulation of sodium transporters, we performed SGLT2 knockdown using siRNA and evaluated responses to multiple stimulatory factors. SGLT2 silencing abolished the stimulatory effects of insulin, PGZ, and Ang II on NBCe1 and NHE3 activities (**Figures 8A–F**; **Supplementary Figure 2E**), indicating that SGLT2 is required for signaling pathways through which these stimuli activate proximal tubule sodium transport.

### 3.9 Dapagliflozin exerts identical inhibitory effects on sodium transport and AMPK signaling

To verify whether the observed effects of canagliflozin represent a fundamental class effect of SGLT2 inhibitors rather than a compound-specific phenomenon related to its lower SGLT2/SGLT1 selectivity, we performed confirmatory experiments using dapagliflozin, a highly selective SGLT2 inhibitor (19). We evaluated its effects on insulin-stimulated sodium transport activities and AMPK signaling in isolated PTs.

Consistent with the results obtained with canagliflozin, dapagliflozin completely abolished the insulin-stimulated activation of both NBCe1 and NHE activities in isolated PTs (**Supplementary Figures 7A, B**). Furthermore, western blot analysis revealed that dapagliflozin restored the insulin-induced suppression of AMPKα1/2 phosphorylation at the protein level (**Supplementary Figures 7C–F**). These pharmacological findings obtained using a highly selective inhibitor suggest that regulation of the AMPK-mediated sodium transport axis represents a class effect shared among SGLT2 inhibitors. Our results obtained by SGLT2-siRNA knockdown also supported this view (**Figures 8A–F**).

### 3.10 Effects of OXSR1 inhibition on Na^+^ reabsorption *in vivo*

Long-term administration of closantel in mice does not affect blood pressure but suppresses phosphorylation of Na–Cl cotransporters (NCCs) and Na–K–Cl cotransporter (NKCC) in DTs (20). OLETF rats, which exhibited clinical and pathological features similar to those of human diabetes and DKD, develop hyperinsulinemia and obesity in a week-dependent manner (21, 22). To create a DKD model, we continuously administered AngII to rats until DKD developed. These rats were then orally treated with closantel for 4 weeks.

**Figure 9:**
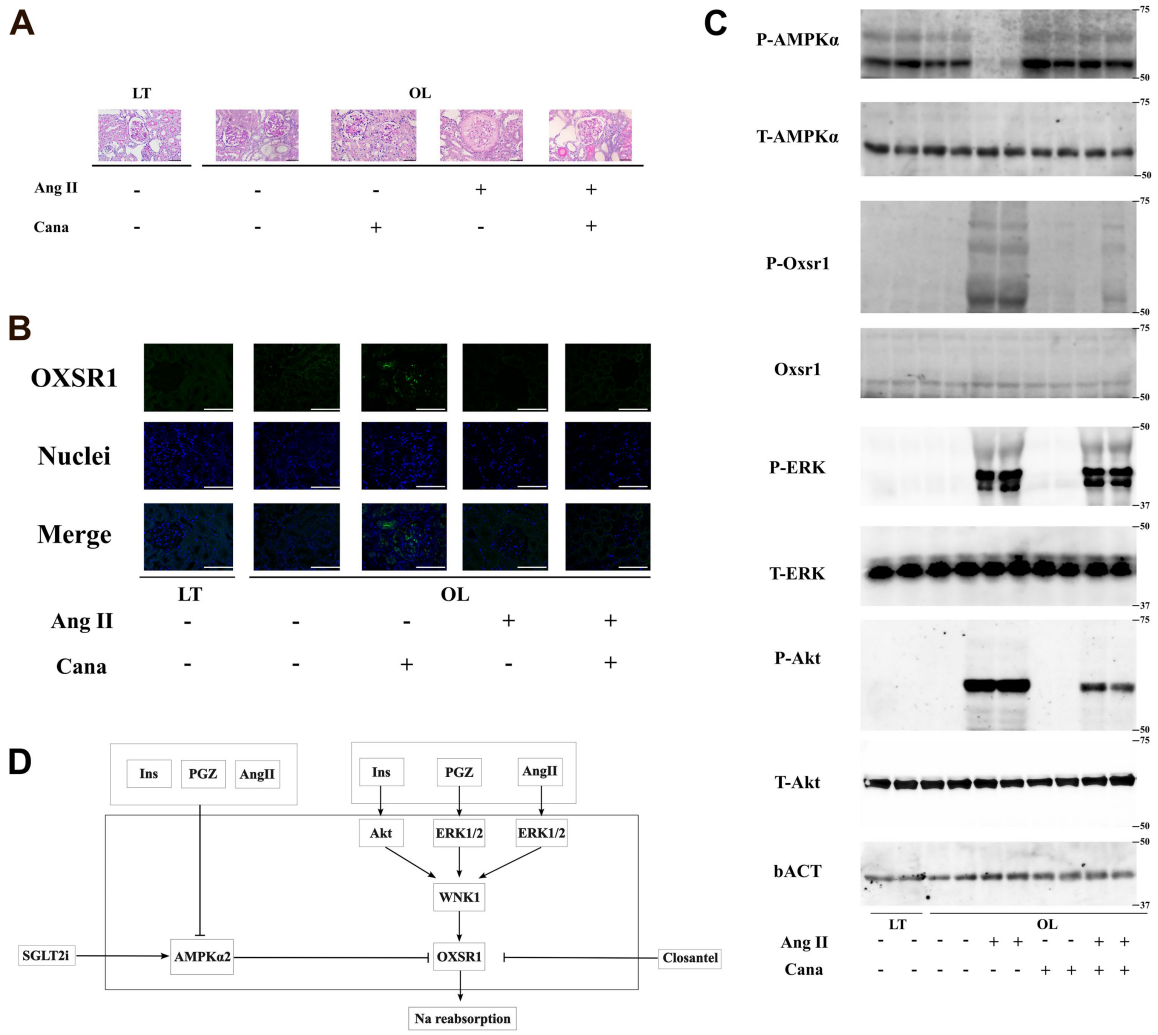
Effects of oxidative stress–responsive kinase 1 (OXSR1) inhibition on Sodium reabsorption *in vivo*. **(A)** Periodic acid–Schiff–stained rat kidney sections showing AngII-induced renal injury and its improvement with canagliflozin (scale bar, 50 μm). **(B)** Immunofluorescence staining of OXSR1 in rat renal cortex. Representative images show OXSR1 (green, top) and DAPI-stained nuclei (blue, middle). The bottom panel displays the merged image. Scale bar, 100 μm. **(C)** Renal cortical protein expression showing AngII-induced OXSR1 phosphorylation and its suppression by canagliflozin. **(D)** Schematic model of signaling pathways regulating Na^+^ reabsorption in the proximal tubule. The sequential hierarchy and upstream/downstream relationships among these signaling nodes (including AKT, ERK, AMPK, WNK1, and OXSR1) were systematically verified through independent pharmacological and genetic validation experiments.

Rats continuously receiving AngII exhibited hyperinsulinemia and kidney dysfunction; however, OLETF rats continuously treated with closantel became severely weakened, which complicated the evaluation (data not shown). Thus, to determine whether canagliflozin inhibits the OXSR1 pathway in PTs, we performed *in vivo* experiments using canagliflozin to analyze the effects of OXSR1 inhibition. Rats were randomly assigned to five groups (see Methods for details). **Table 1** presents the detailed parameters for each group. Continuous administration of AngII led to elevated blood pressure, renal dysfunction, and insulin resistance, and caused significant pathological changes consistent with DKD, including marked glomerular expansion, glomerulosclerosis, and tubular damage. Treatment with canagliflozin markedly improved these conditions, resulting in lower body weight and blood pressure and improved insulin resistance. Canagliflozin markedly ameliorated the renal pathology, with a notable reduction observed in glomerular expansion, glomerulosclerosis, and tubular damage (**Figure 9A**; **Table 1**).

**Table 1:** Effects of 4-week canagliflozin administration, combined with a high-salt diet and continuous angiotensin II infusion, on biological parameters in rats.

Rat strain Unilateral nephrectomy AngII canagliflozin	LETO + - -	OLETF + - -	OLETF + + -	OLETF + - +	OLETF + + +
N	7	7	9	7	7
BW (g)	318.2 ± 8.2	420.2 ± 7.3^§§^	413.0 ± 7.3	316.1 ± 7.3 **^##^	309.3 ± 8.5 **^##^
Heart weight (g/kg BW)	3.6 ± 0.1	4.0 ± 0.2	4.7 ± 0.2	3.8 ± 0.3#	3.5 ± 0.3^##^
UV (mL/min/kg BW)	0.15 ± 0.22	0.28 ± 0.03^§§^	0.13 ± 0.03**	0.24 ± 0.03#	0.34 ± 0.04^##^
sBP (mmHg)	116.9 ± 3.2	119.3 ± 5.8	187.9 ± 5.1**	119.5 ± 5.4^##^	145.2 ± 6.3*^##†^
dBP (mmHg)	72.4 ± 2.8	73.6 ± 2.7	105.2 ± 2.4**	72.0 ± 2.4^##^	80.2 ± 2.9^##^
ACR (g/g Cre)	0.012 ± 0.001	0.04 ± 0.01^§§^	0.11 ± 0.01**	0.02 ± 0.01^##^	0.02 ± 0.01^##^
BUN (mg/dL)	19.6 ± 0.6	26.3 ± 7.3^§^	58.1 ± 6.4*	17.2 ± 6.8^##^	44.2 ± 7.9
Cre (mg/dL)	0.45 ± 0.01	0.45 ± 0.26	1.25 ± 0.22	0.55 ± 0.24	0.45 ± 0.26
CCre (mL/min/kg BW)	8.01 ± 0.48	8.11 ± 0.52	3.03 ± 0.46**	8.72 ± 0.49^##^	8.93 ± 0.56^##^
Serum Na (mmol/L)	147.4 ± 0.8	151.1 ± 1.6	148.6 ± 1.4	147.5 ± 1.5	144.5 ± 1.7
Serum K (mmol/L)	4.7 ± 0.1	4.9 ± 0.2	4.8 ± 0.2	4.6 ± 0.2	3.9 ± 0.3*
Serum Cl (mmol/L)	106.9 ± 1.0	114.9 ± 2.1^§^	105.9 ± 1.9*	106.0 ± 2.0*	101.7 ± 2.3**
FBG (mg/dL)	85.4 ± 6.3	98.1 ± 6.3^§§^	136.4 ± 5.5**	89.0 ± 5.9^##^	93.2 ± 6.8^##^
Fasting insulin level (µU/mL)	4.21 ± 0.18	4.67 ± 0.78	9.50 ± 0.68**	4.00 ± 0.73^##^	3.98 ± 0.84^##^
HOMA-IR	0.89 ± 0.04	1.14 ± 0.45^§^	3.37 ± 0.40**	0.88 ± 0.42^##^	0.91 ± 0.49^##^
HCO3- (mmol/L)	23.8 ± 0.6	27.5 ± 0.9^§§^	34.5 ± 0.8**	24.3 ± 0.8*^##^	22.2 ± 0.9**^##^
Kidney weight (g/Kg BW)	5.94 ± 0.10	6.74 ± 0.23^§§^	8.83 ± 0.21**	6.90 ± 0.22^##^	7.32 ± 0.25^##^
Glomerular Sclerosis index	1.10 ± 0.04	1.45 ± 0.05^§§^	3.19 ± 0.04**	1.32 ± 0.05^##^	1.47 ± 0.05^##^
Tubular injury score	0.11 ± 0.06	0.18 ± 0.04	1.13 ± 0.04**	0.14 ± 0.04^##^	0.20 ± 0.04^##^
Glomerular diameter (µm)	87.88 ± 5.64	87.39 ± 3.45	141.93 ± 3.04**	87.18 ± 3.45^##^	97.08 ± 3.45^##^
OXSR1 intensity (AU)	2695 ± 172	2779 ± 231	3660 ± 656	2791 ± 138	2752 ± 307
NBCe1intensity (AU)	12360 ± 1059	10861 ± 1263	11561 ± 1114	9241 ± 1263	10760 ± 1263
NHE3 intensity (AU)	12377 ± 397	12104 ± 220	11869 ± 194	12104 ± 220	11320 ± 220
SGLT2 intensity (AU)	14343 ± 46	14262 ± 99	14241 ± 88	14471 ± 99	14285 ± 99

Data represent least squares means ± standard error of the mean (SEM) for OLETEF groups and arithmetic means ± SEM for the LETO group.

Statistical significance among the four OLETF groups was determined using two-way analysis of variance (ANOVA) followed by Tukey’s HSD test. ^*^p < 0.05, ^**^p < 0.01 versus OLETF vehicle; ^#^p < 0.05, ^##^p < 0.01 versus OLETF + AngII; ^†^p < 0.05, ^‡^ p < 0.01 versus OLETF + canagliflozin. Significant differences between LETO and OLETF rats with unilateral nephrectomy were determined using Student’s t-test and are indicated by ^§^p < 0.05, ^§§^p < 0.01.

To quantify fluorescence intensity, 8–10 proximal tubules per rat were randomly selected from the renal cortex. The fluorescence intensity per square micrometer of the proximal tubule cells was measured using ImageJ software (NIH, Bethesda, MD, USA) and expressed in arbitrary units (AU). Two-way ANOVA revealed no significant differences among the groups for NHE3, NBCe1, and SGLT2.

LETO, Long-Evans Tokushima Otsuka rats; OLETF, Otsuka Long-Evans Tokushima Fatty rats; ACR, albumin creatinine ratio; BUN, blood urea nitrogen; BW, body weight; Cre, creatinine; FBG, Fasting blood glucose; HOMA-IR, Homeostatic model assessment of insulin resistance; sBP, systolic blood pressure; UV, urinary volume; NBCe1, Na^+^-HCO_3_^−^ cotransporter 1; NHE3, Na^+^/H^+^ exchanger 3; SGLT2, Sodium glucose cotransporter 2.

We assessed the localization of OXSR1 in the renal cortices of OLETF rats treated continuously with AngII. OXSR1 expression increased in the DTs, PTs, and glomeruli of these rats (**Figure 9B**; **Table 1**).

No differences were observed in the expression of NHE3, NBCe1, or SGLT2 between the groups (**Supplementary Figures 8A–C**; **Table 1**). Furthermore, hyperinsulinemia induced by continuous AngII administration resulted in increased insulin-induced AKT phosphorylation, AngII-induced ERK phosphorylation, and OXSR1 phosphorylation in the renal cortex, whereas AMPKα1/2 phosphorylation was suppressed, consistent with our *ex vivo* findings (**Figure 9C**; **Supplementary Figures 8D–G**). In contrast, treatment with canagliflozin improved insulin resistance and considerably suppressed OXSR1 phosphorylation. Although canagliflozin did not affect insulin-induced AKT phosphorylation and AngII-induced ERK phosphorylation, it prevented AMPKα1/2 dephosphorylation.

## 4 Discussion

In this study, we investigated the intricate mechanisms by which SGLT2is regulate renal PT Na^+^ reabsorption, with a specific focus on the WNK1/OXSR1 pathway. Our key findings, supported by gene knockdown experiments using pharmacological antagonists and siRNAs, demonstrate that the WNK1/OXSR1 pathway in the PT acts as a critical convergence point for multiple regulatory mechanisms of Na^+^ reabsorption. Notably, canagliflozin, an SGLT2i, regulated OXSR1 phosphorylation via the AMPKα2 subunit in the PT, leading to suppression of stimulated Na^+^ reabsorption both *ex vivo* and *in vivo*. To visually clarify this sequential signaling hierarchy, the upstream/downstream relationships established by our independent experiments are integrated into a comprehensive schematic model (**Figure 9D**).

The cooperative function of basolateral and luminal Na^+^ transporters in Na^+^ reabsorption suggests the involvement of a master regulator (23). Although the WNK/SPAK/OXSR1 pathway is a well-established master regulator of NCCs and NKCCs in DTs, its specific role in the PT has remained largely unclear. Our findings definitively demonstrate that the WNK/OXSR1 pathway mediates the stimulatory effects of diverse factors, including insulin, PPARγ agonists, and AngII, on PT Na^+^ reabsorption, suggesting that this pathway plays a central role in integrating and promoting multiple Na^+^ reabsorption mechanisms throughout the renal tubules. Furthermore, SGLT2i treatment effectively suppressed this enhanced Na^+^ reabsorption by inhibiting OXSR1 phosphorylation within PTs.

Several hypotheses have been proposed regarding how SGLT2is regulate the WNK/OXSR1 pathway, including alterations in PKC activity, intracellular osmolarity, and cell volume (24–26). However, we observed no significant change in OXSR1 expression in PTs in response to osmotic stress.

Instead, our data strongly implicate AMPK in this regulatory process. Although an association between the WNK1/SPAK/OXSR1 pathway and AMPK has been suggested, this connection has not been clearly established in PTs. Previous studies reporting WNK1-mediated negative regulation of AMPK (27) and WNK463-induced activation of AMPK (28), which inhibits SPAK but not OXSR1 phosphorylation (14), have highlighted a complex regulatory landscape. Conversely, a recent study demonstrated that AMPK inhibition reduces WNK1 expression (29). Our results are consistent with the concept that AMPK negatively regulates SPAK/OXSR1 phosphorylation in PTs. Our results showed a clear dissociation between *Wnk1 mRNA* expression and OXSR1 phosphorylation levels. While insulin and AngII increased Wnk1 mRNA levels, canagliflozin effectively suppressed OXSR1 phosphorylation without affecting this transcriptional upregulation (**Supplementary Figure 5**). This suggests that AMPK functionally overrides WNK1-mediated signaling through post-translational mechanisms. One plausible mechanism is that AMPK directly phosphorylates WNK1 at inhibitory residues, thereby reducing its catalytic activity toward OXSR1, as has been suggested in other contexts in which AMPK acts as a negative regulator of the WNK pathway (14, 28). Biochemically, activated AMPK has been shown to directly phosphorylate the long isoform (L-WNK1) at specific conserved serine residues, which sterically hinders WNK1 kinase activity and disrupts its downstream signaling cascade to OXSR1 (30).

Alternatively, AMPK may influence the formation of the WNK-OXSR1 signaling complex or promote the activity of protein phosphatases that dephosphorylate OXSR1. This hierarchy ensures that the energy-sensing AMPK pathway can dominate over translational stimulatory signals to regulate transepithelial sodium transport, providing a robust mechanism for maintaining cellular energy balance during SGLT2 inhibition.

AMPK, a crucial intracellular energy sensor, is typically activated in response to low glucose availability, caloric restriction, or increased physical activity. Our study provides clear evidence that SGLT2i treatment activates AMPKα2 and suppresses OXSR1 phosphorylation in PTs. Importantly, when AMPKα2 expression was selectively silenced by siRNA in isolated PTs, the inhibitory effect of SGLT2i on OXSR1 phosphorylation was attenuated. Consequently, the SGLT2i-induced reduction in stimulated Na^+^ reabsorption by insulin, PGZ, and AngII was significantly diminished, highlighting the critical role of AMPKα2 in mediating the effects of SGLT2is on PT Na^+^ reabsorption.

We found that canagliflozin increased *Prkaa2* expression as well as AMPKα1/2 phosphorylation in isolated PTs *ex vivo*, consistent with previous reports of SGLT2i-induced AMPK phosphorylation (31–33). This finding suggests that both AMPK synthesis, reflected by increased mRNA expression, and AMPK activation, reflected by phosphorylation, are important and likely act synergistically to regulate AMPK function. Although the precise significance of increased mRNA expression remains unclear due to limited and inconsistent reports, elevated *Prkaa* mRNA levels may indirectly reflect enhanced AMPK protein synthesis.

Interestingly, recent studies have reported the involvement of AMPK in the kidney-protective effects of SGLT2is (15, 16). In particular, these studies proposed that SGLT2i-mediated reduction in intracellular glucose in PTs activates AMPK. This scenario, however, is rather unlikely, because the previous studies (34, 35) demonstrated that SGLT2i did not reduce intracellular glucose in PTs unless the expression of glucose transporter 2 (GLUT2) at the basolateral membrane was severely suppressed. Our results that the AMPK dephosphorylation by insulin and other factors remained intact even at a very low glucose concentration (25 mg/dL) and were completely abolished only under total glucose starvation (0 mg/dL) also supported this view. Nevertheless, our results using siRNA-mediated knockdown of SGLT2 clearly showed that SGLT2 is indispensable for the stimulatory effects of insulin and other factors.

Taken together with these apparently conflicting results, the most likely explanation would be that SGLT2i might activate AMPK by the suppression of transcellular glucose flux but not by the simple reduction of intracellular glucose in PTs. Our results obtained by Cytochalasin B that blocks basolateral GLUT transporters without decreasing intracellular glucose also supported this view (17, 18). Although the detailed mechanism by which the changes in transcellular glucose flux are sensed and transmitted to the intracellular signaling pathways remained to be determined, a similar example linking the suppression of transcellular glucose flux and the energy metabolism in PTs was indeed reported. Thus, the deletion of GLUT2 specifically from PTs induced upregulation of energy metabolism including glycolysis and gluconeogenesis without changing intracellular glucose in PTs (36). The activation of AMPK by glucose starvation, as confirmed in the present study, has been well established (37). Our present study suggests the suppression of transcellular glucose flux also activates AMPK at least in PTs.

Regarding the WNK1 pathway, WNK1 is ubiquitously expressed, with the L-WNK1 present in PTs (38). Our results suggest that the WNK1/OXSR1 pathway in PTs may contribute to fluid retention in pseudohyperaldosteronism type II, a condition traditionally linked to abnormalities in WNK1 and WNK4 in DTs (24). The role of the WNK pathway in regulating Na^+^ transport via NCCs is well established in DTs and other epithelial cells (10, 39, 40), suggesting a broader involvement in hormonal regulatory mechanisms governing Na^+^ transport in PTs.

The cardiovascular and renal protective effects of SGLT2is are well documented and may involve multiple mechanisms, including NHE3 inhibition and correction of hyperfiltration (41). Although canagliflozin did not significantly affect basal NHE activity in our non–heart failure (HF) rat model, it effectively suppressed the stimulatory effects of insulin and AngII on NHE. This finding is particularly important because insulin resistance (42) and AngII hypersecretion (43) are common features of HF. Thus, the organ-protective effects of SGLT2is in HF may, in part, result from their ability to suppress stimulated NHE3 activity. In the present study, we directly confirmed that dapagliflozin, a highly selective SGLT2 inhibitor, also entirely suppresses insulin-stimulated NBCe1 and NHE activities and concurrently prevents the insulin-induced dephosphorylation of AMPK in isolated PTs (**Supplementary Figure 7**). These pharmacological findings, coupled with the data obtained by SGLT2-siRNA knockdown and our previous observations (44), provide definitive evidence that the AMPK-mediated suppression of stimulated Na^+^ reabsorption represents a fundamental class effect of SGLT2 inhibitors rather than a compound-specific trait.

Our rat model, which exhibited hyperinsulinemia, impaired glucose tolerance, hypertension, and proteinuria, also showed renal pathology, including glomerulosclerosis. Glomerular diameter, which was significantly increased in AngII-treated rats, was normalized by canagliflozin, strongly supporting the ability of SGLT2is to correct abnormal afferent arteriolar dilation and suppress hyperfiltration in DKD (45).

While our findings confirm the significant renoprotective effects of canagliflozin, closer examination of its mechanisms suggests a potential link to improved insulin resistance. Continuous AngII administration induced insulin resistance, a known risk factor for both cardiovascular and renal diseases. Our data demonstrate that canagliflozin improved renal pathology and ameliorated insulin resistance. These findings suggest that the nephroprotective effects of canagliflozin may not be solely attributable to direct SGLT2 inhibition within the kidney. Rather, a portion of its renoprotective benefit may be mediated indirectly through improvements in systemic metabolic dysfunction. By alleviating insulin resistance, canagliflozin may reduce metabolic stress on renal cells and attenuate pro-inflammatory and pro-fibrotic signaling pathways implicated in the progression of kidney injury. Collectively, these observations support a more integrated view of canagliflozin-mediated renoprotection, in which improvements in systemic metabolic homeostasis may complement its direct renal actions. It is important to note, however, that the relative contribution of the local AMPK-OXSR1 pathway versus systemic metabolic improvements remains to be fully elucidated. Given that canagliflozin concurrently improved systemic insulin resistance in our model, the observed reduction in OXSR1 phosphorylation *in vivo* may partially reflect a decrease in chronic stimulatory signals (e.g., hyperinsulinemia) reaching the PT. Nevertheless, our *ex vivo* data showing direct AMPK activation by canagliflozin in isolated PTs strongly suggest that intrarenal signaling serves as a primary mechanism that complements systemic metabolic benefits. The renoprotective efficacy of SGLT2is likely stems from this dual action: alleviating systemic metabolic stress while simultaneously resetting the intrarenal signaling convergence point, OXSR1, to prevent maladaptive sodium reabsorption.

Regarding the downstream target regulation, it is important to acknowledge that the precise molecular mechanisms modulating NHE3 and NBCe1 activities—such as specific phosphorylation, cellular trafficking, or protein-protein interactions—were not directly evaluated in this study. Although the WNK1-OXSR1 pathway acts as a master regulator, the functional significance of specific NHE3 phosphorylation sites remains a subject of ongoing debate (46), and it has been documented that PT NHE3 transport activity can fluctuate independently of changes in its total expression or plasma membrane abundance (47). Future studies are warranted to elucidate the exact post-translational modifications of these transporters under SGLT2 inhibition. Furthermore, transcellular sodium reabsorption fundamentally relies on the primary active driving force generated by the basolateral Na^+^/K^+^-ATPase. While we evaluated both apical NHE3 and coupled NBCe1 activities to minimize the oversight of single-transporter measurements, a precise evaluation of basolateral Na^+^/K^+^-ATPase activity was beyond our current technical capabilities. Investigating how the intrarenal AMPK-OXSR1 signaling axis cross-talks with basolateral active transport machineries remains a critical task for future investigations.

This study has some limitations. Genetically modified animals for WNK1, OXSR1, or AMPKα1/2 were not used because of embryonic lethality in knockout models and known species differences. Nevertheless, we effectively investigated the role of the AMPK/WNK1/OXSR1 pathway as a key signaling convergence point governing Na^+^ transport in PTs using siRNA-mediated gene suppression. For protein expression analyses, we used manually isolated PT bundles that are highly enriched in PTs (48), ensuring the reliability of our findings. Despite the lack of reliable antibodies against WNK1, we confirmed the expression of WNK1 and OXSR1 in freshly isolated PTs at the mRNA level, even with a limited RNA yield (~5 ng). These findings were further confirmed using the highly selective SGLT2 inhibitor dapagliflozin (**Supplementary Figure 7**). Together with our recent reports (44), these data reinforce the conclusion that AMPK-mediated suppression of stimulated Na^+^ reabsorption is a fundamental, reproducible feature of SGLT2 inhibition.

In conclusion, our study identifies OXSR1 in PTs and highlights its role as a key signaling convergence point associated with coordinating transepithelial sodium reabsorption. We demonstrate that canagliflozin inhibits the WNK1/OXSR1 pathway via AMPKα2. Our *in-vivo* findings in a DKD rat model show that canagliflozin mitigates tubular injury and modulates molecular pathways relevant to renal pathophysiology. These insights may facilitate the identification of novel therapeutic targets for renal disease, and, given the established cardio- and renoprotective effects of canagliflozin, targeting OXSR1 may represent a promising strategy for the development of new antihypertensive and organ-protective therapies.

## Data Availability

The original contributions presented in the study are included in the article/**Supplementary Material**. Further inquiries can be directed to the corresponding author.

## References

[1] Heerspink HJL Stefánsson BV Correa-Rotter R Chertow GM Greene T Hou FF . Dapagliflozin in patients with chronic kidney disease. N Engl J Med. (2020) 383:1436–46. doi: 10.1056/NEJMoa2024816 32970396

[2] Mahaffey KW Jardine MJ Bompoint S Cannon CP Neal B Heerspink HJL . Canagliflozin and cardiovascular and renal outcomes in type 2 diabetes mellitus and chronic kidney disease in primary and secondary cardiovascular prevention groups. Circulation. (2019) 140:739–50. doi: 10.1161/CIRCULATIONAHA.119.042007 31291786 PMC6727954

[3] Neal B Perkovic V Matthews DR . Canagliflozin and cardiovascular and renal events in type 2 diabetes. N Engl J Med. (2017) 377:644–57. doi: 10.1056/NEJMoa1611925 28605608

[4] Perkovic V Jardine MJ Neal B Bompoint S Heerspink HJL Charytan DM . Canagliflozin and renal outcomes in type 2 diabetes and nephropathy. N Engl J Med. (2019) 380:2295–306. doi: 10.1056/NEJMoa1811744 30990260

[5] Horita S Nakamura M Suzuki M Satoh N Suzuki A Homma Y . The role of renal proximal tubule transport in the regulation of blood pressure. Kidney Res Clin Pract. (2017) 36:12–21. doi: 10.23876/j.krcp.2017.36.1.12 28428931 PMC5331971

[6] Nakamura M Tsukada H Seki G Satoh N Mizuno T Fujii W . Insulin promotes sodium transport but suppresses gluconeogenesis via distinct cellular pathways in human and rat renal proximal tubules. Kidney Int. (2020) 97:316–26. doi: 10.1016/j.kint.2019.08.021 31735358

[7] Endo Y Suzuki M Yamada H Horita S Kunimi M Yamazaki O . Thiazolidinediones enhance sodium-coupled bicarbonate absorption from renal proximal tubules via PPARγ-dependent nongenomic signaling. Cell Metab. (2011) 13:550–61. doi: 10.1016/j.cmet.2011.02.015 21531337

[8] Mizuno T Satoh N Horita S Tsukada H Takagi M Sato Y . Oxidized alkyl phospholipids stimulate sodium transport in proximal tubules via a nongenomic PPARγ-dependent pathway. J Biol Chem. (2022) 298:101681. doi: 10.1016/j.jbc.2022.101681 35124009 PMC8892145

[9] Shirai A Yamazaki O Horita S Nakamura M Satoh N Yamada H . Angiotensin II dose-dependently stimulates human renal proximal tubule transport by the nitric oxide/guanosine 3′,5′-cyclic monophosphate pathway. J Am Soc Nephrol. (2014) 25:1523–32. doi: 10.1681/ASN.2013060596 24511122 PMC4073427

[10] Shekarabi M Zhang J Khanna AR Ellison DH Delpire E Kahle KT . WNK kinase signaling in ion homeostasis and human disease. Cell Metab. (2017) 25:285–99. doi: 10.1016/j.cmet.2017.01.007 28178566

[11] Fezai M Elvira B Warsi J Ben-Attia M Hosseinzadeh Z Lang F . Up-regulation of intestinal phosphate transporter NaPi-IIb (SLC34A2) by the kinases SPAK and OSR1. Kidney Blood Press Res. (2015) 40:555–64. doi: 10.1159/000368531 26506223

[12] Davies M Fraser SA Galic S Choy SW Katerelos M Gleich K . Novel mechanisms of Na+ retention in obesity: phosphorylation of NKCC2 and regulation of SPAK/OSR1 by AMPK. Am J Physiol Renal Physiol. (2014) 307:F96–F106. doi: 10.1152/ajprenal.00524.2013 24808538

[13] Pasham V Pathare G Fajol A Rexhepaj R Michael D Pakladok T . OSR1-sensitive small intestinal Na^+^ transport. Am J Physiol Gastrointest Liver Physiol. (2012) 303:G1212–9. doi: 10.1152/ajpgi.00367.2011 23019198

[14] Pasham V Rotte A Yang W Zelenak C Bhandaru M Föller M . OSR1-sensitive regulation of Na^+^/H^+^ exchanger activity in dendritic cells. Am J Physiol Cell Physiol. (2012) 303:C416–26. doi: 10.1152/ajpcell.00420.2011 22648948

[15] Schaub JA AlAkwaa FM McCown PJ Naik AS Nair V Eddy S . SGLT2 inhibitors mitigate kidney tubular metabolic and mTORC1 perturbations in youth-onset type 2 diabetes. J Clin Invest. (2023) 133:e164486. doi: 10.1172/JCI164486 36637914 PMC9974101

[16] Tuttle KR . Digging deep into cells to find mechanisms of kidney protection by SGLT2 inhibitors. J Clin Invest. (2023) 133:e167700. doi: 10.1172/JCI167700 36856116 PMC9974093

[17] Cheung PT Hammerman MR . Cytochalasin B binding to rabbit proximal tubular basolateral membranes. Kidney Int. (1989) 35:1290–4. doi: 10.1038/ki.1989.124 2770109

[18] Blodgett AB Kothinti RK Kamyshko I Petering DH Kumar S Tabatabai NM . A fluorescence method for measurement of glucose transport in kidney cells. Diabetes Technol Ther. (2011) 13:743–51. doi: 10.1089/dia.2011.0041 21510766 PMC3118926

[19] Hummel CS Lu C Liu J Ghezzi C Hirayama BA Loo DDF . Structural selectivity of human SGLT inhibitors. Am J Physiol Cell Physiol. (2012) 302:C373–82. doi: 10.1152/ajpcell.00328.2011 21940664 PMC3328840

[20] Kikuchi E Mori T Zeniya M Isobe K Ishigami-Yuasa M Fujii S . Discovery of novel SPAK inhibitors that block WNK kinase signaling to cation chloride transporters. J Am Soc Nephrol. (2015) 26:1525–36. doi: 10.1681/ASN.2014060560 25377078 PMC4483590

[21] Nakamura M Yamazaki O Shirai A Horita S Satoh N Suzuki M . Preserved Na/HCO3 cotransporter sensitivity to insulin may promote hypertension in metabolic syndrome. Kidney Int. (2015) 87:535–42. doi: 10.1038/ki.2014.351 25354240

[22] Nakamura M Satoh N Suzuki M Kume H Homma Y Seki G . Stimulatory effect of insulin on renal proximal tubule sodium transport is preserved in type 2 diabetes with nephropathy. Biochem Biophys Res Commun. (2015) 461:154–8. doi: 10.1016/j.bbrc.2015.04.005 25866180

[23] Aperia A Fryckstedt J Holtbäck U Belusa R Cheng XJ Eklöf AC . Cellular mechanisms for bi-directional regulation of tubular sodium reabsorption. Kidney Int. (1996) 49:1743–7. doi: 10.1038/ki.1996.259 8743489

[24] Naguro I Umeda T Kobayashi Y Maruyama J Hattori K Shimizu Y . ASK3 responds to osmotic stress and regulates blood pressure by suppressing WNK1-SPAK/OSR1 signaling in the kidney. Nat Commun. (2012) 3:1285. doi: 10.1038/ncomms2283 23250415

[25] Ishizawa K Wang Q Li J Xu N Nemoto Y Morimoto C . Inhibition of sodium glucose cotransporter 2 attenuates the dysregulation of Kelch-like 3 and NaCl cotransporter in obese diabetic mice. J Am Soc Nephrol. (2019) 30:782–94. doi: 10.1681/ASN.2018070703 30914436 PMC6493993

[26] Watanabe K Umeda T Niwa K Naguro I Ichijo H . A PP6-ASK3 module coordinates the bidirectional cell volume regulation under osmotic stress. Cell Rep. (2018) 22:2809–17. doi: 10.1016/j.celrep.2018.02.045 29539411

[27] Gallolu Kankanamalage S Lee AY Wichaidit C Lorente-Rodriguez A Shah AM Stippec S . Multistep regulation of autophagy by WNK1. Proc Natl Acad Sci USA. (2016) 113:14342–7. doi: 10.1073/pnas.1617649113 27911840 PMC5167150

[28] Prisco SZ Eklund M Raveendran R Thenappan T Prins KW . With no lysine kinase 1 promotes metabolic derangements and RV dysfunction in pulmonary arterial hypertension. JACC Basic Transl Sci. (2021) 6:834–50. doi: 10.1016/j.jacbts.2021.09.004 34869947 PMC8617575

[29] Wang F Yan X Shi G Zhang L Jing X . Loss of WNK1 suppressed the Malignant behaviors of hepatocellular carcinoma cells by promoting autophagy and activating AMPK pathway. Dis Markers. (2022) 2022:6831224. doi: 10.1155/2022/6831224 36618969 PMC9822739

[30] Lin SC Ma C Chang KJ Cheong HP Lee MC Lan YT . The post-translational modification networking in WNK-centric hypertension regulation and electrolyte homeostasis. Biomedicines. (2022) 10:2169. doi: 10.3390/biomedicines10092169 36140271 PMC9496095

[31] Liu X Xu C Xu L Li X Sun H Xue M . Empagliflozin improves diabetic renal tubular injury by alleviating mitochondrial fission via AMPK/SP1/PGAM5 pathway. Metabolism. (2020) 111:154334. doi: 10.1016/j.metabol.2020.154334 32777444

[32] Mancini SJ Boyd D Katwan OJ Strembitska A Almabrouk TA Kennedy S . Canagliflozin inhibits interleukin-1β-stimulated cytokine and chemokine secretion in vascular endothelial cells by AMP-activated protein kinase-dependent and -independent mechanisms. Sci Rep. (2018) 8:5276. doi: 10.1038/s41598-018-23420-4 29588466 PMC5869674

[33] Yang X Liu Q Li Y Tang Q Wu T Chen L . The diabetes medication canagliflozin promotes mitochondrial remodelling of adipocyte via the AMPK-Sirt1-Pgc-1α signaling pathway. Adipocyte. (2020) 9:484–94. doi: 10.1080/21623945.2020.1807850 32835596 PMC7469612

[34] Zhang A Nakano D Kittikulsuth W Yamashita Y Nishiyama A . Luseogliflozin, a SGLT2 inhibitor, does not affect glucose uptake kinetics in renal proximal tubules of live mice. Int J Mol Sci. (2021) 22:8169. doi: 10.3390/ijms22158169 34360935 PMC8347119

[35] Zhang Y Nakano D Guan Y Hitomi H Uemura A Masaki T . A sodium-glucose cotransporter 2 inhibitor attenuates renal capillary injury and fibrosis by a vascular endothelial growth factor-dependent pathway after renal injury in mice. Kidney Int. (2018) 94:524–35. doi: 10.1016/j.kint.2018.05.002 30045814

[36] Ahmad M Permyakova A Baraghithy S Sahu N Abramovich I Agranovich B . Metabolic consequences of altered kidney glucose reabsorption under normoglycemic conditions. Mol Metab. (2025) 98:102192. doi: 10.1016/j.molmet.2025.102192 40550327 PMC12270078

[37] Leprivier G Rotblat B . How does mTOR sense glucose starvation? AMPK is the usual suspect. Cell Death Discov. (2020) 6:27. doi: 10.1038/s41420-020-0260-9 32351714 PMC7176732

[38] Cheng CJ Yoon J Baum M Huang CL . STE20/SPS1-related proline/alanine-rich kinase (SPAK) is critical for sodium reabsorption in isolated, perfused thick ascending limb. Am J Physiol Renal Physiol. (2015) 308:F437–43. doi: 10.1152/ajprenal.00493.2013 25477470 PMC4346744

[39] Goldsmith EJ Rodan AR . Intracellular ion control of WNK signaling. Annu Rev Physiol. (2023) 85:383–406. doi: 10.1146/annurev-physiol-031522-092550 36228173

[40] Uchida S Mori T Susa K Sohara E . NCC regulation by WNK signal cascade. Front Physiol. (2022) 13:1081261. doi: 10.3389/fphys.2022.1081261 36685207 PMC9845728

[41] Verma S McMurray JJ . SGLT2 inhibitors and mechanisms of cardiovascular benefit: a state-of-the-art review. Diabetologia. (2018) 61:2108–17. doi: 10.1007/s00125-018-4670-7 30132036

[42] Inoue BH dos Santos L Pessoa TD Antonio EL Pacheco BP Savignano FA . Increased NHE3 abundance and transport activity in renal proximal tubule of rats with heart failure. Am J Physiol Regul Integr Comp Physiol. (2012) 302:R166–74. doi: 10.1152/ajpregu.00127.2011 22031782

[43] Borges-Júnior FA Silva Dos Santos D Benetti A Polidoro JZ Wisnivesky ACT Crajoinas RO . Empagliflozin inhibits proximal tubule NHE3 activity, preserves GFR, and restores euvolemia in nondiabetic rats with induced heart failure. J Am Soc Nephrol. (2021) 32:1616–29. doi: 10.1681/ASN.2020071029 33846238 PMC8425656

[44] Nakamura M Satoh N Mizuno T Takagi M Horita S Nangaku M . Combined effect of esaxerenone and dapagliflozin on aldosterone-mediated sodium reabsorption and potassium excretion. Front Physiol. (2025) 16:1677518. doi: 10.3389/fphys.2025.1677518 41458378 PMC12738813

[45] Kidokoro K Cherney DZI Bozovic A Nagasu H Satoh M Kanda E . Evaluation of glomerular hemodynamic function by empagliflozin in diabetic mice using *in vivo* imaging. Circulation. (2019) 140:303–15. doi: 10.1161/CIRCULATIONAHA.118.037418 30773020

[46] Kocinsky HS Zhang J Edwards A Girardi MN Biemesderfer D Caplan MJ . NHE3 phosphorylation at serines 552 and 605 does not directly affect NHE3 activity. Am J Physiol Renal Physiol. (2007) 293:F130–9. doi: 10.1152/ajprenal.00042.2007 17409282

[47] Wang T Liu T Xu S Frindt G Weinstein AM Palmer LG . High dietary K^+^ intake inhibits proximal tubule transport. Am J Physiol Renal Physiol. (2023) 324(5):F713–23. doi: 10.1152/ajprenal.00013.2023 37318989 PMC10396284

[48] Clark JZ Chen L Chou CL Jung HJ Lee JW Knepper MA . Representation and relative abundance of cell-type selective markers in whole-kidney RNA-seq data. Kidney Int. (2019) 95:787–96. doi: 10.1016/j.kint.2018.11.028 30826016 PMC7466803

