# Supplementary material for: AMPK-OXSR1 signaling coordinates proximal tubular sodium transport and is modulated by canagliflozin
Source: Front Endocrinol (Lausanne). 2026 Jul 31;17:1828652. doi: 10.3389/fendo.2026.1828652 (PMC13472824; doi:10.3389/fendo.2026.1828652)
Supplement: Supplementary file 1 [file DataSheet1.pdf]

Supplementary Material

1 Supplementary Methods

1.1 Animal samples

All animal experiments were conducted in accordance with the Guidelines for Proper Conduct of Animal Experiments by the Science Council of Japan and the ARRIVE reporting guidelines, with approval from the Institutional Animal Care and Use Committee of the University of Tokyo (authorization number: P17-070, H-18-260). Experiments were performed using adult male rats exclusively to avoid any potential confounding effects from sexual dimorphism, which protects female mice from obesity and glucose intolerance. Male Wistar rats used in control experiments were purchased from CLEA Japan, Inc. (Tokyo, Japan), and male Long-Evans Tokushima Otsuka (LETO) and Otsuka Long-Evans Tokushima Fatty (OLETF) rats were purchased from SLC Japan, Inc. (Tokyo, Japan). All rats were housed in cages under a 12/12-h light/dark cycle and provided with water and a regular diet containing 0.5% NaCl (MF; Oriental Yeast Co., Ltd., Tokyo, Japan) until unilateral nephrectomy was performed. After unilateral nephrectomy, rats were fed a high-salt chow diet of 8% NaCl-containing feed (F2Dahl-8.0; Oriental Yeast Co., Ltd.) and *ad libitum* access to water.

All rats were anesthetized with a mixture of three anesthetics—medetomidine (intraperitoneally, 0.75 mg/kg; Sandoz, Tokyo, Japan), midazolam (intraperitoneally, 4 mg/kg; Nippon Zenyaku Kogyo, Fukushima, Japan), and butorphanol (intraperitoneally, 5 mg/kg; Meiji Seika Pharma, Tokyo, Japan)—before being euthanized.

Finally, 45 and 57 rats were selected for *in vivo* and *ex vivo* experiments, respectively.

1.2 Human samples

Intact human renal cortical tissue was obtained from surgical specimens based on a study protocol approved by the Research Ethics Committee of the Faculty of Medicine of the University of Tokyo (approval number: 2520–[13]). Signed informed consent was obtained from all participants. The study was conducted following the principles of the Declaration of Helsinki. Eligible participants were adults with an estimated glomerular filtration rate (eGFR) ≥45 mL/min/1.73 m^2, without diabetes and hypertension, who had undergone unilateral nephrectomy for renal cancer at the Department of Urology, University of Tokyo Hospital. In this study, participants with eGFR<45 mL/min/1.73 m^2, impaired glucose tolerance, or hypertension in any one of the screening tests before nephrectomy were excluded.

1.3 Proximal tubule (PT) isolation

PTs were isolated from human and rat kidney cortices, as previously described.[S1-4] Briefly, nephrons were collected from 1.5-mm-thick renal cortical tissue samples using micro tweezers in an ice-cold HEPES-buffered solution. The S2 segments of the PTs were collected based on their morphological characteristics.

1.4 Measurements of Na^+-HCO_3^- cotransporter (NBCe1) activity in renal PTs from rats and humans

NBCe1 activity was determined as previously described. Briefly, the PT (S2 segment) fragment was manually microdissected from rat or human kidneys without collagenase treatment and transferred to a perfusion chamber mounted on an inverted microscope. To avoid the influence of luminal transporters, the PT fragment was collapsed using two holding pipettes. The luminally collapsed PT was incubated with the acetoxymethyl ester form of the pH-sensitive fluorescent dye 2′,7′-bis(carboxyethyl)-5(6)-carboxyfluorescein acetoxymethyl ester (BCECF/AM; Dojindo Laboratories, Kumamoto, Japan) in Dulbecco's modified Eagle's medium (DMEM) for 10 min, and intracellular pH (pHi) was monitored using a photometry system, MetaFluor 7.7 software (Molecular Devices, Sunnyvale, CA, USA). The chamber was perfused with prewarmed (38°C) DMEM equilibrated with 5% CO_2/95% O_2 gas. Subsequently, bath HCO_3^- concentrations were repeatedly alternated between 25 and 12.5 mM in the absence and presence of insulin (Fujifilm Wako Pure Chemical, Osaka, Japan), pioglitazone (Sigma-Aldrich, St Louis, MO, USA), angiotensin II (AngII, Fujifilm Wako Pure Chemical), the pan-WNK inhibitor WNK463 (ChemScene LLC, Monmouth Junction, NJ, USA) at 1 μM, the SPAK/OXSR1 inhibitor closantel (Sigma-Aldrich) at 10 μM, the SGLT2 inhibitor canagliflozin (Tanabe Pharma Corporation, Osaka, Japan) at 300 nM and dapagliflozin (Sigma-Aldrich) at 30 μM, the AKT-specific inhibitor AKT inhibitor VIII (Sigma-Aldrich) at 1 μM, the ERK1/2-specific inhibitor PD98059 at 10 μM, the AMPK-specific inhibitor dorsomorphin at 50 μM, and the AMPK-specific stimulator A-769662 at 50 μM. Canagliflozin, the AKT inhibitor, the ERK1/2 inhibitor, the AMPK inhibitor, and the AMPK stimulator were first dissolved in DMSO at the prescribed concentrations, diluted in DMEM in two steps, and added to DMEM to a final DMSO concentration of ≤0.1%. Isolated PTs were cultured overnight and used for the experiments.

All selected agents exhibited sufficient inhibitory activities without affecting basal NBCe1 activity in PTs.[S1-4] NBCe1 activity was calculated using the rate of pHi decrease in response to bath HCO_3^- reduction and the buffer capacity. WNK463 was used at a concentration that reliably inhibited WNK1. The following PT data were excluded from the analysis: pHi reduction below 0.1, basal pH below 6.5.

1.5 Measurement of luminal Na^+-H^+ exchanger (NHE) activity in renal PTs from rats and humans

Luminal NHE activity was determined as previously described.[S3-4] Briefly, the PT (S2 segment) fragment was freshly isolated, similar to that for NBCe1 activity measurements, and attached to a glass coverslip with Cell-Tak glue (Corning, One Riverfront Plaza, NY, USA). The PTs were placed in a perfusion chamber mounted on an inverted microscope, and the end of the tubule was cut with capillary glass to expose its lumen sufficiently. The lumen-opened PT was incubated with BCECF/AM in HEPES-buffered solution (144 mM Na, 5 mM K, 1.5 mM Ca (2), 1 mM Mg (2), 137 mM Cl, 2 mM H_2PO (4), 1 mM SO_4 (2), 5.5 mM glucose, and 25 mM HEPES; adjusted to pH 7.4) for 10 min, and pHi was monitored using MetaFluor 7.7 software.[S3, S5, S6] A prewarmed (38°C) HEPES-buffered solution was used for the bath perfusate, and 200 nM bafilomycin A1 (Fujifilm Wako Pure Chemical) and 10 µM caliporide (Santa Cruz Biotechnology, Santa Cruz, CA, USA) were added to block the effect of vacuolar ATPase and NHE1 on PT transport.[S3, S5, S6] The perfusate was repeatedly switched from a HEPES-buffered solution to an isotonic Na-free solution (144 mM N-methyl-D-glucamine, 5 mM K, 1.5 mM Ca, 1 mM Mg, 137 mM Cl, 2 mM H_2PO_4, 1 mM SO_4, 5.5 mM glucose, and 25 mM HEPES; adjusted to pH 7.4) in the absence and presence of insulin, pioglitazone, AngII, or other agents, including WNK463 (1 µM), closantel (10 µM), canagliflozin (300 nM), the specific AKT inhibitor VIII (Sigma-Aldrich) at 1 µM, the specific ERK1/2 inhibitor PD98059 (7 µM), the specific AMPK inhibitor dorsomorphin (50 µM), and the specific AMPK stimulator A-769662 (50 µM). All selected materials exhibited sufficient inhibitory activities without affecting basal NHE activity in PTs. WNK463 was used at a concentration that reliably inhibited WNK1. Luminal NHE activity was calculated using the rate of pHi decrease caused by bath Na^+ removal and the buffer capacity. The following PT data were excluded from the analysis: pHi reduction below 0.1, basal pH below 6.5.

1.6 Small interfering (siRNA) treatment in isolated rat PTs

The siRNA treatment of isolated rat PTs was performed as previously described.[S1, S3, S5, S6] Briefly, freshly isolated rat PTs were treated with one or two different siRNAs, including siRNAs targeting *OXSR1* (s164577 and RSS321852; Invitrogen; Thermo Fisher Scientific, Waltham, MA, USA) at 40 nM, *STK39* (s132640; Invitrogen, and RSS329088; Invitrogen) at 40 nM, *WNK1* (195755; Invitrogen, and 116477; Dharmacon; Horizon Discovery Ltd., Cambridge, UK) at 40 nM, *slc5a2* at 40nM(AM16708; Invitrogen), *Prkaa2* (RSS330429; Invitrogen; Thermo Fisher Scientific), or a scrambled negative

control (sc-37007; Santa Cruz Biotechnology or siGENOME Non-Targeting siRNA Pool #1 D-001206-13-05; Dharmacon; Horizon Discovery Ltd). Transfection was performed using Lipofectamine 2000 and Opti-MEM I Reduced Serum Medium (both from Invitrogen; Thermo Fisher Scientific). The PTs were incubated overnight in DMEM supplemented with 10% fetal bovine serum (Gibco; Thermo Fisher Scientific) at 37°C for subsequent measurement of NBCe1 and luminal NHE activities and for quantitative polymerase chain reaction (PCR).

1.7 Osmotic stress treatment

Following a previous report,[S7] isolated PTs were exposed to osmotic stress. Specifically, three buffer solutions with different osmolarities (hypotonic, isotonic, and hypertonic) were prepared. The isotonic buffer used HEPES buffer, hypertonic buffer contained 200 mM D-mannitol (Fujifilm Wako Pure Chemical) in isotonic buffer, and hypotonic buffer was an isotonic Na-free solution. For pre-incubation, PT samples were incubated in DMEM at 37°C for 40 min under 5% CO_2, followed by 10-min exposure to the respective solutions.

1.8 Effect of glucose concentration on AMPK phosphorylation in PTs

Isolated PT bundles were cultured for 24 h in glucose-free DMEM. For the glucose concentration study, the medium was supplemented with various concentrations of D-glucose (100, 75, 50, 25, and 0 mg/dL), with the 0 mg/dL D-glucose medium containing canagliflozin. In separate experiments, the effects of 0.1 µM insulin, 0.3 µM pioglitazone, or 0.1 nM AngII were assessed. D-mannitol (Fujifilm Wako Pure Chemical) was used to maintain the osmolality of all media at 280–290 mOsm/L. Osmolality was measured as described in the Osmotic stress treatment section and based on the methods described in the literature. Following the culture period, the phosphorylation of AMPK was assessed.

1.9 Evaluation of transcellular glucose flux using Cytochalasin B

To evaluate the effects of transcellular glucose flux on the AMPK-OXSR1 signaling pathway, PTs were incubated for 24 h in glucose-free or insulin-free DMEM. To selectively disrupt transcellular glucose flux without decreasing intracellular glucose availability, PTs were pretreated with 10 μM Cytochalasin B for 30 min prior to the experimental treatments, as previously described with minor modifications. [S8](Chopra et al., Diabetologia, 2012)

1.10 RNA extraction and quantitative PCR analysis

Total RNA was extracted from tissues and cells using Isogen II (Nippon Gene, Tokyo, Japan) based on the instructions of the manufacturer. First-strand cDNA was synthesized using a cDNA Synthesis Kit (Takara, Tokyo, Japan), as previously reported.[S1, S3, S5, S6] mRNA levels were estimated using quantitative PCR (Prism 7000; Applied Biosystems, Foster City, CA, USA) with TaqMan Gene Expression Master Mix (Applied Biosystems) and TaqMan Gene Expression Assay kits: Rn00671521_m1 for rat *WNK1*, Rn00598070_m1 for *WNK4*, Rn01423506_m1 for *OXSR1*, Rn00667869_m1 for rat β-actin, Rn00584747_m1 for rat Slca4a, Rn00561944_m1 for rat Slc9a3, Rn00574917_m1 for rat Slc5a2, Rn00665045_m1 for rat Prkaa1/AMPKα1, and Rn00576935_m1 for rat *Prkaa2*/AMPKα2, Rn07313311_m1 for rat *Nphs2*, Rn01531762_m1 for rat *Slc12a3*, Rn00563755_m1for rat *Aqp2* (Thermo Fisher Scientific). mRNA levels were normalized to β-actin expression.

1.11 Western blot analysis

Thin kidney cortex slices were obtained from rats and humans and divided into small bundles, as previously described, while excluding glomeruli, Henle's loop, distal tubules (DTs), and collecting ducts as much as possible.[S1-S6] Small bundles were homogenized in ice-cold buffer A [25 mM Tris-HCl (pH 7.4), 10 mM sodium orthovanadate, 10 mM sodium pyrophosphate, 100 mM sodium fluoride, 10 mM EDTA, 10 mM EGTA, and 1 mM phenylmethylsulfonyl fluoride] and centrifuged (4°C, 12,000 × *g*, 10 min). Supernatants were collected for analysis.

Samples were incubated in DMEM at 37°C under 5% CO_2 for 40 min in the presence or absence of inhibitors, such as 10 μM closantel, 1 μM WNK463, or 300 nM canagliflozin, and subsequently incubated for 15 min in DMEM containing 0.1 μM insulin, 0.3 μM pioglitazone, or 0.1 nM AngII. After incubation, the samples were homogenized in ice-cold buffer A [25 mM Tris-HCl (pH 7.4), 10 mM sodium orthovanadate, 10 mM sodium pyrophosphate, 100 mM sodium fluoride, 10 mM EDTA, 10 mM EGTA, and 1 mM phenylmethylsulfonyl fluoride][S1-S6] and centrifuged at 12,000 × *g* for 10 min. Based on our previous reports, the supernatants from each sample were collected and divided into aliquots containing equal amounts (approximately 20 μg) of protein.[S1-S6] The samples were then separated using 10% sodium dodecyl sulfate-polyacrylamide gel electrophoresis and transferred onto nitrocellulose membranes. Transfer was performed at 25 V with a current of 5.5 mA/cm^2. After blocking the membranes with 5% skim milk in Tris-buffered saline (137 mM NaCl, 2.68 mM KCl, and 25 mM Tris; adjusted to pH 7.4) for 1 h, they were incubated with primary antibodies at 4°C overnight. Following

this step, the samples were incubated with horseradish peroxidase (HRP)-conjugated secondary antibodies at 23–25℃ for 1 h. Primary antibodies against phospho-SPAK (Ser373)/phospho-OXSR1 (Ser325) (07-2273; 1:1,000 dilution) were purchased from Sigma-Aldrich, Inc. AKT (#9272; 1:1,000 dilution), phospho-AKT (Ser473) (#9271; 1:1,000 dilution), ERK1/2 (#9102;1:1,000 dilution), phospho-ERK1/2 (Thr202/Tyr204) (#9101;1:1,000 dilution), AMPK (#2532;1:500 dilution), phosphor-AMPK (Thr172) (#2535;1:500 dilution), and beta-actin (#4970; 1:1,000 dilution) were purchased from Cell Signaling Technology (Danvers, MA, USA). SGLT2 (sc-393350; 1:100 dilution), NHE3 (SC-136368; 1:100 dilution), and OXSR1 (SC-27175; 1:100 dilution) were purchased from Santa Cruz Biotechnology. NBCe1 (GTX54676; 1:500 dilution) was purchased from GeneTex Inc. (Irvine, CA, USA).

The HRP-conjugated anti-rabbit (111-035-003) and anti-mouse (115-035-003) immunoglobulin G (IgG) antibodies were purchased from Jackson ImmunoResearch Laboratories (West Grove, PA, USA). Protein bands were detected using a chemiluminescence detection system (ImageQuant LAS 4000 Mini; GE Healthcare, Little Chalfont, UK).

1.12 Rat experiment *in vivo*

Male LETO and OLETF rats were housed under a 12-h light/12-h dark cycle and allowed *ad libitum* access to sterile water and standard food. Rats underwent unilateral nephrectomy at 8 weeks of age and were categorized into five groups: 1) LETO rats with unilateral nephrectomy serving as controls; 2) OLETF rats with unilateral nephrectomy; 3) OLETF rats with unilateral nephrectomy and continuous AngII administration; 4) OLETF rats with unilateral nephrectomy plus daily administration of canagliflozin; 5) OLETF rats with unilateral nephrectomy, continuous AngII administration, and daily administration of canagliflozin.

LETO rats served as control animals, OLETF rats were treated with either a continuous pump of 400 ng/kg/min AngII (dosage based on a previous report[S9]), canagliflozin, or a continuous pump of AngII at 400 ng/kg/min and canagliflozin. Each experimental group consisted of 6–8 rats, and canagliflozin was administered orally once a day. Blood pressure and body weight were measured weekly, and rats were fasted for 12 h before euthanasia. Urinary output was measured by keeping the rats in a metabolic cage and measuring their urination after 4 h. All blood tests were performed using i-STAT® (Abbott Laboratories, Abbott Park, IL, USA), and blood samples were collected after anesthesia and prior to euthanasia. AngII was infused into rats for 30 days using subcutaneously implanted ALZET osmotic pumps 2ML4 (DURECT Corporation,

Cupertino, CA, USA). Canagliflozin was administered by oral gavage at 10 mg/kg once daily in the morning. Finally, 37 rats were included in the *in vivo* experiments.

1.13 Blood pressure measurement

Blood pressure was measured in conscious rats using the tail-cuff method (BP-98A, Softron Co., Ltd., Brussels, Belgium). Measurements were taken at the beginning of the treatment period and every 2 weeks thereafter. The average of three stable measurements was recorded for each animal.

1.14 Biochemical parameter measurements

Fasting blood samples were collected from the tail vein after a 12-h fast. Plasma insulin concentrations were measured using an enzyme-linked immunosorbent assay (ELISA) kit (Shibayagi Co., Ltd., Gunma, Japan) according to the manufacturer's instructions. At the time of euthanasia, blood samples were collected from the vena cava after anesthesia. Plasma levels of blood urea nitrogen (BUN), creatinine, potassium, and glucose were immediately measured using the i-STAT® handheld clinical analyzer with the CHEM8+ cartridge (Abbott Laboratories).

1.15 Randomization in the *in vivo* experiments

For the *in vivo* studies, forty-four OLETF rats were randomly allocated into four groups. In addition, LETO and OLETF rats were assigned random numbers generated using the standard RAND() function in Microsoft Excel (Microsoft Corp., Redmond, WA, USA). Then, each rat was assigned a cage number based on its position on the rack.

1.16 Immunohistochemistry studies

To evaluate transporter expression in model rats, kidneys were fixed in 4% paraformaldehyde, sectioned 5-μm thick at 25°C, air-dried, immersed in phosphate-buffered saline, and incubated overnight at 4°C with the various antibodies described below.

The primary antibodies used included anti-SGLT2 (sc-393350; 1:50 dilution), anti-NHE3 (SC-136368; 1:50 dilution), anti-NBCe1 (GTX54676; 1:50 dilution), and OXSR1 (Ab230627; 1:100 dilution).

Subsequently, samples were incubated for 60 min at 24°C with a mixture of Alexa Fluor 488 anti-rabbit or anti-goat IgG, Alexa Fluor 568 phalloidin for F-actin labeling, and DAPI for nuclear labeling. The fluorescence signals were then observed and evaluated using a confocal laser scanning microscope (Olympus, Tokyo, Japan).

1.17.1 Quantitative pathological analysis

1.17.1.1 Glomerular diameter measurement

In total, 8–10 glomeruli were randomly selected from the renal cortex of each rat, and the largest diameter of the Bowman sac was measured using periodic acid-Schiff (PAS)- or hematoxylin and eosin -stained specimens.

1.17.1.2 Glomerular Sclerosis Index

The glomerular Sclerosis Index was used for evaluation.[S10] Glomeruli were classified as unchanged glomeruli (-), mild mesangial thickening (-), segmental sclerosis (+), and global sclerosis (++). Fifty glomeruli were identified from the renal cortex of each rat, and each was scored 1–4 based on the degree of glomerular sclerosis. The mean score was used as the glomerular Sclerosis Index.

1.17.1.3 Tubular injury score

PAS-stained samples were visualized using a digital microscope camera at 100× magnification, and 10 arbitrary non-overlapping areas were imaged. The percentage of the area occupied by tubular injury was semi-quantified and calculated as a tubular injury score as follows: 0, no tubular injury; 0.5,<5%; 1.0, 5–20%; 1.5, 21–35%; 2.0, 36–50%; 2.5, 51–65%; 3.0, >65%.

1.17.1.4 *Quantification and statistical analysis*

All data are expressed as means ± standard errors of the mean. Data were analyzed using JMP Pro 18 software (SAS Institute, Cary, NC, USA). For multiple group comparisons, a two-way analysis of variance (ANOVA) followed by Tukey's post-hoc test, or the Kruskal–Wallis test followed by the Steel or Steel–Dwass test was employed, depending on the data distribution. For comparisons between two groups, an unpaired, two-tailed Student's t-test or Wilcoxon signed-rank test was used.

Statistical significance was defined as $p<0.05$.

2 Supplementary Figures

A

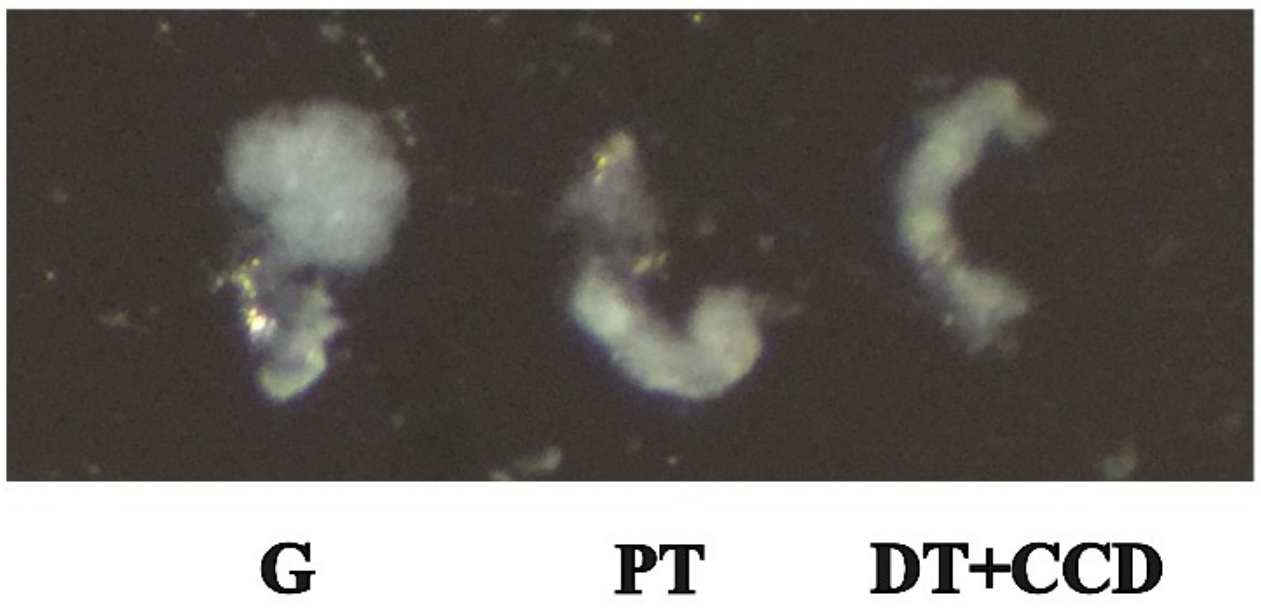

B

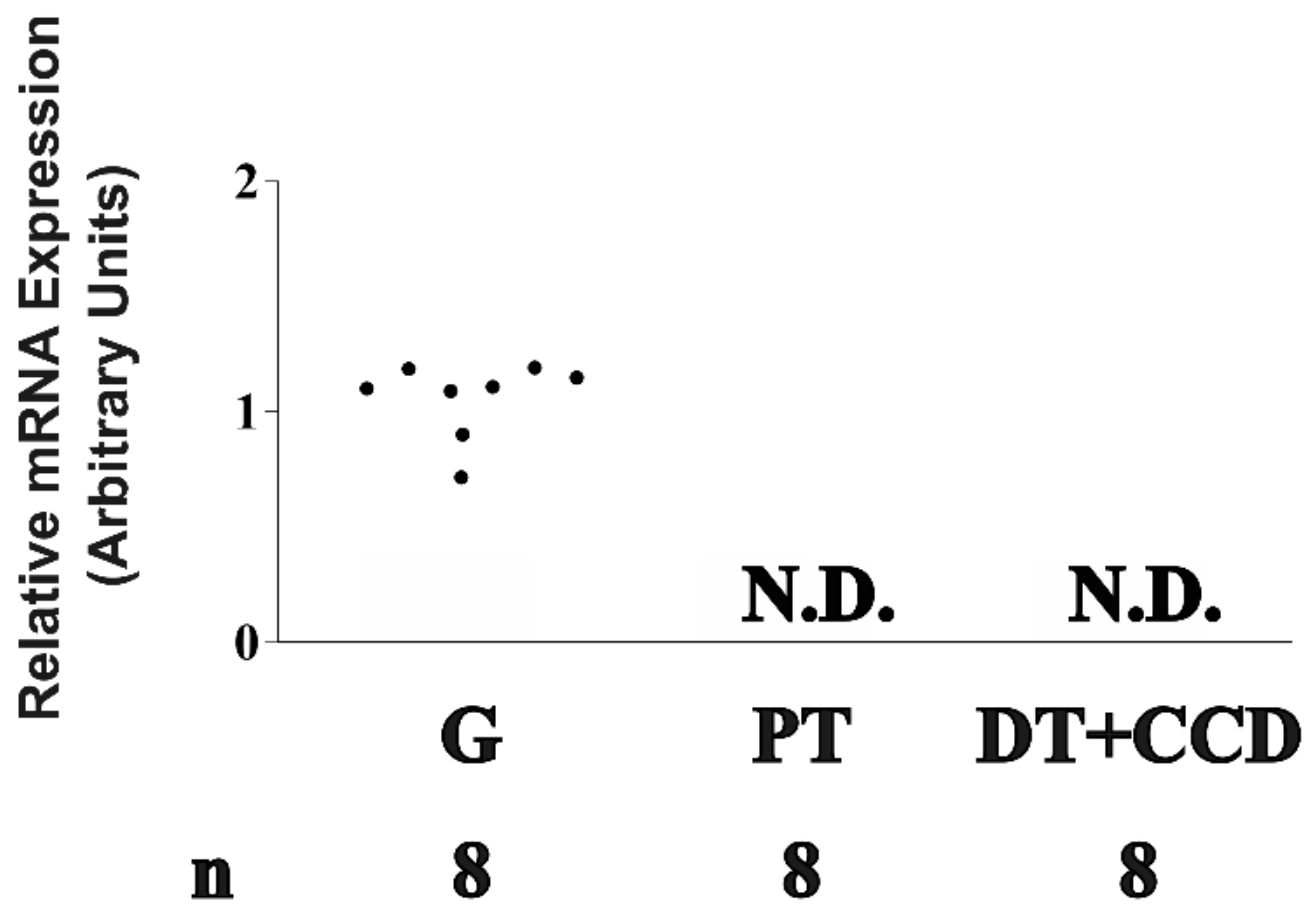

C

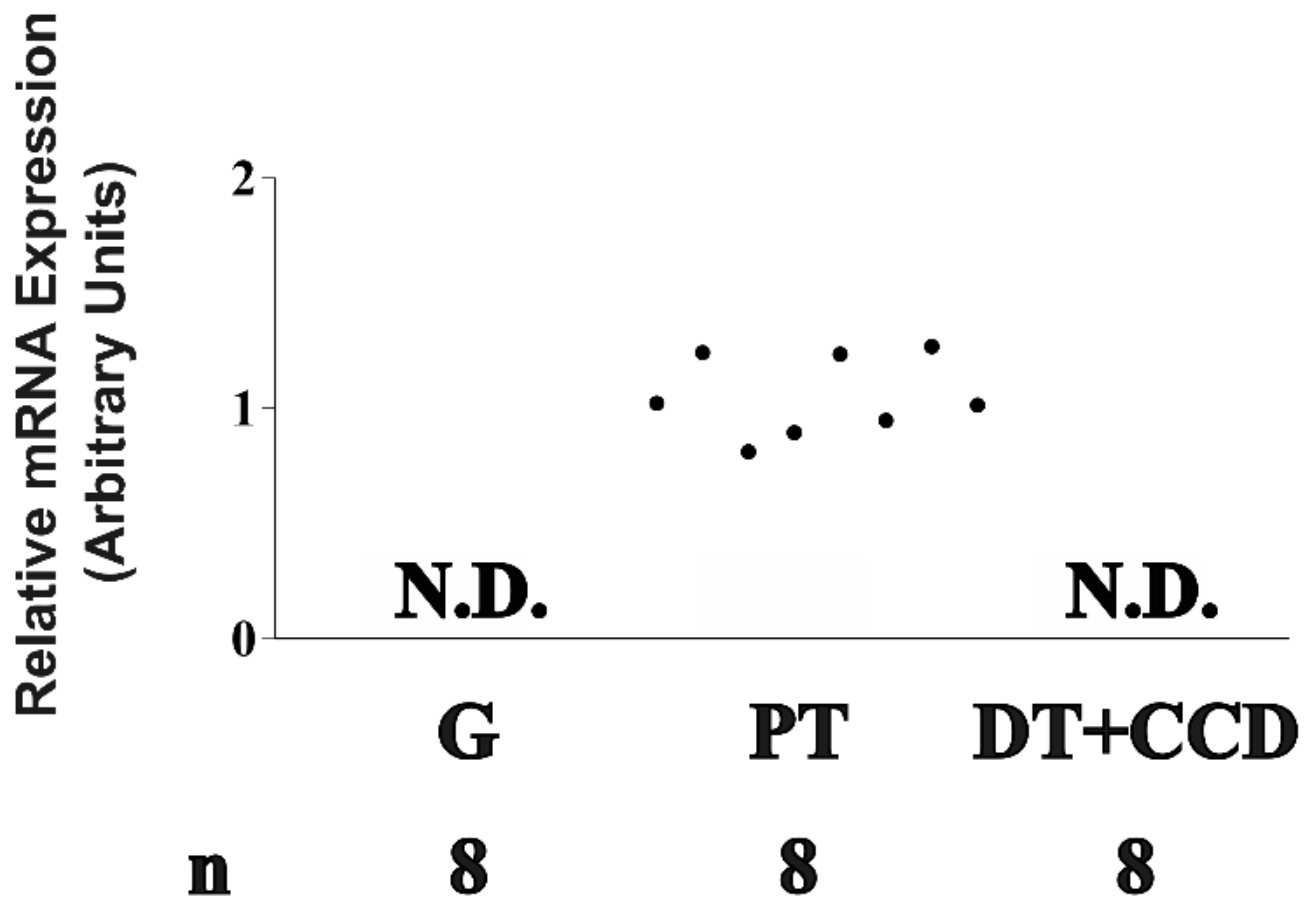
Relative mRNA Expression
(Arbitrary Units)
2
1
0
N.D.
N.D.
G
PT
DT+CCD
n
8
8
8

D

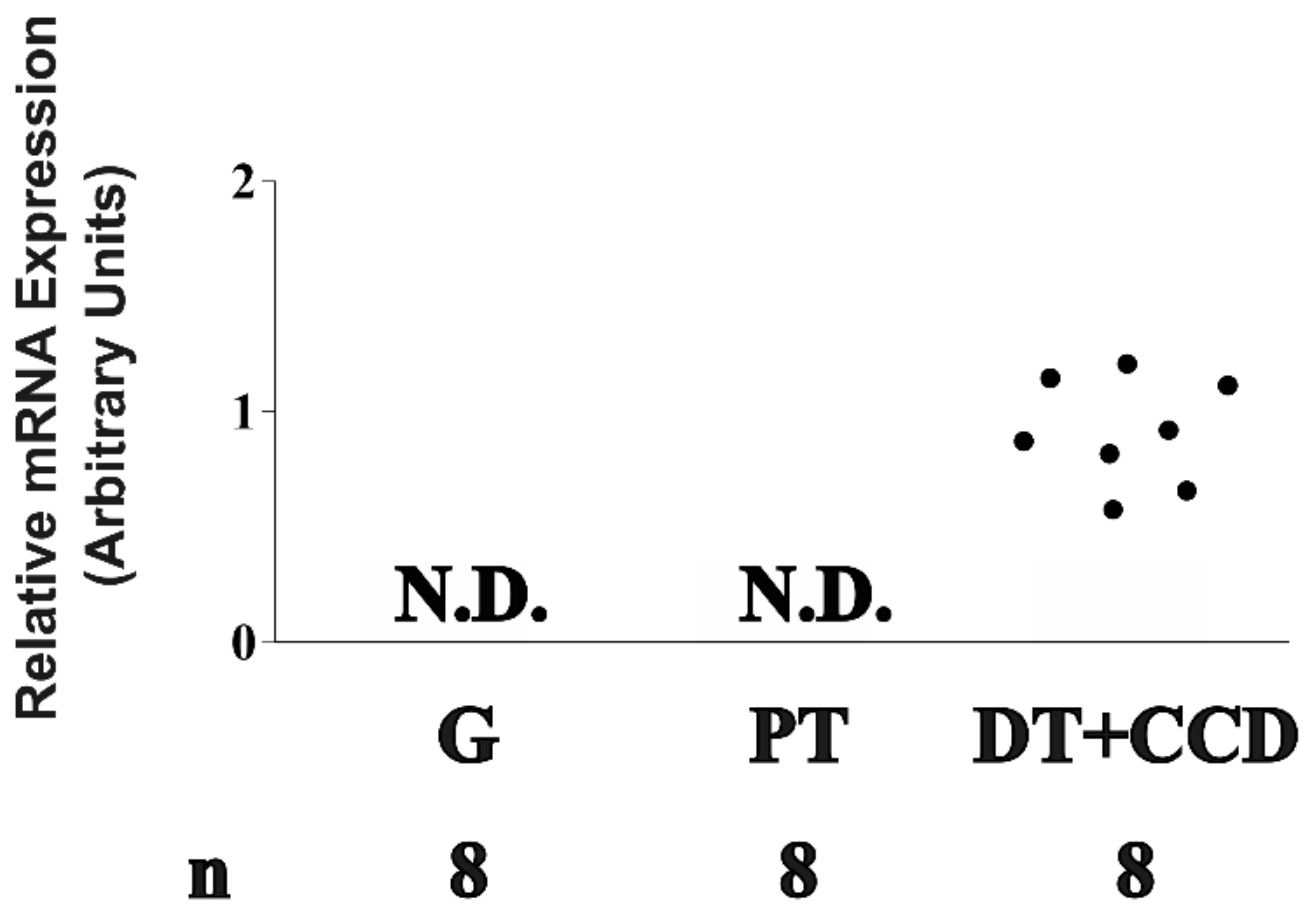
Relative mRNA Expression
(Arbitrary Units)
2
1
0
N.D.
N.D.
G
PT
DT+CCD
n
8
8
8

E

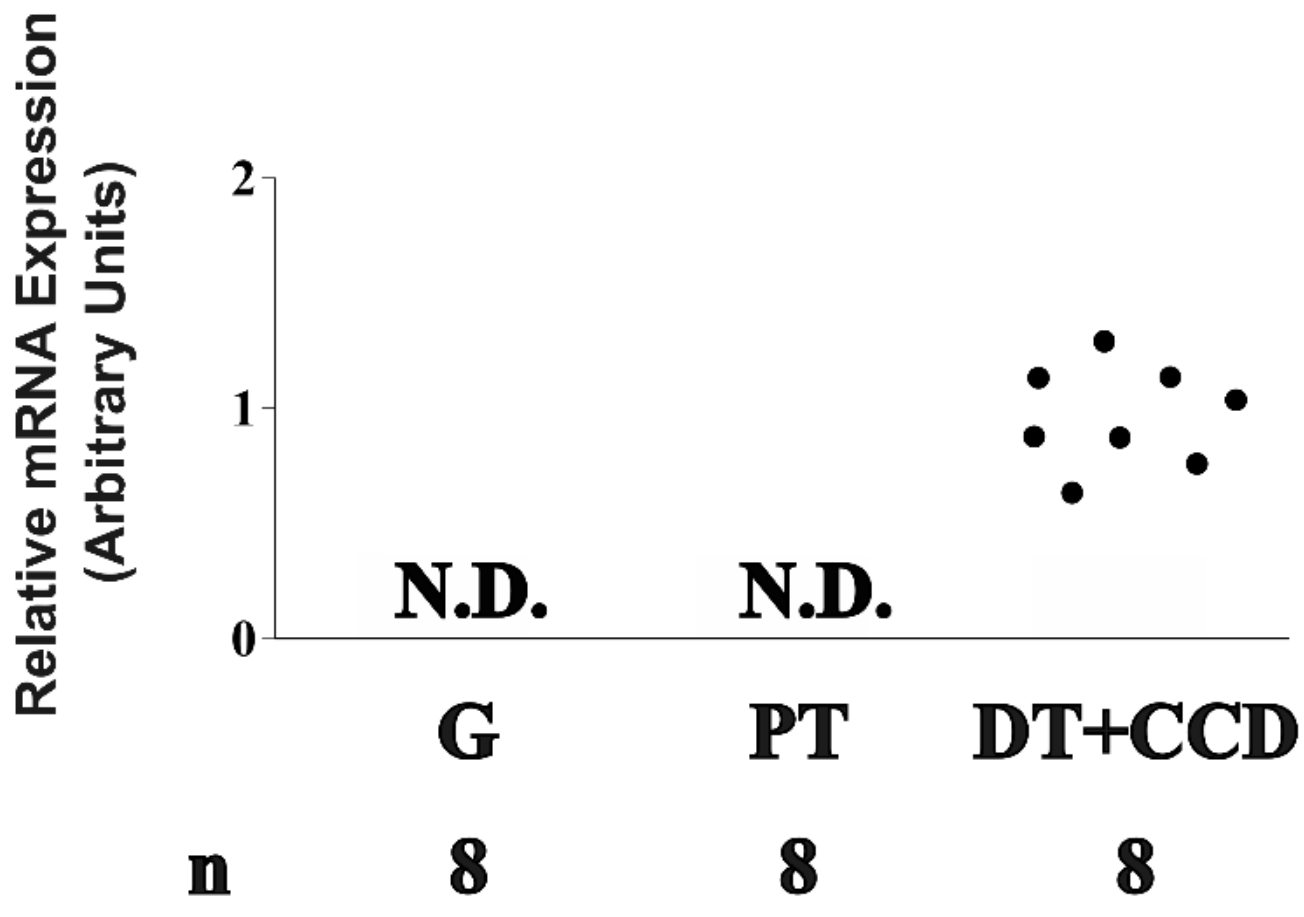
Relative mRNA Expression
(Arbitrary Units)
2
1
0
N.D.
N.D.
G
PT
DT+CCD
n
8
8
8

F

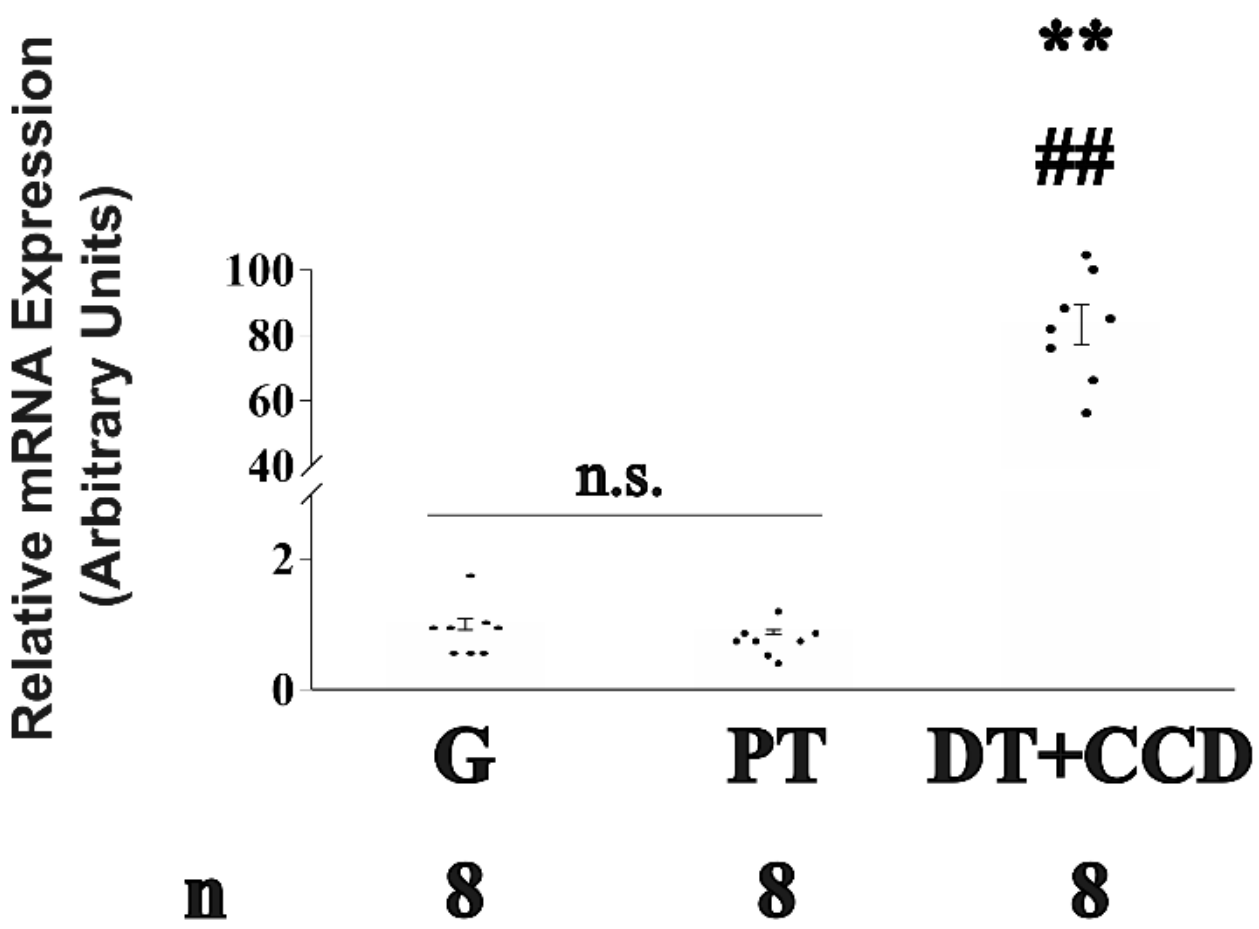
**
##
Relative mRNA Expression
(Arbitrary Units)
100
80
60
40
2
0
n.s.
G
PT
DT+CCD
n
8
8
8

G

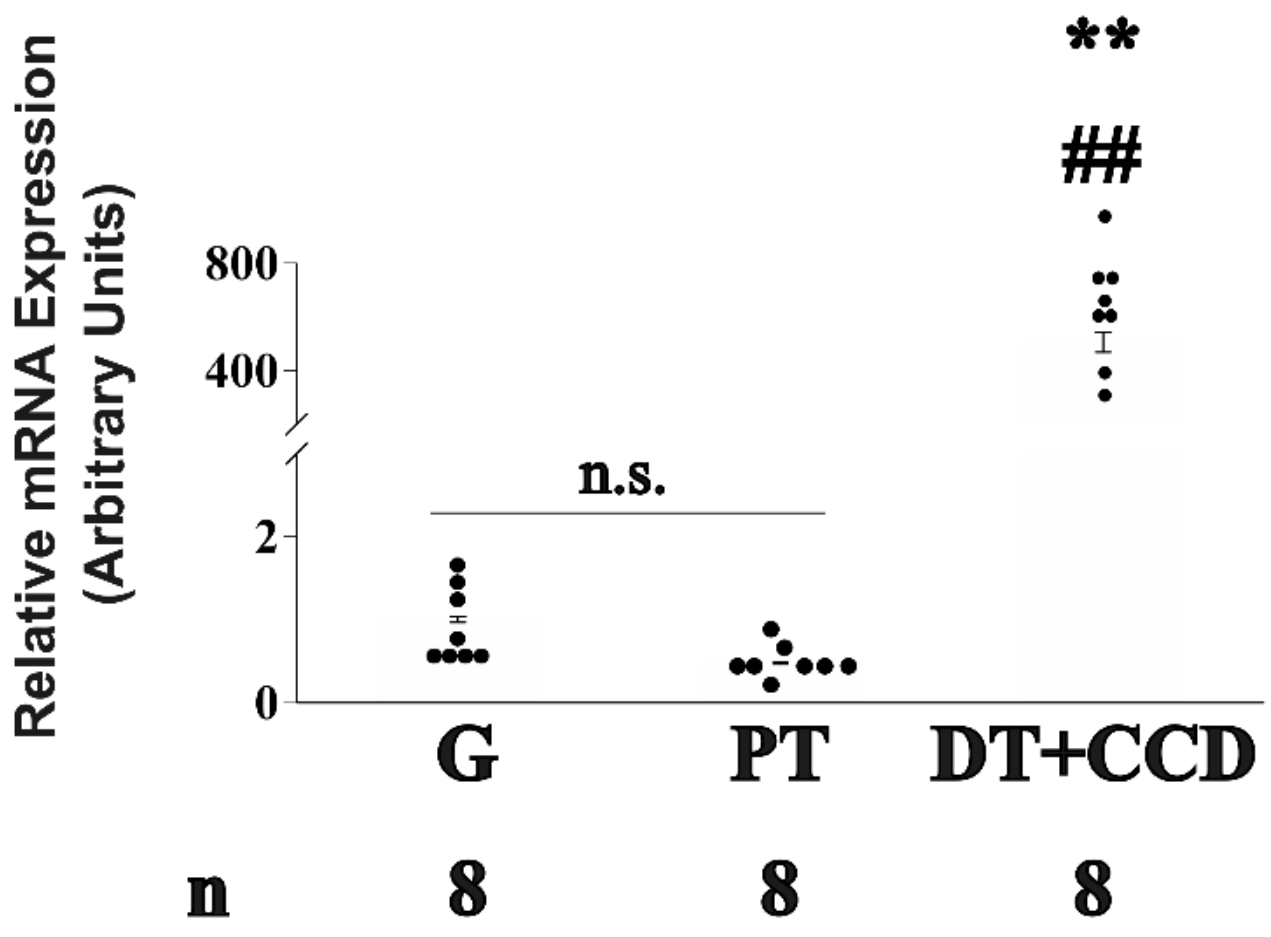

Relative mRNA Expression
(Arbitrary Units)
800
400
2
0
**
##
n.s.
G
PT
DT+CCD
n
8
8
8

H

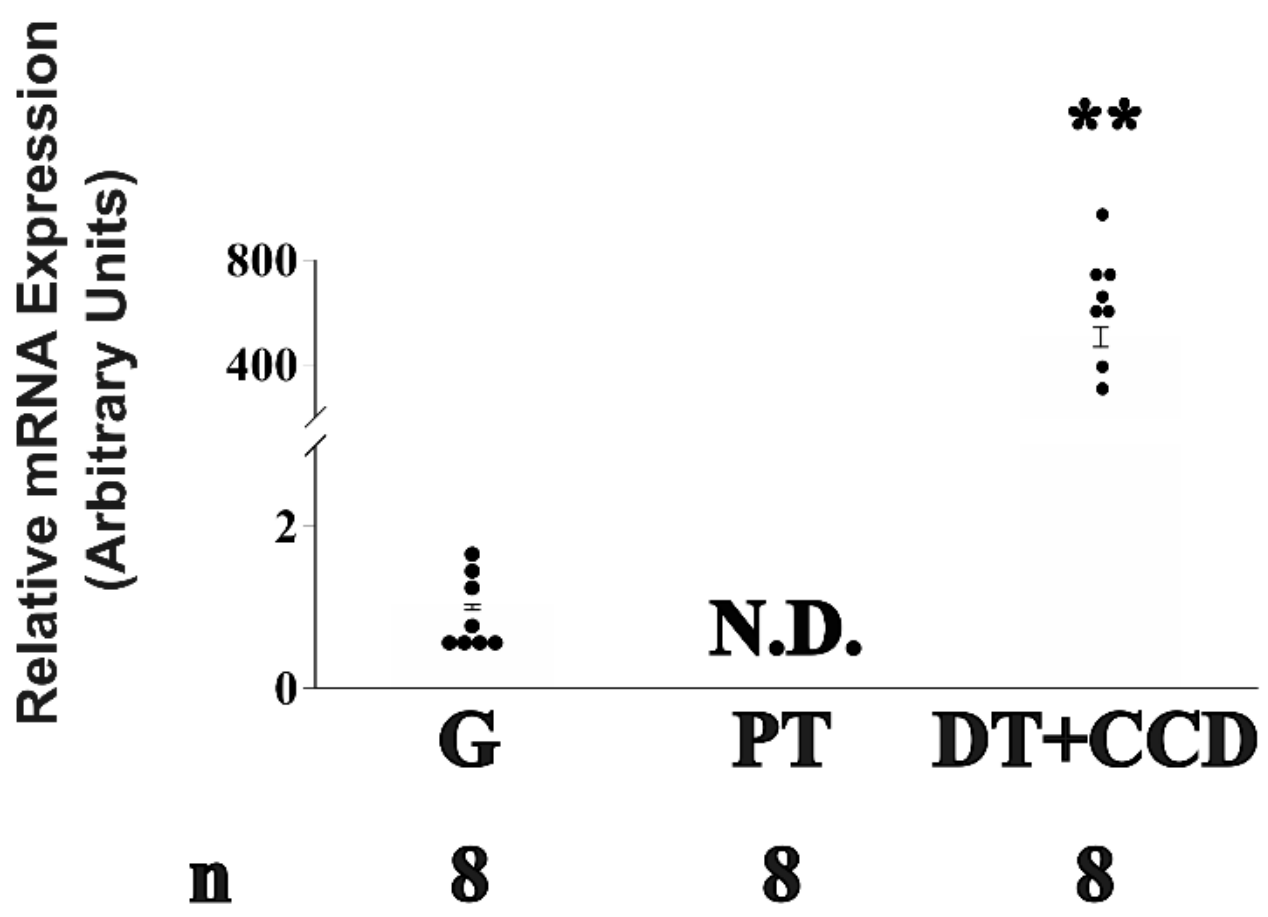

Relative mRNA Expression
(Arbitrary Units)
800
400
2
0
**
N.D.
G
PT
DT+CCD
n
8
8
8

I

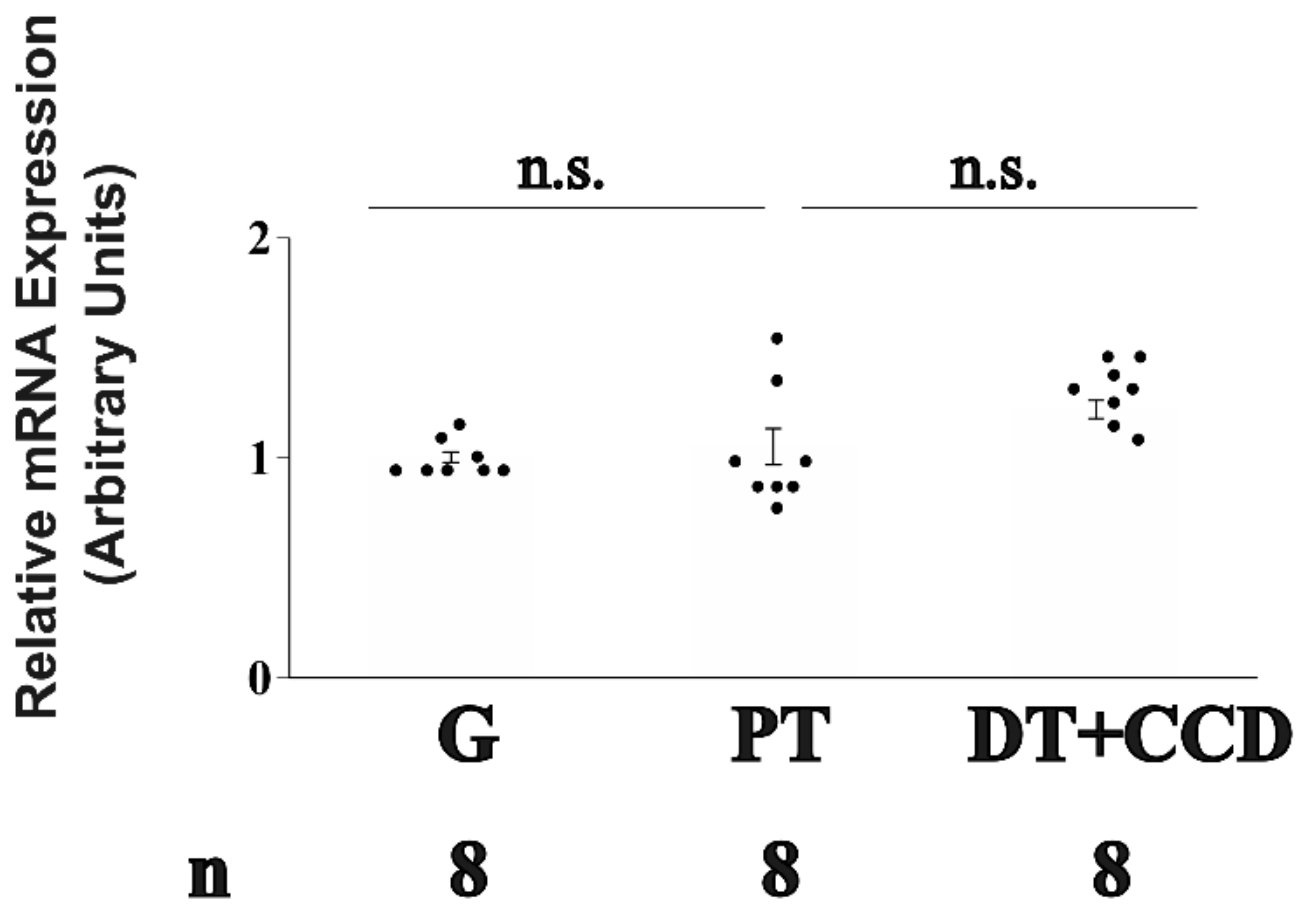
n.s.
n.s.
Relative mRNA Expression
(Arbitrary Units)
2
1
0
G
PT
DT+CCD
n
8
8
8

J

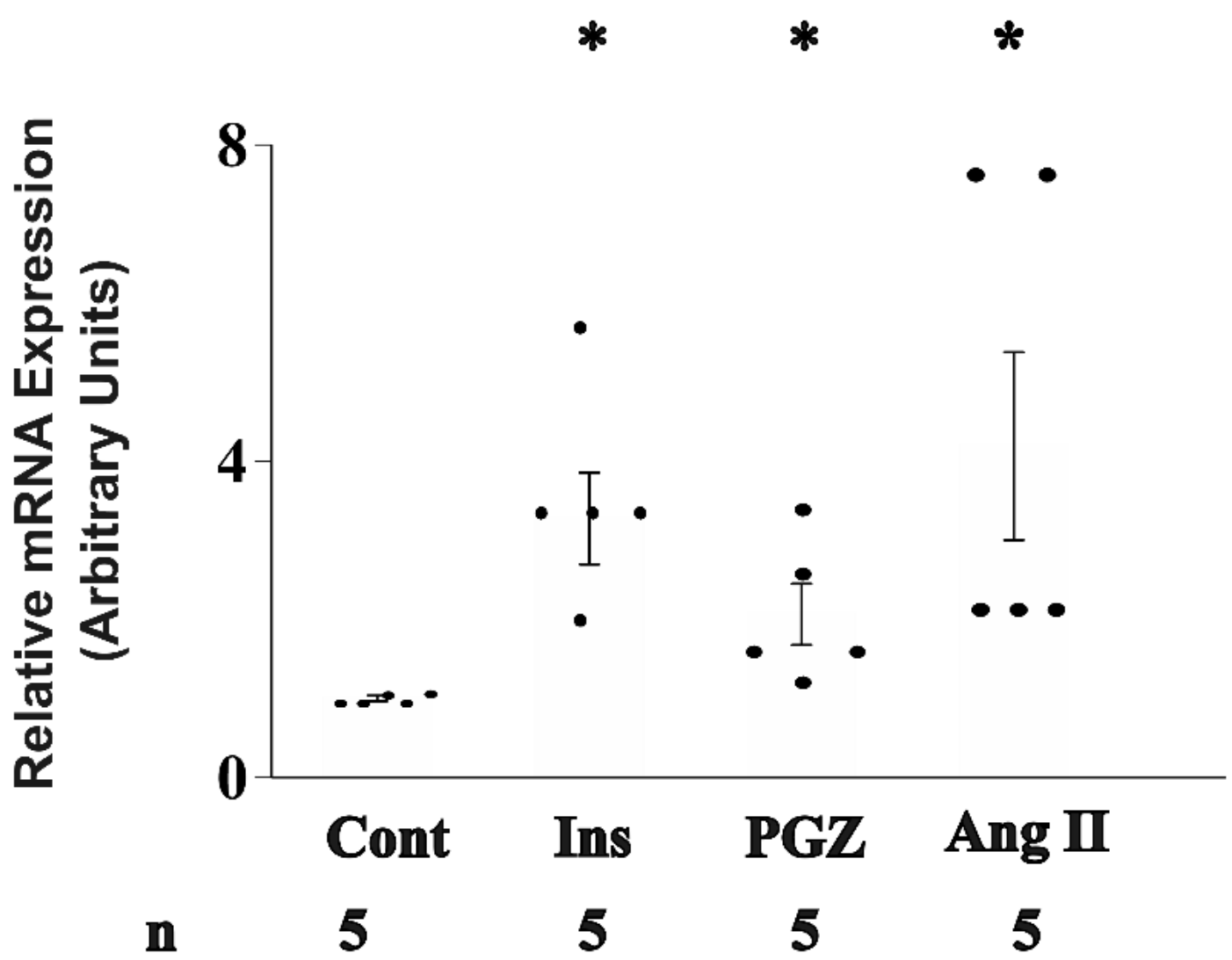
*
*
*
Relative mRNA Expression
(Arbitrary Units)
8
4
0
Cont
Ins
PGZ
Ang II
n
5
5
5
5

K

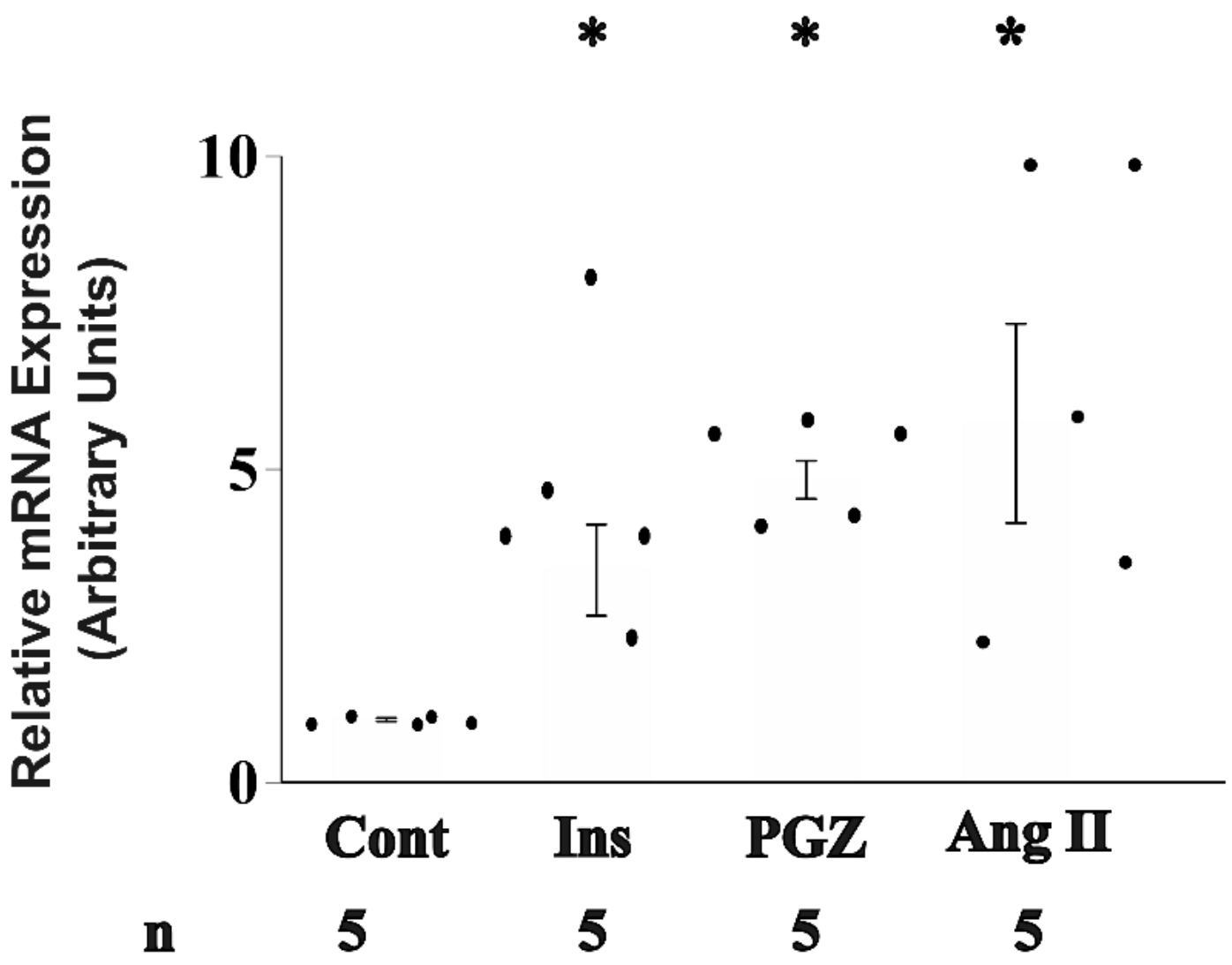
* * *
Relative mRNA Expression
(Arbitrary Units)
10
5
0
Cont
Ins
PGZ
Ang II
n
5
5
5
5

Supplementary Figure 1. mRNA localization in the nephron segments and effects of stimulators on mRNA expression in proximal tubules (PTs)

A) Manually isolated nephron segments: glomerulus (left); proximal tubule, S1+S2 (center); distal tubule (right).

B) Expression of *Nphs2* in the kidney and mRNA expression ratios in glomeruli, PTs, and other compartments isolated manually from fresh renal cortices.

C) Expression of *Slc5a2* in the kidney and mRNA expression ratios in glomeruli, PTs, and other compartments isolated manually from fresh renal cortices.

D) Expression of *Slc12a3* in the kidney and mRNA expression ratios in glomeruli, PTs, and other compartments isolated manually from fresh renal cortices.

E) Expression of *Aqp2* in the kidney and mRNA expression ratios in glomeruli, PTs, and other compartments isolated manually from fresh renal cortices.

F) Expression of *Wnk1* in the kidney and mRNA expression ratios in glomeruli, PTs, and other compartments isolated manually from fresh renal cortices.

G) Expression of *Wnk4* in the kidney and mRNA expression ratios in glomeruli, PTs, and other tubules isolated manually from fresh renal cortex.

H) Expression of *Stk39* in the kidney and mRNA expression ratios in glomeruli, PTs, and other tubules isolated manually from fresh renal cortex.

Stk39 was barely expressed in PTs; thus, it could not be detected.

N.D., not detected.

I) Expression of oxidative stress-responsive kinase 1 (*Oxsr1*) in the kidney and mRNA expression ratios in glomeruli, PTs, and other tubules isolated manually from fresh renal cortex.

J) Modifications of *Wnk1* mRNA expression in PTs by stimulating factors.

K) Modifications of *OXSR1* mRNA expression in PTs by stimulating factors.

Expression levels of each mRNA were normalized to beta-actin and expressed as relative arbitrary units.

B–I; G, glomerulus; PT, proximal tubule; DT+CCD, distal tubule and cortical collecting duct, **p<0.01 versus G, ##p<0.01 versus PT.

B–I; For the segment-specific markers (*Nphs2*, *Slc5a2*, *Slc12a3*, and *Aqp2*), the maximum expression segment for each gene was set to 1.

J–K; AngII, isolated PTs cultured in culture medium with angiotensin II 0.1; nM Cont, no stimulating factor added; Ins, isolated PTs cultured in culture medium with insulin 1 nM; PGZ, isolated PTs cultured in culture medium with pioglitazone 0.3 μM; * p<0.05 versus Cont; Arbitrary units were calculated as mRNA expression normalized to β-actin in the glomeruli at 1.

**p<0.01 versus Cont; data are presented as means ± standard errors.

A

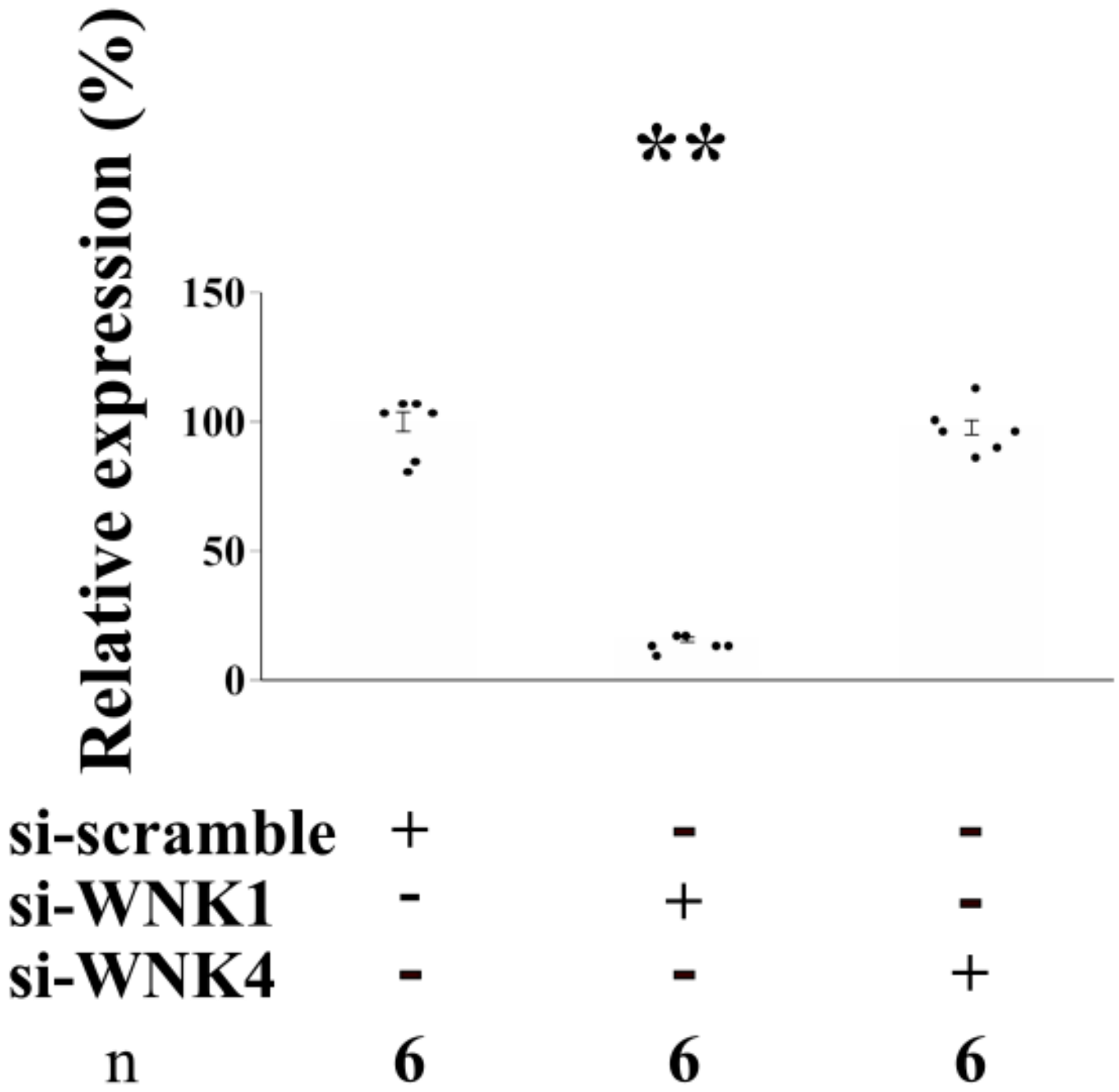

Relative expression (%)
**
150
100
50
0
si-scramble + - -
si-WNK1 - + -
si-WNK4 - - +
n 6 6 6

B

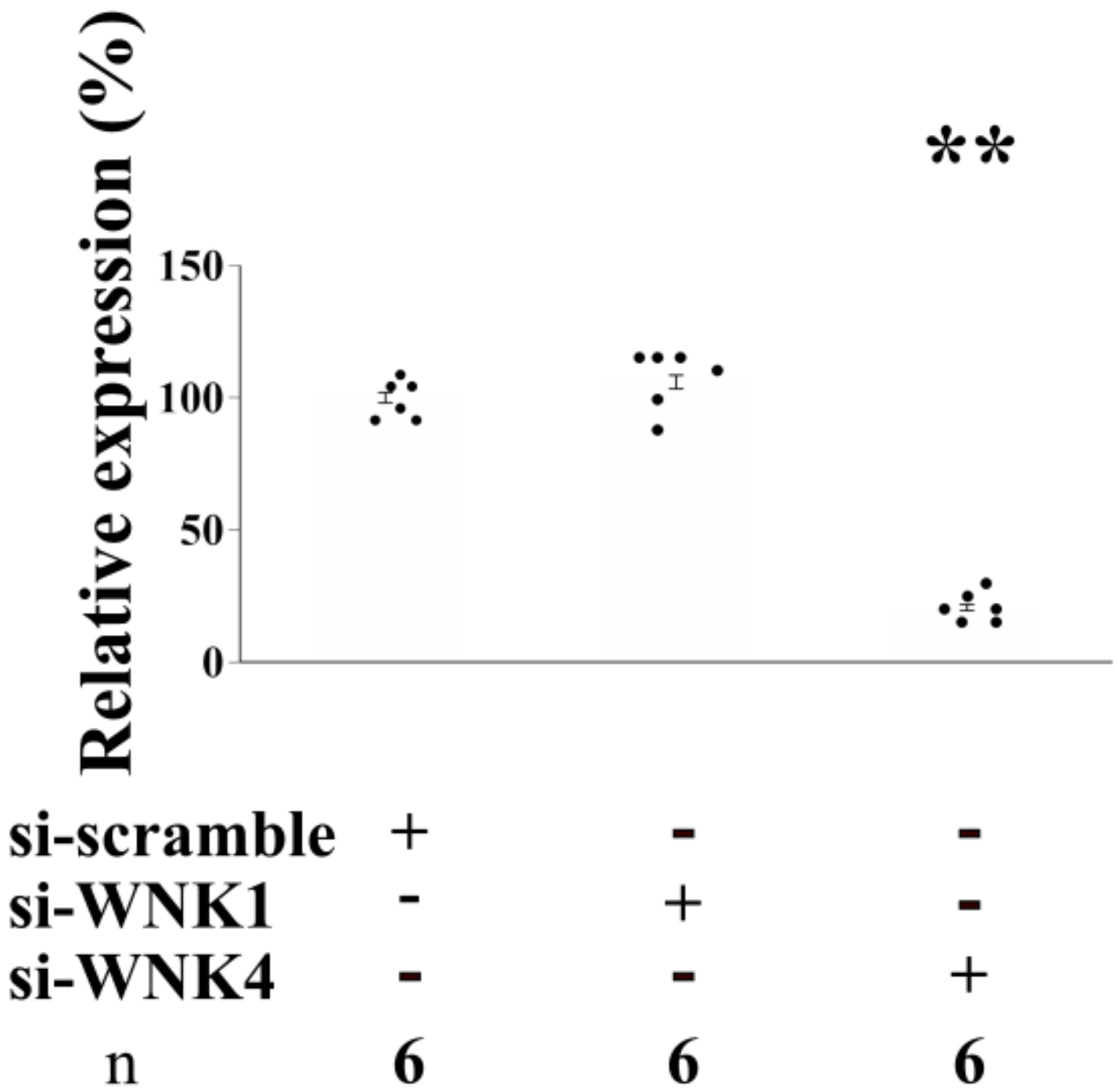

Relative expression (%)
**
150
100
50
0
si-scramble + - -
si-WNK1 - + -
si-WNK4 - - +
n 6 6 6

C

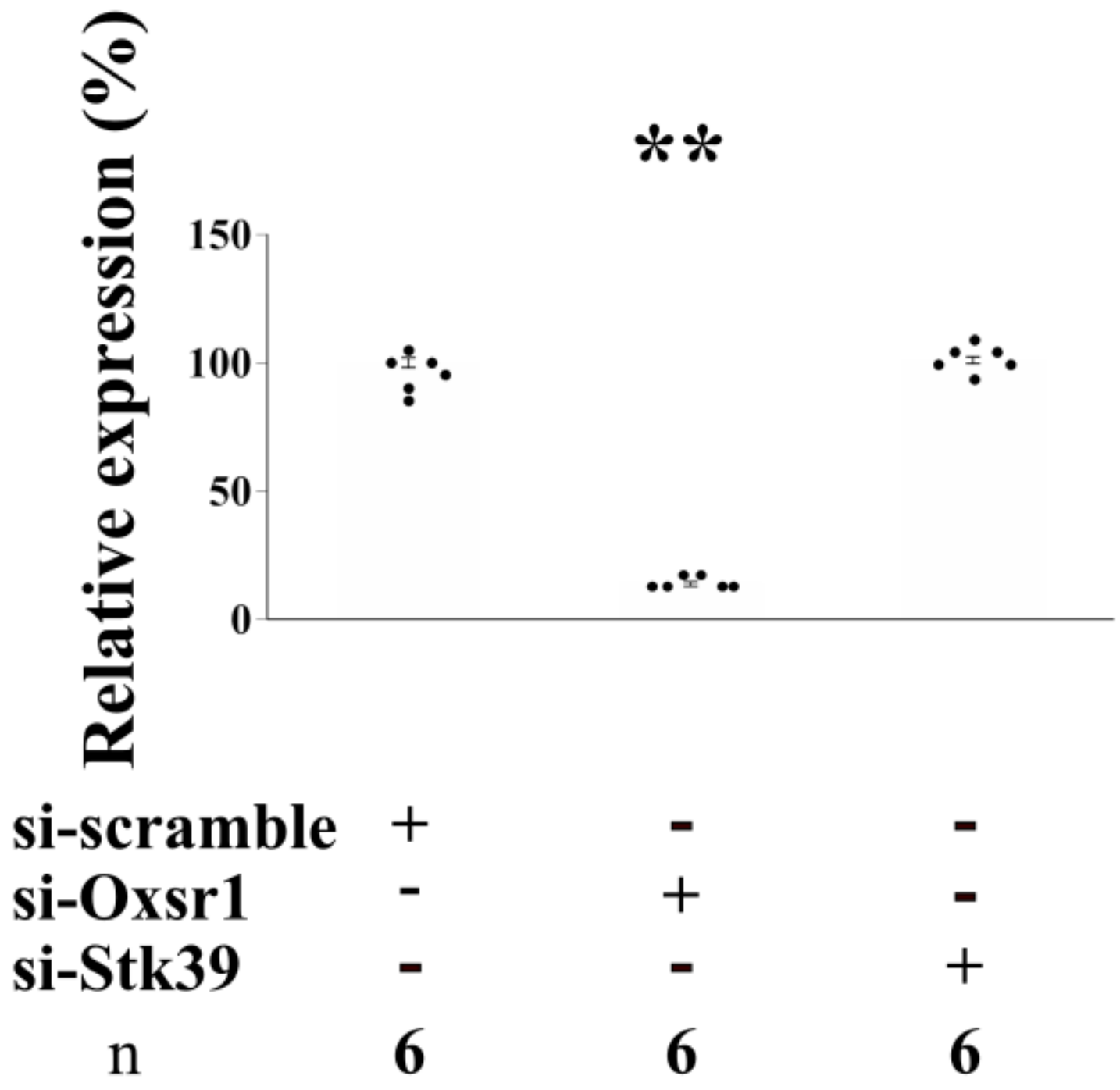
**
150
100
50
0
Relative expression (%)
si-scramble + - -
si-Oxsr1 - + -
si-Stk39 - - +
n 6 6 6

D

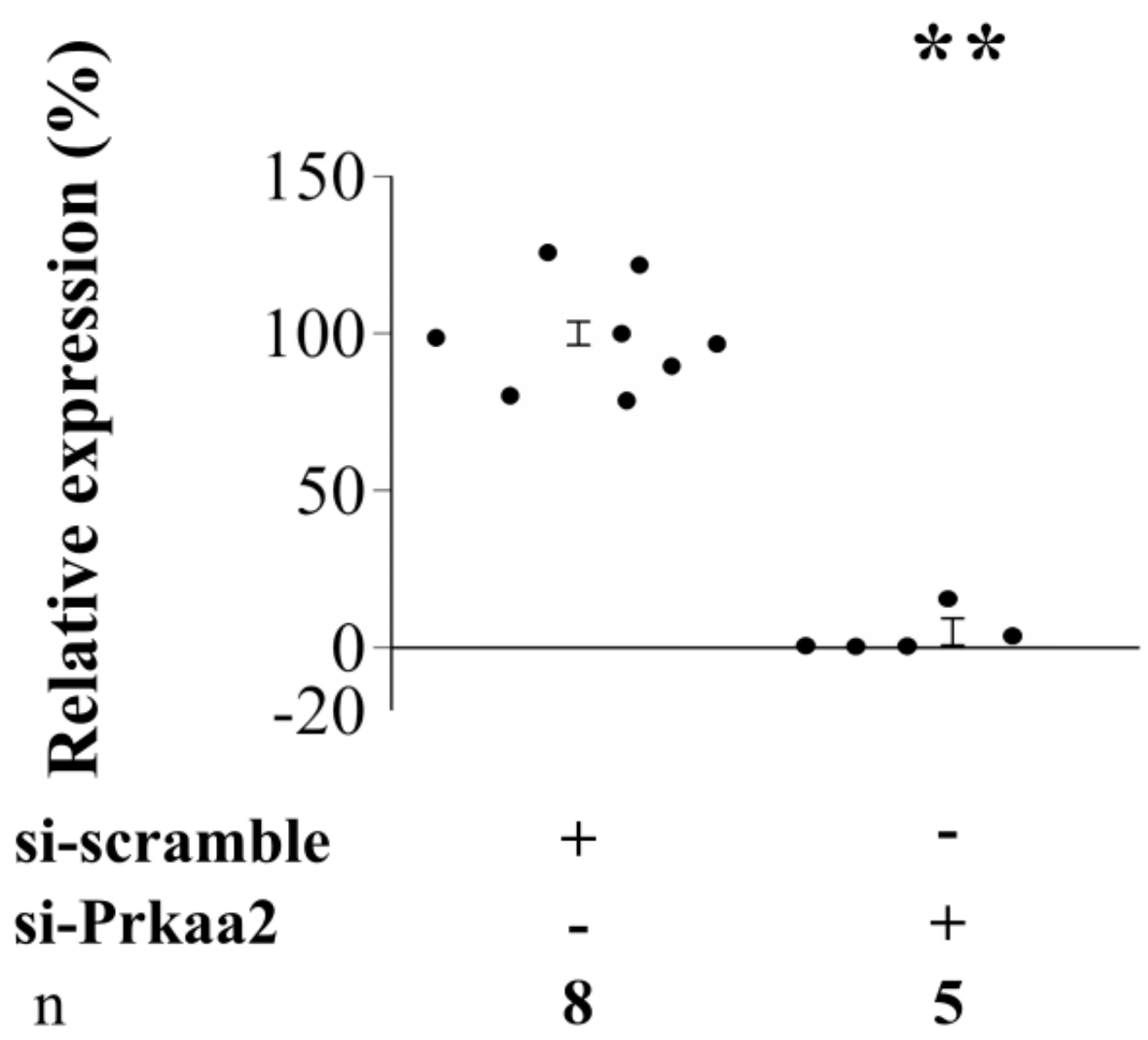
**
150
100
50
0
-20
Relative expression (%)
si-scramble + -
si-Prkaa2 - +
n 8 5

E

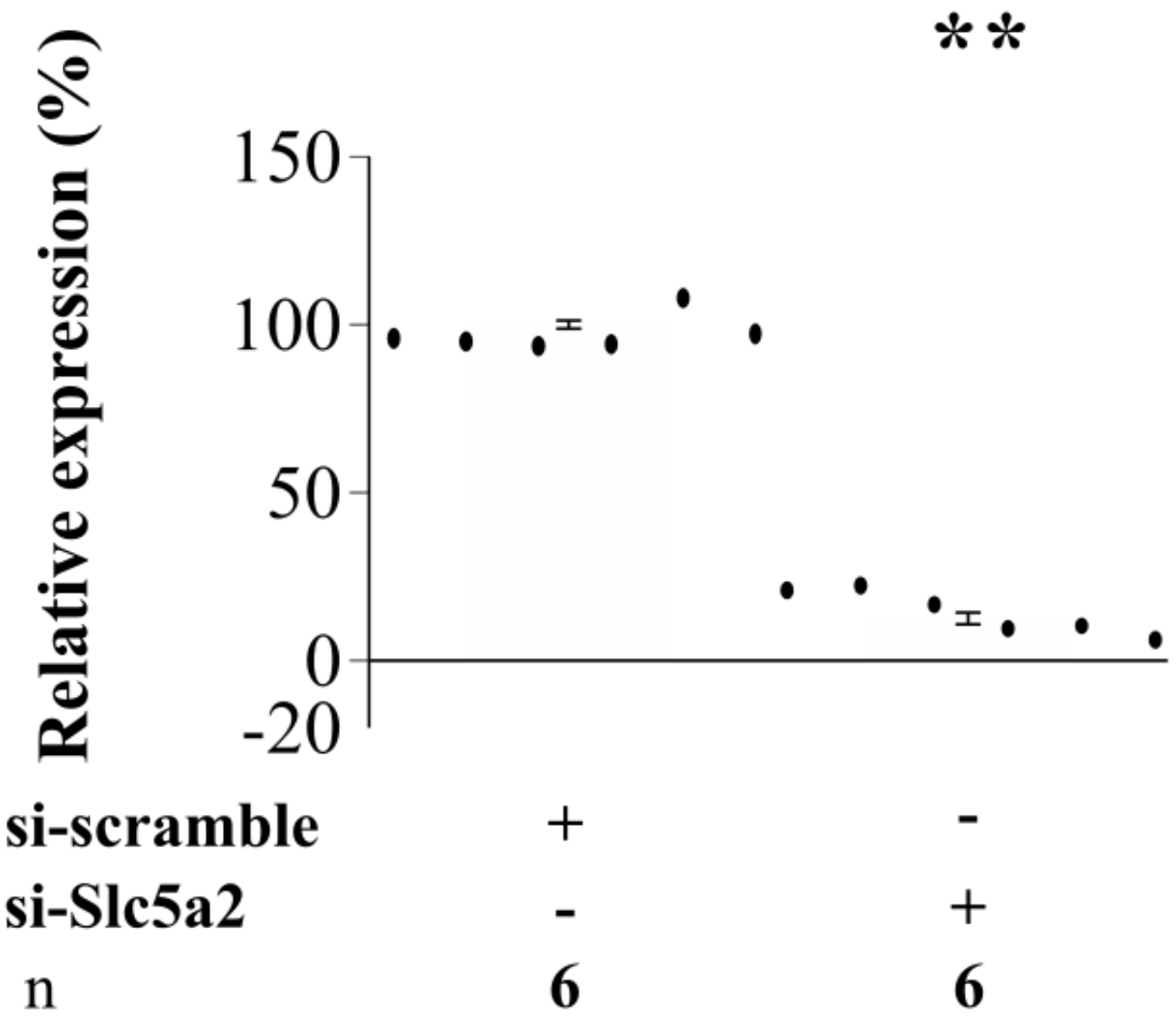
**
Relative expression (%)
150
100
50
0
-20
si-scramble
+
-
si-Slc5a2
-
+
n
6
6

Supplementary Figure 2. Effect of small interfering RNA against with-no-lysine kinase (WNK)1, WNK4, oxidative stress-responsive kinase 1 (OXSR1), *Stk39*, *Prkaa2*/AMPKα2 and *Slc5a2* in isolated rat proximal tubules (PTs)
mRNA expression of (A) *Wnk1*, (B) *Wnk4*, (C) *Oxsr1* (n = 6), (D) *Prkaa2/Ampkα2* (n = 5–8) and (E) *Slc5a2* (n =6).
**p<0.01 versus control PTs treated with scrambled siRNA. As illustrated in Supplementary Figure 1C, *Stk39* was scarcely expressed in the proximal tubules and, therefore, could not be detected; we did not observe changes in the mRNA expression of *Stk39*. Data are presented as means ± standard errors.

A

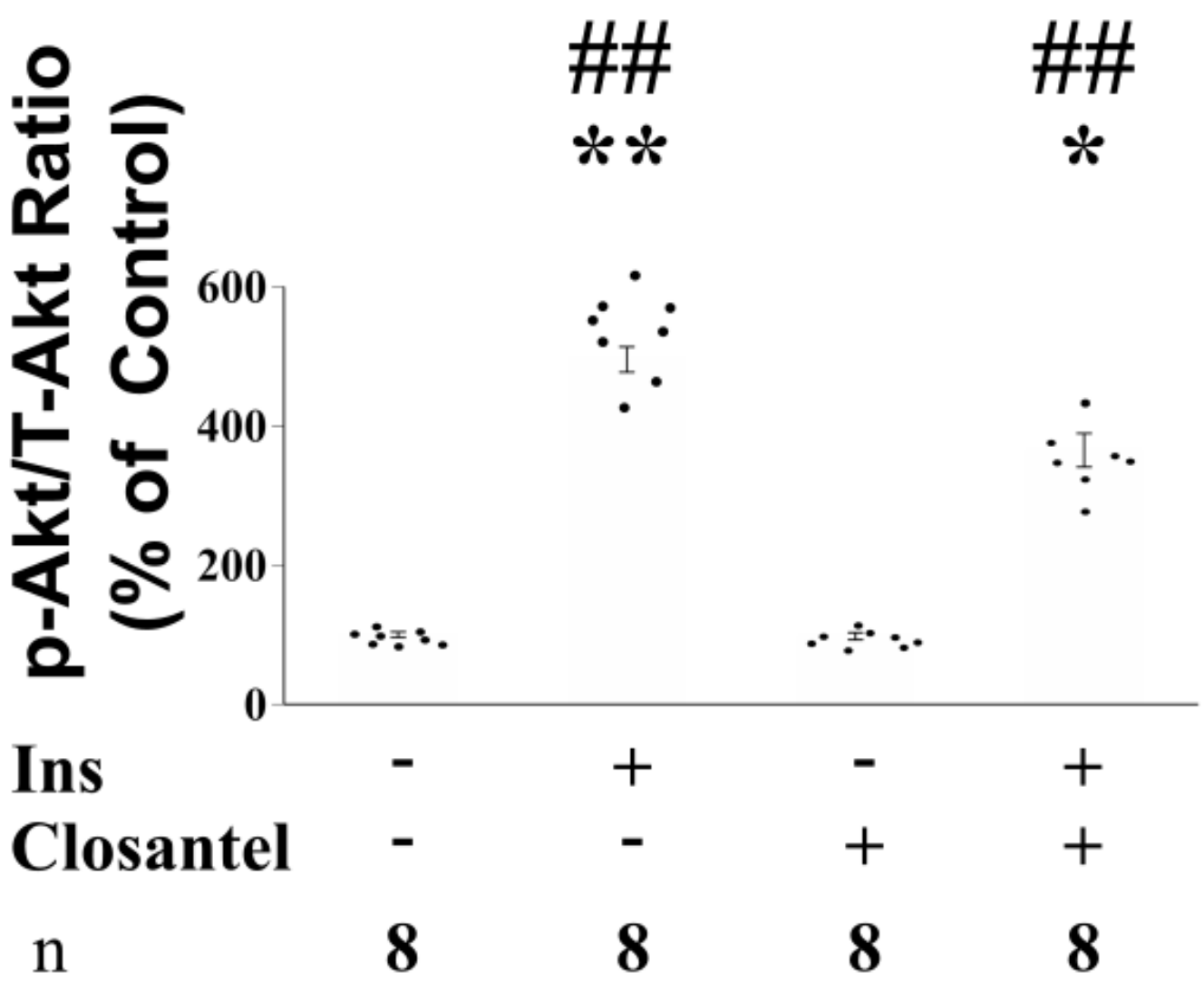

p-Akt/T-Akt Ratio
(% of Control)
##
**
##
*
600
400
200
0
Ins - + - +
Closantel - - + +
n 8 8 8 8

B

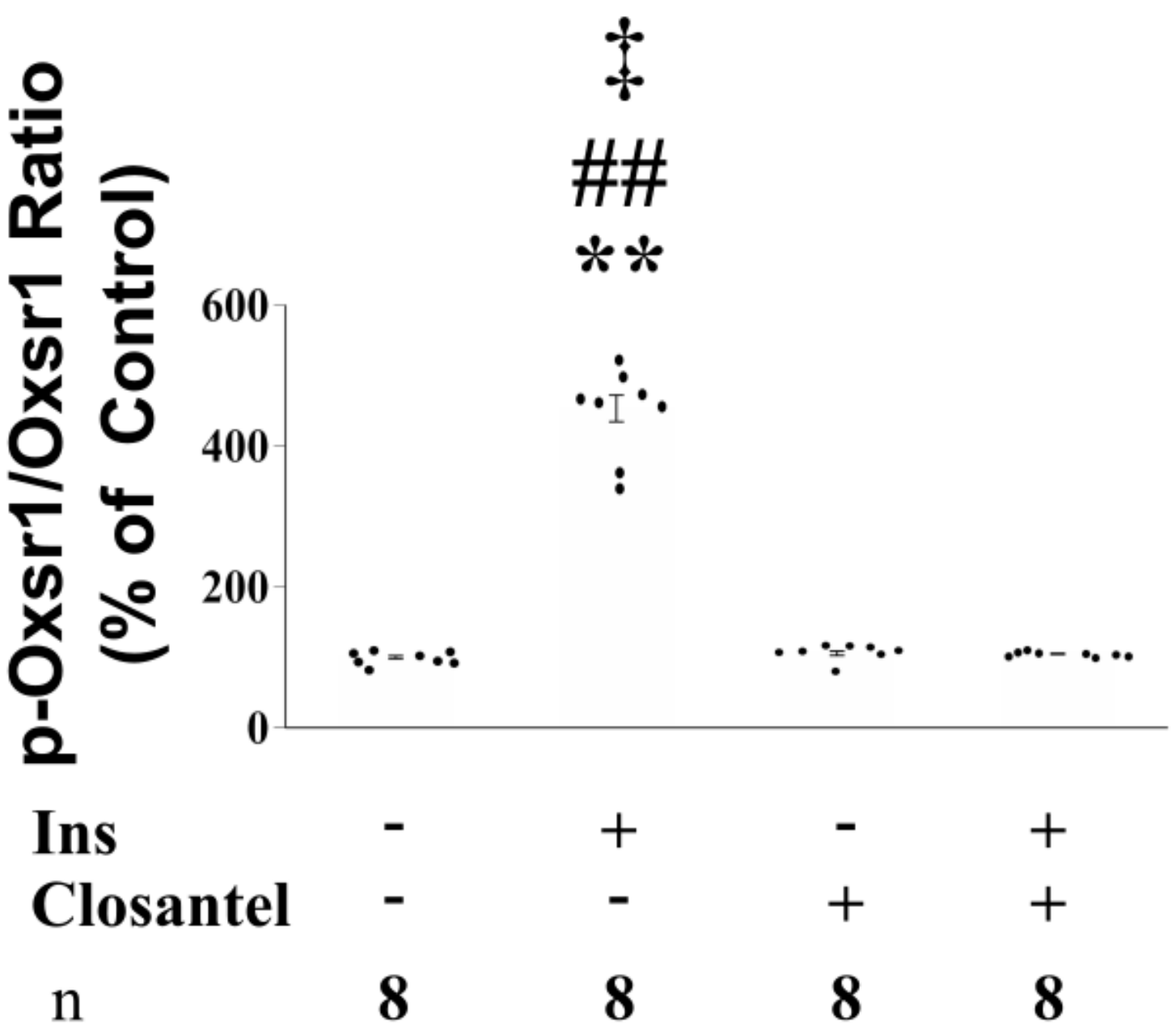

p-Oxsr1/Oxsr1 Ratio
(% of Control)
‡
##
**
600
400
200
0
Ins - + - +
Closantel - - + +
n 8 8 8 8

C

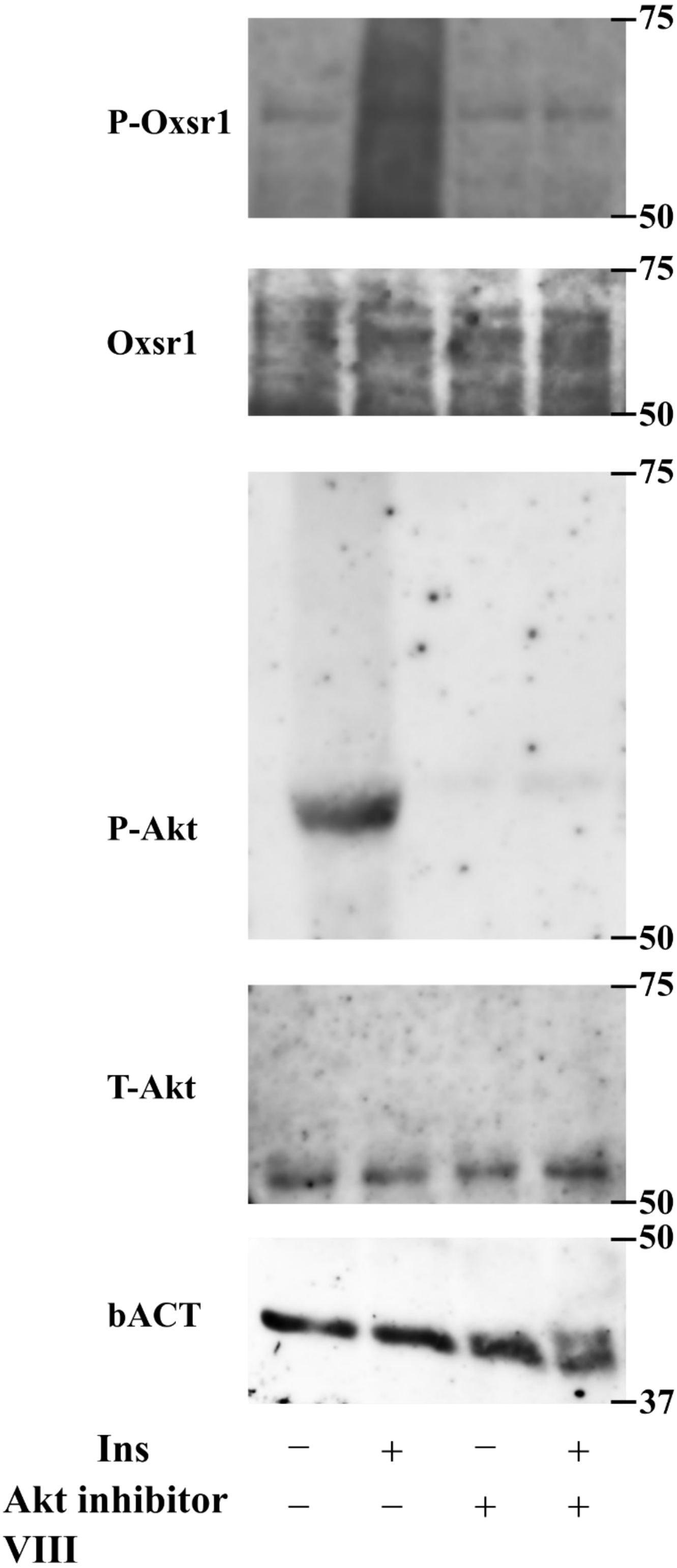
P-Oxsr1
Oxsr1
P-Akt
T-Akt
bACT
75
50
75
50
75
50
75
50
50
37
Ins
−
+
−
+
Akt inhibitor VIII
−
−
+
+

D

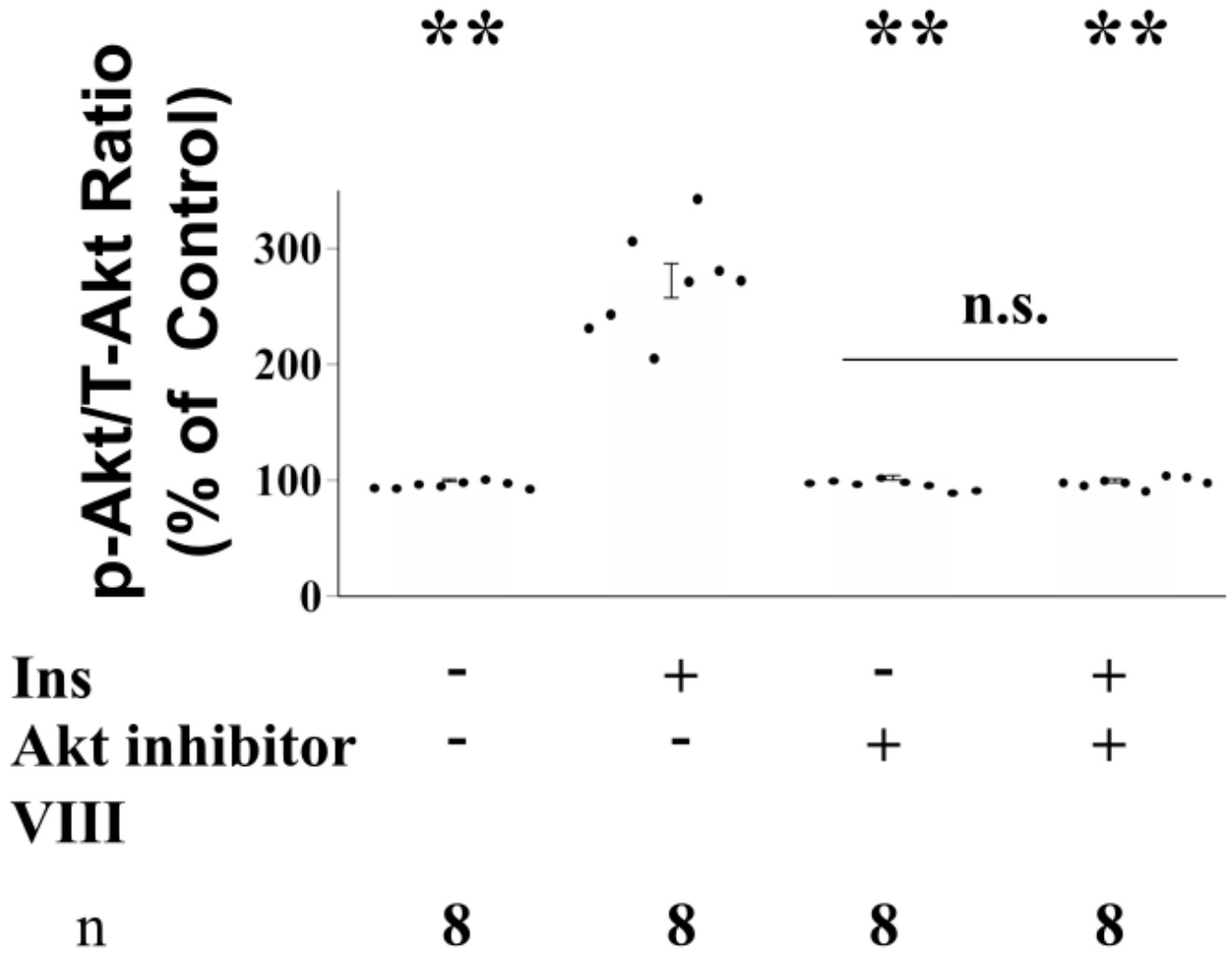
**
**
**
p-Akt/T-Akt Ratio
(% of Control)
300
200
100
0
n.s.
Ins - + - +
Akt inhibitor VIII - - + +
n 8 8 8 8

E

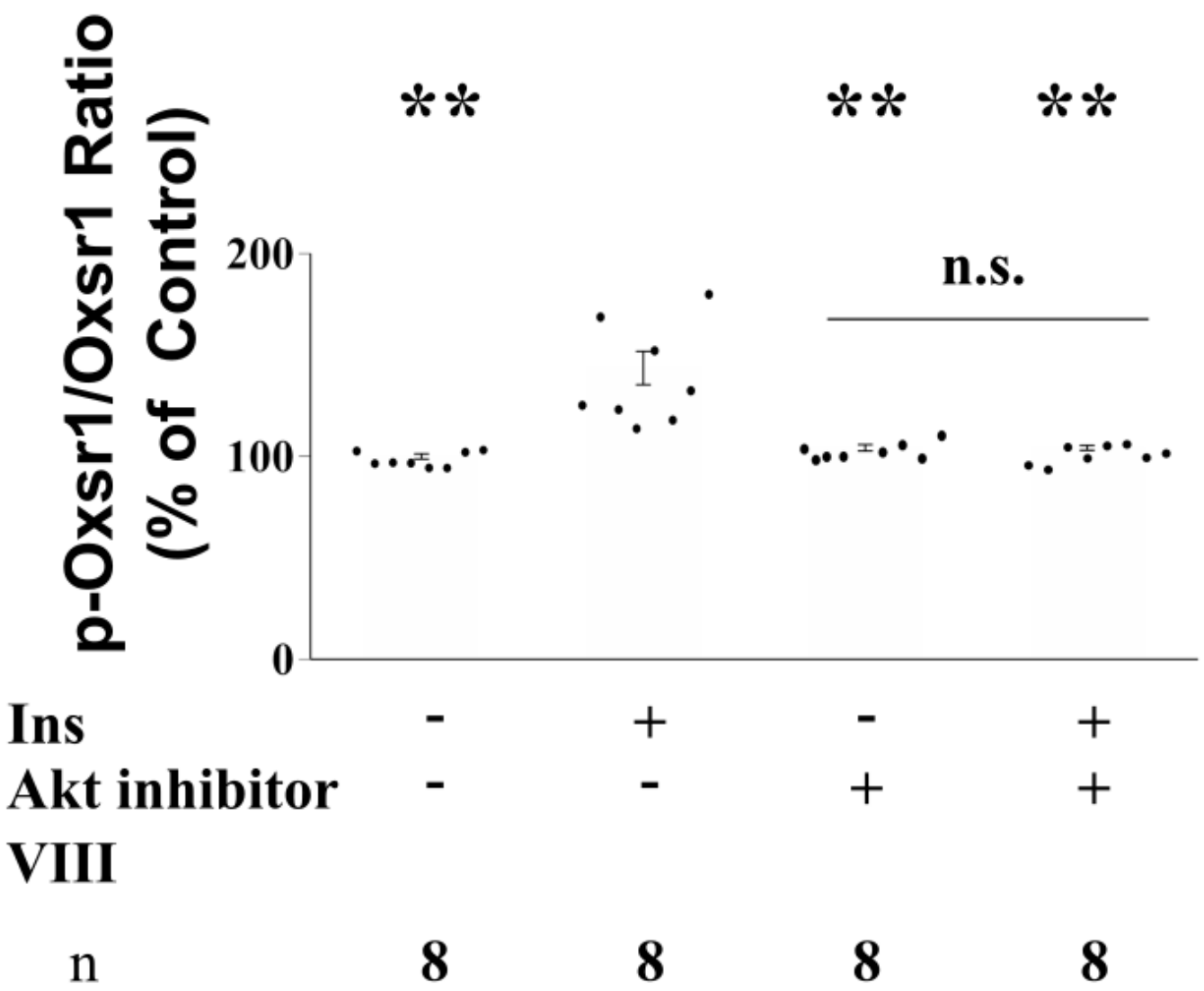
**
**
**
p-Oxsr1/Oxsr1 Ratio
(% of Control)
200
100
0
n.s.
Ins - + - +
Akt inhibitor VIII - - + +
n 8 8 8 8

F

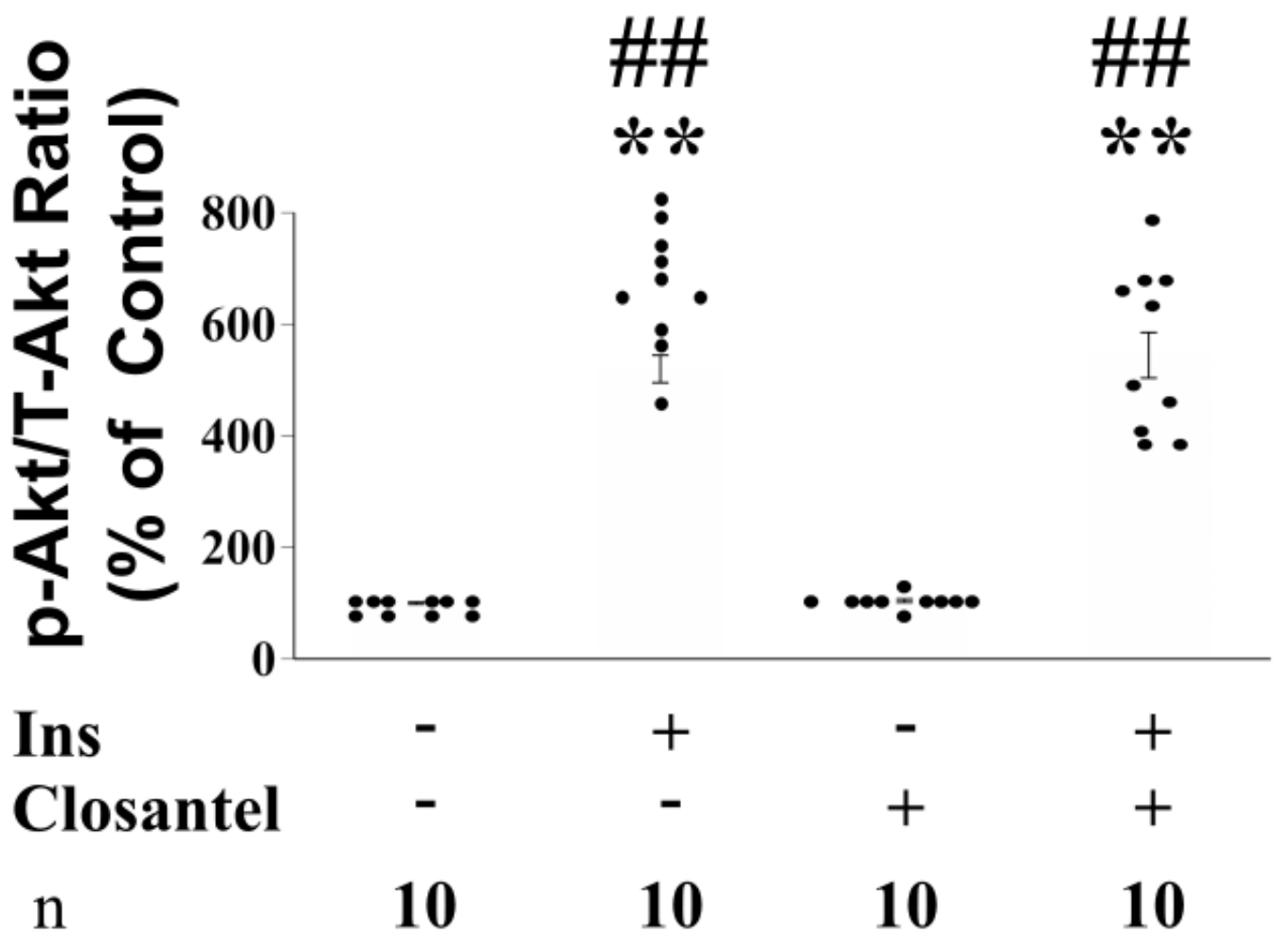
p-Akt/T-Akt Ratio
(% of Control)
##
**
##
**
800
600
400
200
0
Ins - + - +
Closantel - - + +
n 10 10 10 10

G

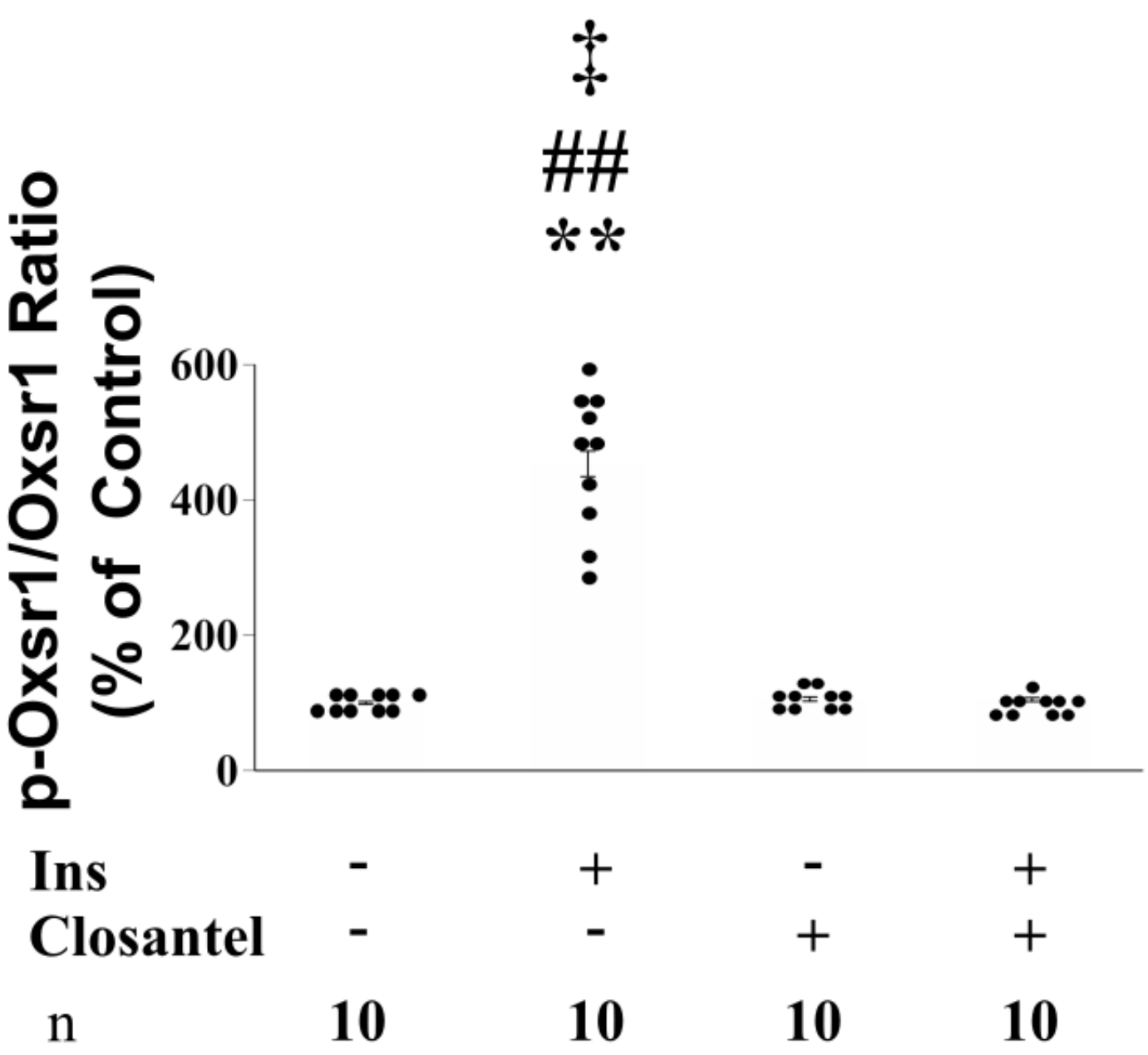
p-Oxsr1/Oxsr1 Ratio
(% of Control)
‡
##
**
600
400
200
0
Ins - + - +
Closantel - - + +
n 10 10 10 10

Supplementary Figure 3. Effect of closantel or AKT inhibitor VIII on insulin-induced AKT and oxidative stress-responsive kinase 1 (OXSR1) phosphorylation

A) Insulin-induced AKT phosphorylation and the effect of closantel on AKT phosphorylation in rat renal cortex (*n* = 8). *p<0.05 versus untreated proximal tubules (PTs), **p<0.01 versus untreated PTs, ##p< 0.01 versus closantel-treated PTs.

B) Insulin-induced OXSR1 phosphorylation and the effect of closantel on OXSR1 phosphorylation in rat renal cortex (*n* = 8). **p<0.01 versus untreated PTs, ##p<0.01 versus closantel-treated PTs, ‡p<0.01 versus closantel and insulin-treated PTs.

C) Effects of AKT inhibitor VIII (AKT-i) on insulin-induced OXSR1 phosphorylation in rat renal cortex.

D) Effect of AKT-i on insulin-induced AKT phosphorylation in rat renal cortex tissue (*n* = 8). **p<0.01 versus insulin-treated PTs.

E) Effect of AKT-i on insulin-induced OXSR1 phosphorylation in rat renal cortex tissue (*n* = 8). **p<0.01 versus insulin-treated PTs.

F) Insulin-induced AKT phosphorylation and the effect of closantel on AKT phosphorylation in the human renal cortex (*n* = 10). *p< 0.05 versus untreated PTs, **p<0.01 versus untreated PTs, ##p<0.01 versus closantel-treated PTs.

G) Insulin-induced OXSR1 phosphorylation and the effect of closantel on OXSR1 phosphorylation in human renal cortex (*n* = 10). **p<0.01 versus untreated PTs, ##p<0.01 versus closantel-treated PTs, ‡ p<0.01 versus closantel and insulin-treated PTs.

Data are presented as means ± standard errors. “n.s.” represents no significant difference.

A

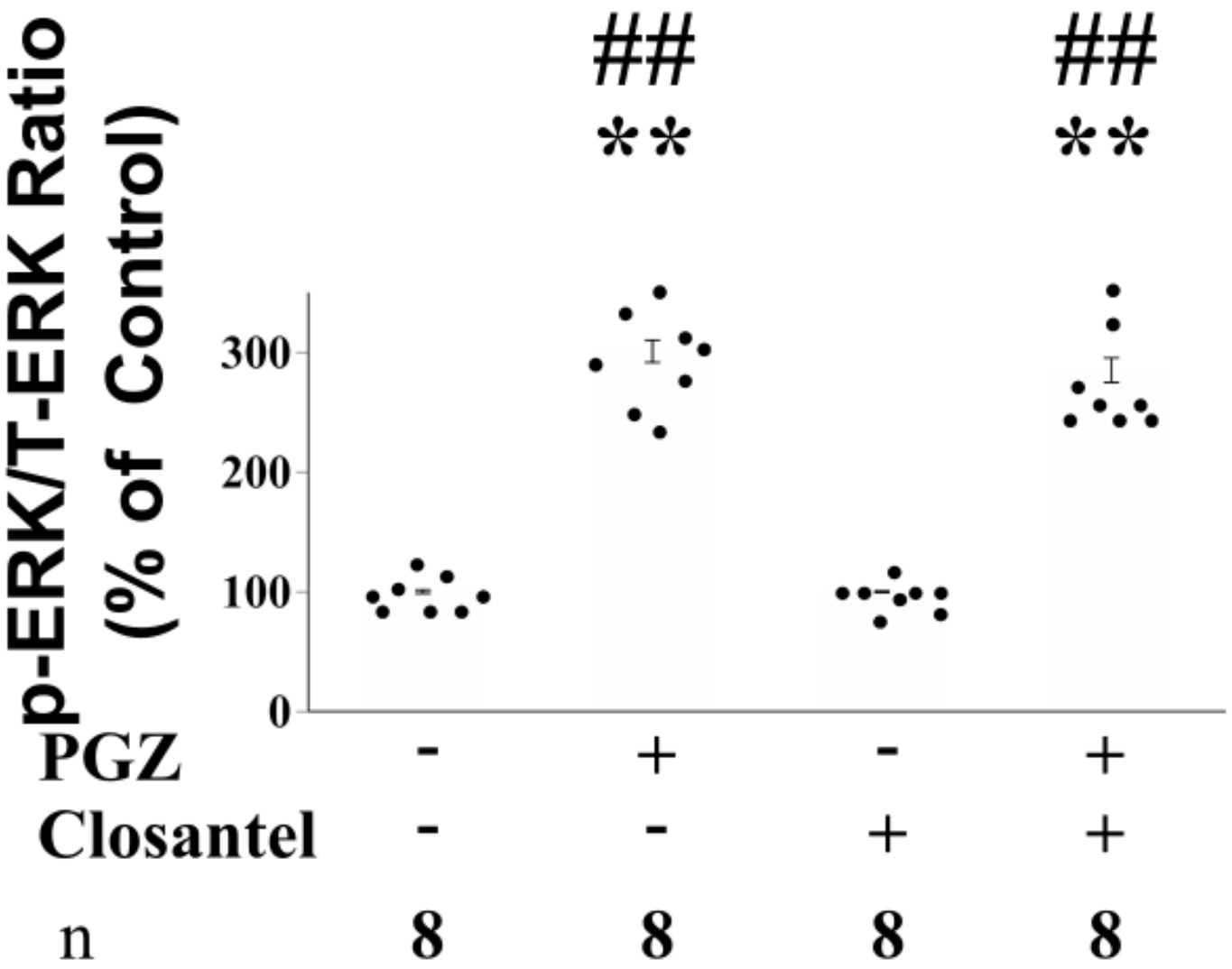
##
**
p-ERK/T-ERK Ratio
(% of Control)
300
200
100
0
PGZ
Closantel
n
-
+
-
+
-
-
+
+
8
8
8
8

B

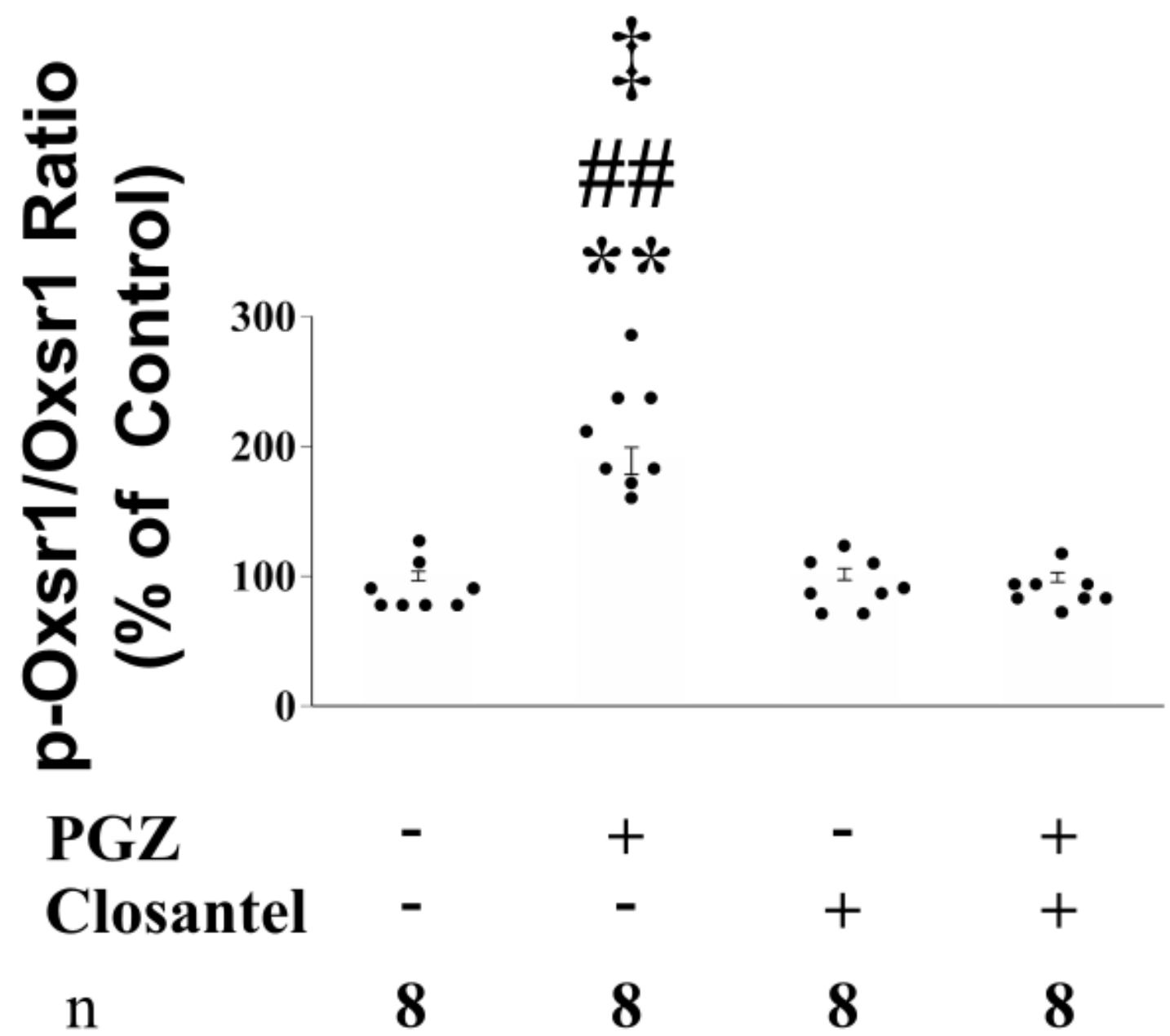
‡
##
**
p-Oxsr1/Oxsr1 Ratio
(% of Control)
300
200
100
0
PGZ
Closantel
n
-
+
-
+
-
-
+
+
8
8
8
8

C

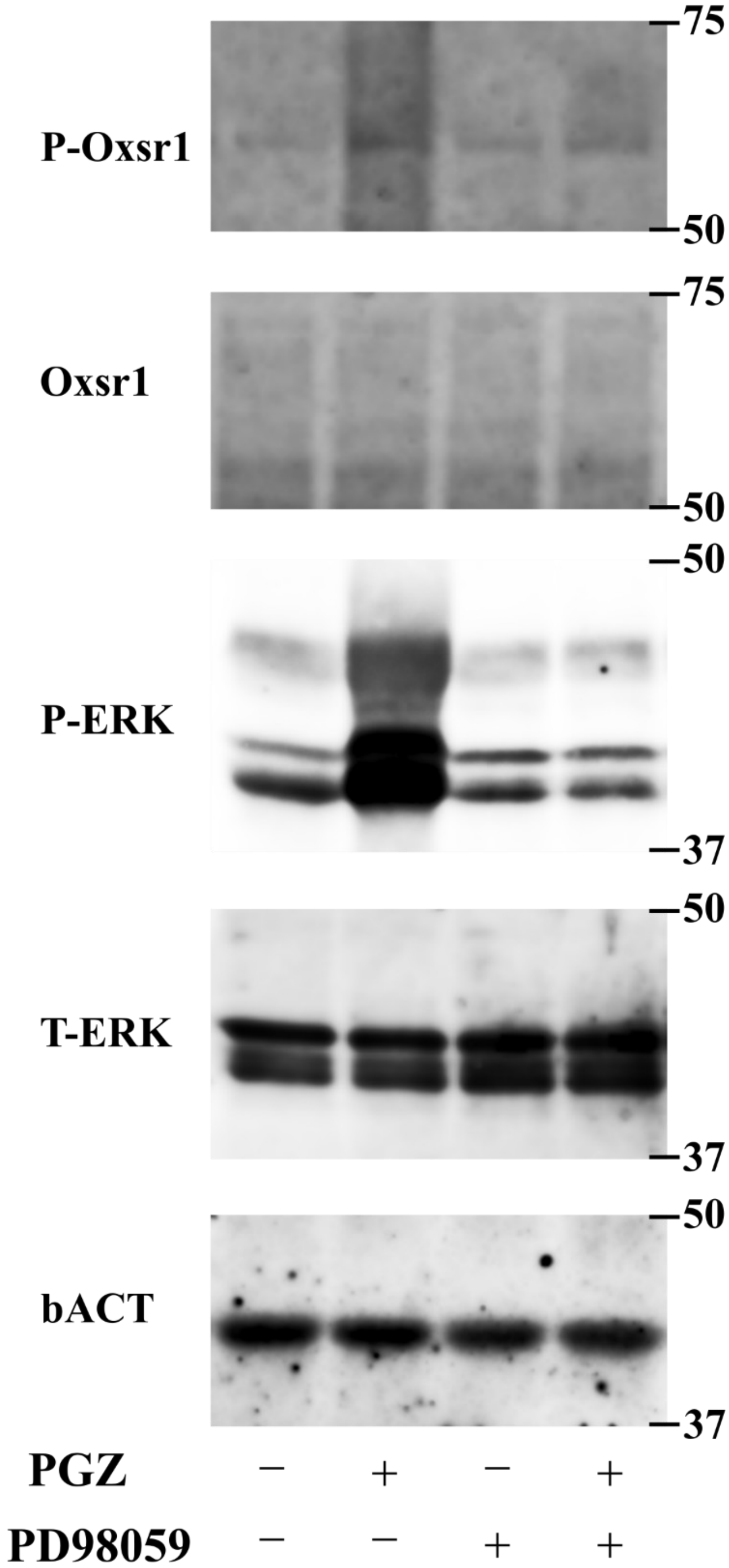

P-Oxsr1
Oxsr1
P-ERK
T-ERK
bACT
75
50
75
50
50
37
50
37
50
37
PGZ
−
+
−
+
PD98059
−
−
+
+

D

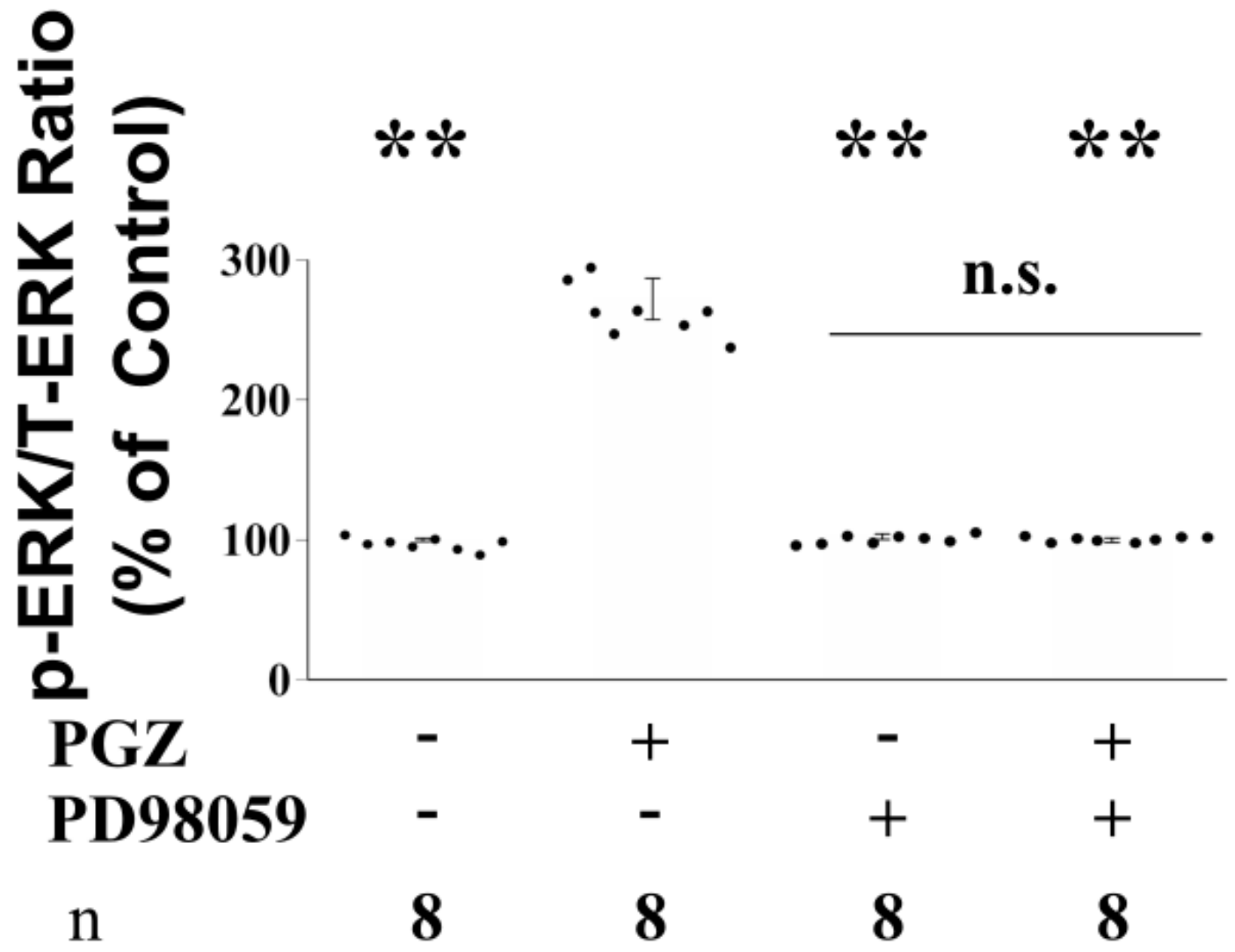

p-ERK/T-ERK Ratio
(% of Control)
**
**
**
n.s.
300
200
100
0
PGZ
-
+
-
+
PD98059
-
-
+
+
n
8
8
8
8

E

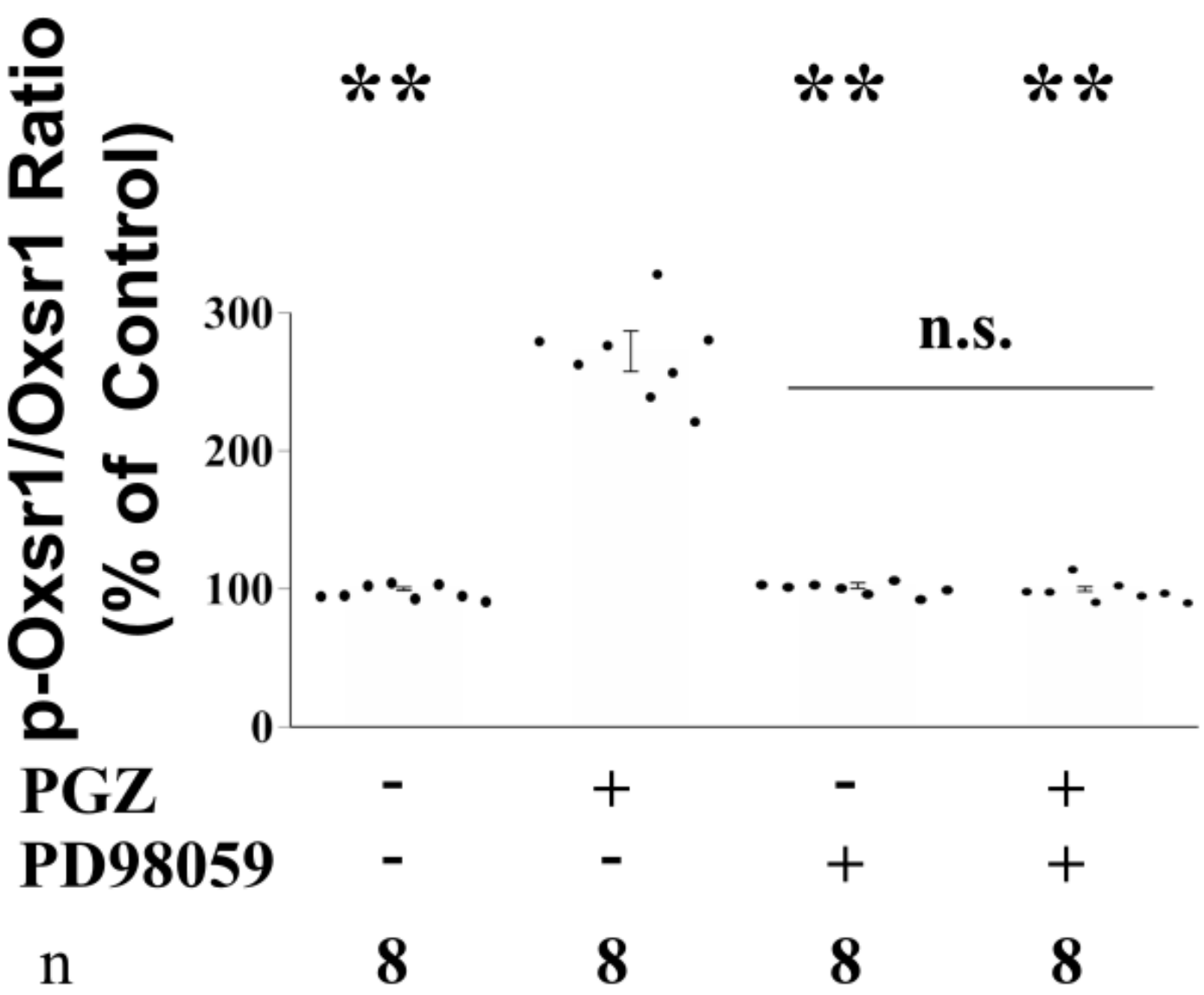

p-Oxsr1/Oxsr1 Ratio
(% of Control)
**
**
**
n.s.
300
200
100
0
PGZ
-
+
-
+
PD98059
-
-
+
+
n
8
8
8
8

F

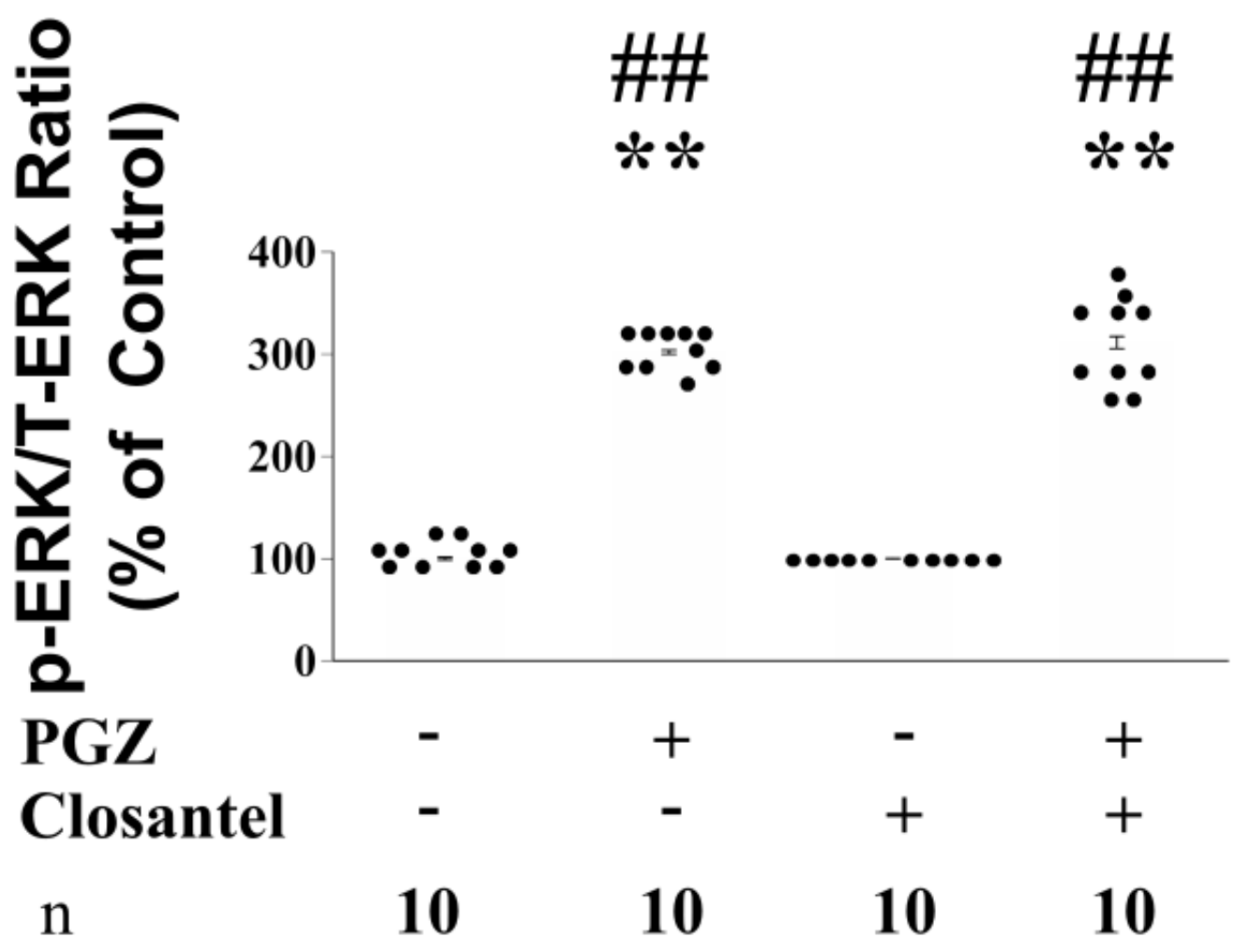
p-ERK/T-ERK Ratio
(% of Control)
400
300
200
100
0
##
**
##
**
PGZ - + - +
Closantel - - + +
n 10 10 10 10

G

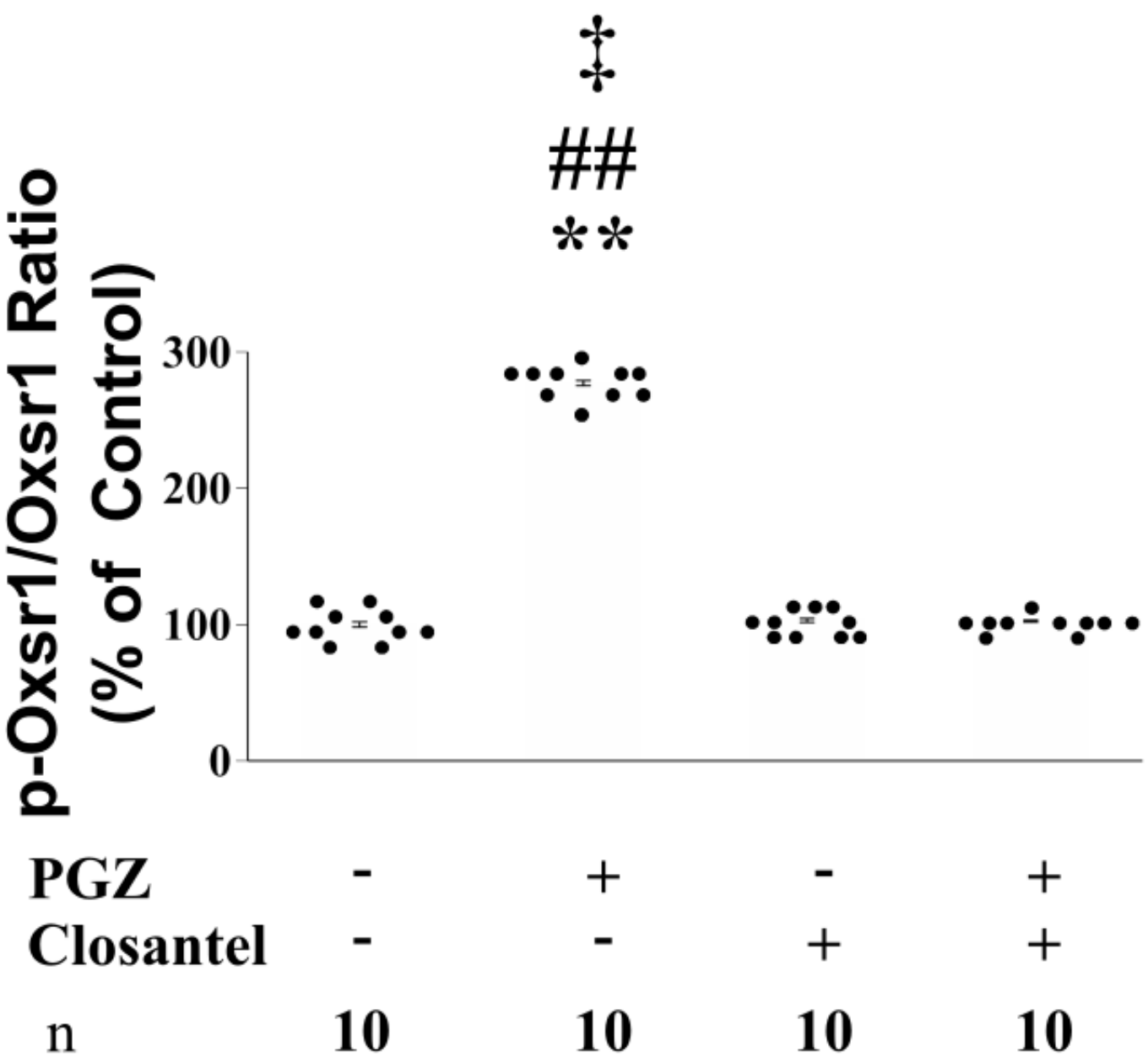
p-Oxsr1/Oxsr1 Ratio
(% of Control)
300
200
100
0
‡
##
**
PGZ - + - +
Closantel - - + +
n 10 10 10 10

H

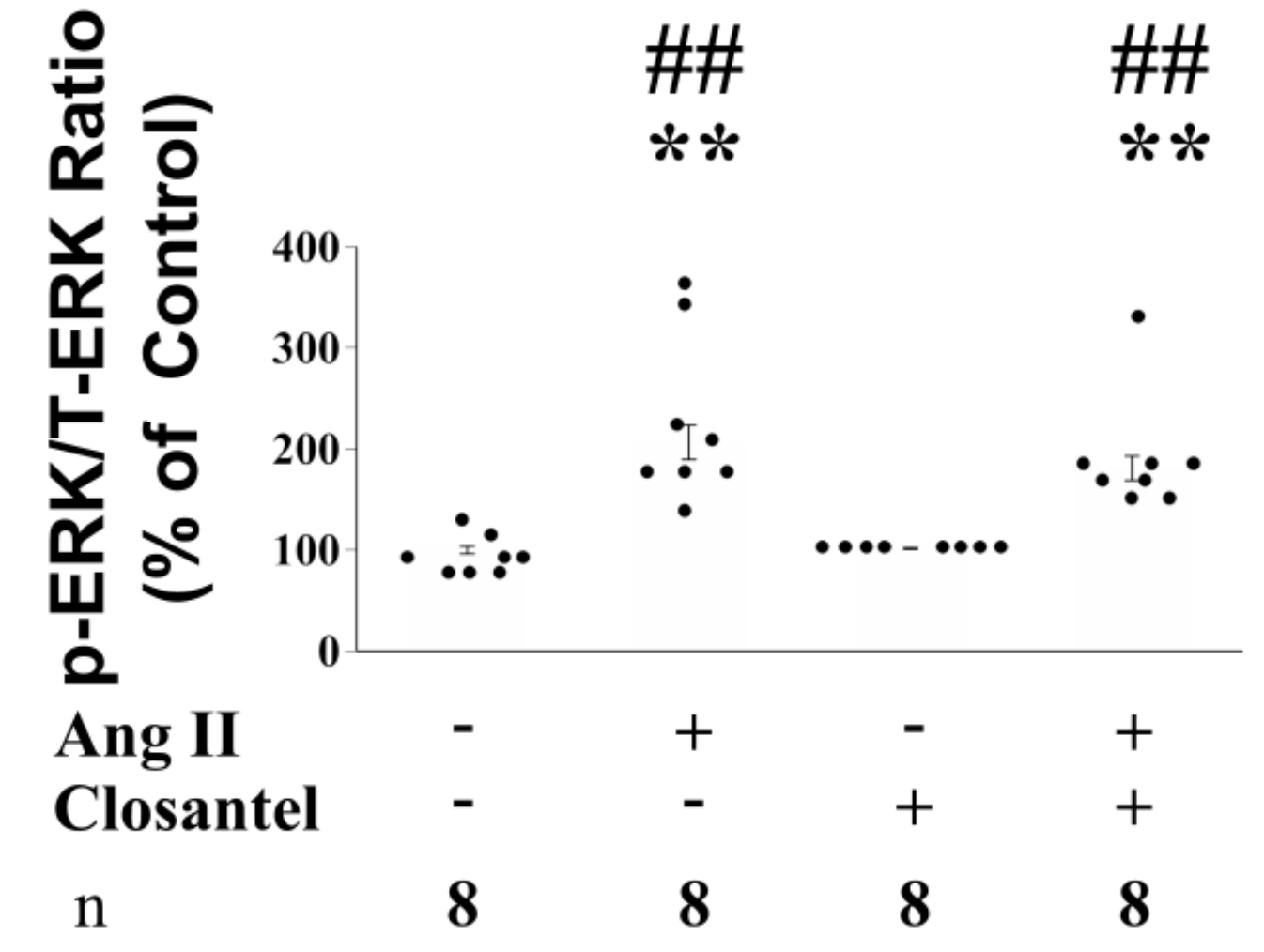
p-ERK/T-ERK Ratio
(% of Control)
##
**
##
**
400
300
200
100
0
Ang II - + - +
Closantel - - + +
n 8 8 8 8

I

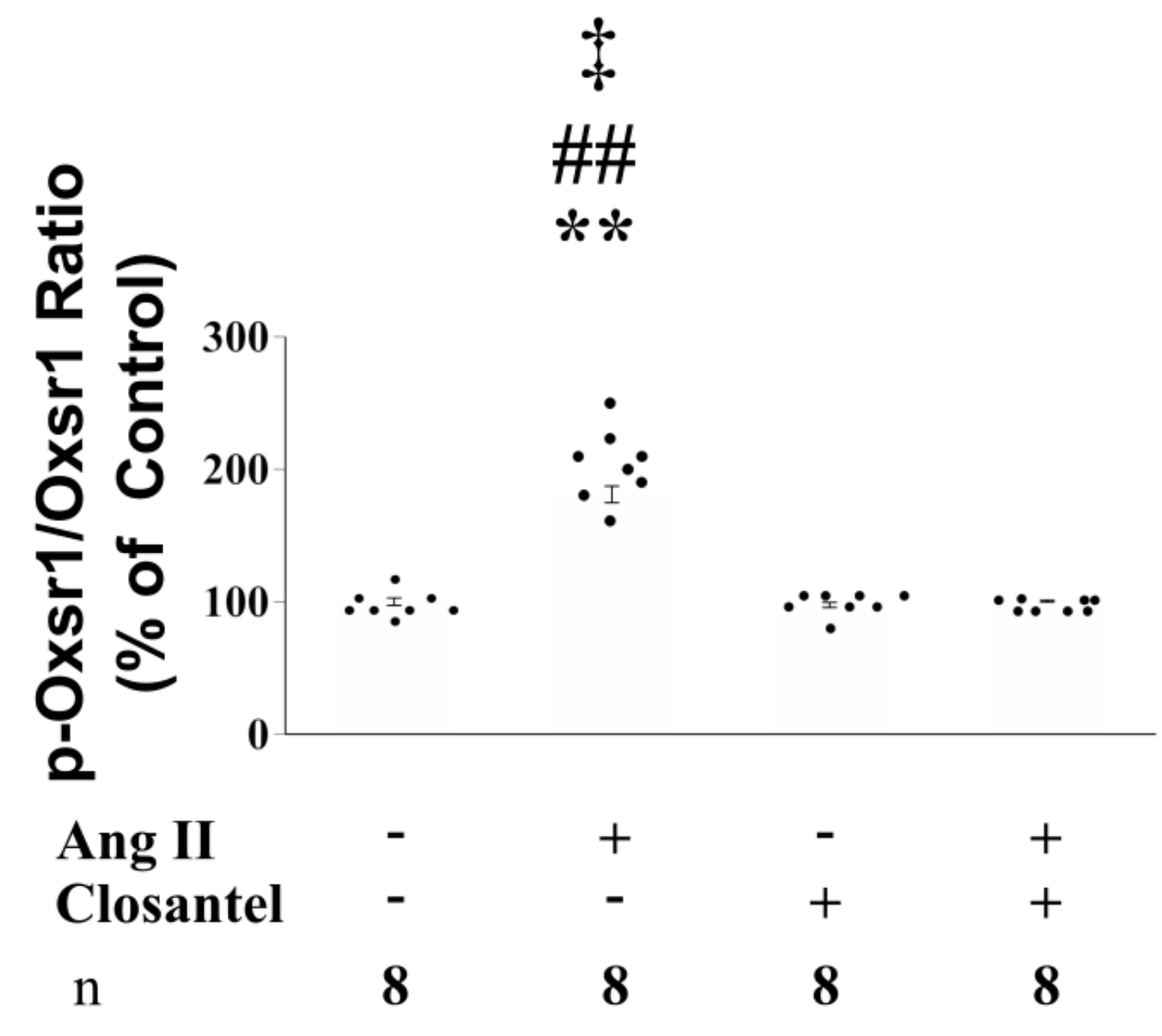
p-Oxsr1/Oxsr1 Ratio
(% of Control)
‡
##
**
300
200
100
0
Ang II - + - +
Closantel - - + +
n 8 8 8 8

J

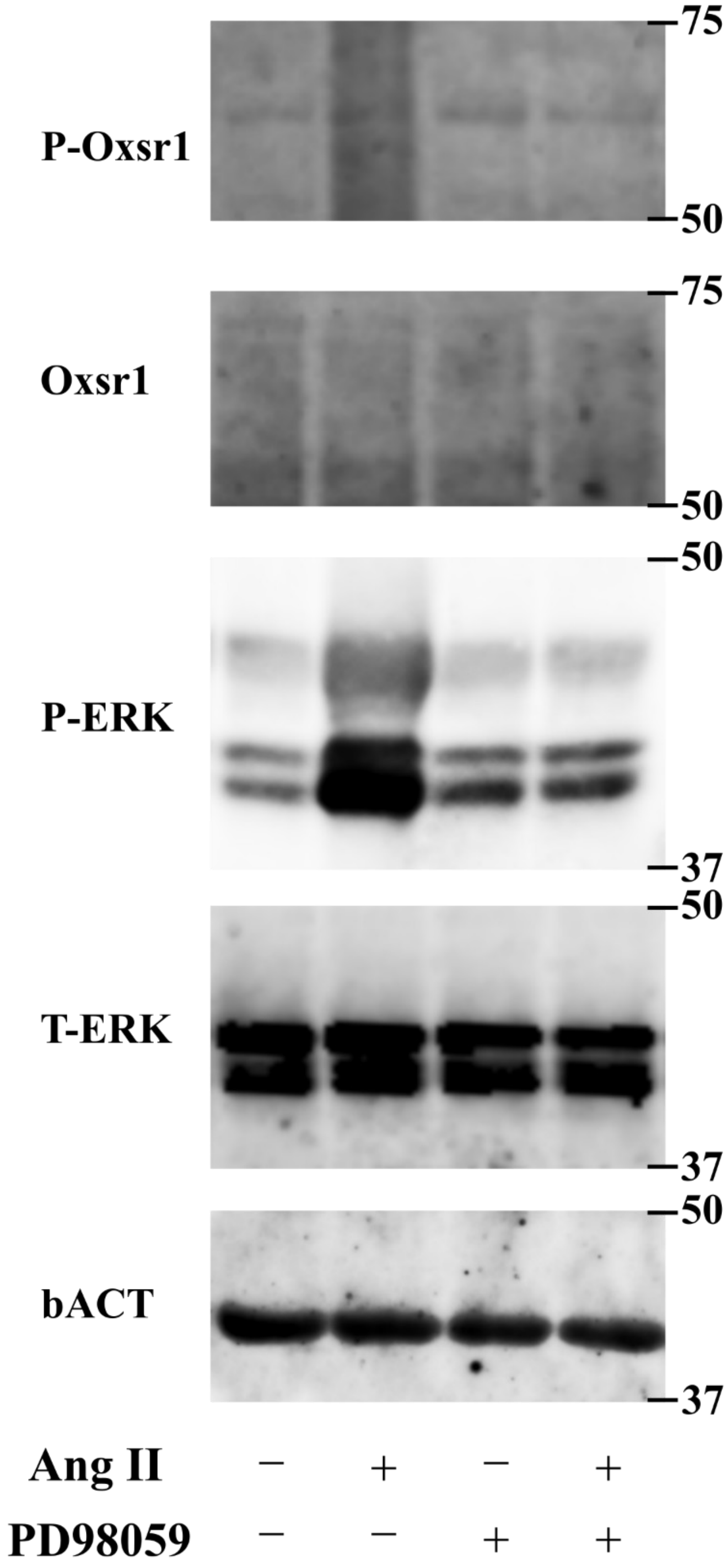
P-Oxsr1
Oxsr1
P-ERK
T-ERK
bACT
75
50
75
50
50
37
50
37
50
37
Ang II − + − +
PD98059 − − + +

K

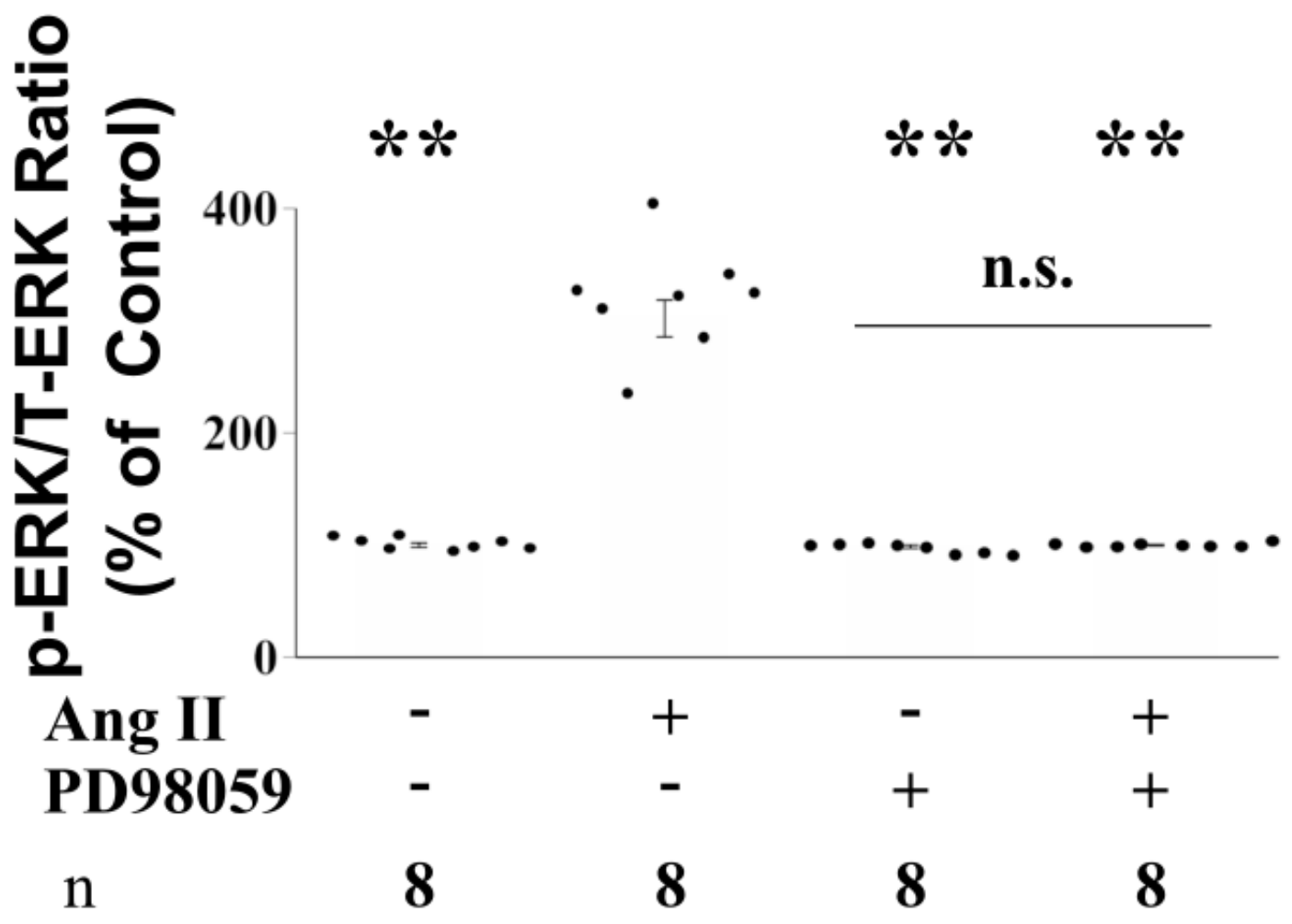

p-ERK/T-ERK Ratio
(% of Control)
**
**
**
n.s.
400
200
0
Ang II
PD98059
n
8
8
8
8

L

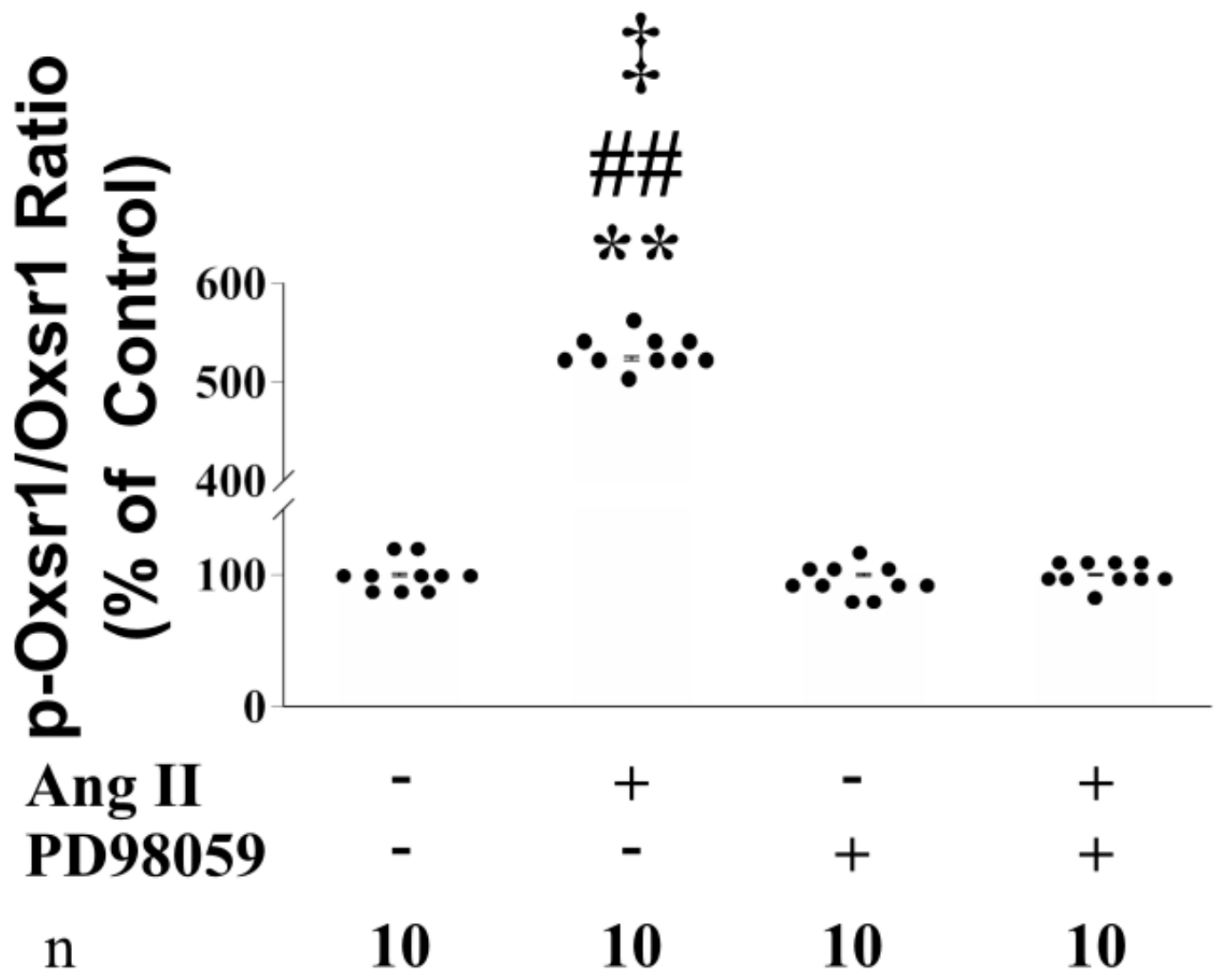

p-Oxsr1/Oxsr1 Ratio
(% of Control)
‡
##
**
600
500
400
100
0
Ang II
PD98059
n
10
10
10
10

M

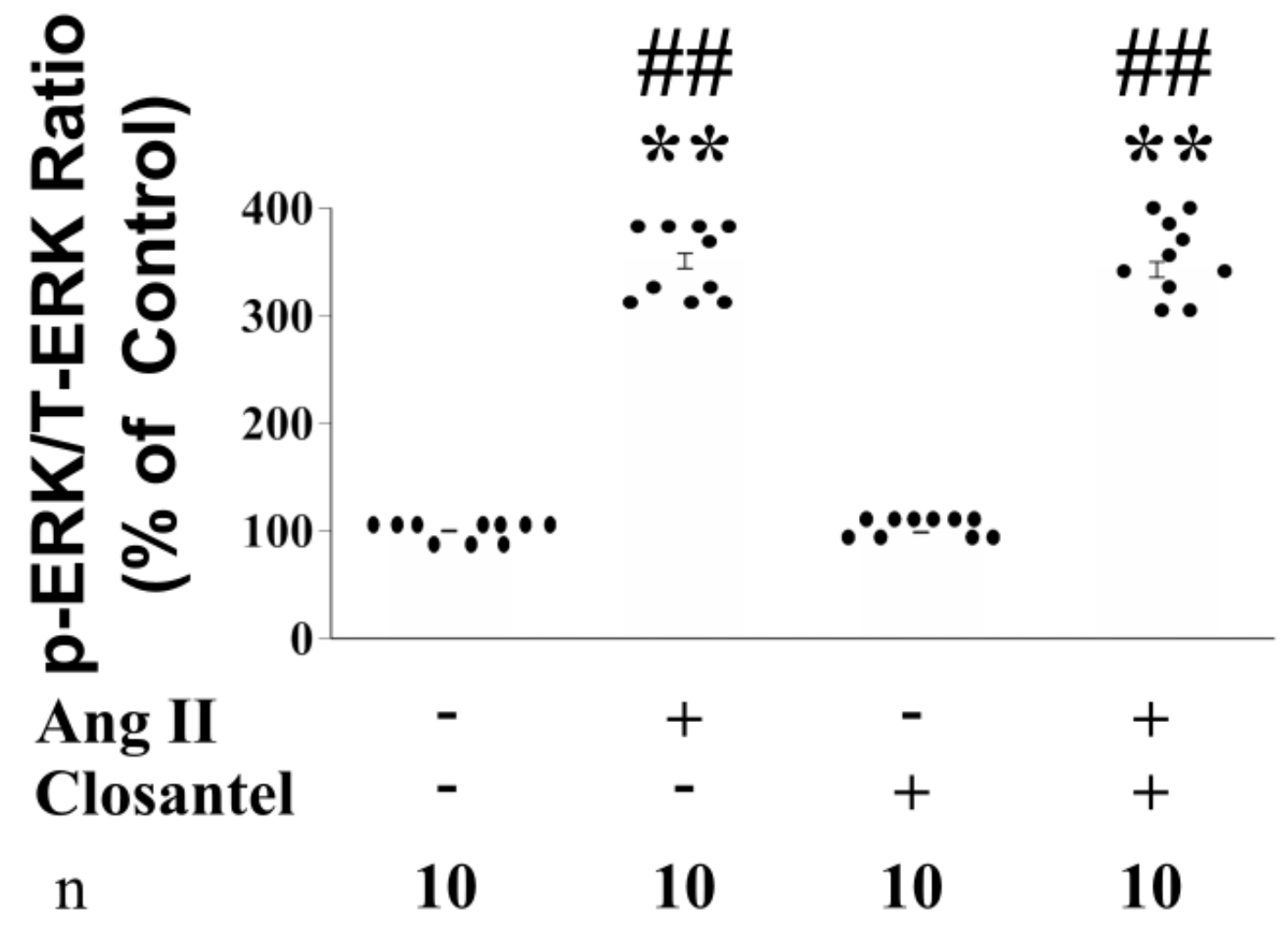

p-ERK/T-ERK Ratio
(% of Control)
##
**
##
**
400
300
200
100
0
Ang II - + - +
Closantel - - + +
n 10 10 10 10

N

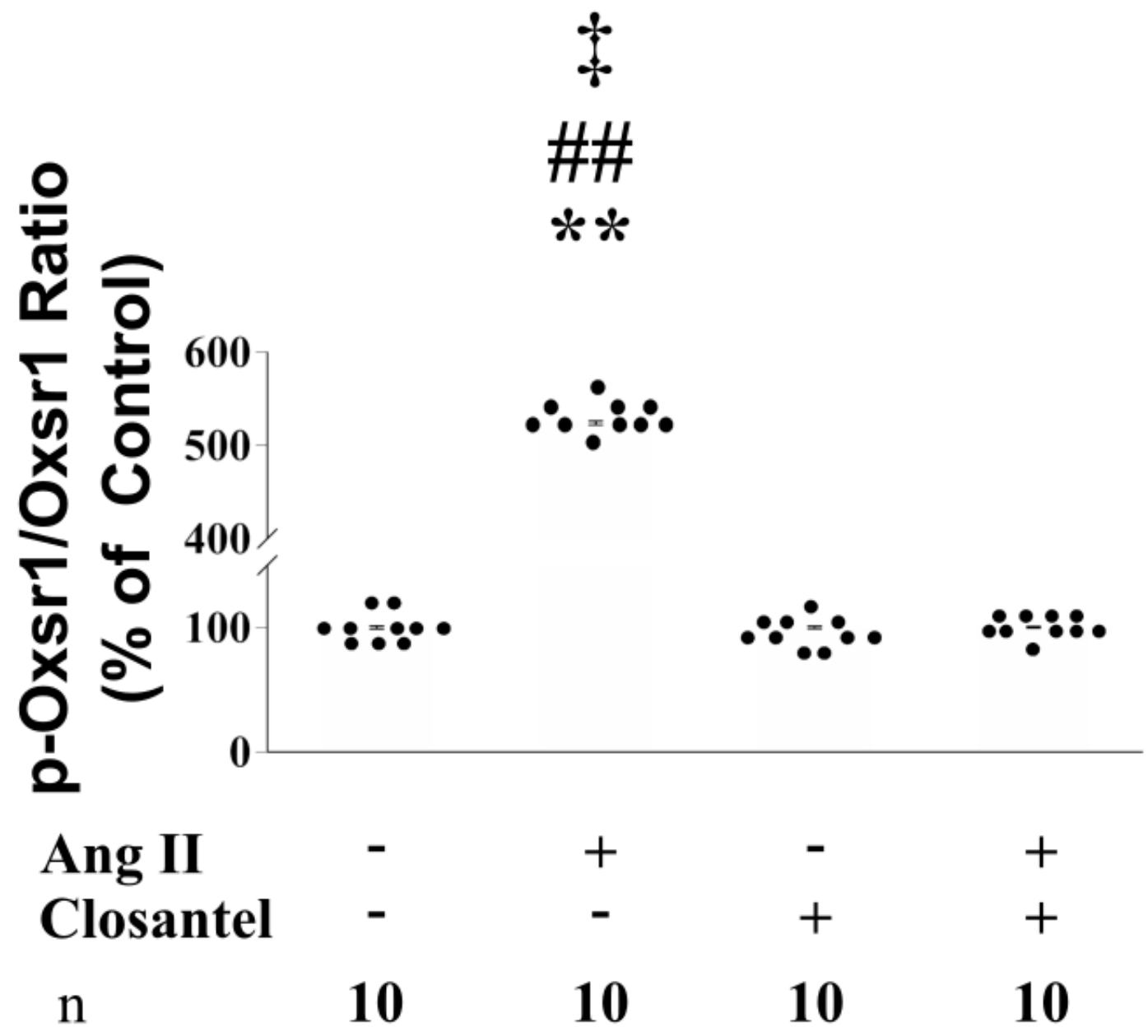

p-Oxsr1/Oxsr1 Ratio
(% of Control)
‡
##
**
600
500
400
100
0
Ang II - + - +
Closantel - - + +
n 10 10 10 10

Supplementary Figure 4. Effect of closantel or ERK1/2 inhibitor on pioglitazone (PGZ) or angiotensin II (AngII)-induced ERK and oxidative stress-responsive kinase 1 (OXSR1) phosphorylation

A) PGZ-induced ERK phosphorylation and the effect of closantel on ERK phosphorylation in rat renal cortex (*n* = 8). **p<0.01 versus untreated proximal tubules (PTs), ##p<0.01 versus closantel-treated PTs.

B) PGZ-induced OXSR1 phosphorylation and the effect of closantel on OXSR1 phosphorylation in rat renal cortex (*n* = 8). **p<0.01 versus untreated PTs, ##p<0.01 versus closantel-treated PTs, ‡ p<0.01 versus closantel and PGZ-treated PTs.

C) Effects of the ERK inhibitor PD98059 on PGZ-induced ERK and OXSR1 phosphorylation in rat renal cortex tissue.

D) Effect of PD98059 on PGZ-induced ERK phosphorylation in rat renal cortex tissue (*n* = 8). **p<0.01 versus PGZ-treated PTs.

E) Effect of PD98059 on PGZ-induced OXSR1 phosphorylation in rat renal cortex tissue (*n* = 8). **p<0.01 versus PGZ-treated PTs.

F) PGZ-induced ERK phosphorylation and the effect of closantel on ERK phosphorylation in human renal cortex (*n* = 10). **p<0.01 versus untreated PTs, ##p<0.01 versus closantel-treated PTs.

G) PGZ-induced OXSR1 phosphorylation and the effect of closantel on OXSR1 phosphorylation in human renal cortex (*n* = 10). **p<0.01 versus untreated PTs, ##p<0.01 versus closantel-treated PTs, ‡ p<0.01 versus closantel and PGZ-treated PTs.

H) Angiotensin II (AngII)-induced ERK phosphorylation and its effect on ERK phosphorylation in rat renal cortex (*n* = 8). **p<0.01 versus untreated PTs, ##p<0.01 versus closantel-treated PTs.

I) Ang II-induced OXSR1 phosphorylation and the effect of closantel on OXSR1 phosphorylation in rat renal cortex (*n* = 8). **p<0.01 versus untreated PTs, ##p<0.01 versus closantel-treated PTs, ‡ p<0.01 versus closantel and AngII-treated PTs.

J) Effects of PD98059 on AngII-induced ERK and OXSR1 phosphorylation in rat renal cortex tissue.

K) Effect of PD98059 on AngII-induced ERK phosphorylation in rat renal cortex tissue (*n* = 8). **p<0.01 versus AngII-treated PTs.

L) Effect of PD98059 on AngII-induced OXSR1 phosphorylation in rat renal cortex tissue (*n* = 10). **p<0.01 versus AngII-treated PTs.

M) Ang II-induced ERK phosphorylation and its effect on ERK phosphorylation in human renal cortex (*n* = 10). **p<0.01 versus untreated PTs, ##p<0.01 versus closantel-treated PTs.

N) Ang II-induced OXSR1 phosphorylation and the effect of closantel on OXSR1 phosphorylation in human renal cortex (*n* = 10). **p<0.01 versus untreated PTs, ##p<0.01 versus closantel-treated PTs, ‡ p<0.01 versus closantel and angiotensin II-treated PTs.

Data are presented as means ± standard errors.

A

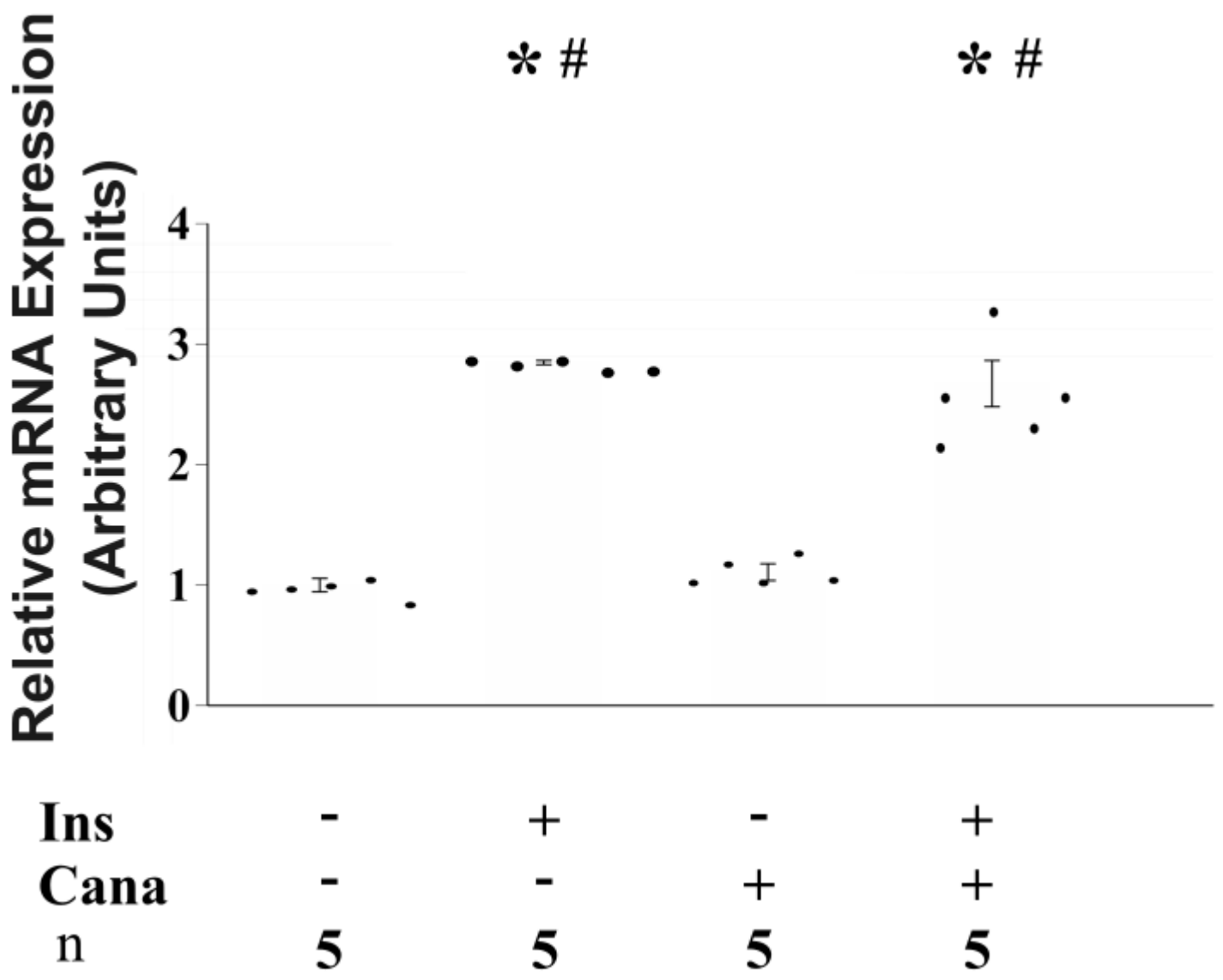
* #
* #
Relative mRNA Expression
(Arbitrary Units)
4
3
2
1
0
Ins - + - +
Cana - - + +
n 5 5 5 5

B

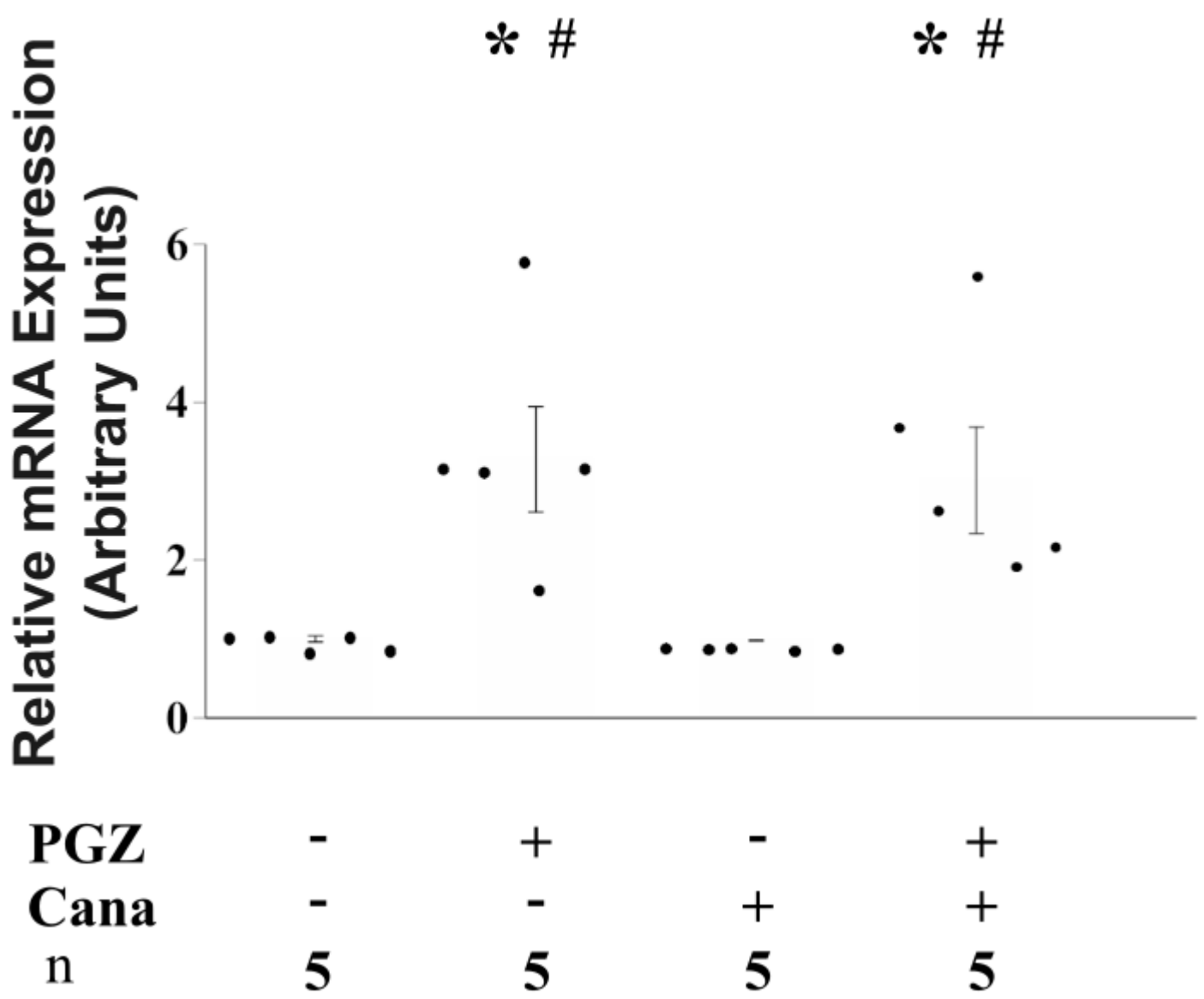
* #
* #
Relative mRNA Expression
(Arbitrary Units)
6
4
2
0
PGZ - + - +
Cana - - + +
n 5 5 5 5

C

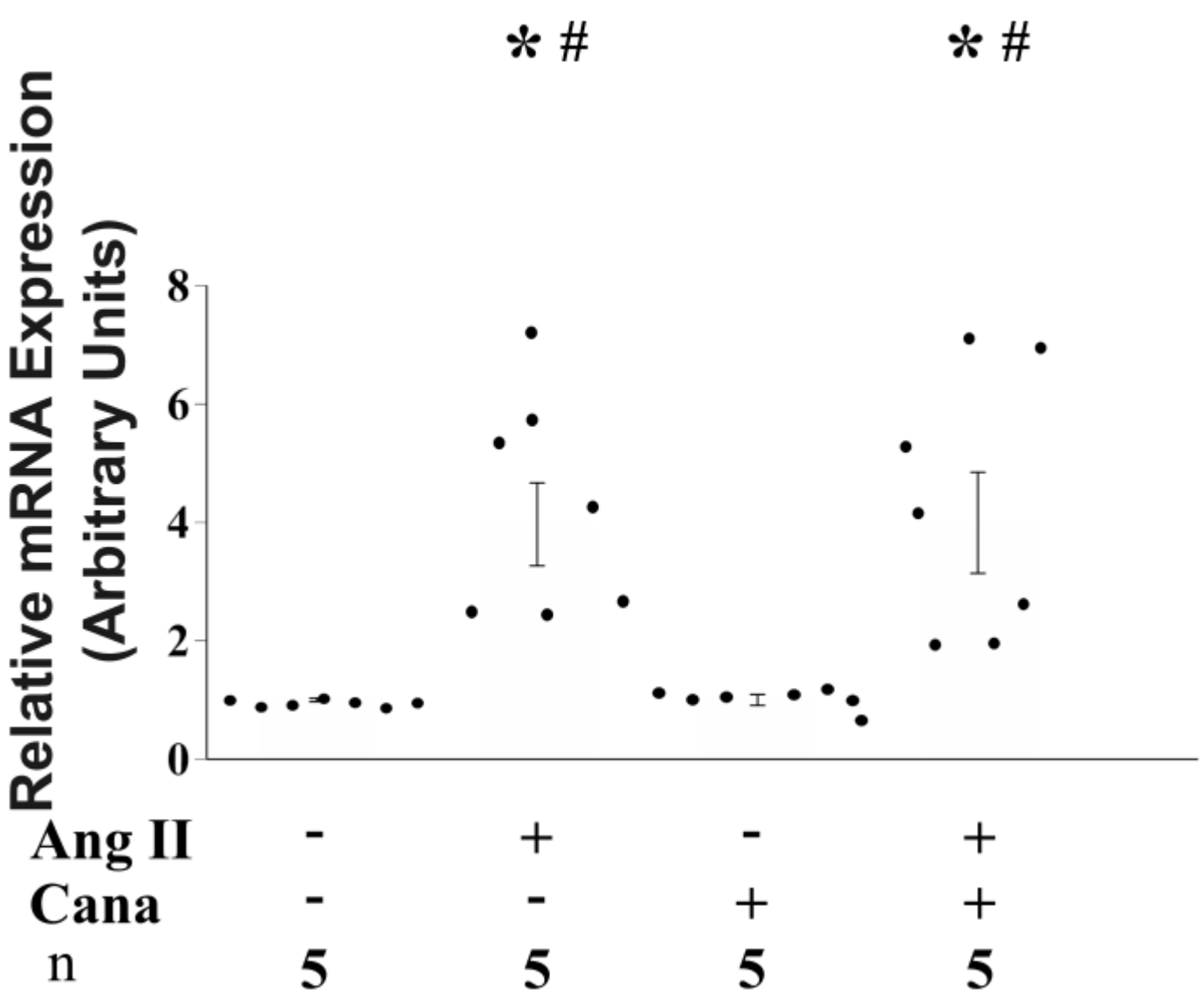
* #
* #
Relative mRNA Expression
(Arbitrary Units)
8
6
4
2
0
Ang II - + - +
Cana - - + +
n 5 5 5 5

D

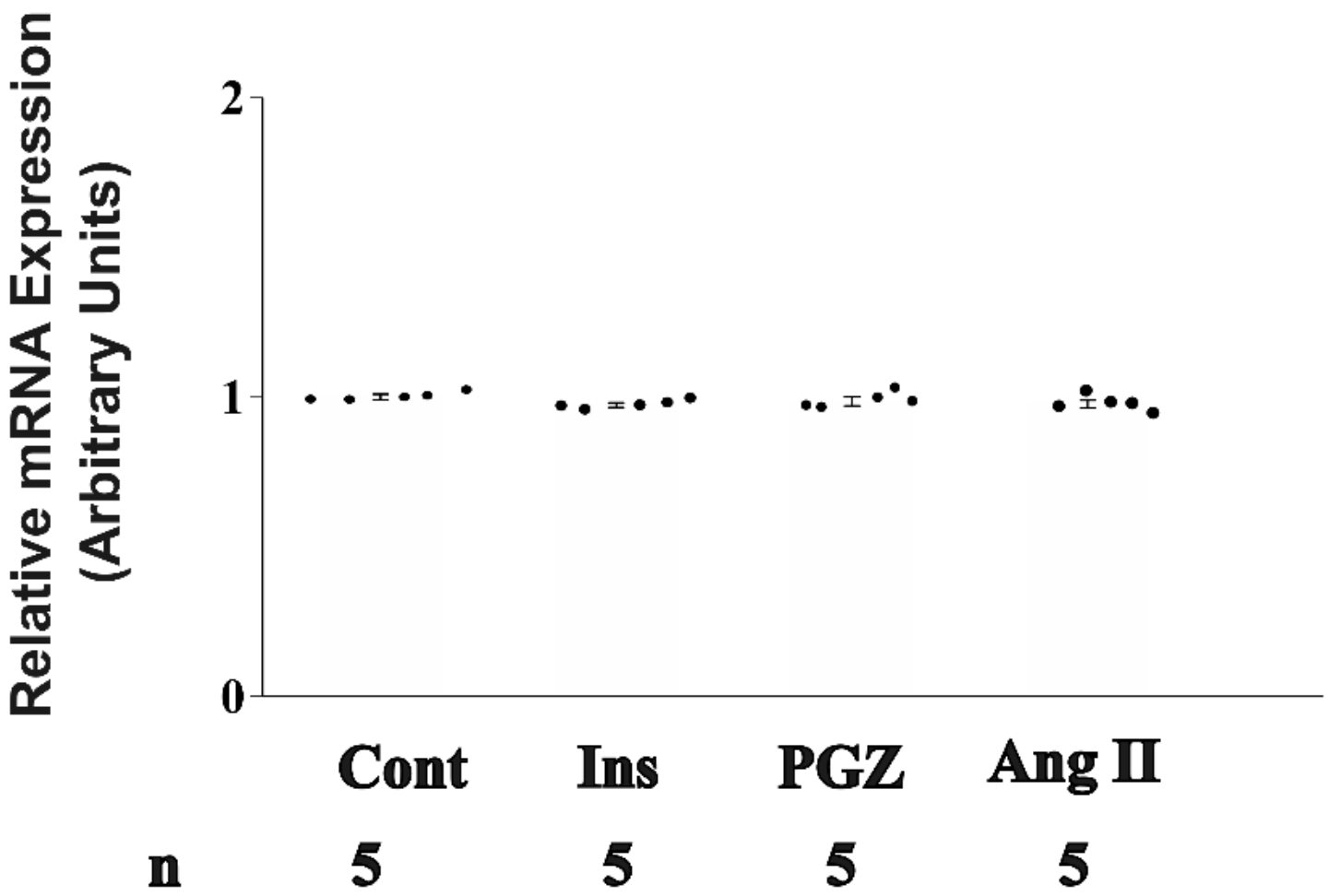
Relative mRNA Expression
(Arbitrary Units)
2
1
0
Cont
Ins
PGZ
Ang II
n 5 5 5 5

E

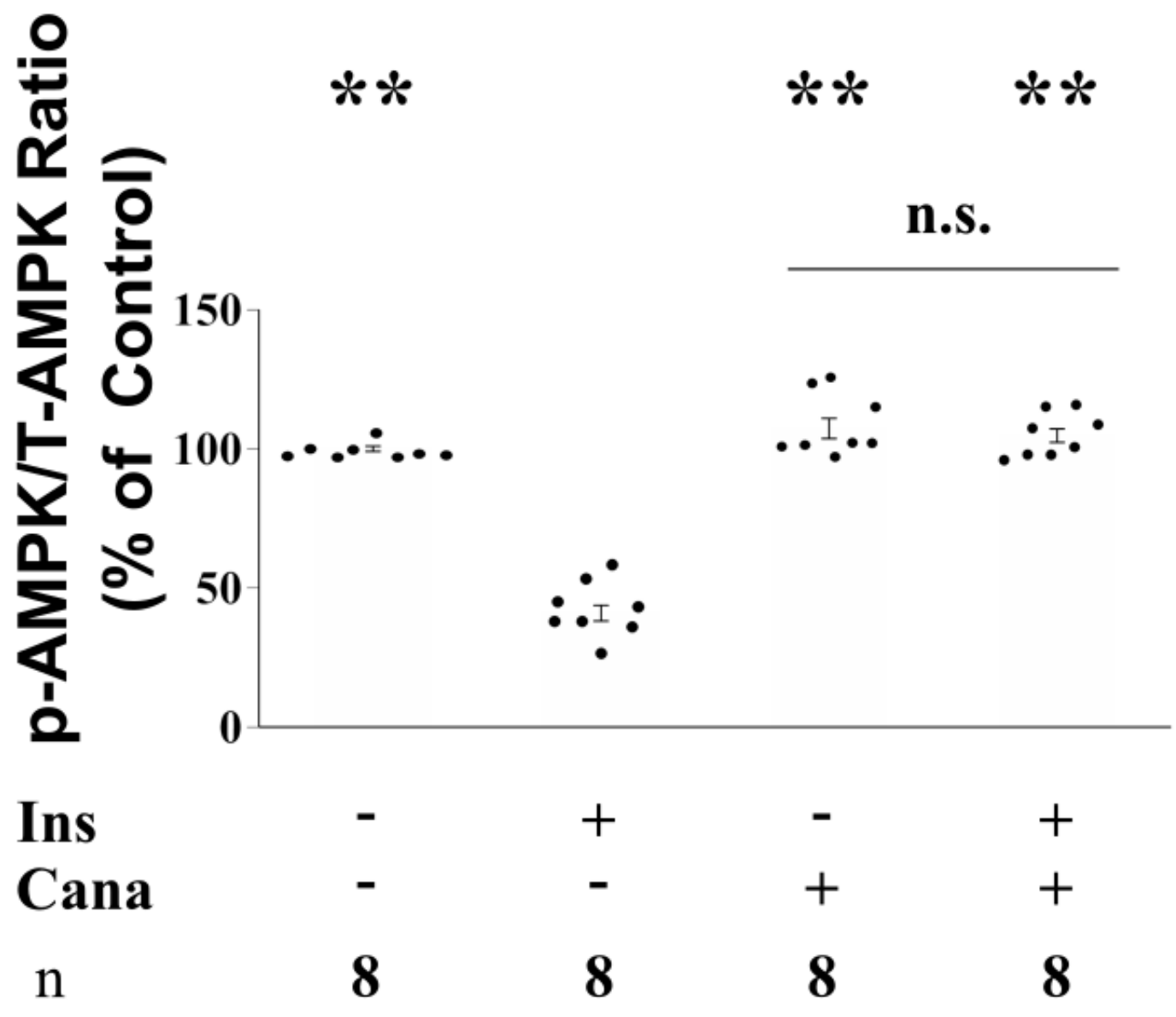
p-AMPK/T-AMPK Ratio
(% of Control)
**
**
**
n.s.
150
100
50
0
Ins - + - +
Cana - - + +
n 8 8 8 8

F

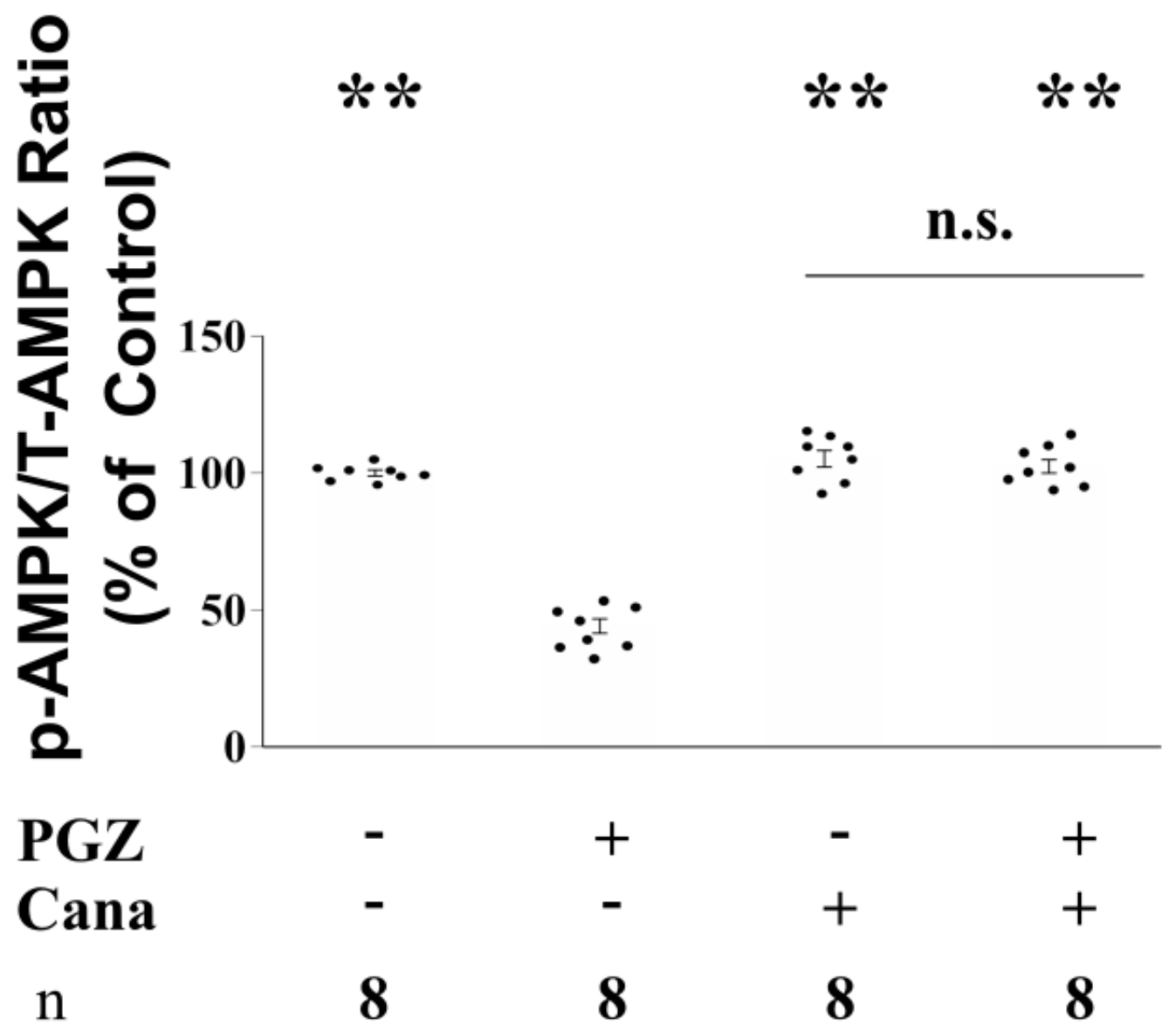
p-AMPK/T-AMPK Ratio
(% of Control)
**
**
**
n.s.
150
100
50
0
PGZ - + - +
Cana - - + +
n 8 8 8 8

G

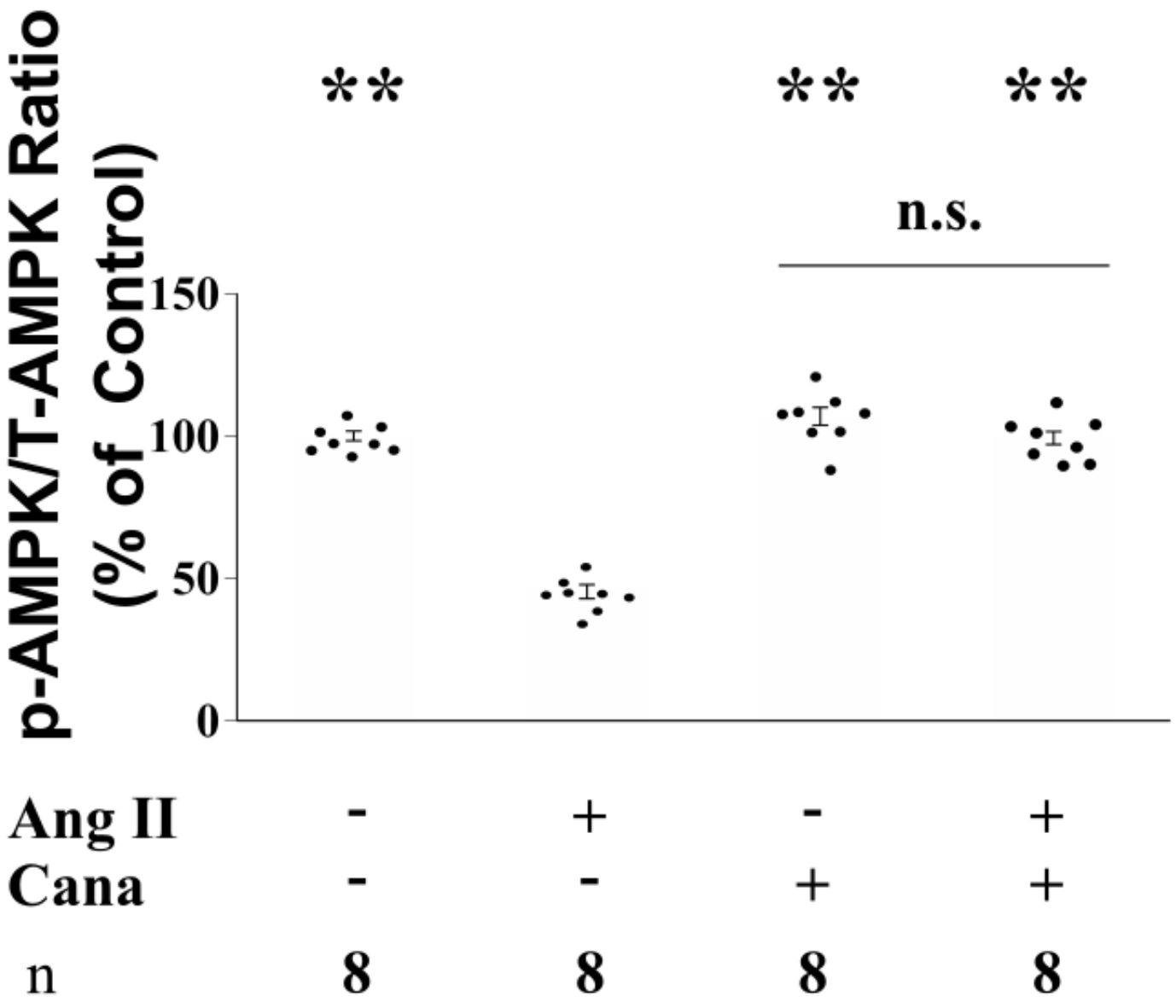

p-AMPK/T-AMPK Ratio
(% of Control)
150
100
50
0
**
**
**
n.s.
Ang II - + - +
Cana - - + +
n 8 8 8 8

H

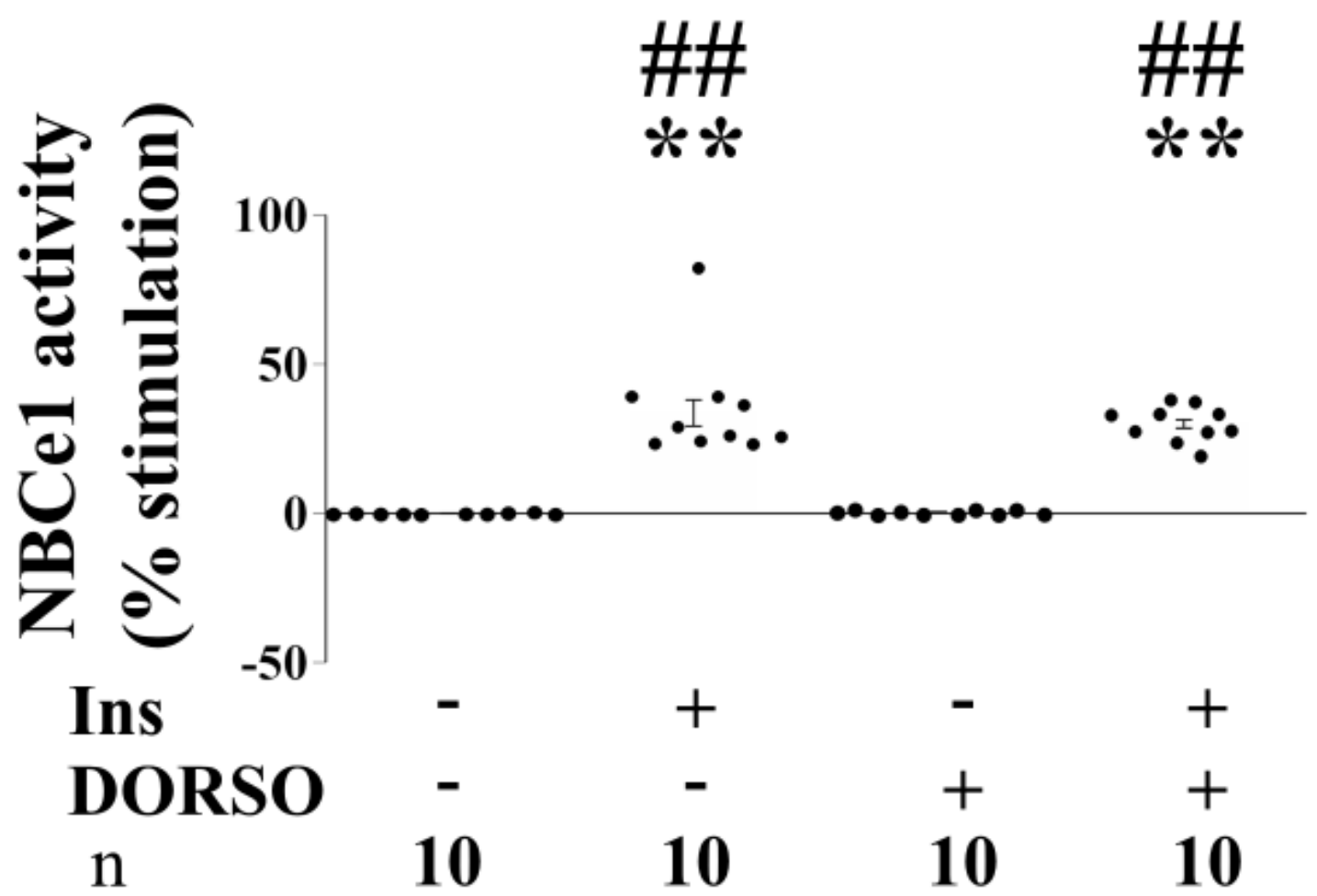

NBCe1 activity
(% stimulation)
100
50
0
-50
##
**
##
**
Ins - + - +
DORSO - - + +
n 10 10 10 10

I

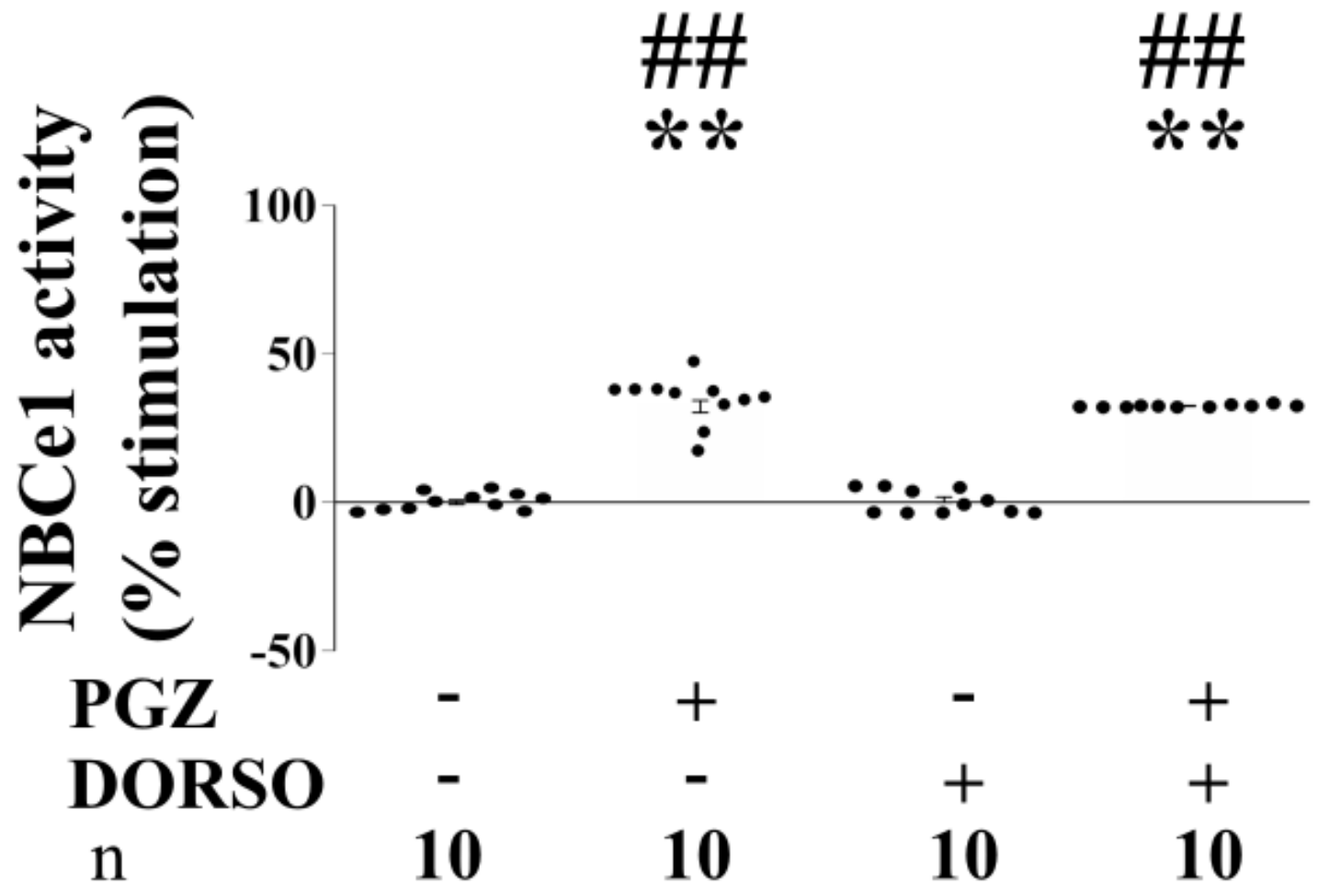

##
**
##
**
NBCe1 activity
(% stimulation)
100
50
0
-50
PGZ - + - +
DORSO - - + +
n 10 10 10 10

J

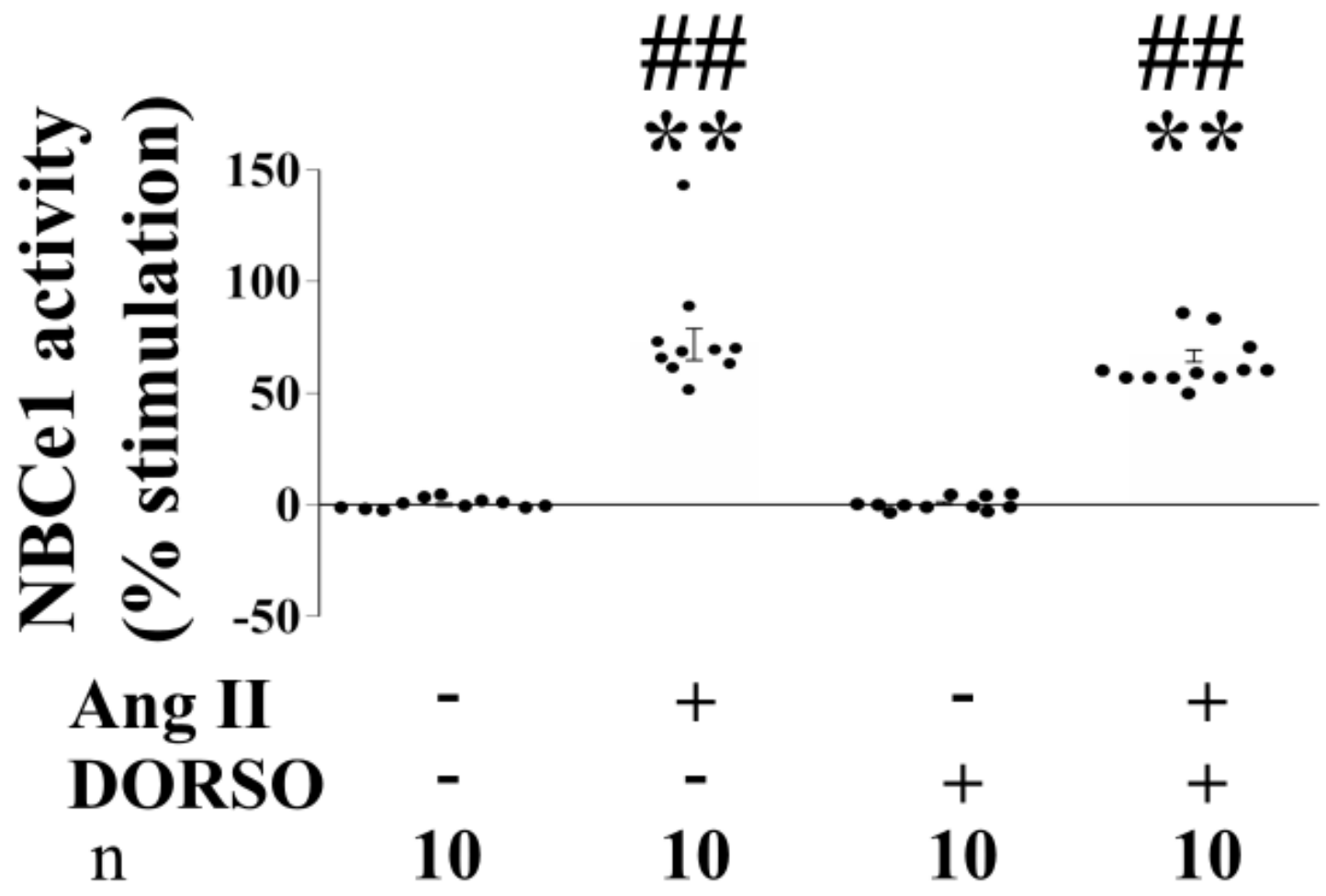

##
**
##
**
NBCe1 activity
(% stimulation)
150
100
50
0
-50
Ang II - + - +
DORSO - - + +
n 10 10 10 10

K

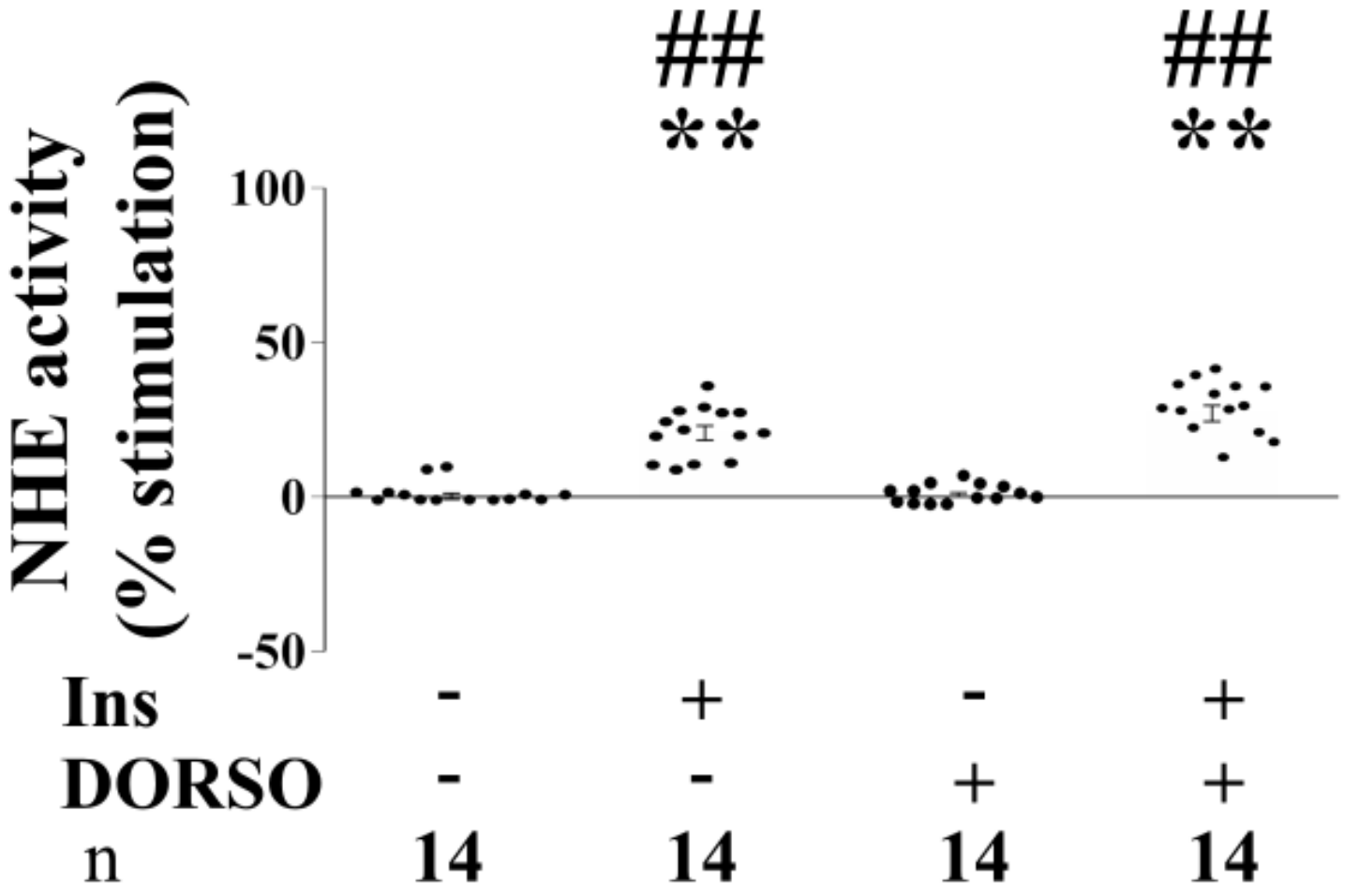

##
**
##
**
NHE activity
(% stimulation)
100
50
0
-50
Ins - + - +
DORSO - - + +
n 14 14 14 14

L

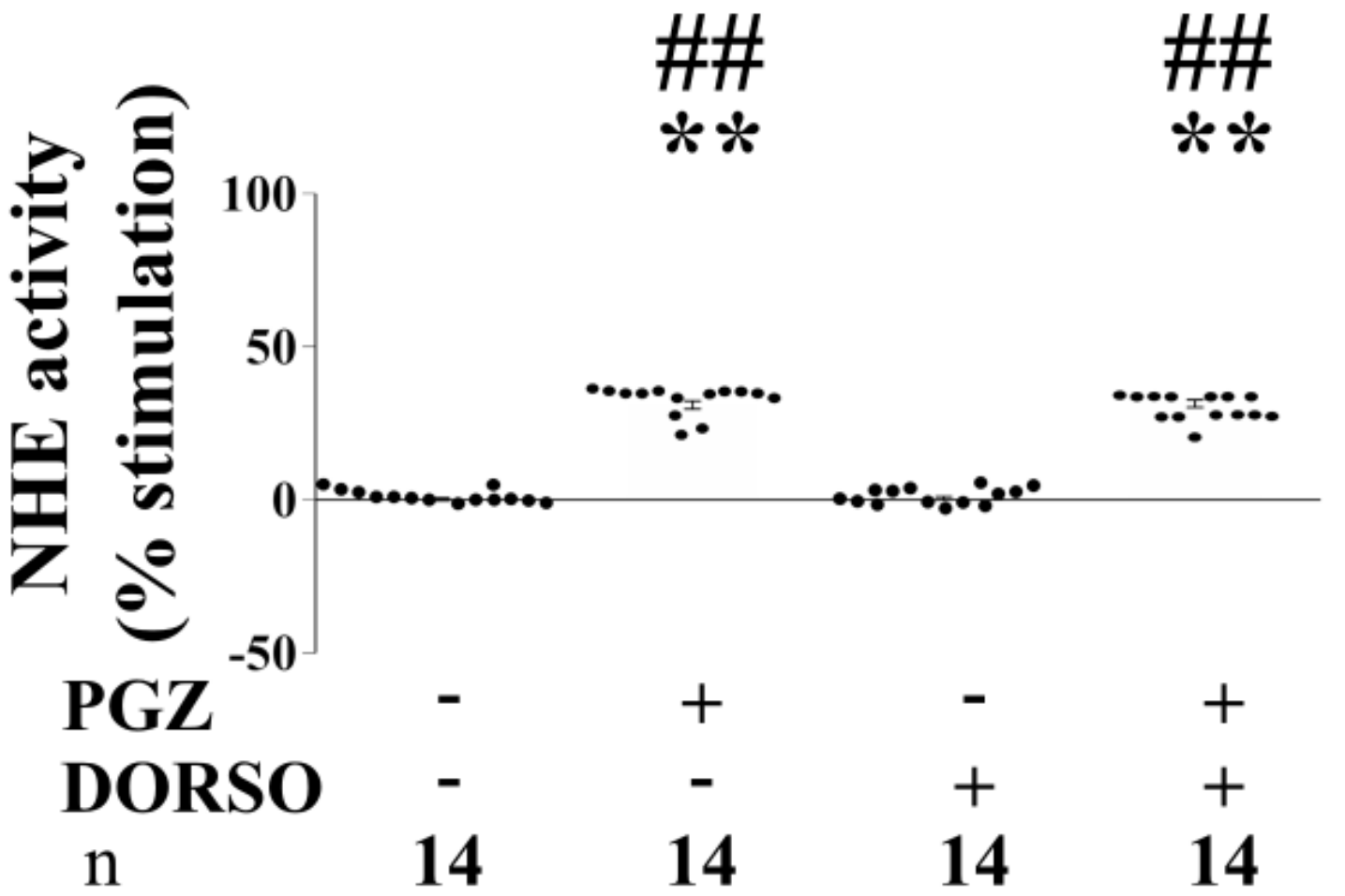

##
**
##
**
NHE activity
(% stimulation)
100
50
0
-50
PGZ - + - +
DORSO - - + +
n 14 14 14 14

M

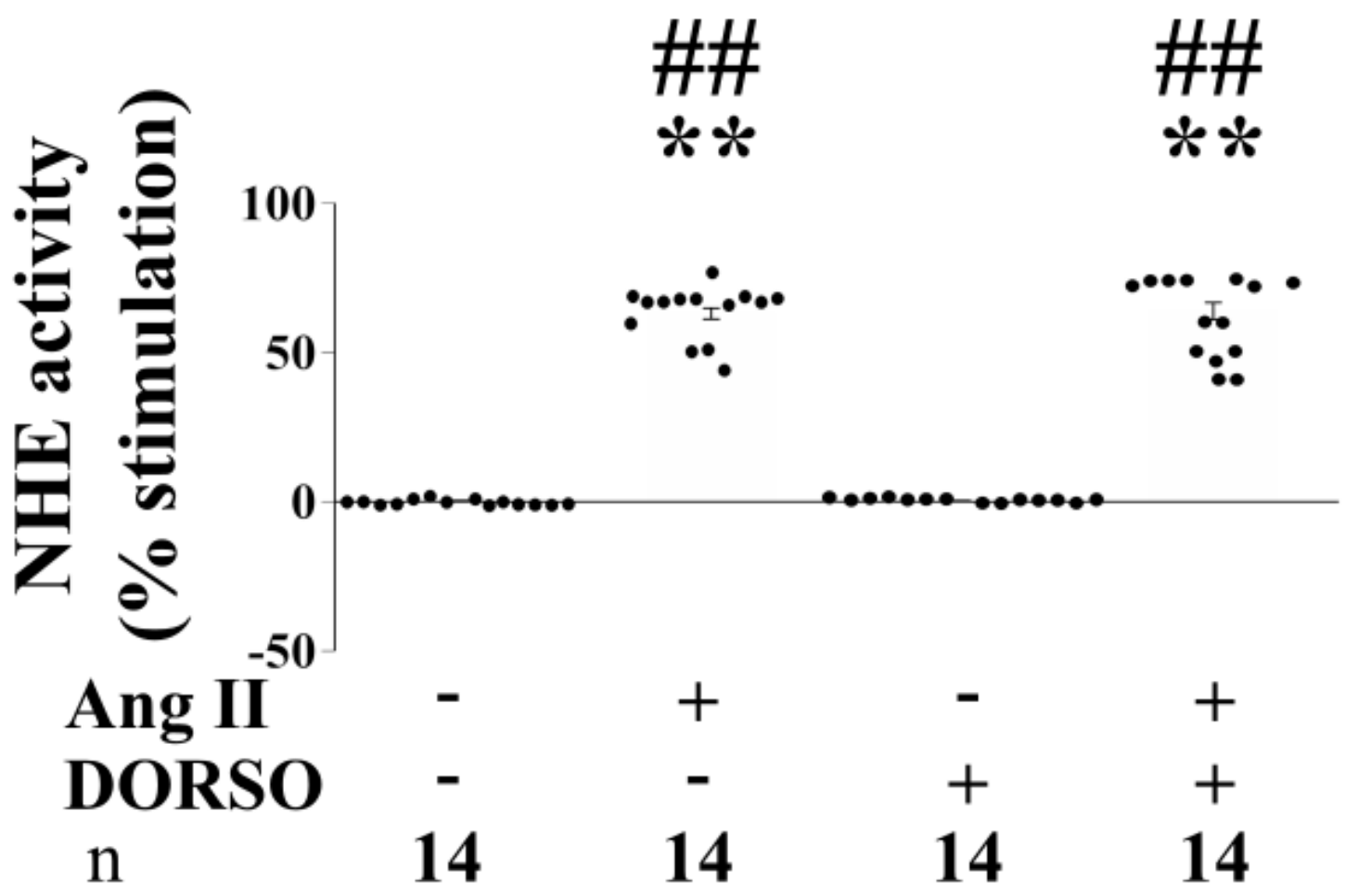

##
**
##
**
NHE activity
(% stimulation)
100
50
0
-50
Ang II - + - +
DORSO - - + +
n 14 14 14 14

N

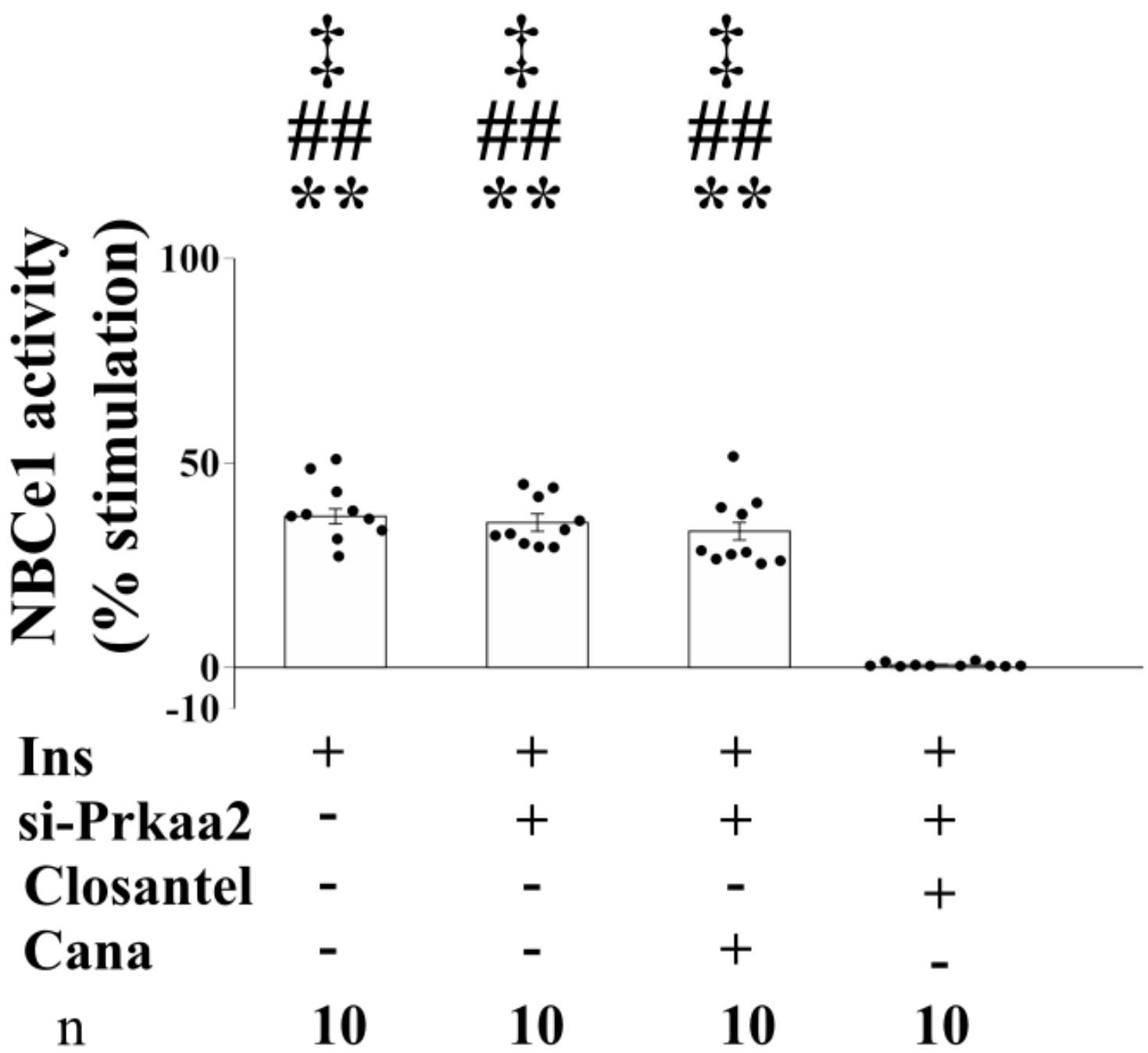

‡
##
**
‡
##
**
‡
##
**
NBCe1 activity
(% stimulation)
100
50
0
-10
Ins + + + +
si-Prkaa2 - + + +
Closantel - - - +
Cana - - + -
n 10 10 10 10

O

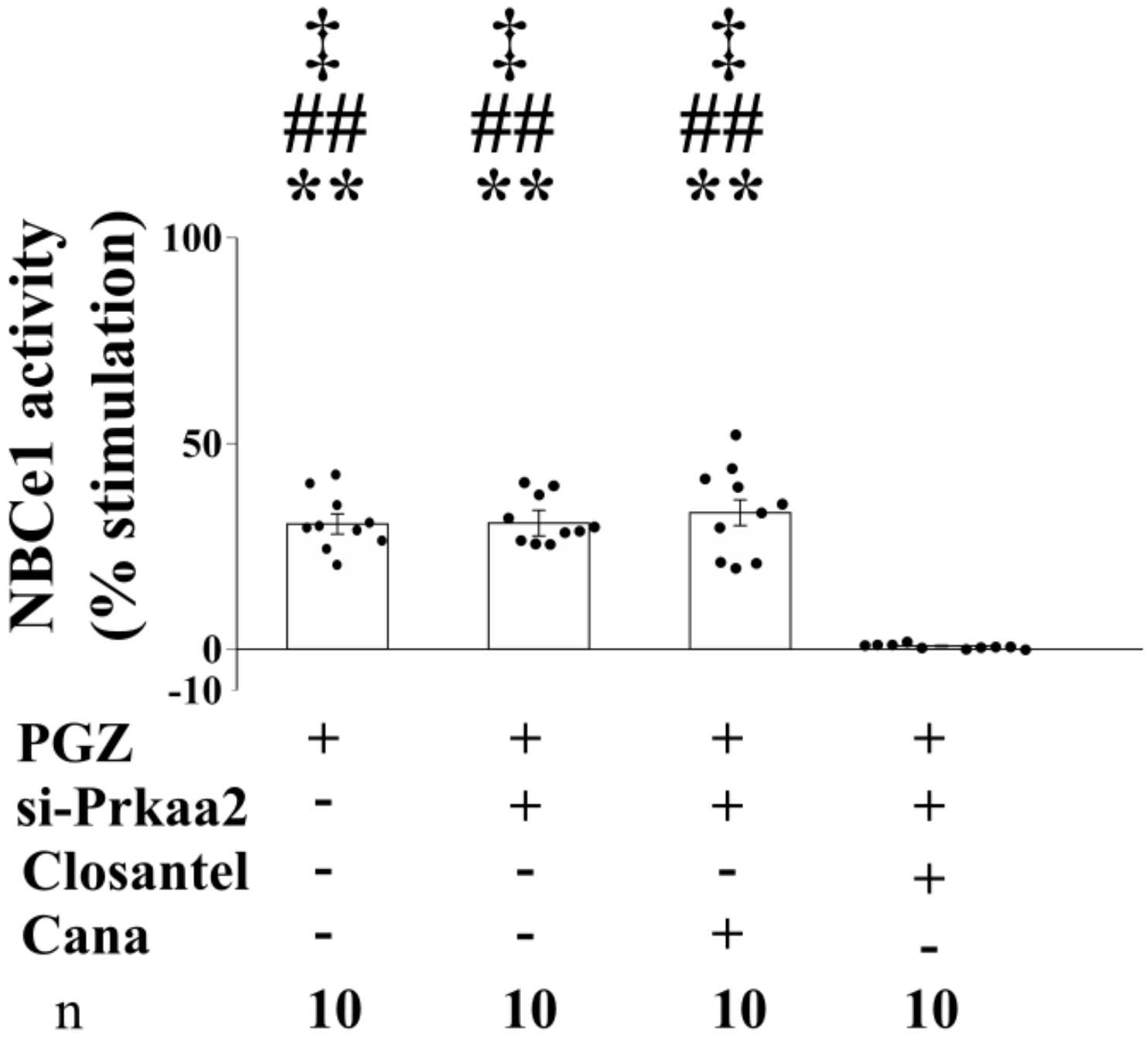
‡
##
**
NBCe1 activity
(% stimulation)
100
50
0
-10
PGZ + + + +
si-Prkaa2 - + + +
Closantel - - - +
Cana - - + -
n 10 10 10 10

P

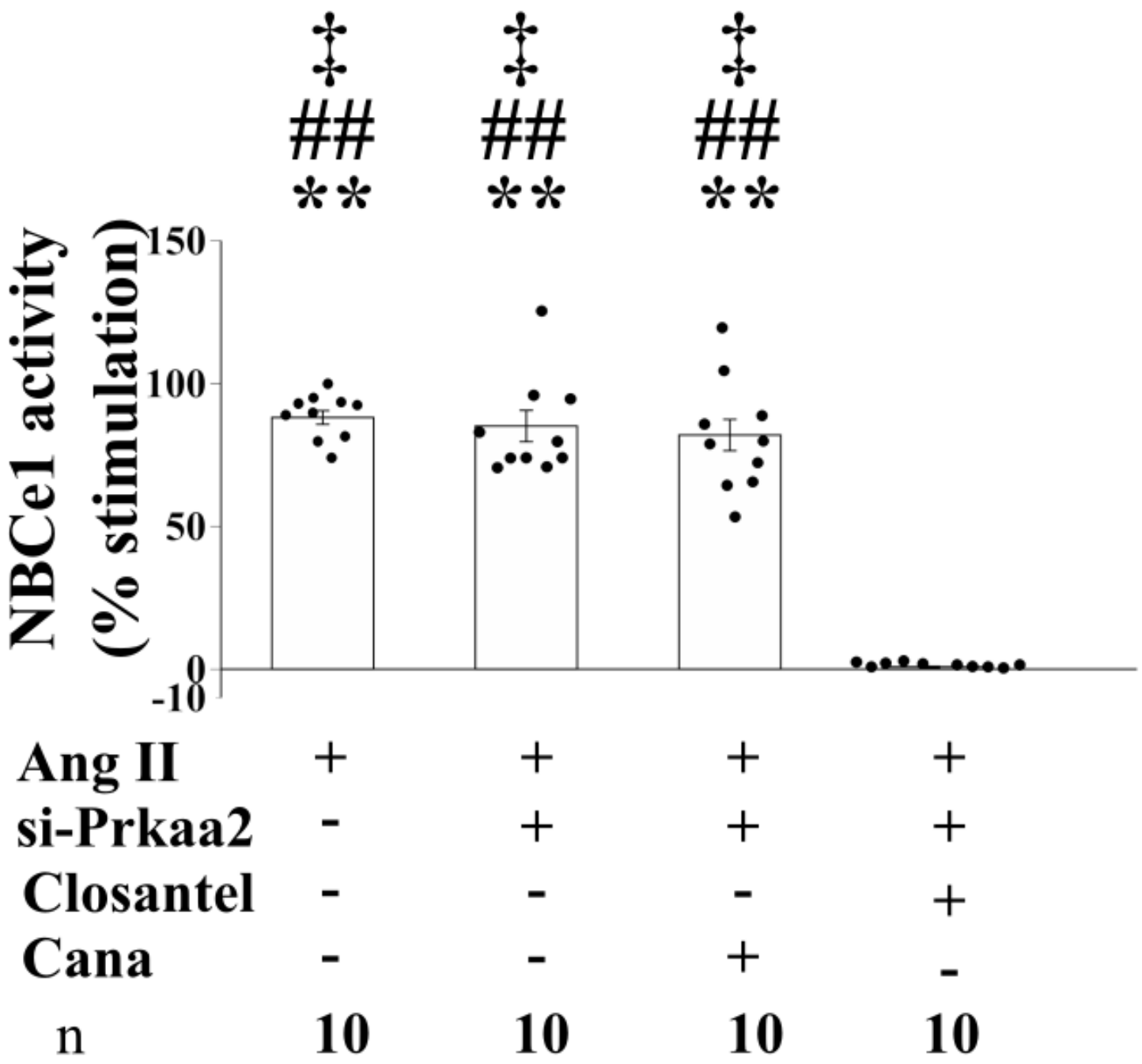
‡
##
**
NBCe1 activity
(% stimulation)
150
100
50
0
-10
Ang II + + + +
si-Prkaa2 - + + +
Closantel - - - +
Cana - - + -
n 10 10 10 10

Q

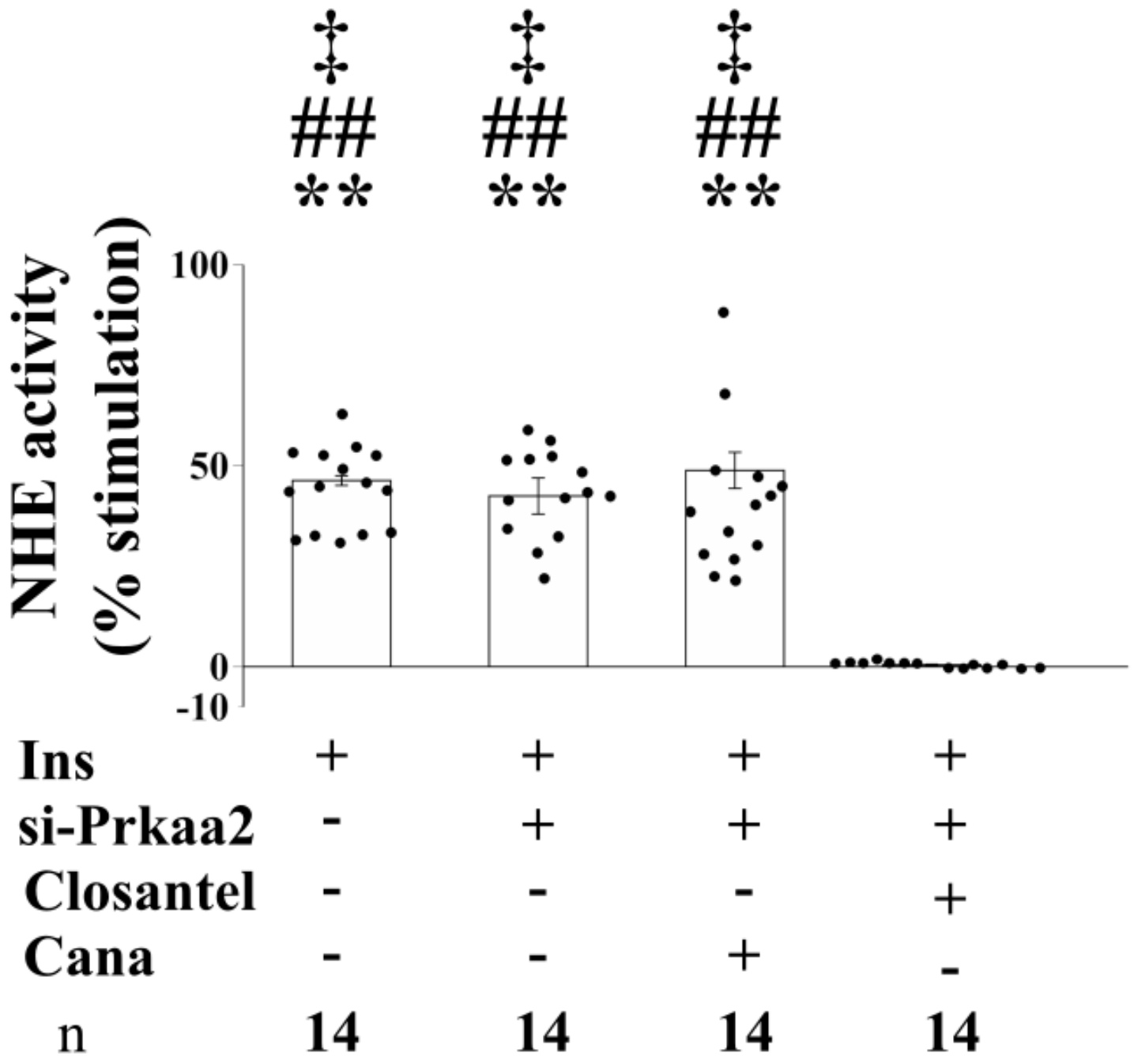
‡
##
**
NHE activity
(% stimulation)
100
50
0
-10
Ins + + + +
si-Prkaa2 - + + +
Closantel - - - +
Cana - - + -
n 14 14 14 14

R

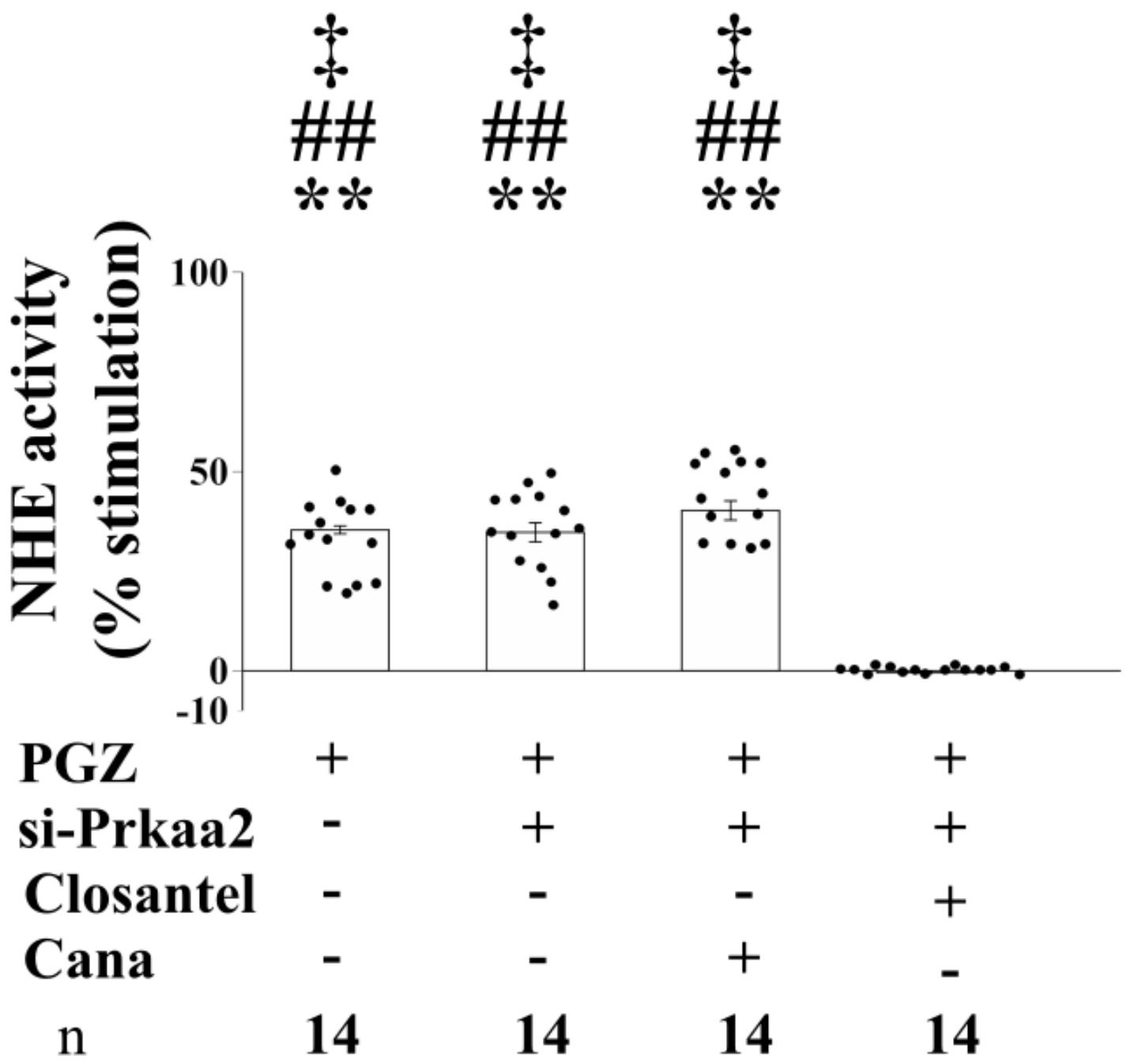
‡
##
**
NHE activity
(% stimulation)
100
50
0
-10
PGZ + + + +
si-Prkaa2 - + + +
Closantel - - - +
Cana - - + -
n 14 14 14 14

S

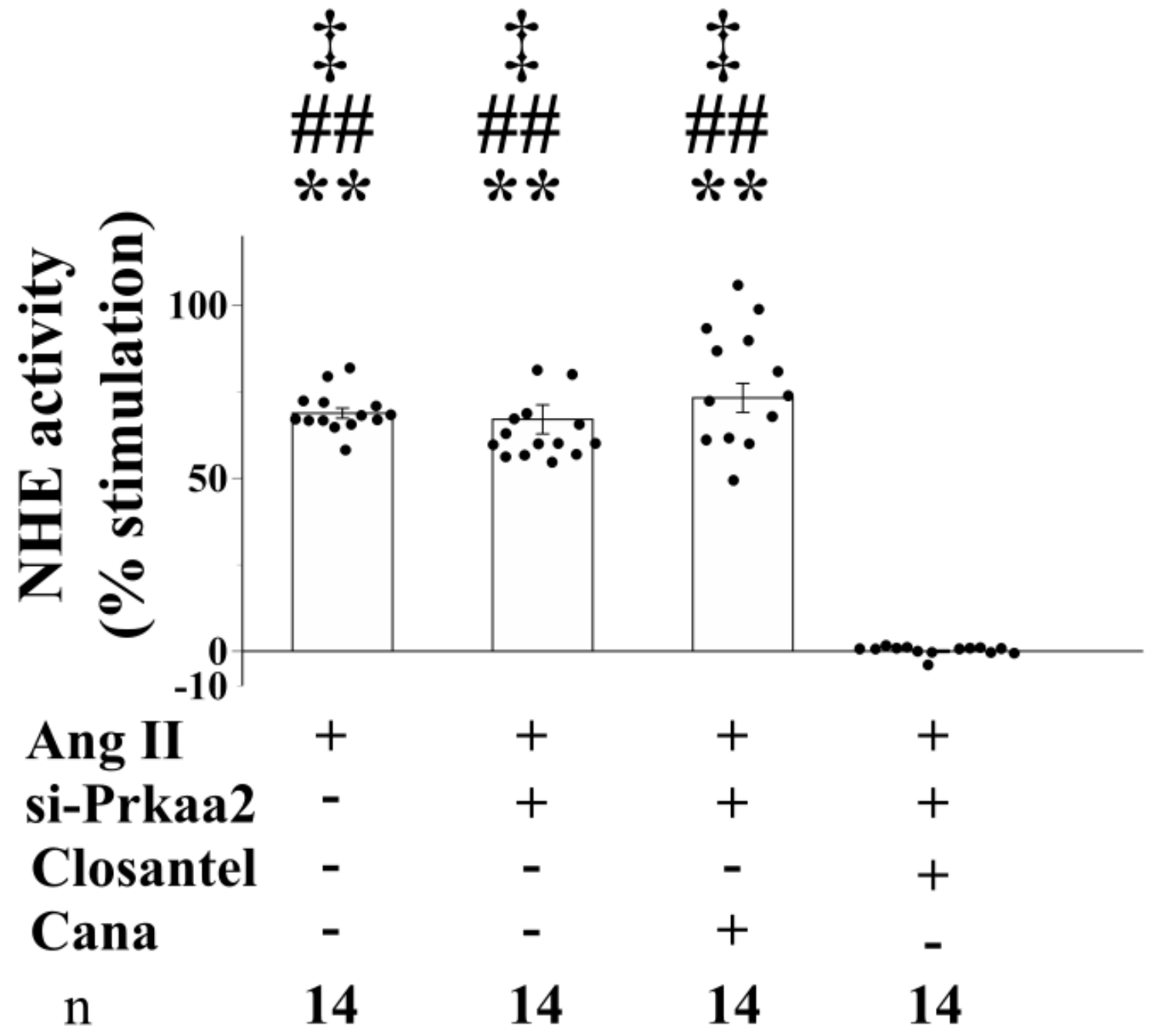

‡
##
**
‡
##
**
‡
##
**
NHE activity
(% stimulation)
100
50
0
-10
Ang II + + + +
si-Prkaa2 - + + +
Closantel - - - +
Cana - - + -
n 14 14 14 14

T

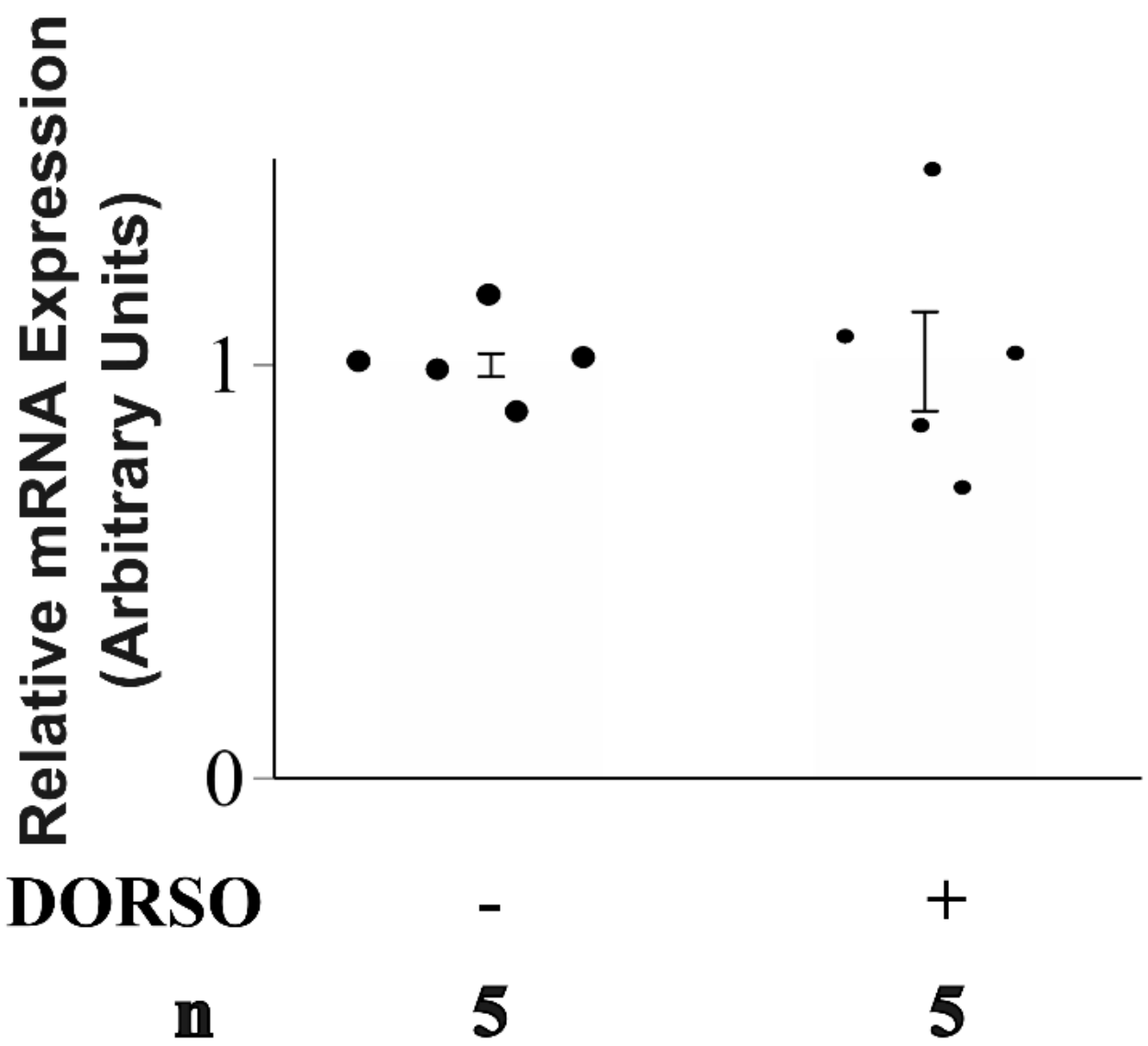
Relative mRNA Expression
(Arbitrary Units)
1
0
DORSO
-
+
n
5
5

U

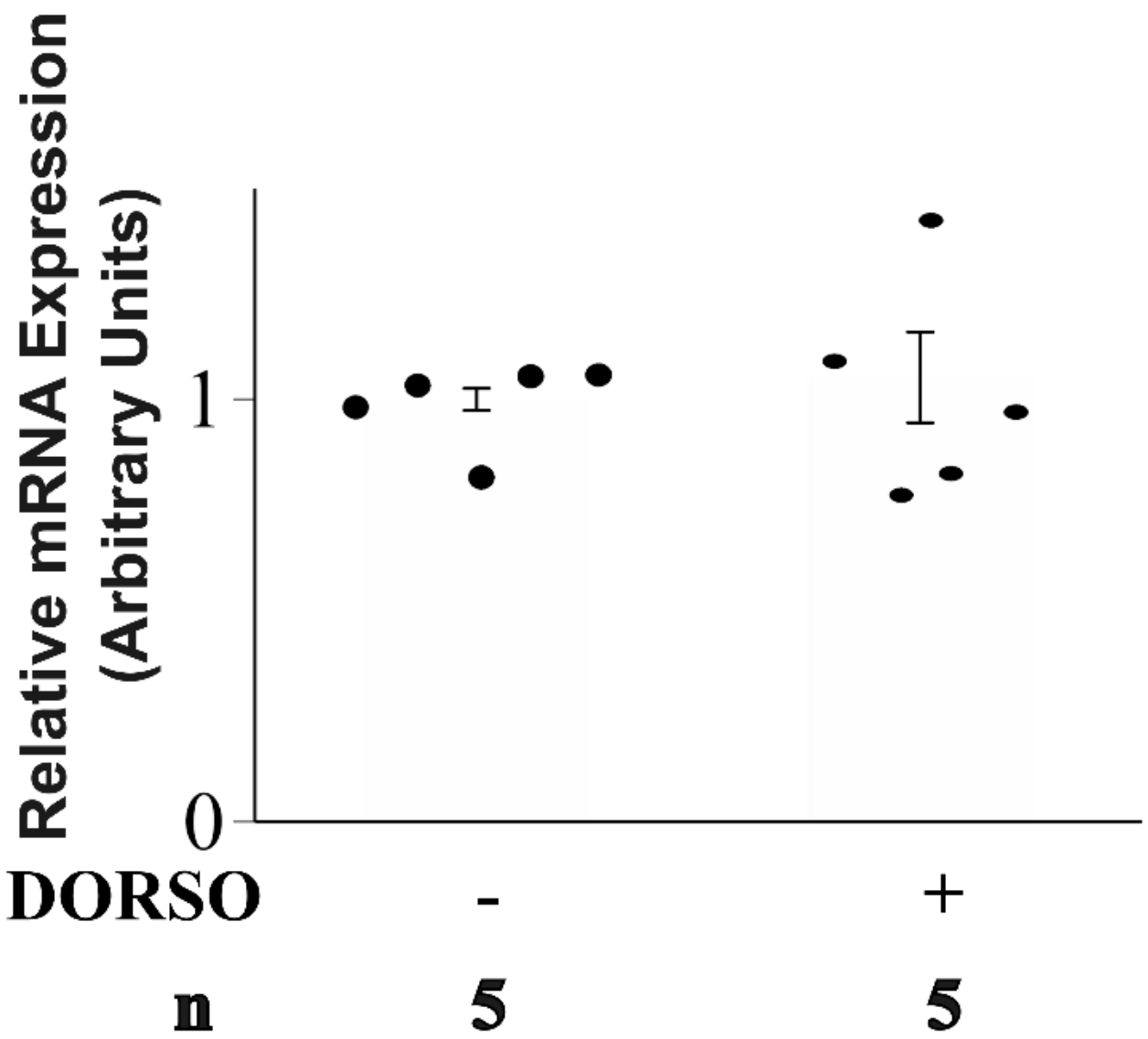
Relative mRNA Expression
(Arbitrary Units)
1
0
DORSO
-
+
n
5
5

V

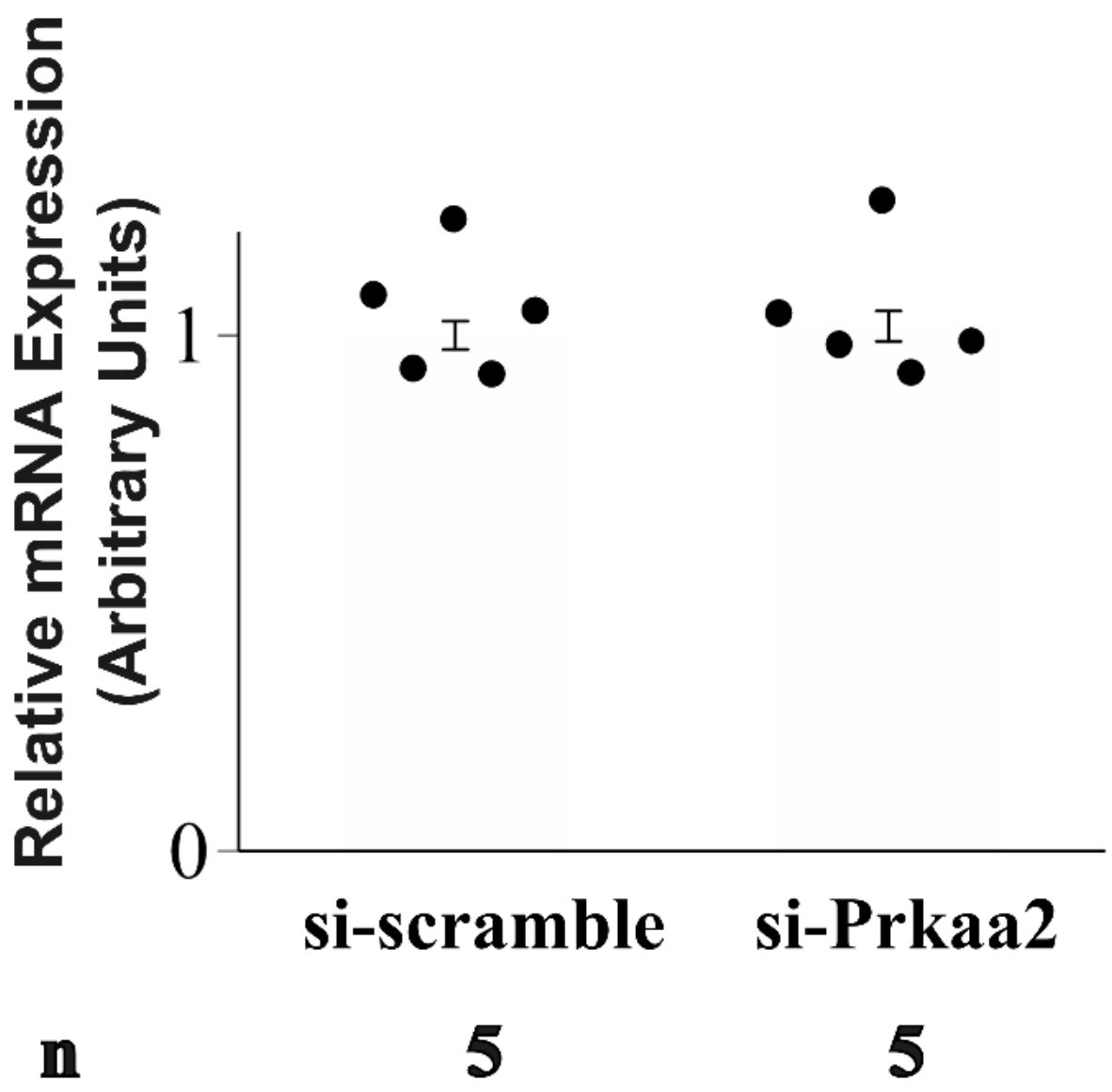
Relative mRNA Expression
(Arbitrary Units)
1
0
si-scramble
si-Prkaa2
n
5
5

W

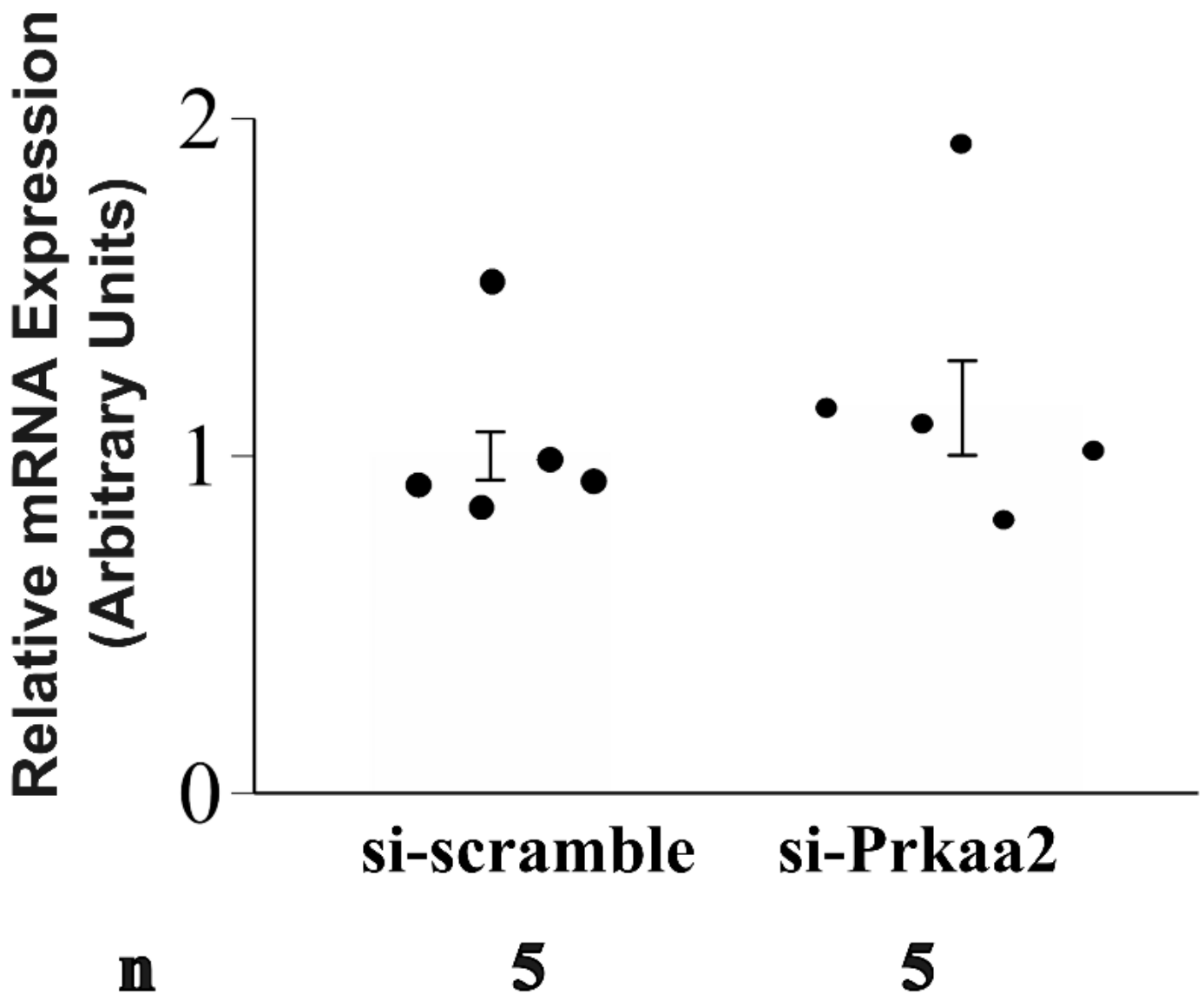
Relative mRNA Expression
(Arbitrary Units)
2
1
0
si-scramble
si-Prkaa2
n
5
5

X

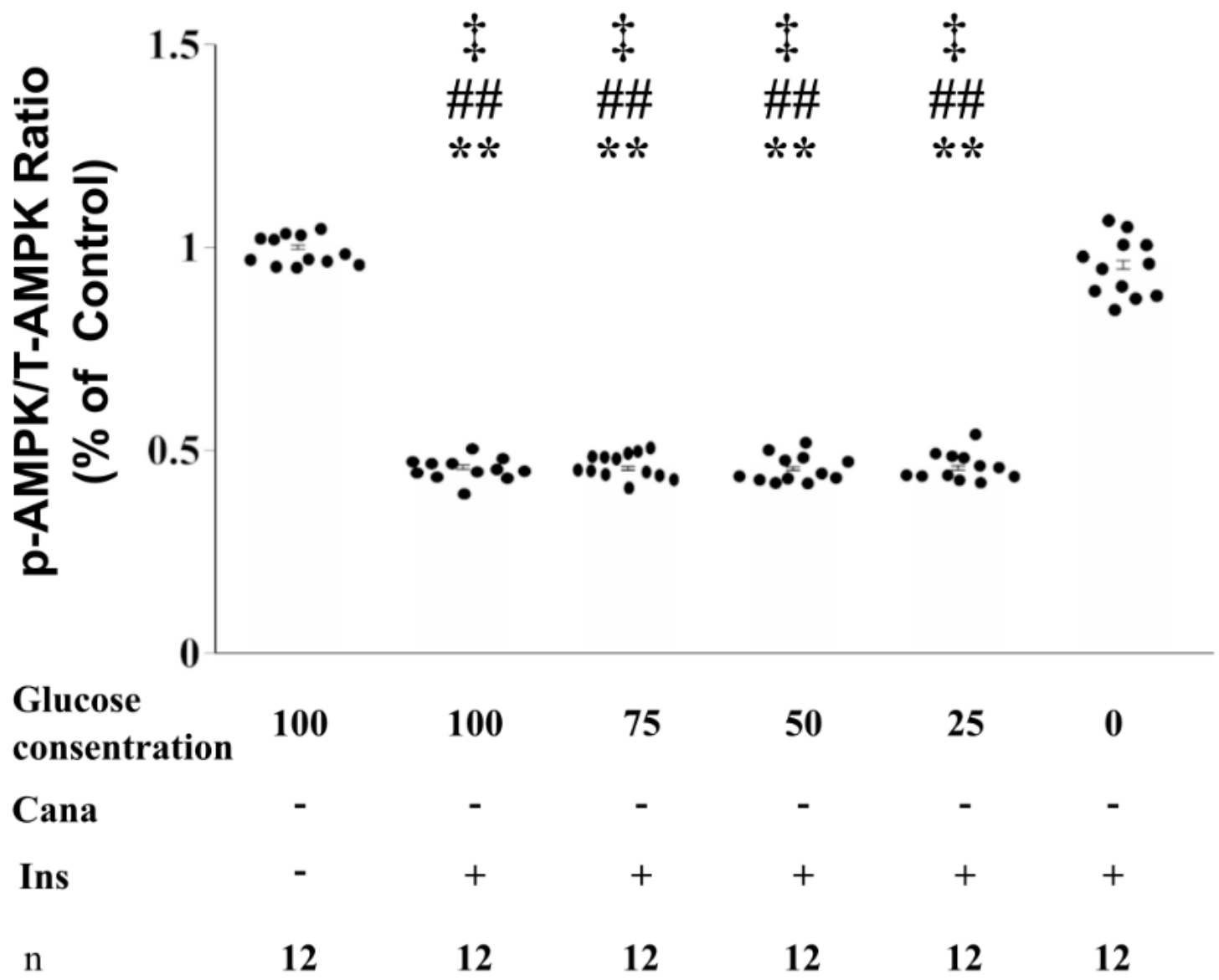
p-AMPK/T-AMPK Ratio (% of Control)
1.5
1
0.5
0
‡ ## **
Glucose consentration 100 100 75 50 25 0
Cana - - - - - -
Ins - + + + + +
n 12 12 12 12 12 12

Y

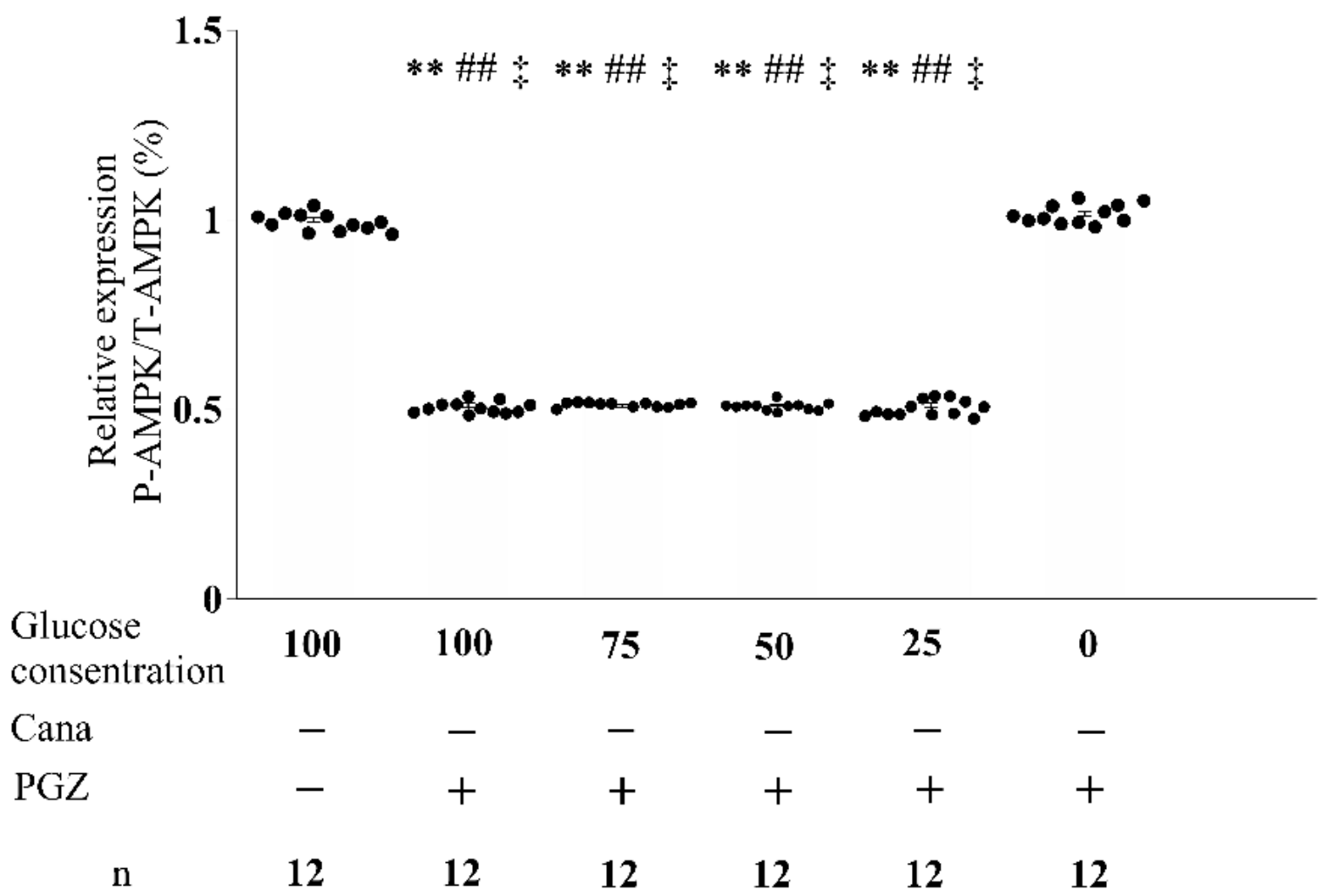
Relative expression P-AMPK/T-AMPK (%)
1.5
1
0.5
0
** ## ‡
Glucose consentration 100 100 75 50 25 0
Cana – – – – – –
PGZ – + + + + +
n 12 12 12 12 12 12

Z

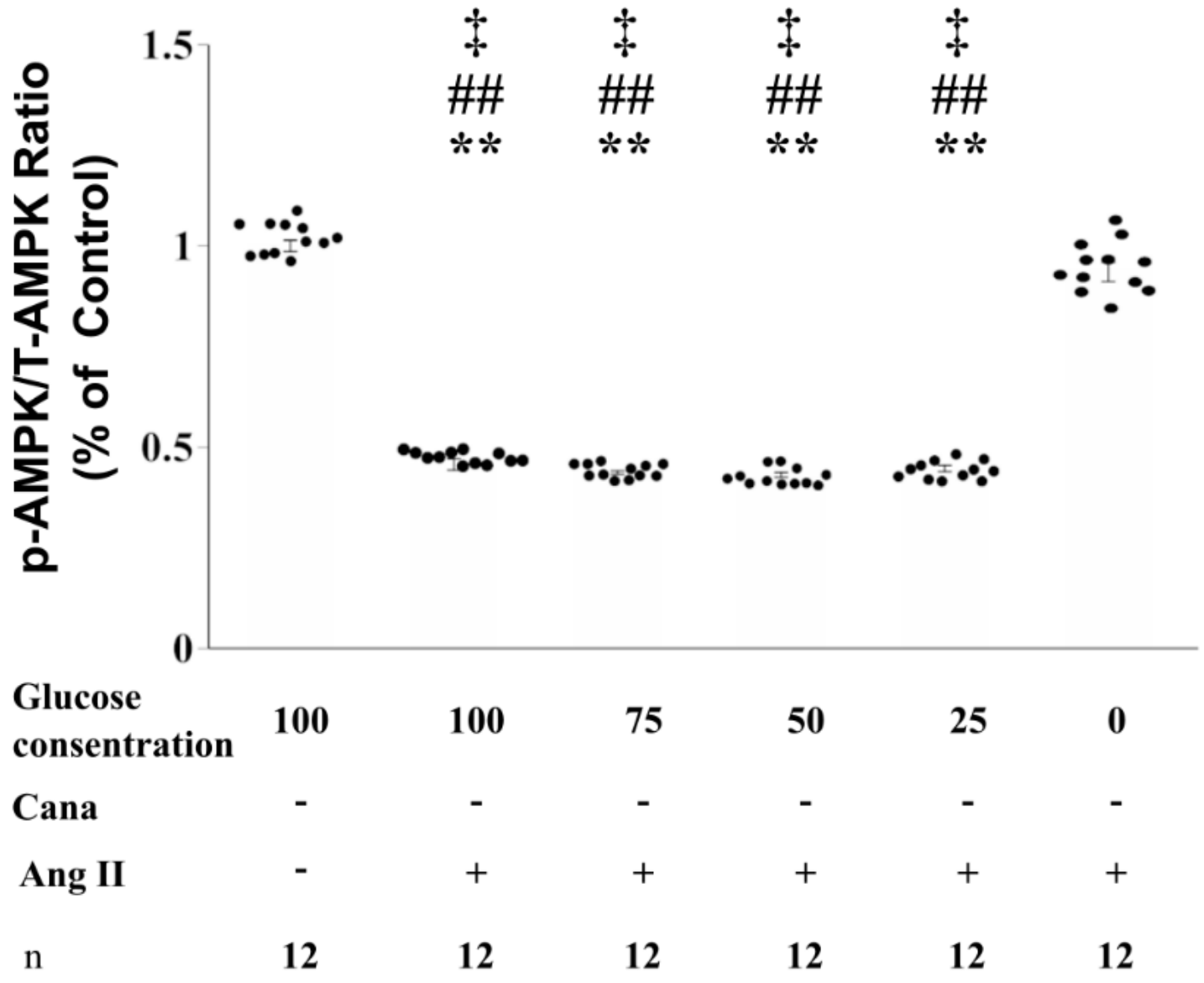

p-AMPK/T-AMPK Ratio (% of Control)
1.5
1
0.5
0
‡
##
**
Glucose consentration
100
100
75
50
25
0
Cana
-
Ang II
+
n
12

Supplementary Figure 5. Effects of AMPK inhibition and canagliflozin on proximal tubule Na^+ transport

A) Effect of 300 nM canagliflozin (Cana) on 1 nM insulin (Ins)-stimulated with-no-lysine kinase (WNK)1 mRNA expression in isolated rat PTs (*n* = 5). *p<0.05 versus untreated proximal tubules (PTs), #p<0.05 versus Cana-treated PTs.

B) Effect of 300 nM Cana on 0.3 μM PGZ-stimulated WNK1 mRNA expression in isolated rat PTs (*n* = 5). *p<0.05 versus untreated PTs, #p<0.05 versus Cana-treated PTs.

C) Effect of 300 nM Cana on 0.1 nM angiotensin II (AngII)-stimulated WNK1 mRNA expression in isolated rat PTs (*n* = 7). *p<0.05 versus untreated PTs, #p<0.05 versus Cana-treated PTs.

D) Modifications of *Prkaa1*/AMP-activated protein kinase (AMPK)α1 mRNA expression in PTs by stimulating factors.

E) Effects of Cana on AMPKα phosphorylation enhancement and suppression of Ins-dependent AMPKα phosphorylation in rat renal cortex (*n* = 8). **p<0.01 versus Ins-treated PTs.

F) Effects of Cana on AMPKα phosphorylation enhancement and suppression of PGZ-dependent AMPKα phosphorylation in rat renal cortex (*n* = 8). **p<0.01 versus PGZ-treated PTs.

G) Effects of Cana on AMPKα phosphorylation enhancement and suppression of AngII-dependent AMPKα phosphorylation in rat renal cortex (*n* = 8). **p<0.01 versus AngII-treated PTs.

H) Effect of 50 μM dorsomorphin (DORSO) on 1 nM Ins-stimulated NBCe1 activity in isolated rat PTs (*n* = 10). **p<0.01 versus untreated PTs, ##p<0.01 versus DORSO-treated PTs.

I) Effect of 50 μM DORSO on 0.3 μM pioglitazone (PGZ)-stimulated NBCe1 activity in isolated rat PTs (*n* = 10). **p<0.01 versus untreated PTs, ##p<0.01 versus DORSO-treated PTs.

J) Effect of 50 μM DORSO on 0.1 nM angiotensin II (AngII)-stimulated NBCe1 activity in isolated rat PTs (*n* = 10). **p<0.01 versus untreated PTs, ##p<0.01 versus DORSO-treated PTs.

K) Effect of 50 μM DORSO on 1 nM Ins-stimulated Na-H exchange transporter (NHE) activity in isolated rat PTs (*n* = 14). **p<0.01 versus untreated PTs, ##p<0.01 versus DORSO-treated PTs.

L) Effect of 50 μM DORSO on 0.3 μM PGZ-stimulated NHE activity in isolated rat PTs (*n* = 14). **p<0.01 versus untreated PTs, ##p<0.01 versus DORSO-treated PTs.

M) Effect of 50 µM DORSO on 0.1 nM AngII-stimulated NHE activity in isolated rat PTs (*n* = 14). **p<0.01 versus untreated PTs, ##p<0.01 versus DORSO-treated PTs.

N) Effect of canagliflozin and closantel on 1 nM Ins-stimulated NBCe1 activity in PTs with specific genetic suppression of *Prkaa2/AMPKα2* using siRNA (*n* = 10). **p<0.01 versus untreated PTs, ##p<0.01 versus *si-Prkaa2/AMPKα2* treated PTs, ‡p<0.01 versus *si-Prkaa2/AMPKα2* treated PTs with closantel and 1 nM Ins.

O) Effect of canagliflozin and closantel on the stimulatory effect of 0.3 µM PGZ on NBCe1 activity in PTs with specific genetic suppression of *Prkaa2/AMPKα2* using siRNA (n = 10). **p<0.01 versus untreated PTs, ##p<0.01 versus *si-Prkaa2/AMPKα2* treated PTs, ‡p<0.01 versus *si-Prkaa2/AMPKα2* treated PTs with closantel and 0.3 µM PGZ.

P) Effect of canagliflozin and closantel on the stimulatory effect of 0.1 nM AngII on NBCe1 activity in PTs with specific genetic suppression of *Prkaa2/AMPKα2* using siRNA (*n* = 10). **p<0.01 versus untreated PTs, ##p<0.01 versus si-Prkaa2/AMPKα2 treated PTs, ‡p<0.01 versus *si-Prkaa2/AMPKα2* treated PTs with closantel and 0.1 nM AngII.

Q) Effect of canagliflozin and closantel on the stimulatory effect of 1 nM Ins on NHE activity in PTs with specific genetic suppression of *Prkaa2/AMPKα2* using siRNA (*n* = 14). **p<0.01 versus untreated PTs, ##p<0.01 versus *si-Prkaa2/AMPKα2* treated PTs, ‡p<0.01 versus *si-Prkaa2/AMPKα2* treated PTs with closantel and 1 nM Ins.

R) Effect of canagliflozin and closantel on the stimulatory effect of 0.3 µM PGZ on NHE activity in PTs with specific genetic suppression of *Prkaa2/AMPKα2* using siRNA (*n* = 14). **p<0.01 versus untreated PTs, ##p<0.01 versus *si-Prkaa2/AMPKα2* treated PTs, ‡p<0.01 versus *si-Prkaa2/AMPKα2* treated PTs with closantel and 0.3 µM PGZ.

S) Effect of canagliflozin and closantel on the stimulatory effect of 0.1 nM AngII on NHE activity in PTs with specific genetic suppression of *Prkaa2/AMPKα2* using siRNA (n = 14). **p<0.01 versus untreated PTs, ##p<0.01 versus *si-Prkaa2/AMPKα2* treated PTs, ‡p<0.01 versus *si-Prkaa2/AMPKα2* treated PTs with closantel and 0.1 nM AngII.

T) Effect of 50 µM DORSO on WNK1 mRNA expression in isolated PTs (*n* = 5).

U) Effect of 50 µM DORSO on oxidative stress-responsive kinase 1 (OXSR1) mRNA expression in isolated PTs (*n* = 5).

V) Effect of *si-Prkaa2/AMPKα2* on WNK1 mRNA expression in isolated PTs (*n* = 5).

W) Effect of *si-Prkaa2/AMPKα2* on OXSR1 mRNA expression in isolated PTs (*n* = 5).

X) Effects of Ins-induced AMPK dephosphorylation in PTs cultured in medium containing different glucose concentrations (100, 75, 50, 25, or 0 mg/dL) with or without canagliflozin

**p<0.01 versus untreated PTs in normal glucose medium; ##p<0.01 versus 1 nM Ins treated PTs in glucose-free medium; ‡p<0.01 versus 1 nM Ins treated PTs with canagliflozin in glucose-free medium.

Y) Effects of PGZ-induced AMPK dephosphorylation in PTs cultured in medium containing different glucose concentrations (100, 75, 50, 25, or 0 mg/dL) with or without canagliflozin

**p<0.01 versus untreated PTs in normal glucose medium; ##p<0.01 versus 0.3 μM PGZ treated PTs in glucose-free medium; ‡p<0.01 versus 0.3 μM PGZ treated PTs with canagliflozin in glucose-free medium.

Z) Effects of AngII-induced AMPK dephosphorylation in PTs cultured in medium containing different glucose concentrations (100, 75, 50, 25, or 0 mg/dL) with or without canagliflozin

**p<0.01 versus untreated PTs in normal glucose medium; ##p<0.01 versus 0.1 nM AngII treated PTs in glucose-free medium; ‡p<0.01 versus 0.1 nM AngII treated PTs with canagliflozin in glucose-free medium.

A

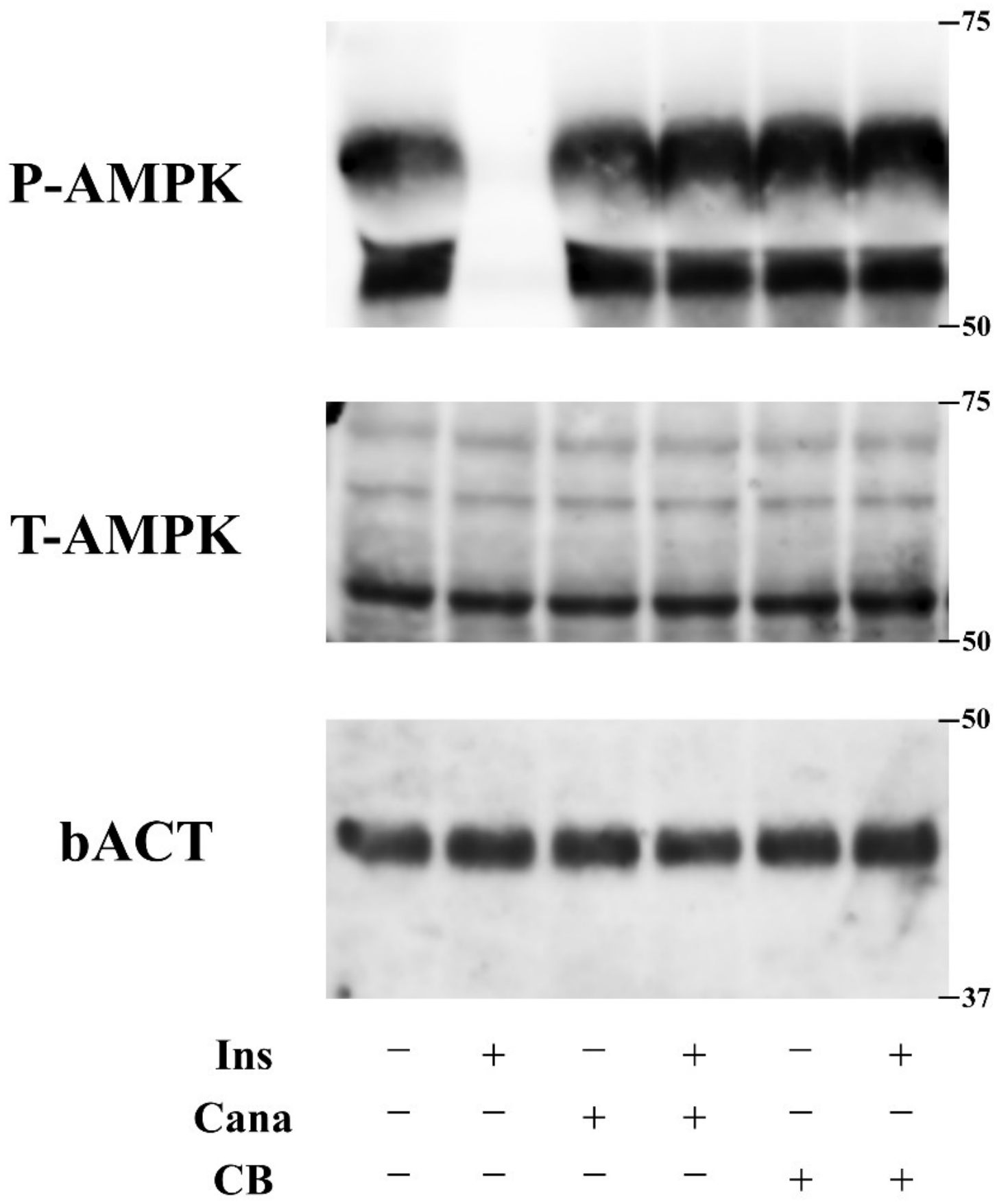
P-AMPK
T-AMPK
bACT
75
50
75
50
50
37
Ins − + − + − +
Cana − − + + − −
CB − − − − + +

B

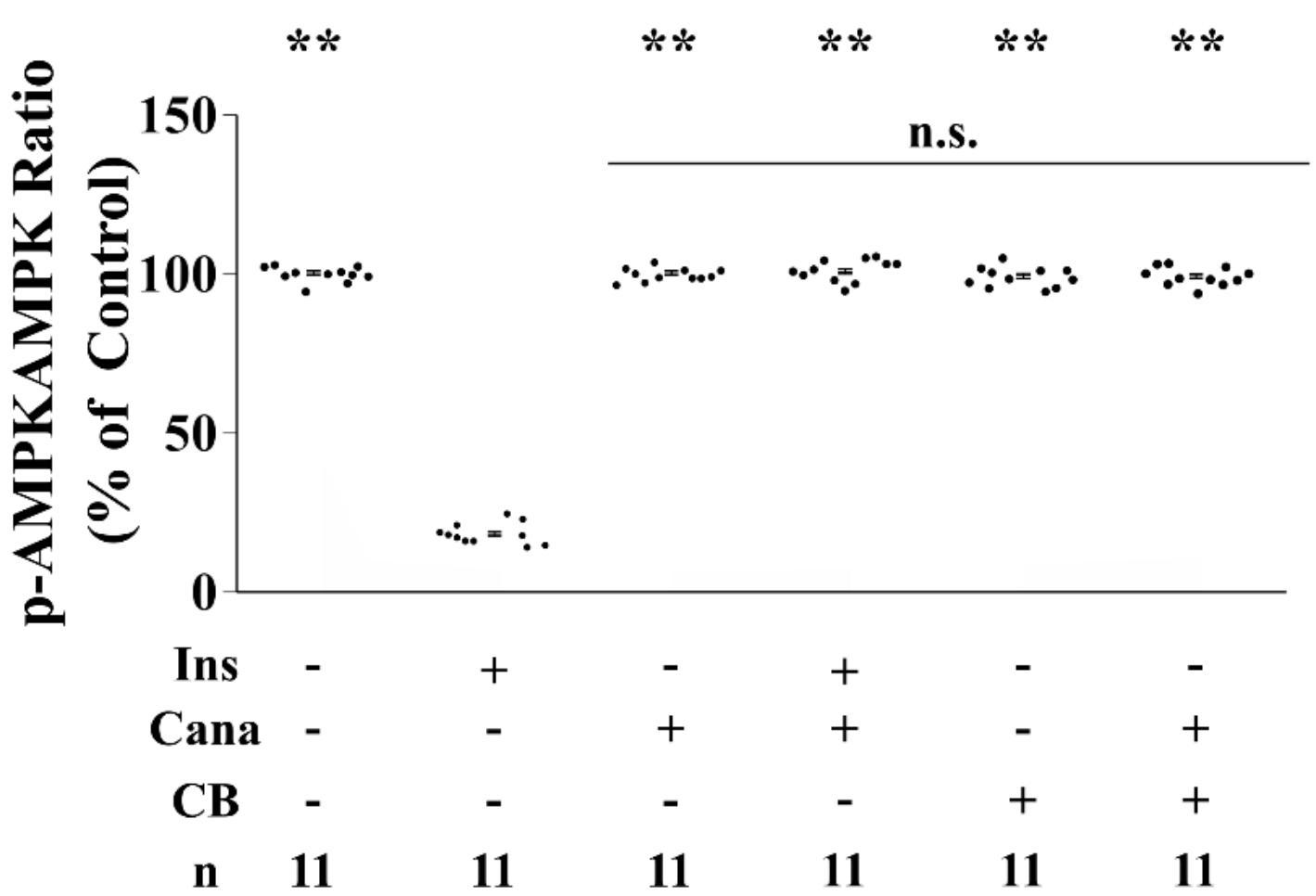
p-AMPKAMPK Ratio
(% of Control)
150
100
50
0
** ** ** ** **
n.s.
Ins - + - + - -
Cana - - + + - +
CB - - - - + +
n 11 11 11 11 11 11

Supplementary Figure 6. Effect of basolateral GLUT inhibition by Cytochalasin B on insulin-induced AMPK dephosphorylation.

A) Representative western blots and densitometric quantification showing the effect of Cytochalasin B on 1 nM insulin (Ins)-induced dephosphorylation of AMPKα1/2 in rat renal cortex.

B) Quantitative analysis of p-AMPK levels normalized to total AMPK in proximal tubules (PTs) (n = 11).. **$p < 0.01$ versus Ins-treated PTs.

A

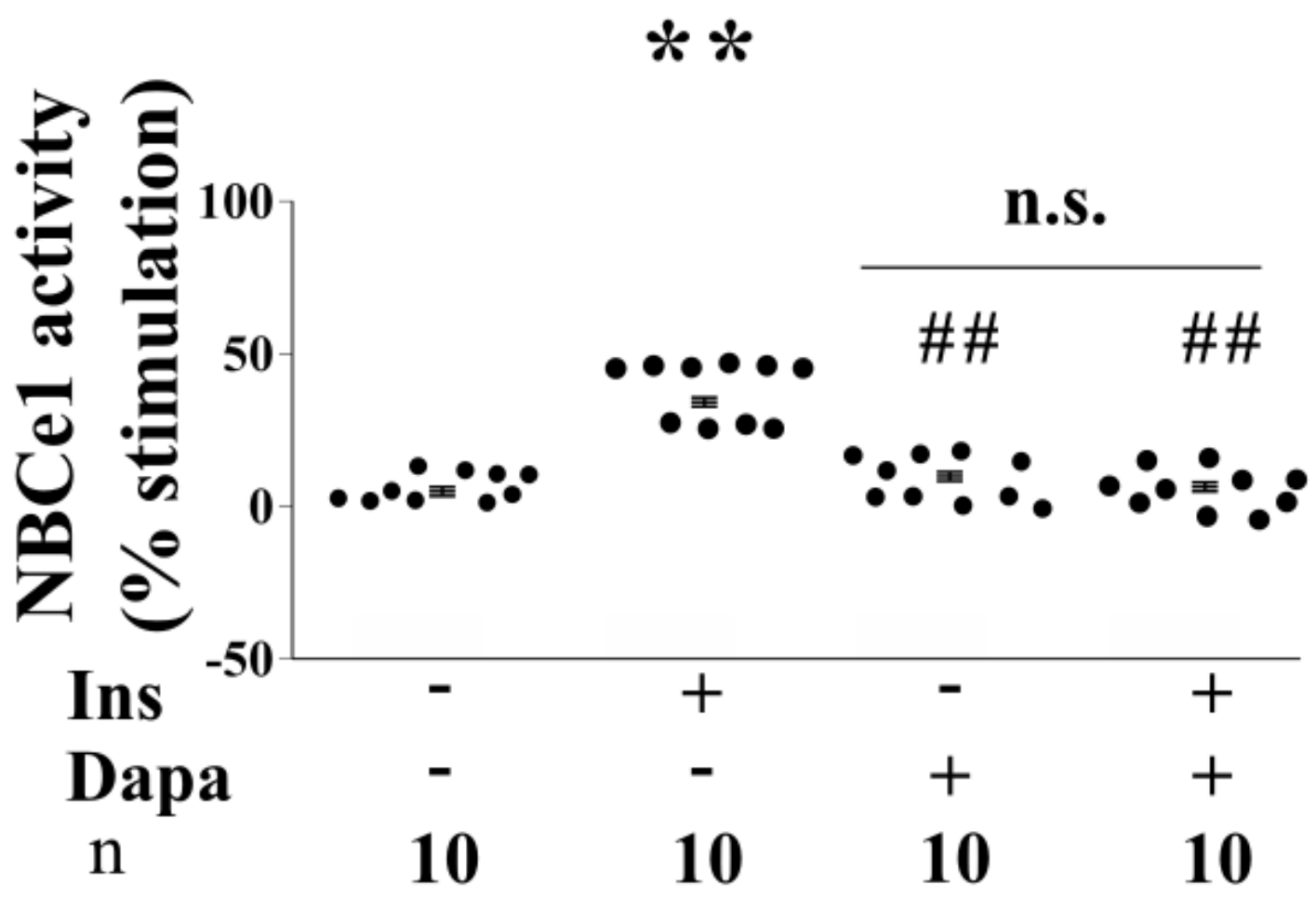

**
n.s.
##
##
NBCe1 activity
(% stimulation)
100
50
0
-50
Ins - + - +
Dapa - - + +
n 10 10 10 10

B

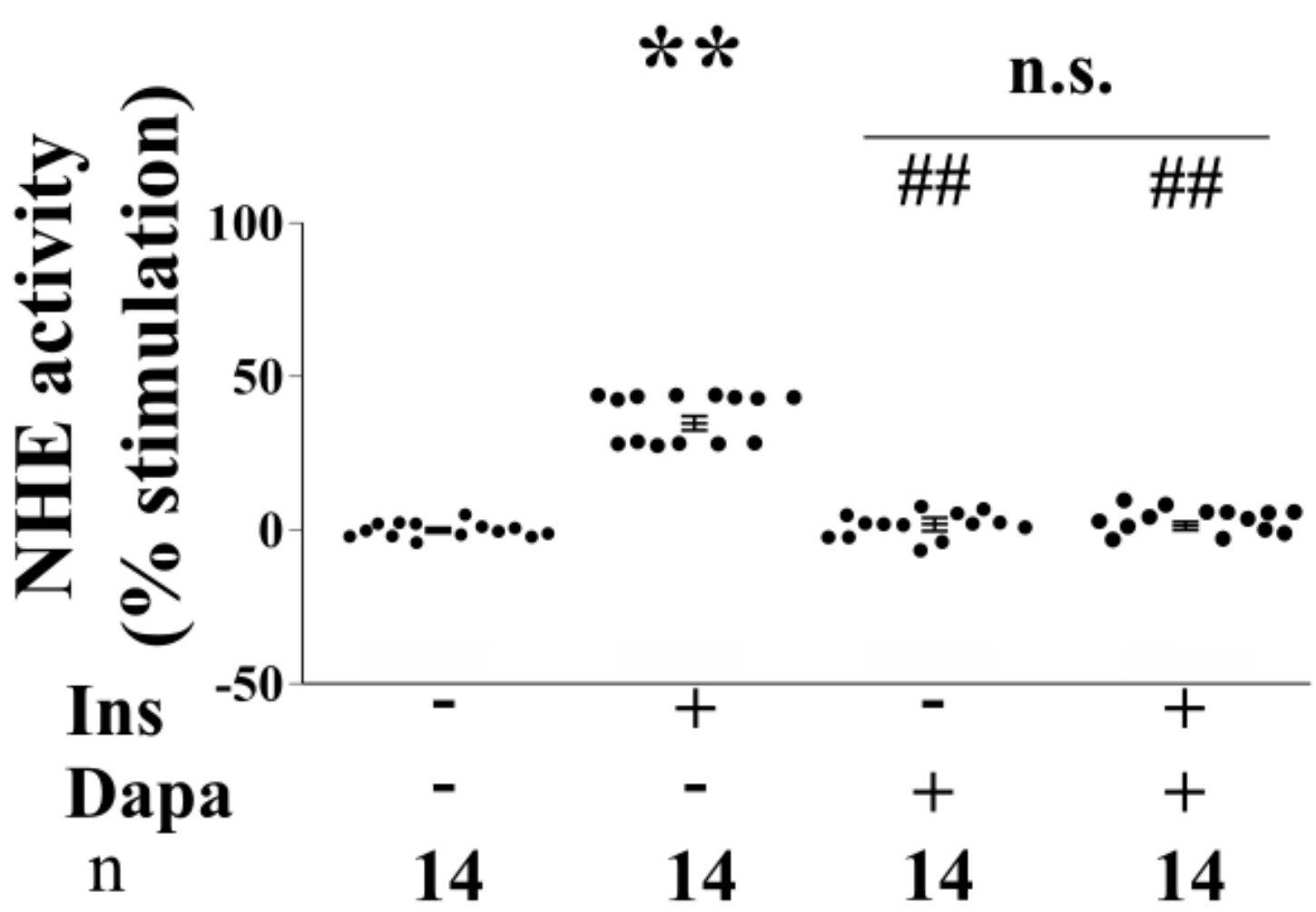

**
n.s.
##
##
NHE activity
(% stimulation)
100
50
0
-50
Ins - + - +
Dapa - - + +
n 14 14 14 14

C

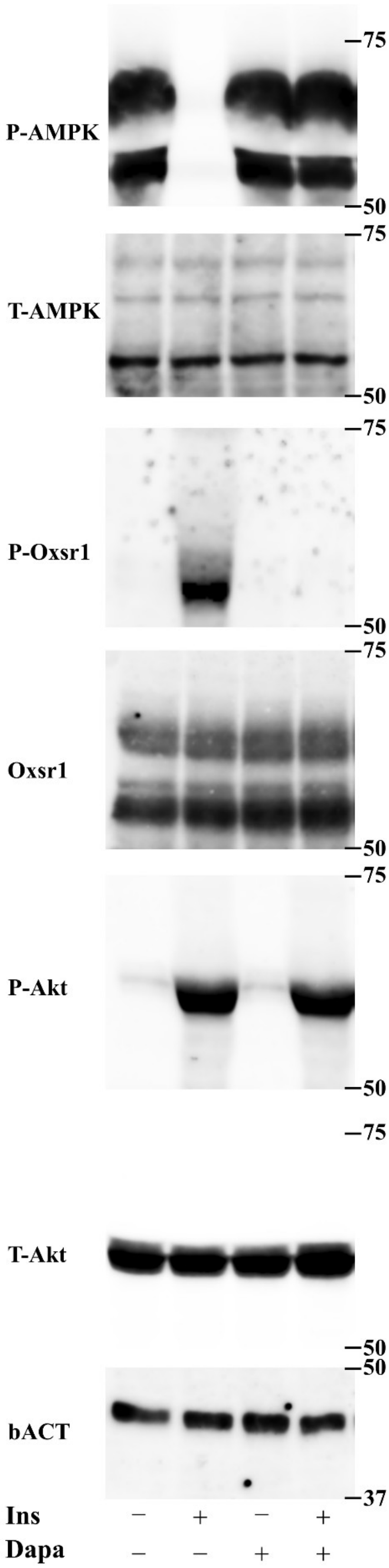
P-AMPK
T-AMPK
P-Oxsr1
Oxsr1
P-Akt
T-Akt
bACT
75
50
75
50
75
50
75
50
75
50
75
50
50
37
Ins – + – +
Dapa – – + +

D

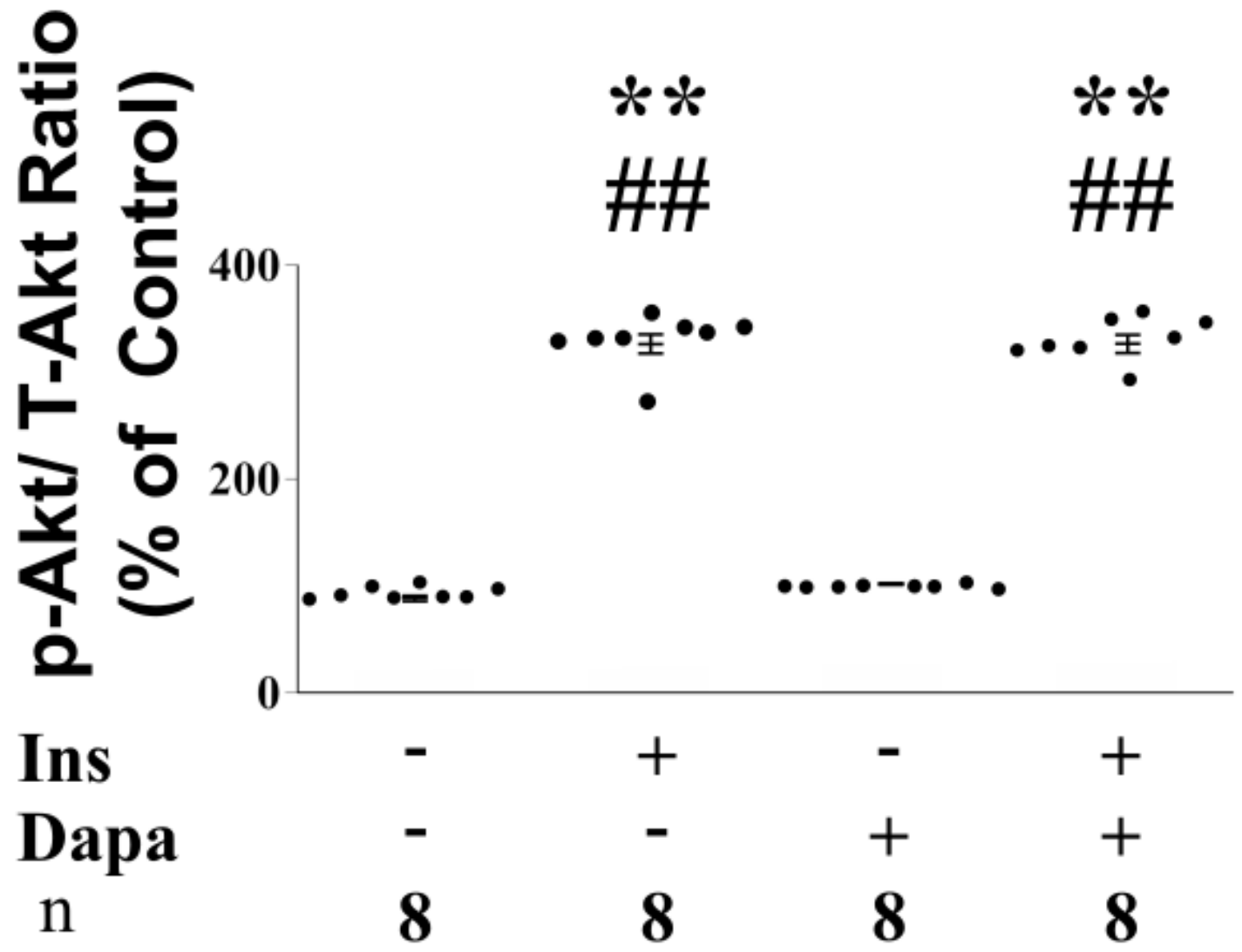
p-Akt/ T-Akt Ratio
(% of Control)
400
200
0
**
##
**
##
Ins - + - +
Dapa - - + +
n 8 8 8 8

E

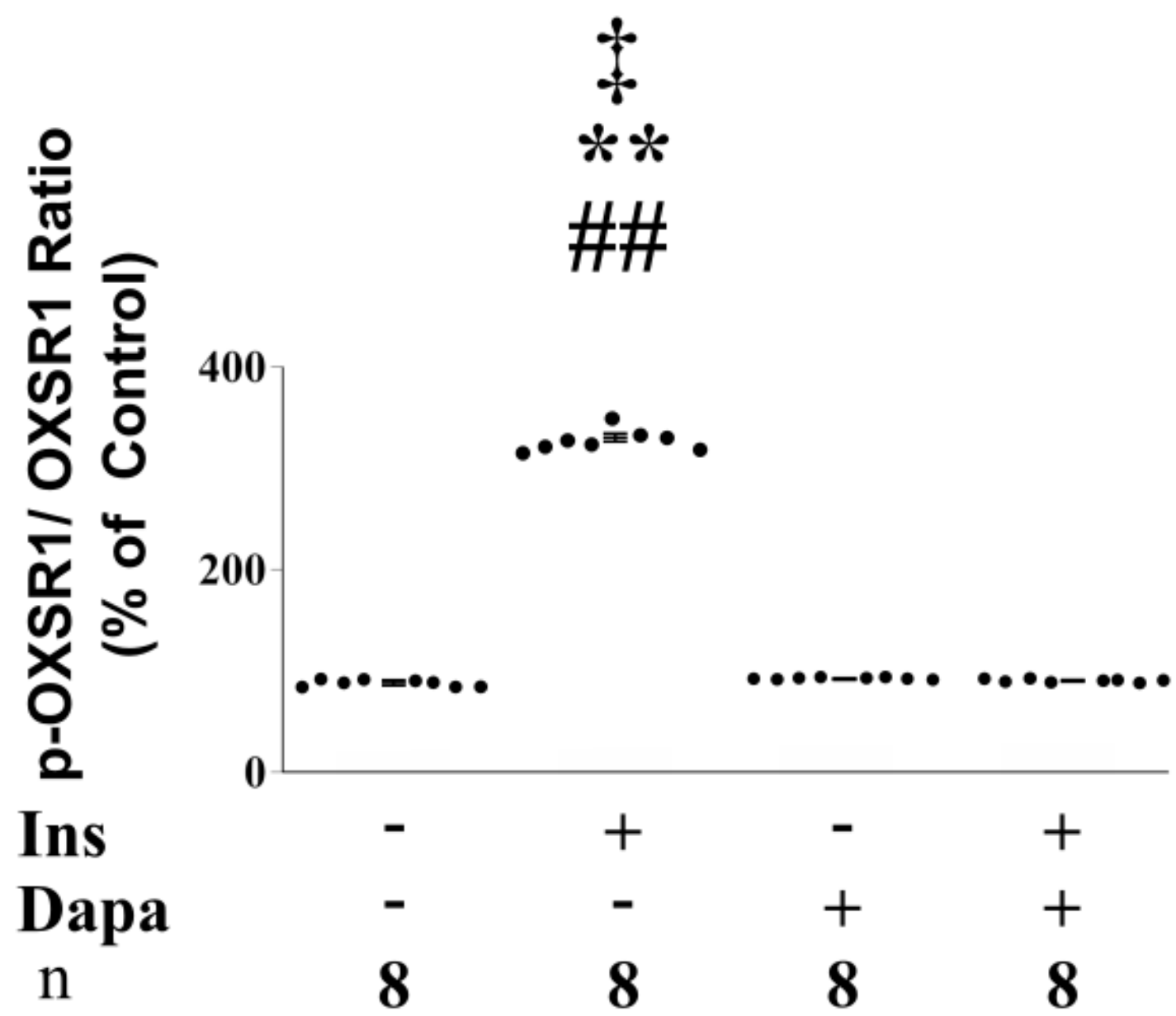
p-OXSR1/ OXSR1 Ratio
(% of Control)
400
200
0
‡
**
##
Ins - + - +
Dapa - - + +
n 8 8 8 8

F

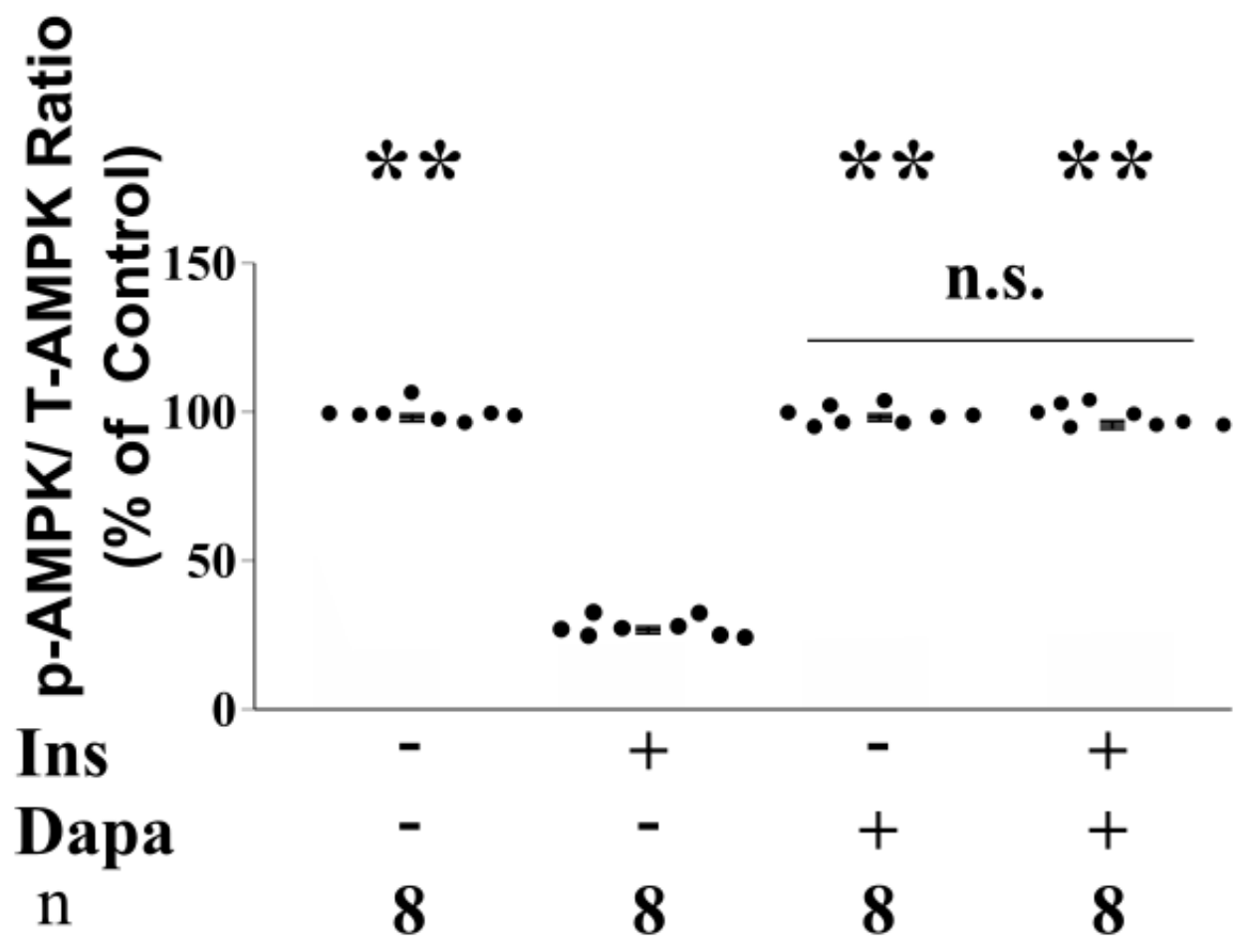

Supplementary Figure 7. Effects of the highly selective SGLT2 inhibitor dapagliflozin on insulin-stimulated sodium transport and AMPK phosphorylation in proximal tubules (PTs).

A) Effect of 1 nM insulin (Ins) and dapagliflozin (Dapa) on NBCe1 activity in isolated rat proximal tubules (PTs).

B) Effect of Dapa on 1 nM Ins-stimulated NHE activity in isolated rat PTs.

C) Representative western blots showing the effect of Dapa on insulin-induced dephosphorylation of AMPKα1/2 and AKT phosphorylation in rat renal cortex.

D) Insulin-induced AKT phosphorylation and the effect of Dapa on AKT phosphorylation in rat renal cortex (n = 8). **p<0.01 versus untreated PTs, ##p<0.01 versus Dapa-treated PTs.

E) Insulin-induced oxidative stress-responsive kinase 1 (OXSR1) phosphorylation and the effect of canagliflozin on OXSR1 phosphorylation in rat renal cortex (n = 8). **p<0.01 versus untreated PTs, ##p<0.01 versus Dapa-treated PTs, ‡ p<0.01 versus canagliflozin and insulin-treated PTs.

F) Ins-induced AMPKα1/2 dephosphorylation and the effect of Dapa on AMPKα1/2 phosphorylation in rat renal cortex (n = 8). **p<0.01 versus untreated PTs, ##p<0.01 versus Dapa-treated PTs, ‡ p<0.01 versus Dapa and insulin-treated PTs.

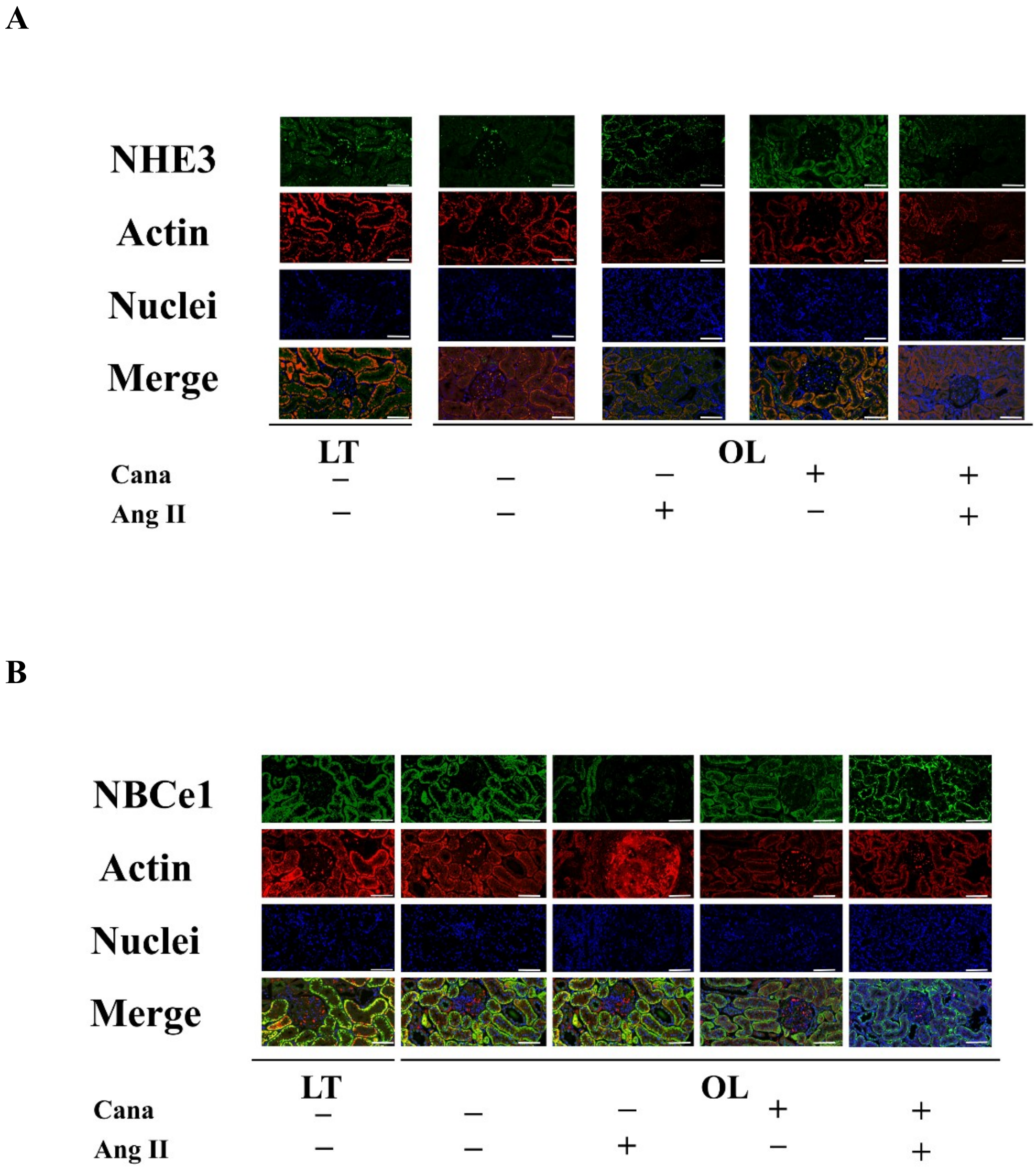
A
NHE3
Actin
Nuclei
Merge
LT
OL
Cana
Ang II
B
NBCe1
Actin
Nuclei
Merge
LT
OL
Cana
Ang II

C

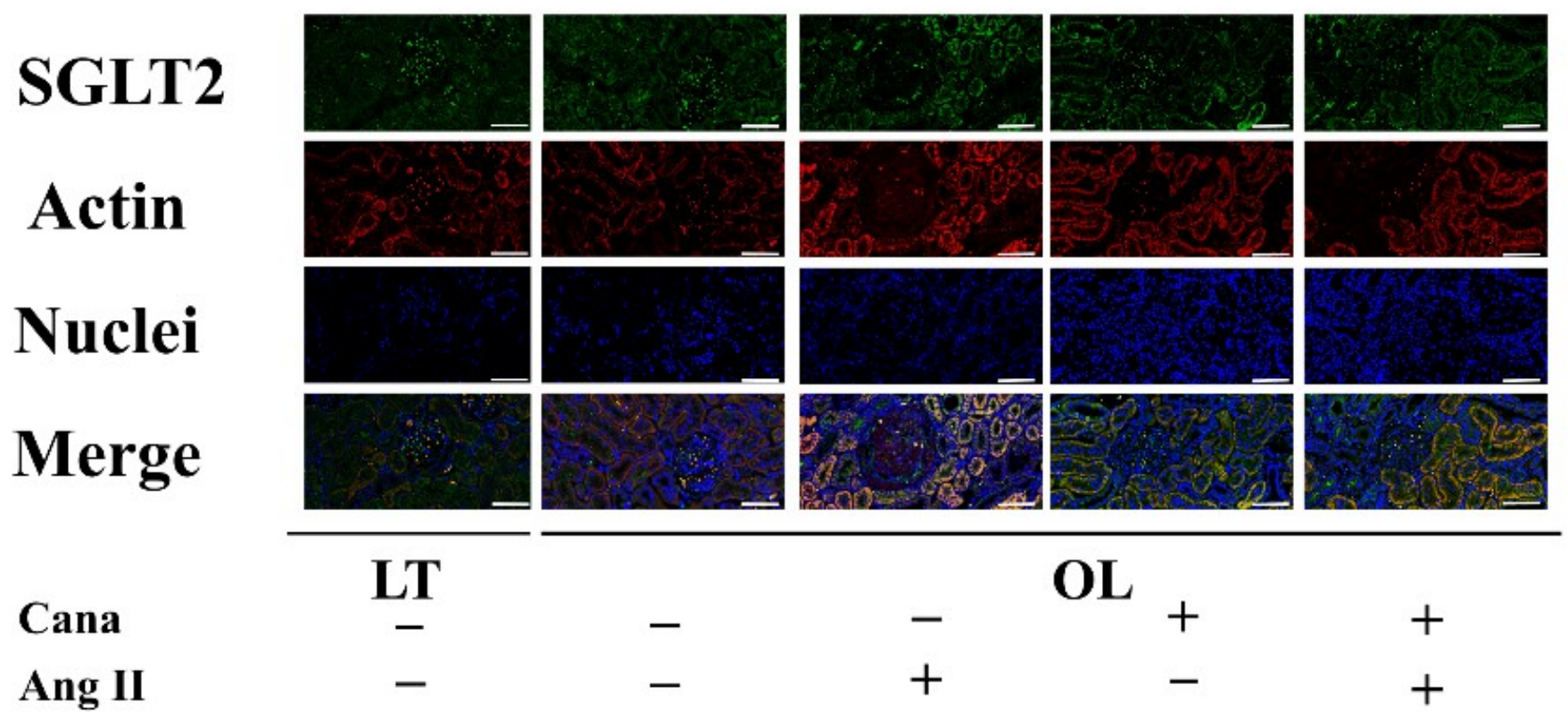
SGLT2
Actin
Nuclei
Merge
LT
OL
Cana – – – + +
Ang II – – + – +

D

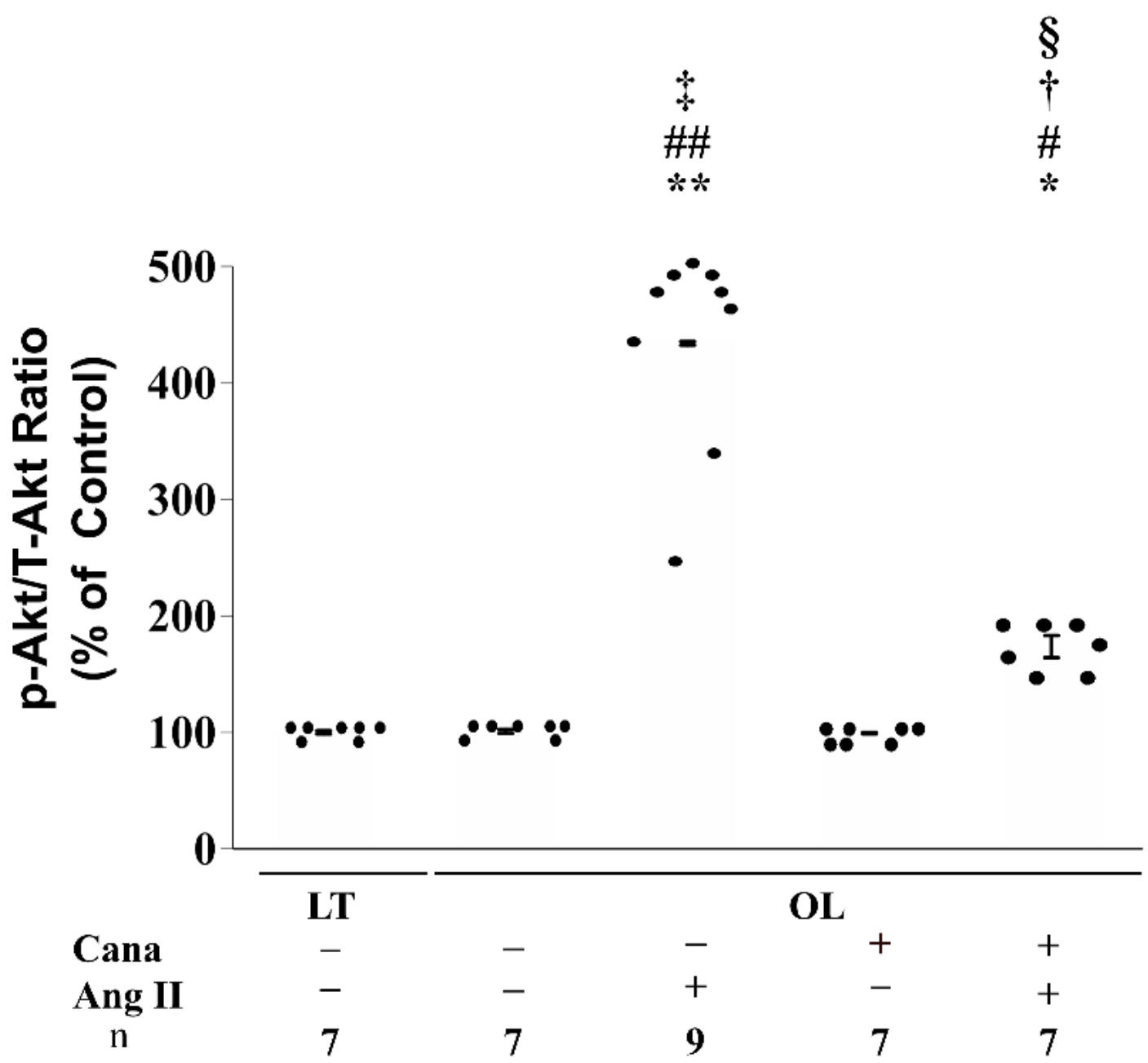
‡
##
**
§
†
#
*
p-Akt/T-Akt Ratio (% of Control)
500
400
300
200
100
0
LT
OL
Cana – – – + +
Ang II – – + – +
n 7 7 9 7 7

E

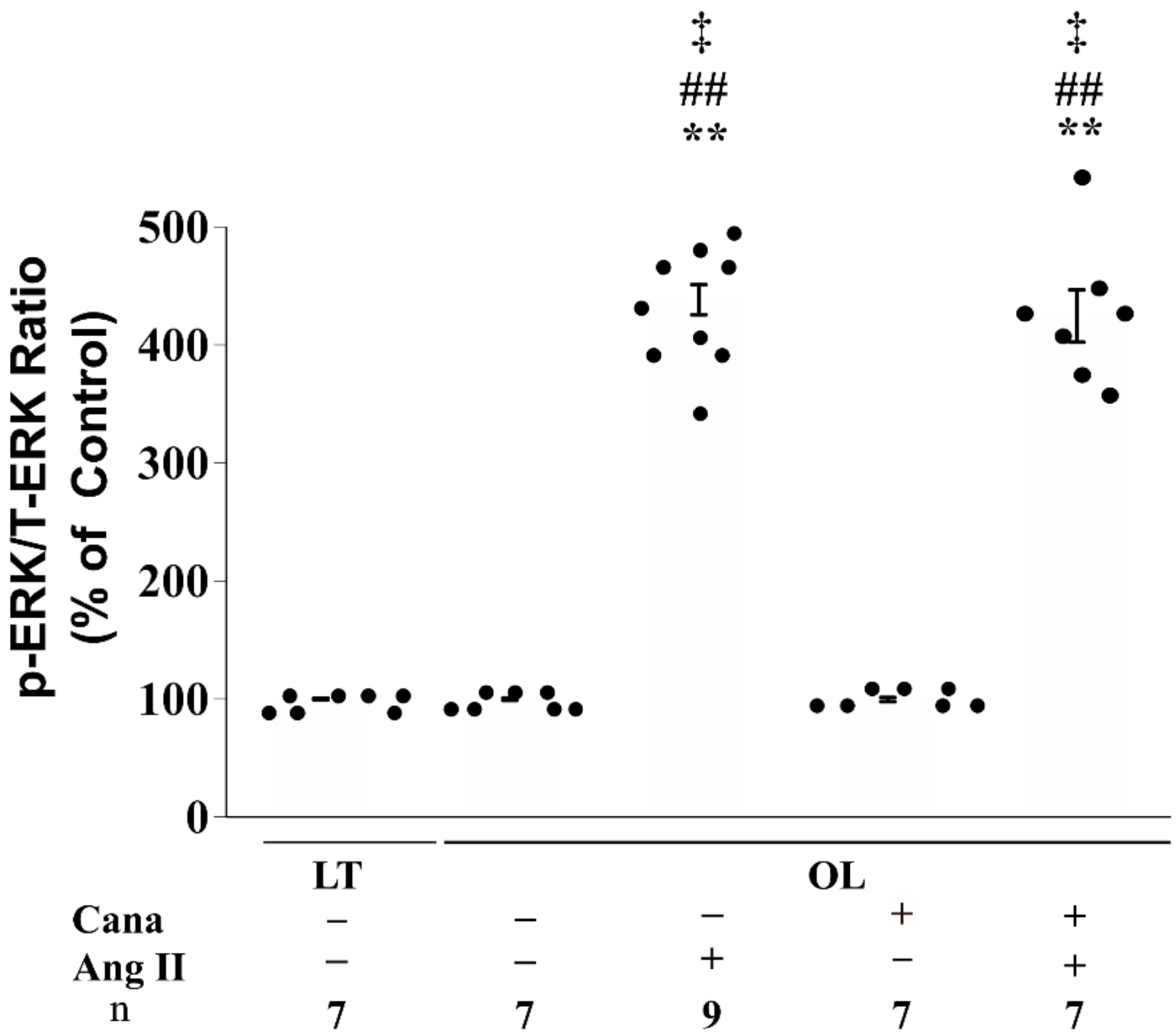
‡
##
**
‡
##
**
500
400
300
200
100
0
p-ERK/T-ERK Ratio
(% of Control)
LT
OL
Cana – – – + +
Ang II – – + – +
n 7 7 9 7 7

F

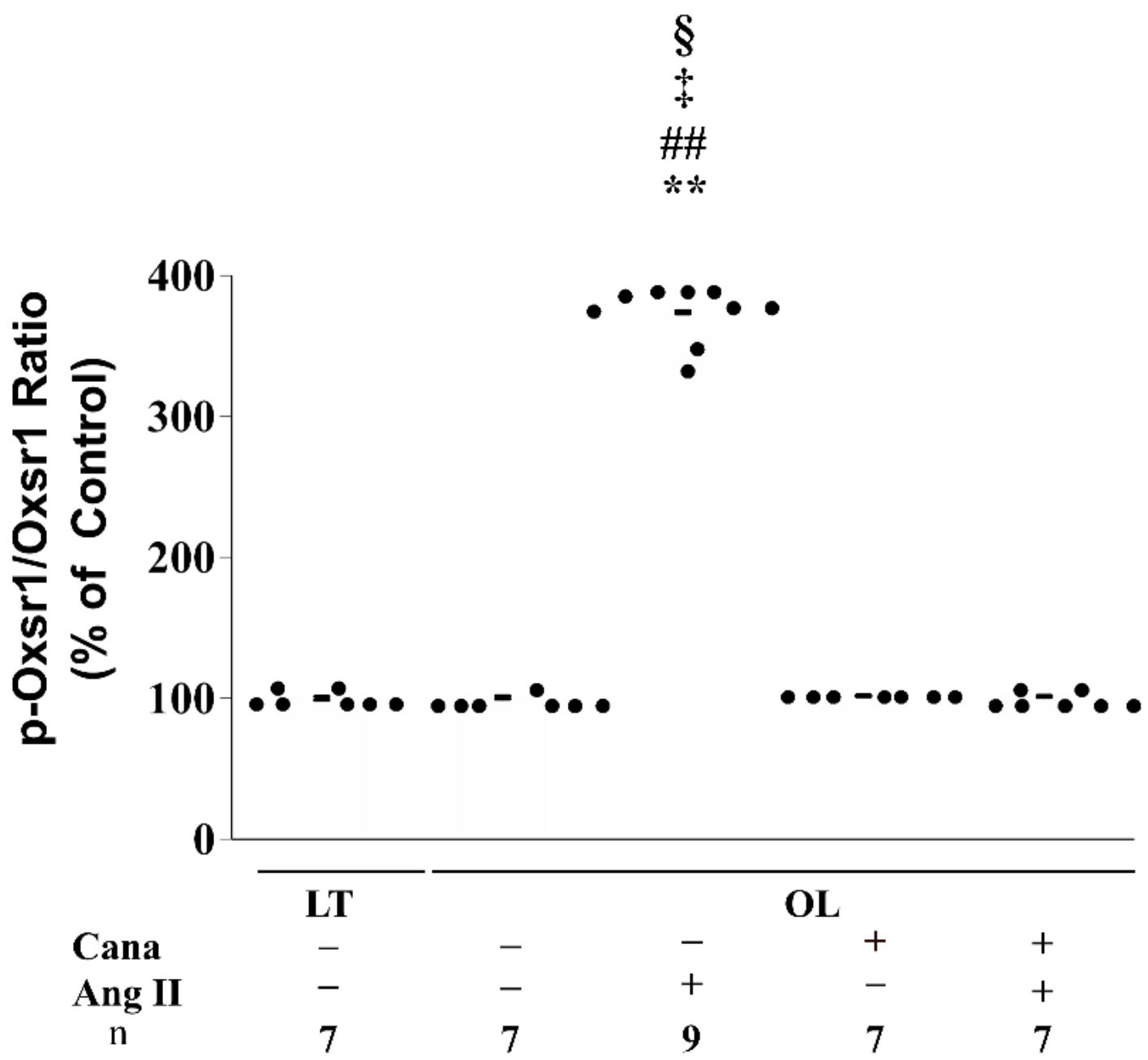
§
‡
##
**
400
300
200
100
0
p-Oxsr1/Oxsr1 Ratio
(% of Control)
LT
OL
Cana – – – + +
Ang II – – + – +
n 7 7 9 7 7

G

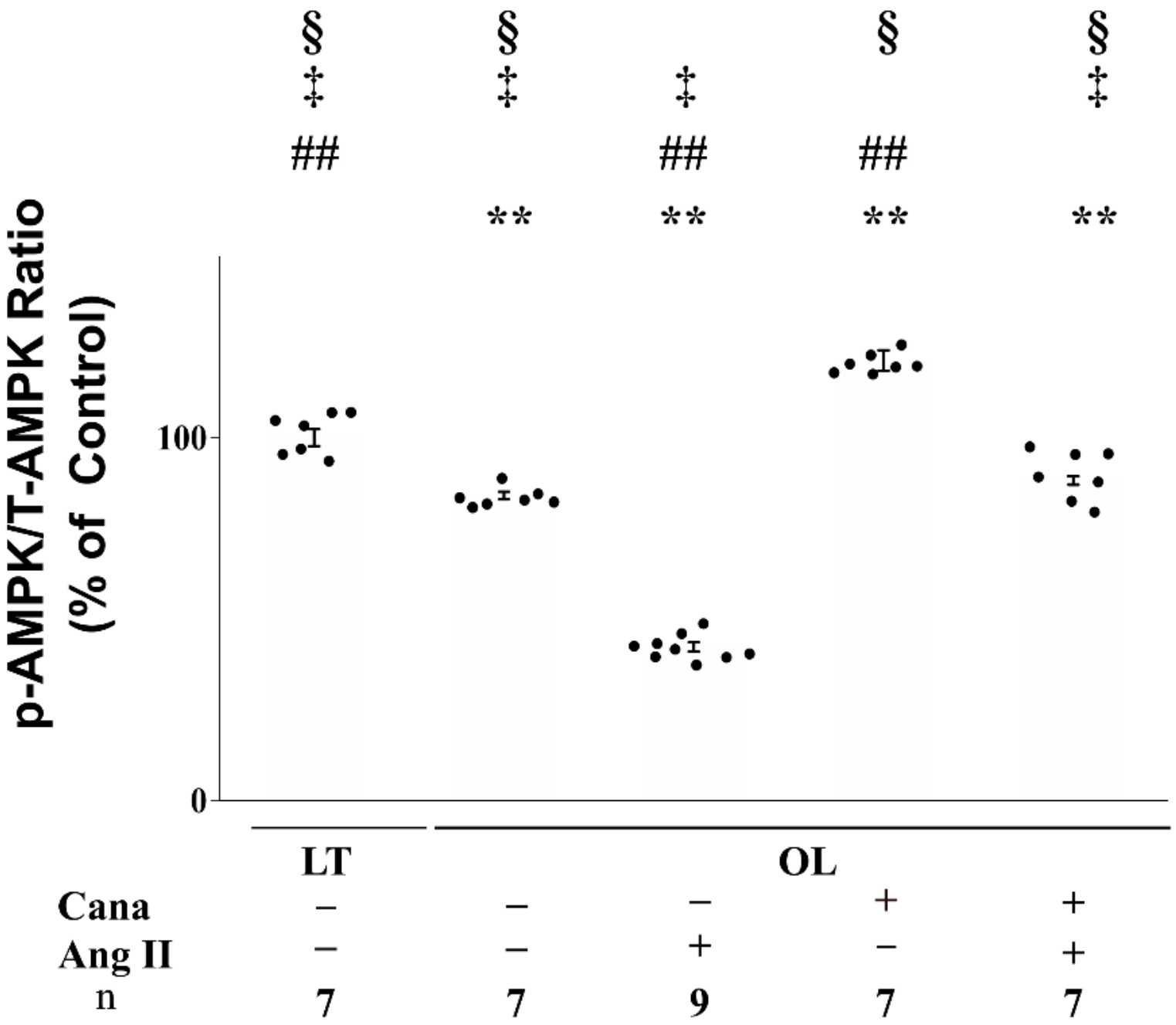

Supplementary Figure 8. Effect of canagliflozin in *in vivo* experiments

A) Immunofluorescence localization of Na^+-H^+ exchange transporter (NHE) 3 in the rat renal cortex. Representative confocal images showing NHE3 (green) and F-actin (red) in the proximal tubules (PTs) of rat renal cortex. F-actin was counterstained with phalloidin. The bottom panel shows the merged image with DAPI-stained nuclei (blue). Scale bar, 100 μm.

B) Immunofluorescence localization of Na^+-HCO_3- cotransporter (NBCe1) in the rat renal cortex. Representative confocal images showing NBCe1 (green) and F-actin (red) in the PTs of rat renal cortex. F-actin was counterstained with phalloidin. The bottom panel shows the merged image with DAPI-stained nuclei (blue). Scale bar, 100 μm.

C) Immunofluorescence localization of SGLT2 in the rat renal cortex. Representative confocal images showing SGLT2 (green) and F-actin (red) in the PTs of rat renal cortex. F-actin was counterstained with phalloidin. The bottom panel shows the merged image with DAPI-stained nuclei (blue). Scale bar, 100 μm.

D) Protein expression in the renal cortex *in vivo*; AKT phosphorylation in rat renal cortex *in vivo* and the effect of canagliflozin on AKT phosphorylation (n = 7–9).

E) Protein expression in the renal cortex *in vivo*; ERK phosphorylation in the rat renal cortex *in vivo* and the effect of canagliflozin on ERK phosphorylation (n = 7–9).

F) Protein expression in the renal cortex *in vivo*; OXSR1 phosphorylation expression in the rat renal cortex *in vivo* and the effect of canagliflozin on OXSR1 phosphorylation (n = 7–9).

G) Protein expression in the renal cortex *in vivo*; AMPKα phosphorylation expression in the rat renal cortex *in vivo,* and the effect of canagliflozin on AMPKα phosphorylation (n = 7–9).

*$p<0.05$ versus LETO, **$p<0.01$ versus LETO, $^{\#}p<0.05$ versus OLETF, $^{\#\#}p<0.01$ versus OLETF, †$p<0.05$ versus OLETF treated with canagliflozin, ‡ $p<0.01$ versus OLETF treated with canagliflozin, § $p<0.01$ versus OLETF administered continuous AngII.

3 Supplementary Tables

Supplementary Table 1. Profiles of human participants

Number	14
Age (years)	68.0 ± 9.3
Gender (% male)	78.5
Fasting plasma glucose (mg/dL)	91.2 ± 9.3
HbA1c (%)	5.8 ± 0.5
BMI (kg/m^2)	21.2 ± 2.9
Serum creatinine (mg/dL)	0.95 ± 0.31
eGFR (mL/min/1.73 m^2)	61.7 ± 16.7

Data are presented as means ± standard deviations. HbA1c, hemoglobin A1c; BMI, body mass index; eGFR, estimated glomerular filtration rate.

4 Supplementary references

S1. Nakamura M, Tsukada H, Seki G, Satoh N, Mizuno T, Fujii W, et al. Insulin promotes sodium transport but suppresses gluconeogenesis via distinct cellular pathways in human and rat renal proximal tubules. Kidney Int (2020) 97:316–326. doi: 10.1016/j.kint.2019.08.021

S2. Endo Y, Suzuki M, Yamada H, Horita S, Kunimi M, Yamazaki O, et al. Thiazolidinediones enhance sodium-coupled bicarbonate absorption from renal proximal tubules via PPARγ-dependent nongenomic signaling. Cell Metab (2011) 13:550–561. doi: 10.1016/j.cmet.2011.02.015

S3. Mizuno T, Satoh N, Horita S, Tsukada H, Takagi M, Sato Y, et al. Oxidized alkyl phospholipids stimulate sodium transport in proximal tubules via a nongenomic PPARγ-dependent pathway. J Biol Chem (2022) 298:101681. doi: 10.1016/j.jbc.2022.101681

S4. Shirai A, Yamazaki O, Horita S, Nakamura M, Satoh N, Yamada H, et al. Angiotensin II dose-dependently stimulates human renal proximal tubule transport by the nitric oxide/guanosine 3′,5′-cyclic monophosphate pathway. J Am Soc Nephrol (2014) 25:1523–1532. doi: 10.1681/ASN.2013060596

S5. Satoh N, Yamada H, Yamazaki O, Suzuki M, Nakamura M, Suzuki A, et al. A pure chloride channel mutant of CLC-5 causes Dent's disease via insufficient V-ATPase activation. Pflugers Arch (2016) 468:1183–1196. doi: 10.1007/s00424-016-1808-7

S6. Nakamura M, Satoh N, Tsukada H, Mizuno T, Fujii W, Suzuki A, et al. Stimulatory effect of insulin on H^+-ATPase in the proximal tubule via the AKT/mTORC2 pathway. Physiol Int (2020) 107:376–389. doi: 10.1556/2060.2020.00030

S7. Morishita K, Watanabe K, Naguro I, Ichijo H. Sodium ion influx regulates the liquidity of biomolecular condensates in hyperosmotic stress response. Cell Rep (2023) 42:112315. doi: 10.1016/j.celrep.2023.112315

S8. Chopra I, Li HF, Wang H, Webster KA. Phosphorylation of the insulin receptor by AMP-activated protein kinase (AMPK) promotes ligand-independent activation of the insulin signalling pathway in rodent muscle. Diabetologia (2012) 55:783–794. doi: 10.1007/s00125-011-2407-y

S9. Reyes-Pardo H, Bautista R, Vargas-Robles H, Rios A, Sánchez D, Escalante B. Role of sodium/glucose cotransporter inhibition on a rat model of angiotensin II-dependent kidney damage. BMC Nephrol (2019) 20:292. doi: 10.1186/s12882-019-1490-z

S10. Raij L, Azar S, Keane W. Mesangial immune injury, hypertension, and progressive glomerular damage in Dahl rats. Kidney Int (1984) 26:137–143.
doi: 10.1038/ki.1984.147
